# Guidance of Navigating Honeybees by Learned Elongated Ground Structures

**DOI:** 10.3389/fnbeh.2018.00322

**Published:** 2019-01-15

**Authors:** Randolf Menzel, Lea Tison, Johannes Fischer-Nakai, James Cheeseman, Maria Sol Balbuena, Xiuxian Chen, Tim Landgraf, Julian Petrasch, Johannes Polster, Uwe Greggers

**Affiliations:** ^1^Institute of Biology, Freie Universität Berlin, Berlin, Germany; ^2^Fachbereich Biowissenschaften, Polytechnische Gesellschaft Frankfurt am Main, Institute für Bienenkunde, Goethe-Universität Frankfurt am Main, Frankfurt, Germany; ^3^Department of Anaesthesiology, Faculty of Medical and Health Science, The University of Auckland, Auckland, New Zealand; ^4^Laboratorio de Insectos Sociales, Departamento de Biodiversidad y Biología Experimental, Facultad de Ciencias Exactas y Naturales, Universidad de Buenos Aires, Instituto de Fisiología, Biología Molecular y Neurociencias (IFIBYNE), CONICET-Universidad de Buenos Aires, Buenos Aires, Argentina; ^5^Dahlem Center of Machine Learning and Robotics, Institute for Informatics, Freie Universität Berlin, Berlin, Germany

**Keywords:** navigation, sun compass, guiding landmarks, object recognition, ground structures, compass alignment

## Abstract

Elongated landscape features like forest edges, rivers, roads or boundaries of fields are particularly salient landmarks for navigating animals. Here, we ask how honeybees learn such structures and how they are used during their homing flights after being released at an unexpected location (catch-and-release paradigm). The experiments were performed in two landscapes that differed with respect to their overall structure: a rather feature-less landscape, and one rich in close and far distant landmarks. We tested three different forms of learning: learning during orientation flights, learning during training to a feeding site, and learning during homing flights after release at an unexpected site within the explored area. We found that bees use elongated ground structures, e.g., a field boundary separating two pastures close to the hive (Experiment 1), an irrigation channel (Experiment 2), a hedgerow along which the bees were trained (Experiment 3), a gravel road close to the hive and the feeder (Experiment 4), a path along an irrigation channel with its vegetation close to the feeder (Experiment 5) and a gravel road along which bees performed their homing flights (Experiment 6). Discrimination and generalization between the learned linear landmarks and similar ones in the test area depend on their object properties (irrigation channel, gravel road, hedgerow) and their compass orientation. We conclude that elongated ground structures are embedded into multiple landscape features indicating that memory of these linear structures is one component of bee navigation. Elongated structures interact and compete with other references. Object identification is an important part of this process. The objects are characterized not only by their appearance but also by their alignment in the compass. Their salience is highest if both components are close to what had been learned. High similarity in appearance can compensate for (partial) compass misalignment, and vice versa.

## Introduction

Elongated landscape features like edges of forests, hedgerows, rivers, roads or boundaries of fields are potentially salient landmarks because they keep essential components of their object properties beyond the area at which the animals may have perceived and learned them (Chan et al., 2012). Thus, they may be recognized as (partially) familiar and spatially related to the intended goal even at different locations and under different viewpoints. Since these objects stretch in a particular direction relative to a compass, they provide a directional component that together with their intrinsic polarity may support navigational tasks to be completed efficiently and reliably. Polarity of elongated landmarks result from view-dependent differences, e.g., at a forest edge. Two or more of such linear objects could potentially be bound together in a network of spatially extended objects characterizing many locations as unique in relation to many (or even any) other location. Following an elongated landmark may thus help the animal to reach a goal although the travel may even involve longer travel time and distances.

Navigating pigeons are known to be guided by roads (Guilford et al., 2004; Lipp et al., 2004); however, the effect may well depend on the structure of the overall landscape e.g., along the south-north stretching landscape and main roads of Italy, and may not be seen in other landscapes, e.g., Germany (Wiltschko et al., 2007; Schiffner et al., 2013). Bats are known to fly along fixed routes called “flyways” stretching along linear landscape elements (Heithaus et al., 1975) and integrate such flyways in their nightly navigation (Geva-Sagiv et al., 2015). Homing bumble bees (Osborne et al., 2013) and honeybees (Wolf et al., 2014) have also a tendency to follow field boundaries and other elongated ground structures. Young honeybees tend to fly along parallel linear mowing structures in an agricultural grassland on their first orientation flights (Degen et al., 2015). Honeybees have to learn the time-compensated sun compass in relation to the landscape structures. Elongated objects may be particularly important in this learning process. von Frisch and Lindauer (1954) discovered that bees read the sun compass direction from extended landmarks (edge of a forest) when the sky is overcast, an observation that was confirmed by Dyer and Gould (1981).

Here, we investigated how bees learn elongated landscape structures and which kind of memory results from this acquisition. These questions were studied under multiple test conditions in two different rural environments: a rather feature-less landscape and a landscape rich in local and far-distant features. The catch-and-release paradigm was applied to test the memory for these structures. Individually identified foraging bees familiar with the surroundings of the hive were trained to a feeding site and captured just as they were about to fly back to the hive. They were then transported to a release site within the explored area and equipped with a transponder that allowed the flight trajectory to be tracked by harmonic radar. Under these conditions the bee usually first performed a vector flight that resembled the direction and distance that would have brought it back to the hive had it not been transported to a remote release site. The vector flight was followed by a search flight and then by a straight flight leading back to the hive during the final homing performance (Menzel et al., 2005, 2011; Menzel, 2013). Bees are known to use multiple learned landscape features during homing, and learned elongated landscape structures are expected to be one of several sources of information. Thus, it is particularly important to evaluate under which conditions such landscape structures are acquired and used. We found that this depends on how prominent these features are, how they had been learned, and with which other guiding information they compete.

## Materials and Methods

### Experimental Areas

The experiments were performed at two rural locations termed area A and area B. Area A was a rather featureless, large open grass field close to Klein Lüben (Brandenburg, Germany, coordinates: N 52.97555, E 11.83677) displaying landmarks on the ground (patches of grass at different stages of growth, clover flowers, a boundary line formed between two meadows mown at different times, and two parallel irrigation channels). A row of bushes ran along the southern border of the test field. It was discernible over a distance of about 100 m to the north; the irrigation channels could be detected by the bees from approximately 30 m on both sides (Menzel and Greggers, 2015). Hives were located either at the boundary line in the middle of the field or 60 m to the east of the boundary line (Experiment 1), at an irrigation channel (Experiment 2), or at the row of bushes in the southwestern corner of the field (Experiment 3). The radar for tracking bees in flight was located at the southern edge of the field close to the row of bushes.

Area B was a highly structured agricultural landscape stretching to the east of the area scanned by the radar (50°48′51.9″N) with trees and bushes, pathways, creeks, and grass fields close to the village Großseelheim (Hessen, Germany, coordinates: 50°48′50.18″N, 8°52′21.01″E). The bee colony used for Experiments 4 and 5 was housed in a cabin close to the radar at the west edge of the study area and close to a gravel road running parallel to the east-facing edge of the village. The colony used for Experiment 6 was located at the southern edge of the study area at a distance of 520 m from the radar close to a road.

A realistic 3-dimensional virtual world was used to examine how the environment of area B appeared to the honeybee eye (Polster et al., 2018). A large 3-D world was reconstructed from aerial imagery and the imaging properties and distributions of ommatidia in both eyes were modeled following Giger (1996) and Stürzl et al. (2010). Using this model, panorama views were computed and used here to address the question whether the panorama at the three essential sites (hive, feeder, release site) contributed to the flight routes during homing. To quantify the amount of ground structure in each image, we calculated three metrics for sub-regions of the bee view, excluding all pixels above the horizon (Supplementary Figures S1–S7). The Michelson contrast was defined as the brightness difference of the brightest and darkest pixel, divided by their sum. Hence, the maximum Michelson contrast was 1.0, the lowest 0.0. The mean Michelson contrast over all given bee views is 0.54 ± 0.15. The Michelson contrast might be sensitive to outliers. We hence used the brightness standard deviation to estimate more robustly the image’s information content. Let the maximum pixel brightness be 1.0, and 0.0 the minimum. The average brightness over all bee views was 0.38 ± 0.03 and the mean standard deviation was 0.07 ± 0.03. The brightness variation can still be high in a strongly textured landscape with no elongated ground structures whatsoever. We therefore also counted the number of connected edge pixels using Canny’s edge detector (Canny, 1986). Edge pixels are defined as strong image gradients. The gradient direction is then used to decide whether edge pixels belong to the same image structure such as a road or field ridge.

### Radar Tracking

Tracking bees with a harmonic radar was achieved as previously described (Riley et al., 1996). Two harmonic radar set-ups were used. The one used in Experiment 1 was described in Riley et al. (1999, 2003), the one used in all other experiments was described in Menzel et al. (2005). In short, the technical details of the latter were as follows. The sending unit consisted of a 9.4 GHz radar transceiver (Raytheon Marine GmbH, Kiel, NSC 2525/7 XU) combined with a parabolic antenna providing approximately 44 dBi. The transponder fixed to the thorax of the bee consisted of a dipole antenna with a Low Barrier Schottky Diode HSCH-5340 of centered inductivity. The second harmonic component of the signal (18.8 GHz) was the target for the radar. The receiving unit consisted of an 18.8 GHz parabolic antenna, with a low-noise pre-amplifier directly coupled to a mixer (18.8 GHz oscillator), and a downstream amplifier with a 90 MHz ZF filter. A 60 MHz ZF signal was used for signal recognition. The transponder was 10.5 mg in weight and 11 mm in length. We used a silver or gold wire with a diameter of 0.33 mm and a loop inductance of 1.3 nH. Radar signals were updated every 3 s. The range of both harmonic radar systems was set to 0.5 nautic miles in most experiments. The improved performance of the radar system used in Experiments 4–6 allowed to set the range temporarily to 0.75 nm or 1.5 nm. The raw radar out-put was captured from the screen at a frequency of 1 Hz, stored as bitmap file and further analyzed off-line by a custom made program that detected and tracked radar signals, and converted circular coordinates into a Cartesian coordinates taking into account multiple calibration posts in the environment. Finally the radar signals were displayed in a calibrated geographic map created with the software Pix4D from aerial images (Strecha et al., 2012) taken with a commercial drone (DYI Inspire). If no signals were received from a bee for more than 30 s the flight trajectory was interrupted, and the last as well as the first signal before and after interruption was marked.

### Experimental Design

The respective colonies were placed at their locations at least 3 weeks prior to the start of the experiments ensuring that foragers were familiar with their respective landscapes. The experimental design followed the catch-and release procedure as applied in many of our previous navigation experiments (Menzel et al., 2005, 2011). A full protocol of all foragers coming to the feeder was established by marking each bee with a colored number tag. Single bees were captured in a small vial at the feeder when they prepared to return to the hive (“homing flight”). They were carried to a release site within the explored area in the dark and then released after a radar transponder had been fixed to their thorax. Usually, animals were tested for their homing flights only once, thus ensuring that they did not use any experience from previous homing flights. In Experiment 6, foragers were trained to a feeder close to the hive (5–10 m), caught when leaving the feeder and then transported to a release site as in the other experiments. Other than in the other experiments, these animals were released multiple times at different release sites and also tested in release sites where they were not released before.

### Statistics

The variance of circular data in Experiment 1 was analyzed by means of the Wheeler Watson test using Igor Pro 7 circular statistics^1^. The Fisher Exact Test was applied in Experiment 2 to examine the proportions of turns at the guiding landmark (irrigation channel) using STATISTICA (Statsoft, Inc., Tulsa, OK, USA). Circular data in Experiment 1 were analyzed used Oriana circular statistics^2^.

## Results

### Experiment 1: Hive Training, Field Boundary

**Figure 1:**
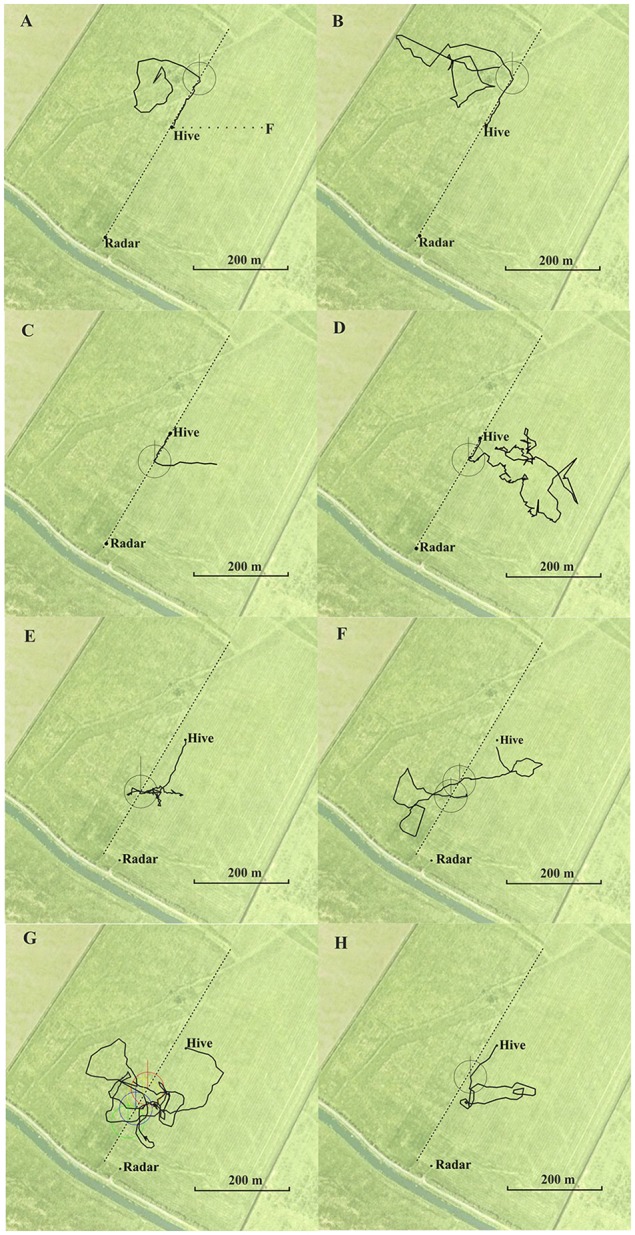
Eight representative examples of flight trajectories of homing bees released at different release sites **(A**,**B** north, **C–G** south of the hive). The hive was located at the boundary between two meadows in **(A–E)**, and 60 m east of the boundary in **(F,G)**. The feeding site (F) and the flight route between hive and feeder (dotted line) is indicated in **(A)**. The respective release site was the beginning of the trajectory. The trajectory ends at the hive. They first flew approximately 200 m to the west (vector flight; not recorded in **A)**. The circles around each crossing are used to determine the angle of the flight trajectory after crossing the boundary relative to the direction to the hive and relative to north. Statistics for the angular turn when crossing the border line for the condition in which the hive was located at the boundary **(A–D)**: angle to hive: mean vector μ: 2.8°, concertation: 77.3, circular variance 0.006, circular standard deviation 6.6°, standard error of mean 1.1°, 95% confidence interval −/+ for 0.6°, 4.9°; angle to north: mean vector μ: 25.5°, concertation: 145, circular variance 0.415, circular standard deviation 59.4°, standard error of mean 10.5°, 95% confidence interval −/+ for 4.84°, 46.1° (total *N* = 35 crossings in the south of the hive including multiple crossing of the same bee). Statistics of the angular turns when crossing the border line for the condition when the hive was located 60 m east of the boundary **(E–H)**: angle to hive: mean vector μ: 9.5°, concertation: 224.6, circular variance 0.002, circular standard deviation 3.83°, standard error of mean 0.66°, 95% confidence interval −/+ for 8.2°, 10.7°; angle to north: mean vector μ: 52.87°, concertation: 0.28, circular variance 0.864, circular standard deviation 114.4°, standard error of mean 50.79°, 95% confidence interval −/+ for 313.3°, 152.5° (*N* = 34 crossings in the south of the hive including multiple crossing of the same bee). North is upwards.

In Experiment 1, we asked whether a rather weak elongated ground structure (a field boundary separating two meadows mown at different times) guided homing bees when they were released at sites that brought them close to this landscape feature during their search flights. To answer this question, we trained the bees to a feeder 200 m east of the hive (Figure 1A) and released them at various sites either to the north or to the south of the hive. During training the bees did not follow an elongated ground structure but instead flew over rather even grassland. In one test (Figures 1A–D), the hive was located directly at this boundary, in the second test (Figures 1E–H), the hive was located 60 m east of it. The data analyzed here derive from an experiment in which we studied homing behavior from release sites at various directions and distances from the hive in a rather feature-poor environment (Menzel et al., 2005; see, https://doi.org/10.1073/pnas.0408550102). The question addressed here was not analyzed in the published data. We selected those homing flights that brought the bees closer than 60 m to the right or left of the boundary during their vector flight or search flight component.

Fifty-five from 77 bees from the hive close to the boundary performed the correct turn towards the hive when getting close to the boundary, 17 bees crossed it without turning, and five bees turned in the incorrect direction. From the 84 crossings made by the 55 bees, 74 were in the correct direction, 19 of them when the bees were already flying towards the hive. Exemplary trajectories are shown in Figures 1A–D. Bees from the hive 60 m east of the boundary flew significantly less frequently to the boundary (4 out of 34 came closer than ≤60 m) and returned home by trajectories that were more than 30 m further away from the boundary. These four bees followed the boundary in the correct direction towards the hive, and the 34 bees turned home equally well (exemplary flights in Figures 1E–H). We measured the angles when crossing the boundary as a measure of guidance towards the hive relative to the direction to the respective hive and relative to the north (see legend of Figure 1) and found that the angular distribution of bees from the hive at the boundary was significantly narrower than that of bees from the hive located further away from the boundary (angles relative to the respective hive: *P* < 0.001, angles to the north: *P* < 0.001; Wheeler Watson test). Thus, the boundary provided less guidance when the hive was not located directly at it, but it guided homing when the hive was located close to it.

Thus bees learned to use the boundary as a guiding structure when the hive was close to it. In contrast bees, did not use this elongated ground structure when they hive was not located at it although it would have been a useful landmark for bringing them close to their hive from the distance. The high proportion of correct turns towards the hive in the first case must have involved additional landmarks since this boundary was very similar along the whole stretch from SW to NE, and bees performed equally well when released north or south of the hive. These additional landmarks were obviously sufficient for successful homing in both experiments as bees from the hive at the boundary that did not get close to the boundary returned home equally well, and bees from the hive located 60 m east of the boundary also returned home equally well without following the boundary. These additional landmarks are likely to be other ground structures because no higher rising landmarks were in the vicinity and the horizon was flat within 2° visual angle. Figure 1 shows additional ground structures (e.g., different grasses growing in a dip in the ground stretching from SW to NE and local patches of vegetation differing between the area SW and NE of the hives) that possibly indicated to the bees if they were south or north of their respective hive. Since the hive could not be seen beyond a distance of 60 m, beacon orientation towards the hive can also be excluded.

### Experiment 2: Hive Training, Irrigation Channel

**Figure 2:**
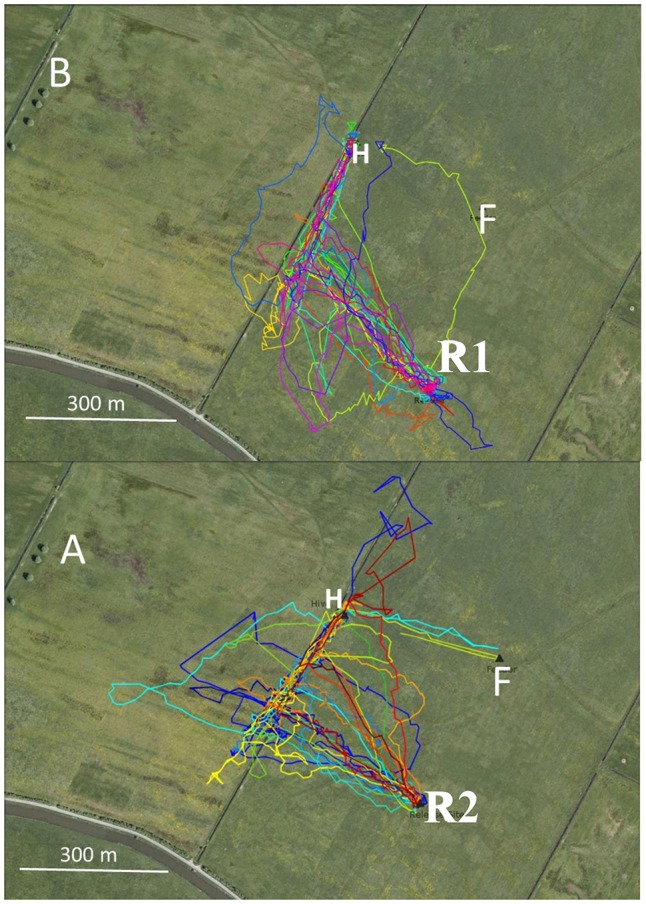
Flight trajectories of homing bees that were trained from two hives (H) to respective feeders (F) in two consecutive years (2011: **B**, 2012; **A**). The hives were located close to an irrigation channel. The respective feeders (were equidistant from the hives. The release site R1 in 2011 was located at the same distance from the irrigation channel as the distance between the hive and the feeder. Therefore, the bees released at that site reached the irrigation channel at the end of their vector flight. In 2012, the release site R2 was further away from the channel leading to a termination of the vector flight before reaching the channel. These test conditions allowed us to ask if the irrigation channel was a necessary guiding structure for homing. Statistics: 2011 first turn correct: 22/25; 2012: 12/22, *P* < 0.05; multiple turns until reaching the hive: 2011; 3/25, 2012: 6/22, *P* < 0.05.

In Experiment 2 two hives were located close to a narrow irrigation channel in the NW corner of the experimental field in two successive years (2011, 2012, Figure 2). The respective feeding stations were located SE of the hives at an equal distance. The corresponding release sites in the catch-and-release experiment were selected so that the homing bees either reached the irrigation channel during their initial vector flights (in 2011), or the vector flights ended at about 80 m southeast of the irrigation channel (in 2012). The data analyzed here derive from data addressing the question of whether neonicotinoids effect bees’ homing behavior (Fischer et al., 2014; see, https://doi.org/10.1371/journal.pone.0091364). The data used here correspond to the flight trajectories of the control groups that did not receive drugs and were not analyzed according to the question addressed here. In 2011, all 25 bees reached the channel and 22 turned towards the hive. Three bees crossed the channel and flew further to the NW aiming towards the hive on trajectories either close to (within 30 m) or further away (up to 100 m) from the channel to the W (Figure 2).Thus, each bee made only one turn that brought it to the hive. In 2012, none of 22 bees reached the channel during the initial vector flight (Figure 2). Thirteen out of the 22 bees turned to the hive either at the end of their vector flight or earlier without closely following the channel, six bees did not fly directly to the hive but did multiple turns, and three crossed the channel without turns. Four of six bees first turned away from the hive, then made a 180° turn within 100 m and then continued flying towards the hive. The precision of homing was significantly better in 2011 (see Figure 2 caption). Since the two hive locations did not differ with respect to the irrigation channel, we can assume that the bees from both hives learned the spatial relation of their respective hives to the channel equally well. The only difference between the two groups was the distance between the respective release site and the channel. If the bees reached the channel at the end of their vector flight, they used it as a “highway” to reach the hive. If their vector flight ended before, they aimed towards the hive but less precisely.

### Experiment 3: Hive and Feeder Training, Hedgerow

**Figure 3:**
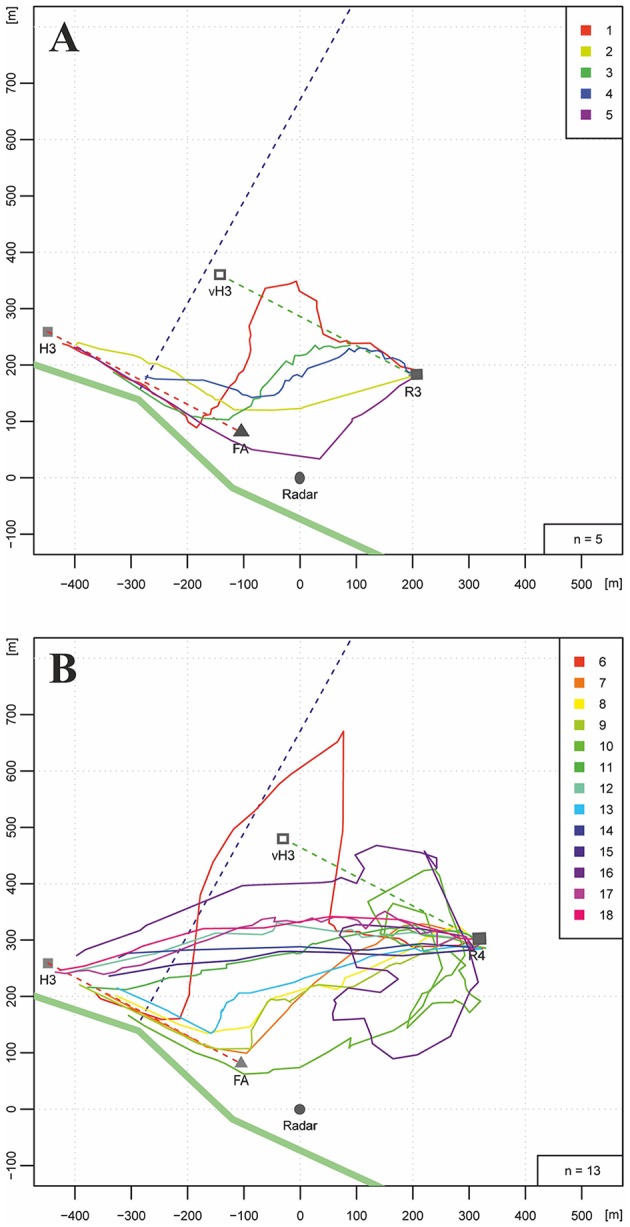
Homing flights of bees that had been trained along a hedgerow (bold green line stretching NW to SE) from hive H3 to the feeder (FA). The dotted violet line indicates an irrigation channel. During training the bees flew at a distance of 5–15 m along the hedgerow over 400 m (dotted red line). Two release sites were selected. R3 was 350 m NE of the hedgerow, R4 was 500 m NE of the hedgerow. A total of 23 bees were tested (color marks in the right upper corner of **(A**,**B)**. **(A)** Five bees released at R3 flew over different distances along the vector between feeder and hive (dotted green line connecting R3 with the location of the virtual hive, vH3). Two of them performed no vector flight. **(B)** Five of 13 bees released at R4 chose to fly first towards the hedgerow and then followed it. Seven bees flew along a shortcut directly from the release site to the hive. One flight trajectory (light red line) was rather irregular.

We trained bees along a hedgerow at the southern border of the same experimental field as in Experiments 1 and 2, and asked if the bees would use the hedgerow as a guiding structure when they were trained along it for 400 m and released at two different distances from the hedgerow (Figures 3A,B, R3: 350 m, R4: 500 m). The data presented here were obtained from a control experiment addressing the question of whether long-lasting anesthesia affects homing (Cheeseman et al., 2014b; see, https://doi.org/10.1073/pnas.1201734109). Data correspond to control, untreated bees, and were not analyzed with respect to the question addressed here. The distance of the release sites from the hedgerow was selected so that the hedgerow would appear at a visual angle <2° from R4 (500 m), whereas it would be visible from R3 (350 m, 2.5°). We hypothesized that the hedgerow would compete as a guiding structure with the information used during the training flights, namely the memory of the hedgerow as seen during the flights between hive and feeder (>50°). If the vector memory dominated, the bees should aim towards the virtual hive location (Figures 3A,B: vH3). Yet, if the memory about the hedgerow dominated, the bees should fly directly to this structure and then continue along it towards the hive. The salience of the hedgerow as a guiding structure should depend on the distance of the release site; a larger distance (R4, 500 m) would correspond to a lower salience.

Figures 3A,B show the results for R3 and R4, respectively. The hedgerow was indeed a salient landmark either leading to a strong reduction in the vector flight length (flight trajectories 1, 3, 4, in Figure 3A) or to a complete absence of the vector flight (flight trajectories 2 and 5 in Figure 3A, and flight trajectories 8, 9, 10, 13 in Figure 3B). Most interestingly, 7 out of 11 flights in Figure 3B followed direct connections between R4 and the hive. No such flights were found for the closer release site R3 indicating guidance by the hedgerow if it could be seen during the homing flights. Direct homing flights from the release site to the hive indicate a short-cut to the hive although it could not be seen from the release site. In these cases guidance is expected to derive from memory of ground structures as learned during orientation flights.

### Experiment 4: Hive and Feeder Training, Gravel Road

**Figure 4:**
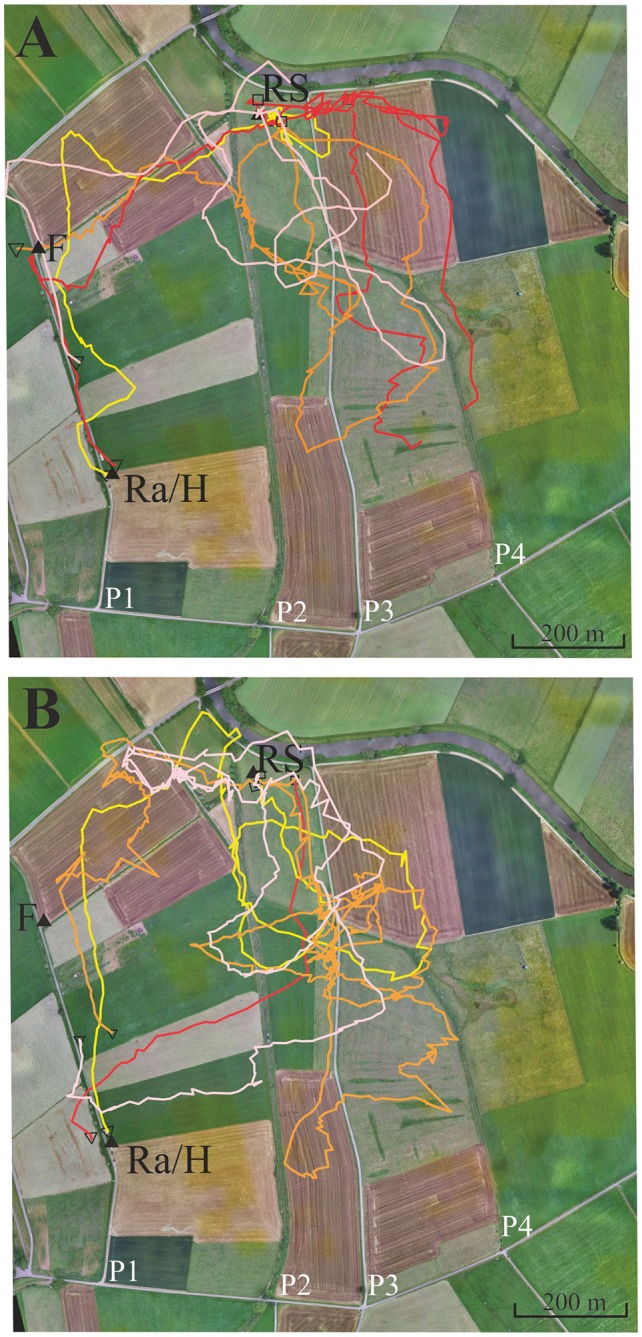
Eight representative examples of flight trajectories of bees that showed a tendency to follow path 3 (P3, see text). The bees were trained from the hive close to the radar cabin (Ra/H) along a gravel road (P1) to the feeder F. They were then transported to the release site RS. The flight trajectories of the eight bees are given in different colors in the two subfigures (see text). **(A)** Four representative examples of flight trajectories with lower tendency to follower P3. **(B)** Four representative examples with higher tendency to follow P3. Notice that the red line in **(A)** is interrupted because >3 fixes were missing at the end of the vector flight most like due to either low flight height or/and landing of the test animal.

**Figure 5:**
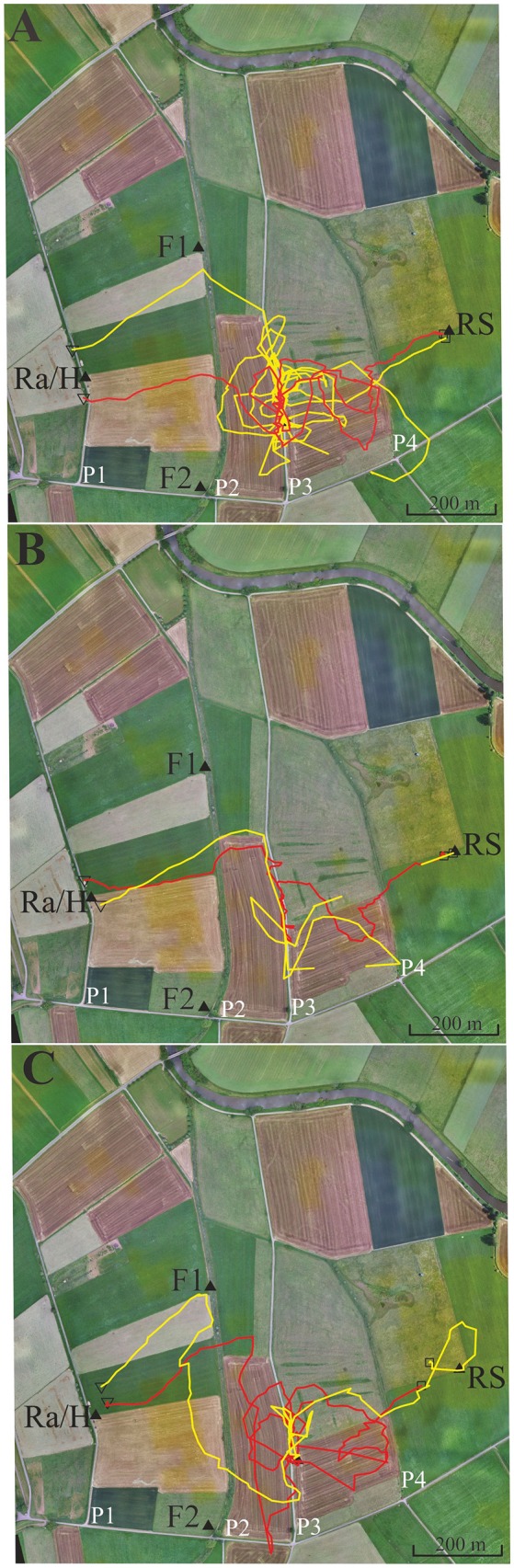
Six representative flight trajectories of bees that were distracted by path 3 (P3) on their homing flights from the release site RS to the hive. The bees were trained to feeder F1 (closed black triangle) and then released at RS 780 m east of the hive respectively marked with a closed black triangle. They passed P2 and P3, and in some cases even reached P4. **(A)** Two representative examples of high distraction by P3. **(B)** Two representative examples for low distraction by P3. **(C)** Two examples with different forms of distraction, one by a delayed effect leading to a distraction flight after passing P3. Trajectories were interrupted if no radar signals were recorded for more than 30 s. The black open square indicates the beginning of the flight, and the open black triangle the end of the flight as recorded by the harmonic radar.

**Figure 6:**
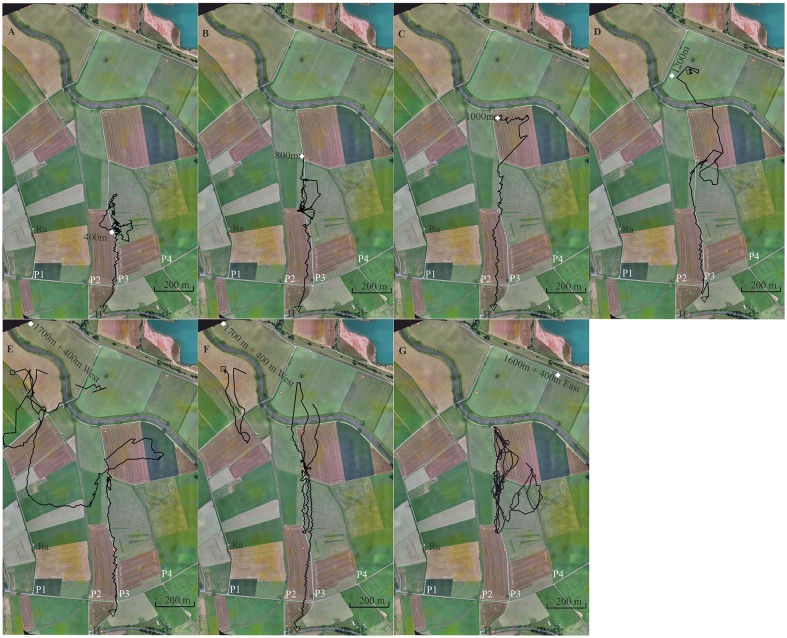
Seven representative flight trajectories of bees released multiple times along a linear ground structure, a gravel road (P3). The first three release sites were at 400 m **(A)**, 800 m **(B)** and 1,000 m **(C)** north of the hive (H), respectively. Further release sites were used as test sites at 1,200 m **(D)**, 1,700 + 400 m W **(E,F)** and 1,600 + 400 mE **(G)**. Each bee was released only once at these latter 3 release sites. The panels show in the background a section of the experimental area together with the respective release sites (white cross), and the S-N stretching elongated ground structures, paths 1, 2, 3 and 4 (P1, P2, P3, P4). P1 and P3 were gravel roads. P3 was the one along which the release sites were located. The open black square marks the beginning of the flight, and the black open triangle the end of the flight. Trajectories are interrupted if no radar signals were recorded for more than 30 s.

Experiment 4, as well as Experiments 5 and 6, were performed in area B, a landscape rich in local and far distant landmarks. Several linear structures ran parallel to each other in approximately S-N direction (Figure 4, paths P1, P2, P3, P4). Other elongated structures running approximately E-W divided up pastures. A narrow road also followed the approximate direction E-W (see background of Figures 4–6, see also Supplementary Figures S1, S2 showing the modeled bee eye views; Polster et al., 2018) together with the values of the Michelson contrast, lightness (including standard deviation), and the respective panoramas. The bees were trained to fly along path P1 to a feeder 400 m north of the hive. The data analyzed here come from an experiment aimed to examine the effect of the herbicide glyphosate on navigation (Balbuena et al., 2015; see, https://doi.org/10.1242/jeb.117291) and correspond to control, non-treated animals (*N* = 71). These data were not analyzed according to the question addressed here. The release site was chosen so that bees were likely to cross one of two (or both) S-N stretching paths (P2, P3). These paths ran approximately parallel to P1.

Four different homing strategies were observed: (1) bees first performed a vector flight leading to the virtual location of the hive and then reached the hive directly (37 out of 71, representative example trajectory in Figure 4B, red line); (2) they flew first to the feeder and then to the hive along the trained path P1 (12 out of 71, representative example in Figure 4A, yellow line); (3) bees first performed a vector flight leading to the virtual location of the hive and then flew to the hive *via* the feeder (14 out of 71, representative example in Figure 4A, red line); and (4) some bees (8 out of 71, Figure 4) followed path P3 running parallel to the trained P1 and then took a short cut to the hive. These eight trajectories belonged to 16 trajectories that got closer than 60 m to P3 and followed it to the south for a rather short stretch (>200 m). No bee followed P2 although 22 bees crossed it during the first 400 m, the length of the vector flight. P3 was rather similar in appearance to P1 as both were gravel roads, whereas P2 was overgrown with grass. It thus appears that only a small proportion of bees were attracted to a parallel path (P3) that mimicked important components of the linear ground structure they had learned during their foraging flights (P1), its S-N direction and appearance (gravel road). The road leading to the radar/hive is visible in the bee views at the feeder—and these views have a high Michelson contrast. No obvious panorama feature appeared to guide the bees during their four different homing strategies (Supplementary Figures S1, S2).

### Experiment 5: Feeder Training, Path, Irrigation Channel and Its Higher Rising Riverine Vegetation

In this experiment, we asked if bees learn an elongated landscape structure (path P2, see Figure 4) at the feeder and generalize it to a structure (path P3) that resembles components of P2 (see also Supplementary Figures S3, S4 showing the modeled bee eye views together with the values of the Michelson contrast, lightness (including standard deviation), and the respective panoramas). Two hives (A, B) were located in the radar cabin at P1. In the first test, bees from colony A were trained to feeder F1 in the NE at a 350 m distance, and bees of colony B were trained to a feeder F2 in the SE at a distance 340 m (Figure 5). In a second test, the training feeders were reversed. The two feeders differed with respect to the close surrounding landscape. F1 was located in the open landscape, and the bees could see path P2 and a parallel narrow irrigation channel with its higher rising riverine vegetation stretching S-N during their flights to the feeder both from the distance and during their final approach to it. Contrarily F2 was located under a group of trees and bushes. Bees trained to F2 were exposed to highly salient landmarks (trees, a EW stretching road, close-by bridge) and P2 was not seen as an elongated ground structure when the bees performed their final approach to the feeder located within the branches of the bushes. The data analyzed in this section come from experiments demonstrating the effect of thiacloprid, a neonicotinoid pesticide, on navigation and dance communication (Tison et al., 2016; see, https://doi.org/10.1021/acs.est.6b02658). They correspond to control, non-treated bees and have not been analyzed before with respect to the question addressed here, namely the guiding effect of elongated ground structures.

Foragers were collected when they left the respective feeders and were transported to the release site R5 at a 780 m distance to the hives. We analyzed the successful homing flights that reached P2 and P3. As expected the bees flew first along a vector that resembled in direction and distance the one they would have flown from the respective feeder to the hive (Figure 5). Most interestingly more than half of the foragers at F1 (43 out of 71) followed P3. Some bees did this several times (Figure 5). Foragers from F2, however, behaved differently. Only 5 out of 56 followed P3 (F1 vs. F2, Fischer exact test, *P* < 0.0001). The effect did not depend on which colony was trained to F1 or F2, but depended on the feeding site. Both groups of foragers came from colonies inside the same cabin and should have equal experience with the close P1. Thus, hive training to P1 cannot have caused the following of P3 since otherwise both groups of foragers should have shown the same behavior. Rather, this different behavior must result from the characteristics of their respective feeding sites. P2 and P3 run approximately parallel to each other but their appearance was rather different (P2: overgrown with grass, P3: gravel road). Furthermore, an irrigation channel that ran close and parallel to P2, was a feature lacking in P3. There was also no high-growing riverine vegetation along P3. We conclude that bees foraging at F1 but not those foraging at F2 associated the S-N stretching P2 and may have mistaken P3 with P2 during their homing flights. The elongated character of P2 was visible to the bees only when they reached feeder F1.

The bee views at the hive, feeders and release site are shown in Supplementary Figures S3, S4. The respective views and the panorama could not be calculated for feeder F2 since it is below trees and bushes, and the necessary data could not be collected with the helicopter. The ground structures at the hive, feeder F1 and release site were very different, and so any matching directly at the release site was most unlikely. A similar argument applies to the fine structure of the respective panoramas, and a coarse resemblance of the panoramas did not exist making it very unlikely that panorama matching played a guiding role.

### Experiment 6: Exploratory Learning, Gravel Road

Bees learn landmarks by exploration not only during their first orientation flights (Degen et al., 2016) but also during searching when homing from an unexpected release site. The learning effect is seen in repetitive releases from the same site (Menzel et al., 2005). In this experiment, we asked if bees released several times at an elongated ground structure (a gravel road, P3) learn to use this landmark for effective homing and prefer to fly along it rather than above unstructured grassland [Supplementary Data for Experiment 6 and also Supplementary Figures S5–S7 showing the modeled bee eye views together with the values of the Michelson contrast, lightness (including standard deviation), and the respective panoramas. The model calculations could not be run for release site R1700m + 400 W because it was outside the virtual world]. The test procedure emphasizes exploratory learning along a constant compass direction (southwards towards the hive) and asks whether a highly salient elongated ground structure (gravel road running in the same direction) is used by the bees as an additional guide. The hive was located at the S border of the experimental field (Figure 6).

After the bees became familiar with the landscape and performed regular foraging flights, individually marked bees were trained to a feeding site very close to the hive (<10 m). Single bees were released multiple times at increasing distances from the hive in a north direction along path P3. Each test bee was first brought to release site R400 m (400 m north of the hive), then to R800 m, then to R1000 m, and finally to R1200 m. R1200 was not located at the S-N stretching gravel road but behind a group of trees at a small road stretching from SW to NE. Two additional release sites were chosen to test whether the bees searched for P3 and followed it during homing. These release sites were located 400 m E or W of P3 (R1600 m + 400 m E and R1700 m + 400 W). The radar signals were rather unreliable at distances >1,000 m and therefore the initial flight paths after releases at R1200, R1600 and R1700 were usually not seen (Figures 6E–G).

A total of 126 flights were tracked (see Supplementary Data for Experiment 6). Figure 6 shows one representative trajectory for each release site (two for R1700 + 400 m W). Bees learned well to fly along P3. When they were released at R400 for the first time, they performed a few search flights that brought them back to the release site and then followed P3 to the S reaching the hive. When released at R800 for the first time, they usually headed S along P3 immediately reaching R400 and then continued towards the hive. Sometimes the bees searched up and down along P3 over short distances (see Figure 6B). Path following was not always directly above the path but over the even grassland to the E or W. Releases at R1200 brought the bees E or W of P3 at its N end, as seen in Figure 6D. Some bees corrected their flights and followed P3, others continued their vector flight parallel to P3 at a distance of >60 m over the whole distance. Those that flew W of P3 possibly mistook P2 with P3 although these two ground structures appeared rather differently (see Experiment 5). At least some of these bees came closer than 100 m to P3 and even crossed it but ignored it as a guiding structure. When they were displaced by 400 m to the E or W and released further in the N, they first flew according to the vector they had learned to the S. Since the radar’s limited range did not allow us to record the flights beyond 1,200 m away from the radar (Figures 6E–G), we detected only the S ends of these vector flights. The bees then searched but showed a clear tendency to fly towards the E when released W of P3 and to the W when released E of P3. The straight homing flights started in 6 of the 7 animals when they reached P3 coming either from the W or the E. One animal released at R1600 + 400 east hit P3, traveled it up and down several times but was not seen flying back to the hive. The tight connections of these 6 trajectories to the path suggests that the bees were guided by P3 although they did not fly accurately above it but rather approximately ≤60 m to the W.

The gravel road appeared as a salient feature throughout all flights starting along it. There were no corresponding ground structures at the release sites 1,200 m and 1,600 + 400 W m. Since the gravel road was not seen by the bees at the hive entrance one can rule out that this elongated ground structure was learned at the hive. No obvious feature of the panoramas appeared at the various release sites, supporting the interpretation that the guiding structures were only those at the ground seen by the bees on their homing flights.

Exploratory learning during multiple homing trips along a constant compass direction leads to both learning of this compass direction and learning of an equally directed salient ground structure (gravel road, P3). The release sites E and W of the gravel road indicate a dominance of the memory for the compass direction and only partial generalization to similar elongated ground structures aligned with the compass direction. The learned path is chosen over these displaced similar ground structures and displaced other landscape structures indicating object identification.

## Discussion

An extended landmark like the edge of a forest or a linear boundary on the ground can function as unique guiding structure for a flying animal, with characters different from a localized single beacon associated with the goal or the panorama learned as a picture at the goal. The object’s elongation keeps a stable relationship to a compass direction, a property that has been particularly well characterized for the honeybee in experiments by von Frisch and Lindauer (1954) and Dyer and Gould (1981) showing that bees read the compass direction from the edge direction of a forest under fully overcast sky. If the goal has been experienced as being close to the elongated landmark, an animal getting close to it at any other place will possibly be reminded about the learned object as a marker of the goal and may be guided towards the goal. Guidance to the correct direction can result either from the elongated structure’s polarity (e.g., in the case of a forest edge) or from additional landmarks that inform the animal whether it is upstream or downstream of the goal along this elongated landmark. This alignment effect has been well studied in humans (McNamara et al., 2003; Valiquette et al., 2007) and requires a functional hippocampus possibly via multiple co-activated place cells (referred to as “boundary vector cells,” Barry et al., 2006). Route directions taken by animated navigation systems is also strongly supported by line-like landmarks allowing robots to navigate more efficiently (Se et al., 2005; Furlan et al., 2007). Bats use stereotypical routes (“flyways”) along linear landmarks (Heithaus et al., 1975), and integrate such flyways in their navigation system (Geva-Sagiv et al., 2015). Both migratory birds and homing pigeons have also been observed to follow elongated ground structures like rivers (Geyr von Schweppenbug, 1933; Able, 1980). Guidance by such linear structures may become particularly obvious if the animal follows them to such an extent that even detours are made to keep tracking them, as has been observed in navigating pigeons in response to highways (Guilford et al., 2004; Lipp et al., 2004).

Navigation in bees, as in other animals, involves several sensory modalities, multiple sensory cues and different neural processes. Here, we focused on one environmental component, the presence of elongated structures in the environment, which could act as potentially salient guides for navigation. Guidance may be reflected by a “highway response” (following the elongated structure, Collett and Graham, 2015), by turns when hitting or crossing the structure leading the animals to a course along it, by attraction from the distance, by confusion with or generalization to similar landscape structures, and by the ways in which these structures are learned. We tried to overcome the problem of isolating the effect of one component over the other by comparing experimental data from different training regimes and different spatial relations between the particular elongated structures at the training as well as the test sites. We also varied the overall layout of the landscape under which these training sessions and tests were performed. One landscape was rather feature-less (Klein Lüben, Area A) while the other was rich in both local and distant features (Großseelheim, Area B). Other components of the experimental design were kept constant. The question asked was how these structures are learned as guideposts for their homing flights.

Experiment 1 proves that a linear landmark (a boundary between two pastures) was learned as a cue for the location of the hive. A strong innate tendency to follow such a linear structure was not seen when the hive was not located close to this ground structure. The correct turns towards the hive, both NE and SW of the boundary, emphasized the role and use of features of the boundary (e.g., its polarity although close to negligible) and additional local landmarks. In addition, orientation towards a beacon at the goal or by a view of the panorama was excluded based on the lack of spatial modulation given the resolution of the compound eyes of honeybees. This latter argument was questioned (Cheung et al., 2014) but emphasized by reference to the literature on spatial recognition of bees (Cheeseman et al., 2014a). Experiment 2 was set up to test whether a highly contrasting elongated landmark (an irrigation channel) leads to better homing performance if the bees reached it at the end of their vector flight as compared to a situation in which bees terminate their vector flight earlier and thus start their search flights before homing. No strong tendency was seen to follow the channel when bees terminated their vector flights before reaching the ground structure, but the bees that followed the channel more closely were more precise in homing.

Experiment 3 introduced a different elongated landmark, a hedgerow along which bees flew between the hive and a feeder. Such an arrangement allowed us to study the guiding role of vector flights in competition with this elongated structure. Two release sites were located at different distances from the hedgerow so that they had a higher or lower influence on homing flights. Bees starting at the release site at a shorter distance from the hedgerow performed short or no vector flights indicating that the hedgerow exerted a strong guiding influence on them. When the release site was at a larger distance, bees performed either flights towards the hedgerow or along shortcuts from the release site to the hive. A shortcut may be a form of spatial reference based on a memory that stores the spatial relations of two locations, the release site and the hive. Under these conditions, the hedgerow would be one of several local features characterizing the location of a further distant release site in its spatial relation to the hive. A beacon at the hive can be excluded since the distance was too large for the bees to see the hive situated low down on the ground.

Experiment 4 addressed the question of whether homing bees generalize a well-trained elongated ground structure (P1) to other elongated structures of similar appearance and quite similar compass orientation. Two of them (P2 and P3) were in the range of the release site and both had a similar compass direction (S-N) as P1. P3 matched P1 more closely in terms of its texture (both were grave roads) but it was further east while P2 differed in texture (path overgrown by grass). None of the bees followed P2, and only few followed P3 for short stretches; only one bee followed it extensively at some distance (red line in Figure 4B). Thus, even if such a highly contrasting elongated ground structure was well learned during foraging flights, its attractiveness as a guiding landmark was rather low. This suggests that similar but displaced structures are embedded in the landscape in such a way that they lose their attraction if their spatial relations to other landmarks (e.g., the panorama and local landmarks) do not fit. This argument is emphasized by the finding that the views at the respective sites (hive, feeder, release site) as modeled by a bee eye simulation and their corresponding panoramas did not uncover any specific guidance by the skyline or the distributions of local landmarks close by.

Experiment 5 proved that an elongated ground structure (P2) was learned as a landmark for the location of the feeder only if it was seen in the open as a bypassing linear landscape feature. We did not see any bee flying up and down P2 during training and during the foraging phase, suggesting that the compass direction of P2 was learned without scanning it. This landmark’s compass direction appeared to be the dominant parameter but not its object characteristic, because the scanning flight behavior was triggered by P3, which differed greatly in its appearance (gravel road as compared to a path overgrown by grass with an irrigation channel running parallel). The memory for the S-N stretching structure became effective only after the vector flight was terminated, i.e., during a subsequent search period. This indicates the dominance of the vector memory during the initial part of homing from an unexpected release site under these specific test conditions since there was no elongated ground structure matching the one at the feeder (P2) was visible at the release site. Furthermore, no guidance by the panorama was detectable, possibly because the skyline appeared not as a salient feature even in such a landscape rich in structure.

In Experiment 6, bees learned their homing flights by exploration. The three training sites were arranged along P3 aligned to a constant compass direction, and additional test sites (R1200 m, R1700 + 400 west or at R1600 + 400 east) were used for releasing the bees. Although there was a clear tendency to follow P3 as a highly contrasting linear ground structure and to correct displacements to the E or W, quite a few bees did not use it as a “highway” steering towards the S over rather unstructured grassland further away from it. The surrounding landscape contained multiple landmarks both at the horizon and on the ground providing the bees during their exploratory learning with additional spatial references. Guidance by P3 was, therefore, not the only reference for their homing behavior, and it is not surprising that they did not always follow it tightly. We interpret this result as a further indication that elongated landmarks are embedded in a spatial memory in which multiple landmarks and their spatial relationships are stored.

Taken together, the dominance of linear ground structures is not as high as expected because even rather salient extended directional cues (e.g., an irrigation channel or a gravel road) are selected for guidance only under certain conditions. The bee’s aerial view of the landscape embeds elongated ground structures into multiple landscape features indicating that memory of these linear structures is one of several components in navigation. Elongated structures interact and compete with other references. Object identification is an important part of this process. The objects are characterized not only by their appearance but also by their compass direction. Their salience is highest if both components are close to what had been learned. High similarity in appearance (e.g., texture) can compensate for (partial) misalignment in the compass, and vice versa. These conditions need to be integrated in a concept of navigation memory that avoids isolating particular natural landscape features and this requires experimental approaches under natural conditions with natural dimensions. Several attempts in this direction aim to synthesize and integrate the multiple components of navigation memory, e.g., the navigation toolbox concept (Wiener et al., 2011) or the concept of the integrated map (Jacobs and Menzel, 2014). They share the understanding that the essence of navigation can be captured only if the multiple interacting components are understood as parts of a unified cognitive entity. Such a unifying entity may still be differently expressed by individual bees depending on their life history, experience and age. These circumstances may cause the varying behavior of test bees as we saw it in practically all experiments presented here and in particular in Experiment 4 and 5. So far we lack the data necessary to trace the individual differences to former experience, but experiments are on the way currently to collect such data.

Multiple elongated landmarks may support a dedicated system for processing object geometry. A laboratory test situation would be a rectangular box in which an animal has to decide between locations placed in a particular location relative to the overall geometry of the environment. Since the seminal results by Cheng (1986) indicating a “geometric module” additional data from several animal species (ants, fishes, birds, primates and humans) support this conclusion (Cheng and Newcombe, 2005). It might be questionable whether concepts developed from observations under highly restrictive laboratory conditions and for minimal space may be transferable to the natural environment with relevant species specific dimensions. However, one may also argue that model studies under restrictive conditions may capture at least partly cognitive processes that compose multiple elongated landmarks under natural conditions into a global spatial reference frame that represents the intrinsic geometry of discrete object locations (Chan et al., 2012). Interestingly, the encoding of such environmental geometry is related to the hippocampus in laboratory rodents and in humans, as is spatial memory, structured as a “cognitive map” (O’Keefe and Nadel, 1978; Moser and Moser, 2008).

Very little is known about the neural substrate of navigation in insects (Homberg et al., 2011; Seelig and Jayaraman, 2015), and no neural recordings were performed yet that simulate close to natural conditions of navigation in insects close to natural conditions. The central complex is likely to be involved in compass related visual computation, and the mushroom body in multi-perceptual coding, object recognition and memory formation. These two high order neuropils are likely to code two major components of navigation, spatial reference and object identification. Recently, a parsimonious spiking neural network model was proposed that enabled simulated agents to follow learned routes (Müller et al., 2018). We extended the model proposed by Ardin et al. (2016). This model evaluated route following in flying insects (in particular the honeybee) in different worlds with changing object densities. The model included associative learning of sensory input with a behavioral context tempting to simulate foraging and homing. The spiking neural network model used sparse stimulus representation in the mushroom body and reward-based synaptic plasticity in its output synapses. Simulated bees were able to navigate correctly even when panoramic cues were missing, and performance degraded due to both: (a) too many features; and (b) too sparse features like in the flat world. Context related learning enabled the agents to successfully discriminate even partly overlapping routes. The structure of the visual environment was found to be crucial for the success rate. The model failed to reach the goal more often in visually rich environments due to the overlap of features represented by the Kenyon cells. Reducing the landmark density improved the agents route following. In very sparse environments, extended landmarks, such as roads or field edges, were found to help the agent to stay on its route. It thus appears that rather simple route following can be successfully implemented in rather straight forward assumptions about the processing of ground structure features in high order neural processes of the insect brain. Additional components might still be necessary for guidance and action selection while navigating along different memorized routes in complex natural environments.

Neural recording data are needed that examine how the central complex and the mushroom body interact, and whether one or the other of these two structures receive the output of the computations in the other structure. The sparse connections between central complex and mushroom body makes it likely that the central complex sends its compass information to the mushroom body because this information can be compressed in commands carrying low amount of information. The mushroom body in such a view (Menzel, 2014) would then treat this information together with the multiple perceptual inputs as one additional component characterizing objects and their spatial relations. In this interpretation the mammalian hippocampus and the mushroom body would have functional similarities possibly including a memory structure as a cognitive map.

## Data Availability

Experiment 1: https://doi.org/10.1073/pnas.0408550102

Experiment 2: https://doi.org/10.1371/journal.pone.0091364

Experiment 3: https://doi.org/10.1073/pnas.1201734109

Experiment 4: https://doi.org/10.1242/jeb.117291

Experiment 5: https://doi.org/10.1021/acs.est.6b02658

Experiment 6: see Supplementary Information.

## Ethics Statement

The animals used (Honeybees, Apis mellifera) were not exposed to any uncomfortable or unnatural conditions.

## Author Contributions

RM conceived the goal of the study. JC, JF-N, LT, MB, RM, UG designed and performed the experiments. TL and JPo developed the bee eye view model and made calculations. JPe, TL, XC, UG helped to analyze the data.

## Conflict of Interest Statement

The authors declare that the research was conducted in the absence of any commercial or financial relationships that could be construed as a potential conflict of interest.

## Footnotes

^1^Wavemetrics.com

^2^https://www.rockware.com/product/oriana/

## Supplementary Material

FIGURE S1 Supplemental Material A for Figure 4 (panorama).

FIGURE S2 Supplemental Material B for Figure 4 (ground structures).

FIGURE S3 Supplemental Material A for Figure 5 (panorama).

FIGURE S4 Supplemental Material A for Figure 5 (ground structures).

FIGURE S5 Supplemental Material A for Figure 6 (panorama).

FIGURE S6 Supplemental Material A for Figure 6 (panorama).

FIGURE S7 Supplemental Material A for Figure 6 (ground structures).
