# Supplementary figures and images for: Guidance of Navigating Honeybees by Learned Elongated Ground Structures
Source: Front Behav Neurosci. 2019 Jan 15;12:322. doi: 10.3389/fnbeh.2018.00322 (PMC6341004; doi:10.3389/fnbeh.2018.00322)

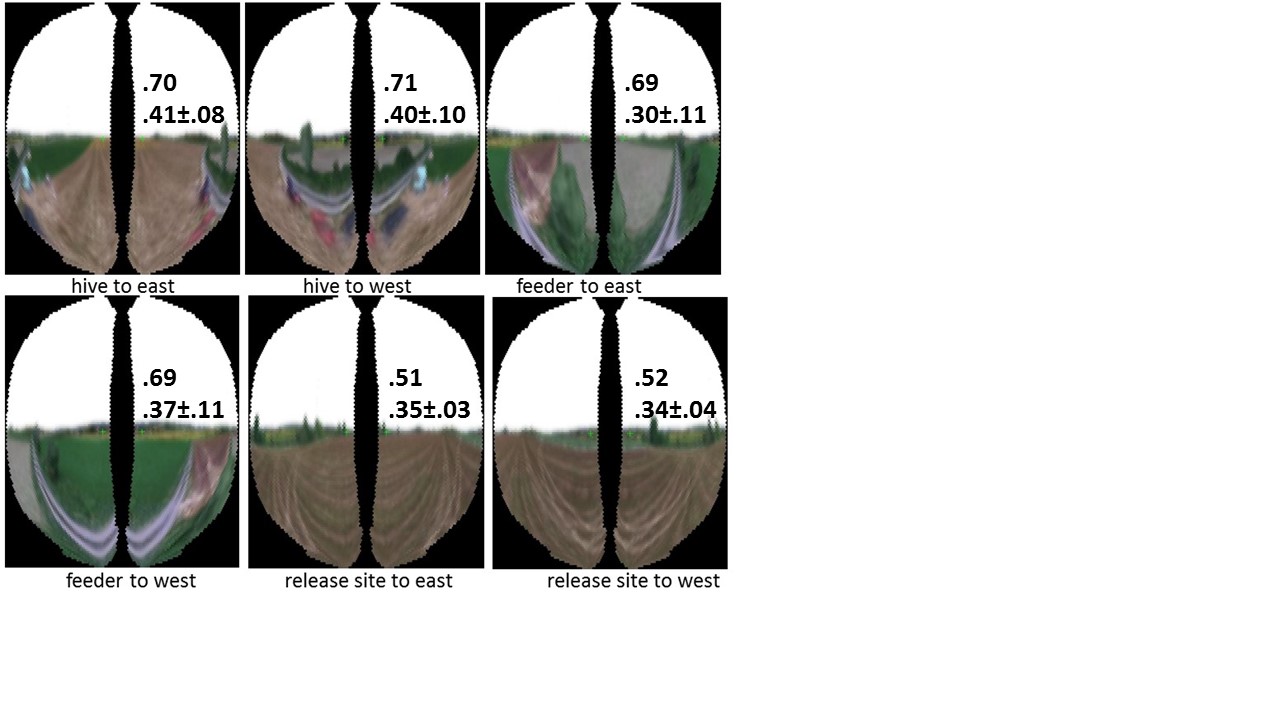

Supplement: FIGURE S1 — Supplemental Material A for Figure 4 (panorama). [file Image_1.JPEG]

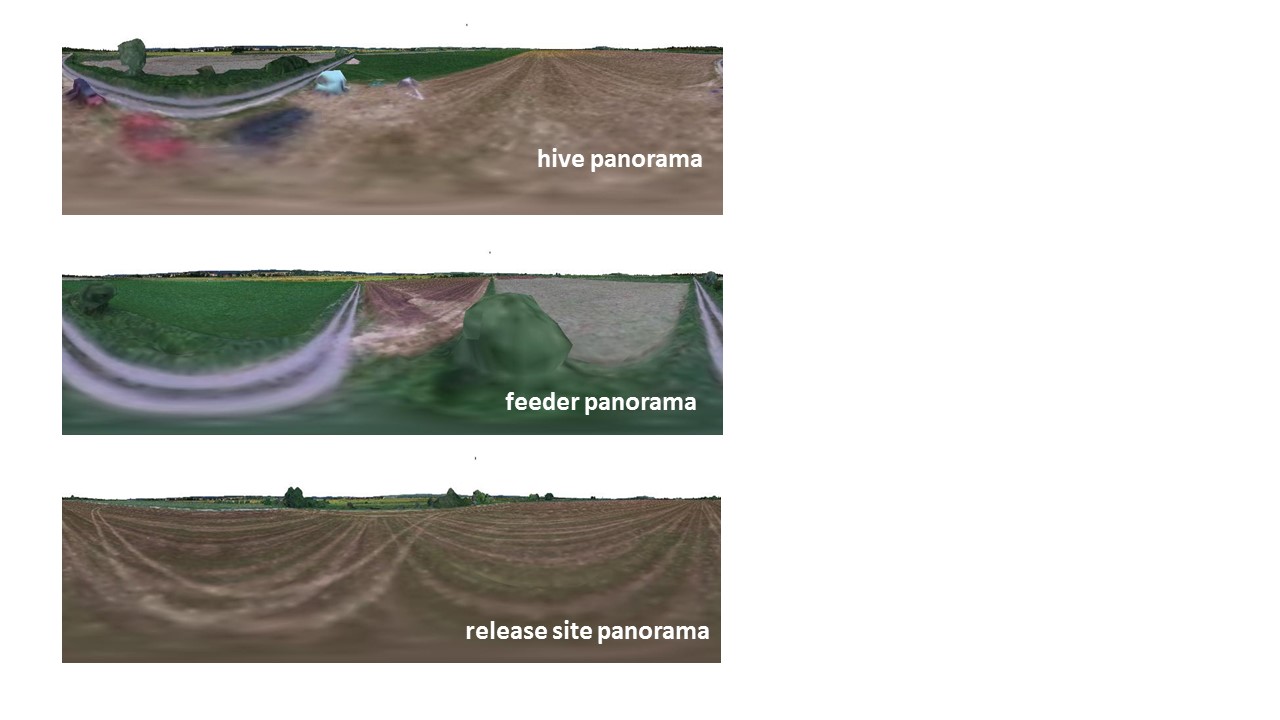

Supplement: FIGURE S2 — Supplemental Material B for Figure 4 (ground structures). [file Image_2.JPEG]

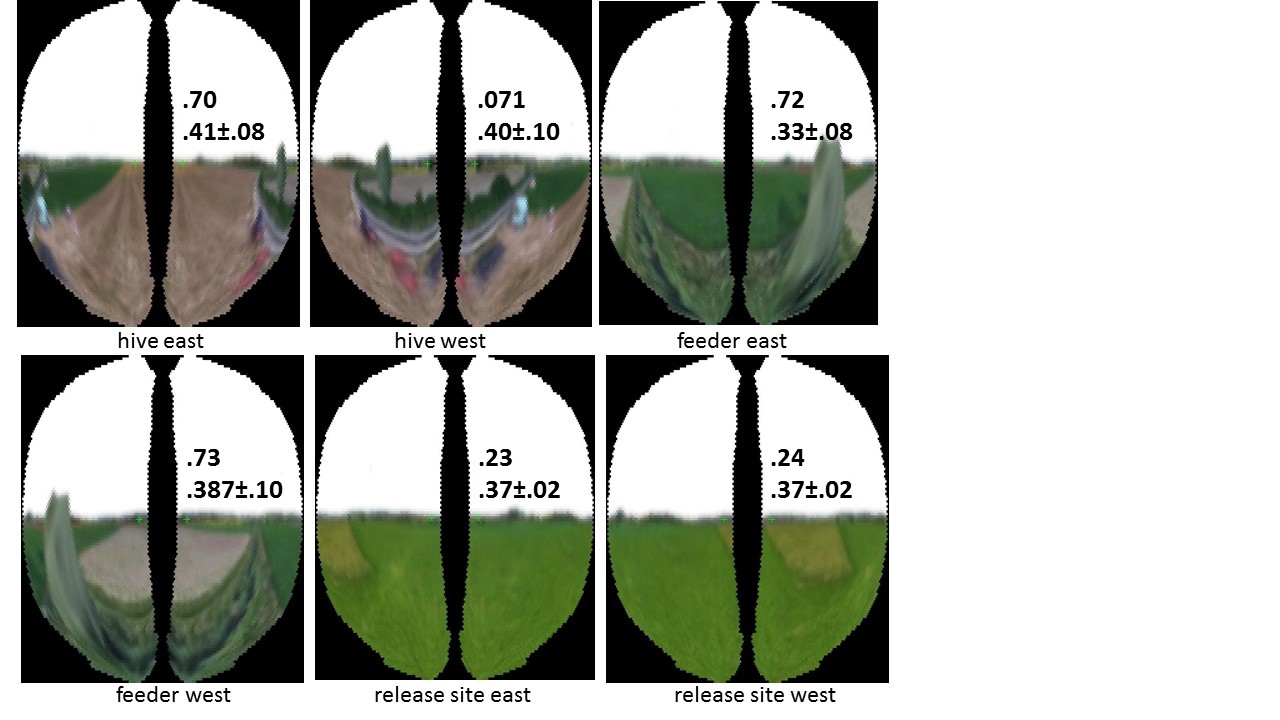

Supplement: FIGURE S3 — Supplemental Material A for Figure 5 (panorama). [file Image_3.JPEG]

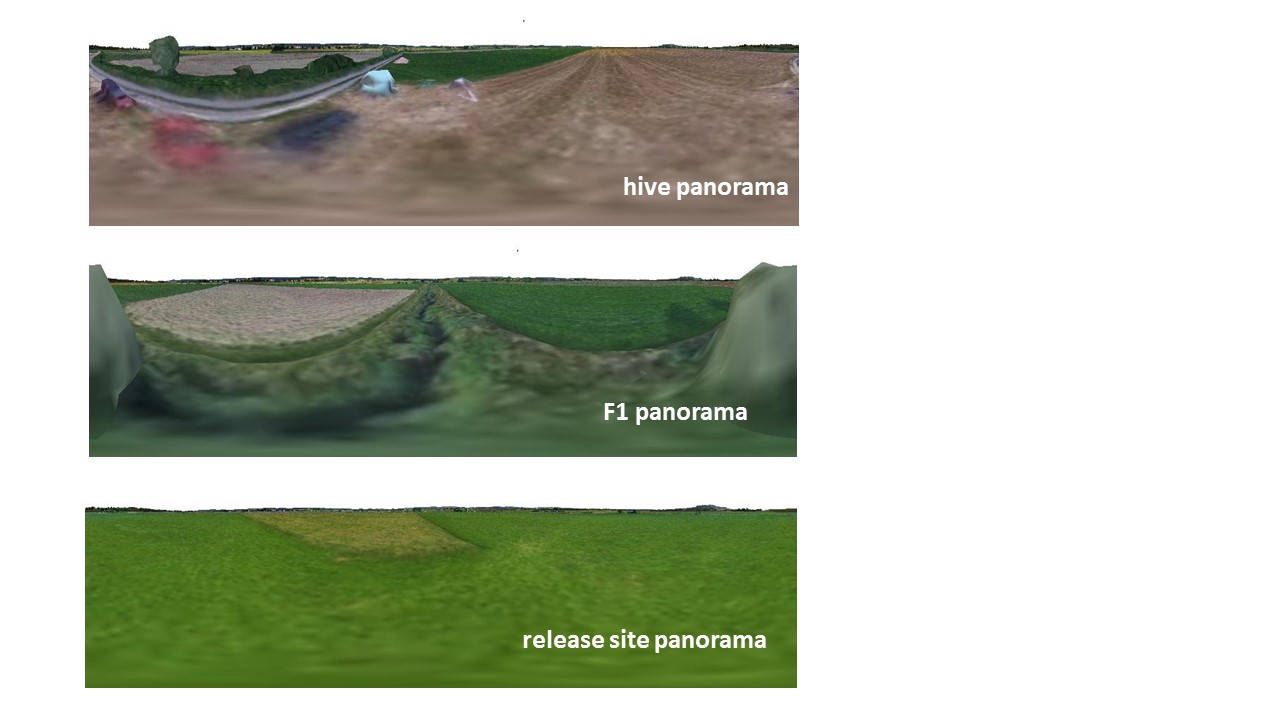

Supplement: FIGURE S4 — Supplemental Material A for Figure 5 (ground structures). [file Image_4.JPEG]

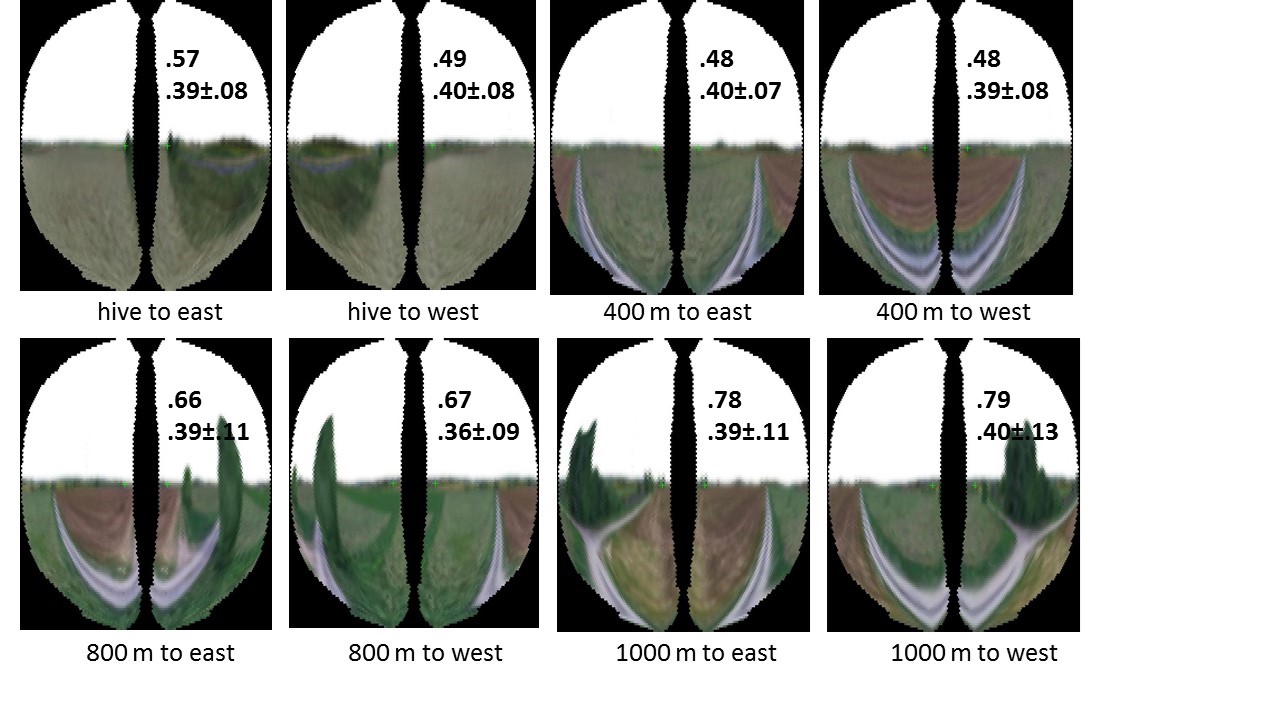

Supplement: FIGURE S5 — Supplemental Material A for Figure 6 (panorama). [file Image_5.JPEG]

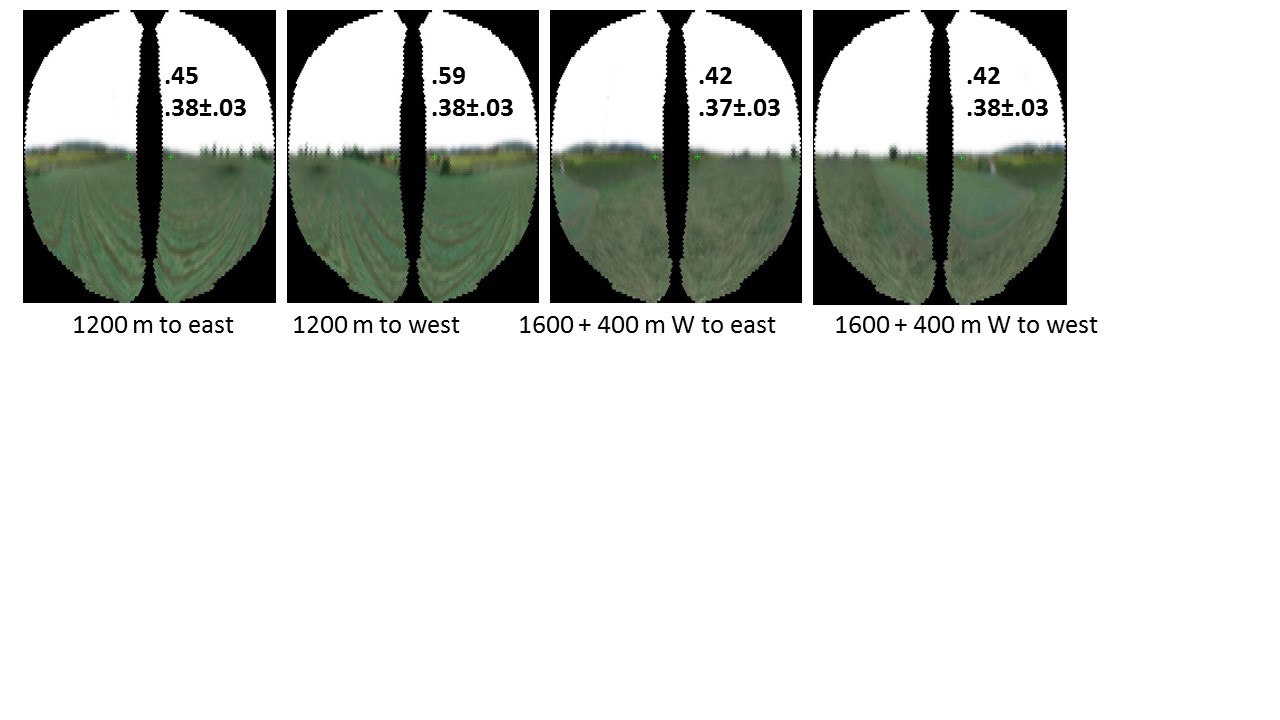

Supplement: FIGURE S6 — Supplemental Material A for Figure 6 (panorama). [file Image_6.JPEG]

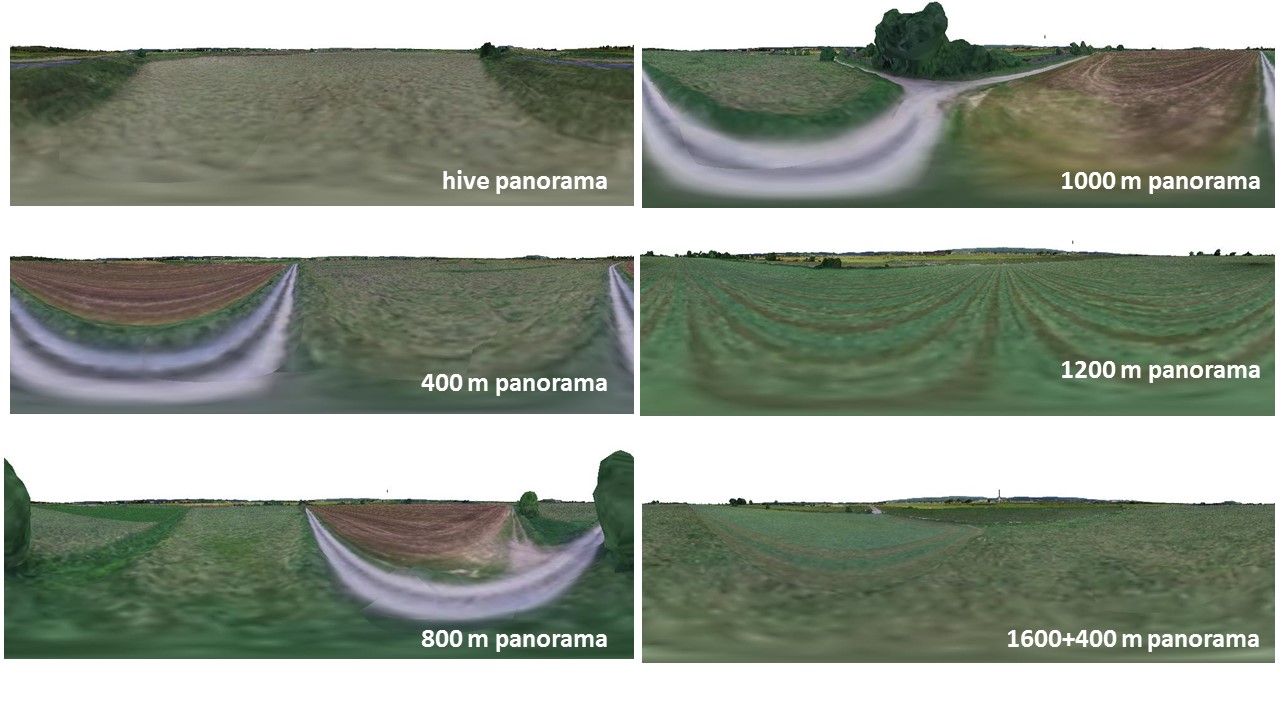

Supplement: FIGURE S7 — Supplemental Material A for Figure 6 (ground structures). [file Image_7.JPEG]
