# Supplementary material for: Guidance of Navigating Honeybees by Learned Elongated Ground Structures
Source: Front Behav Neurosci. 2019 Jan 15;12:322. doi: 10.3389/fnbeh.2018.00322 (PMC6341004; doi:10.3389/fnbeh.2018.00322)

13082013_pink4_400m(2)

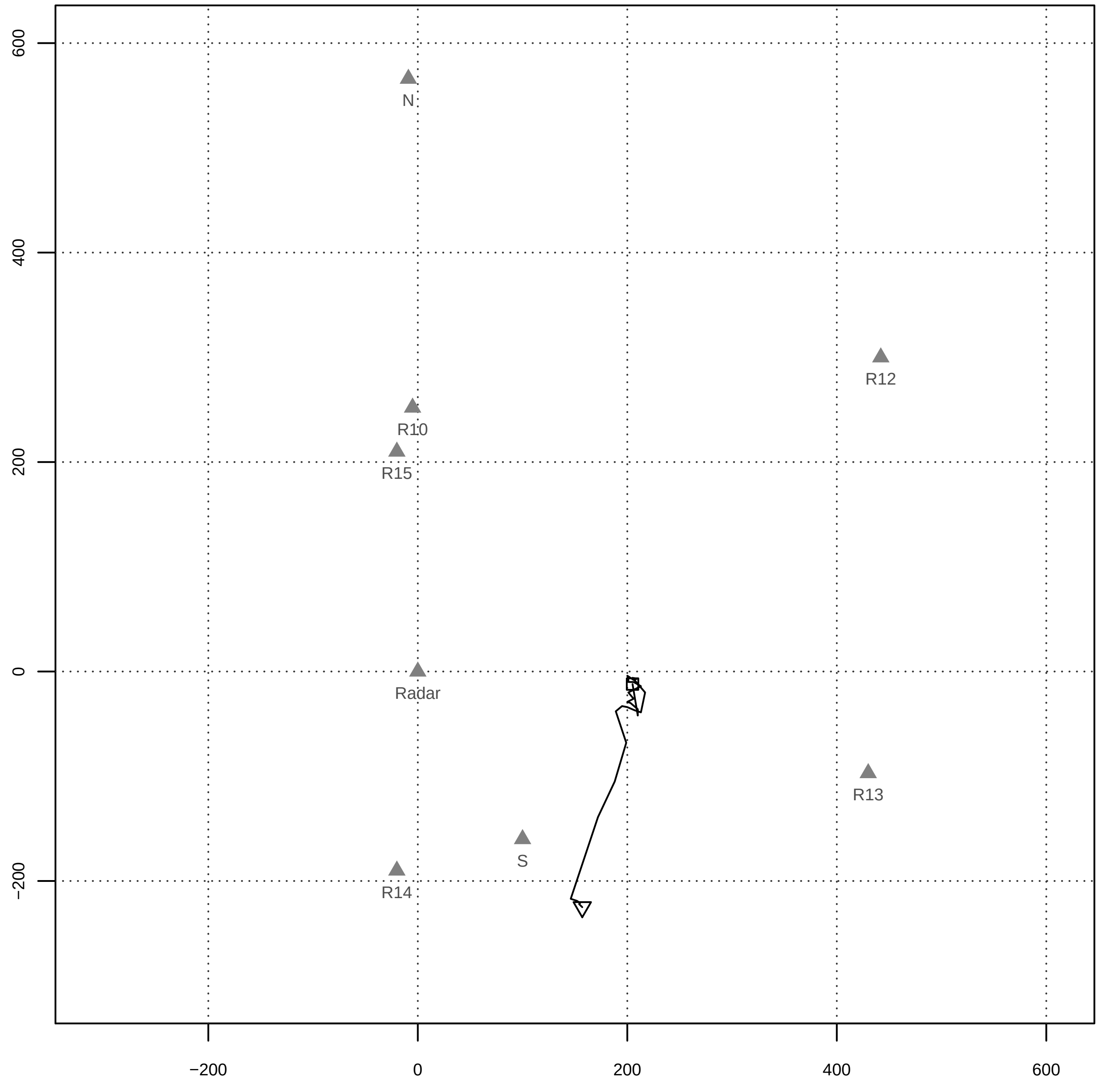

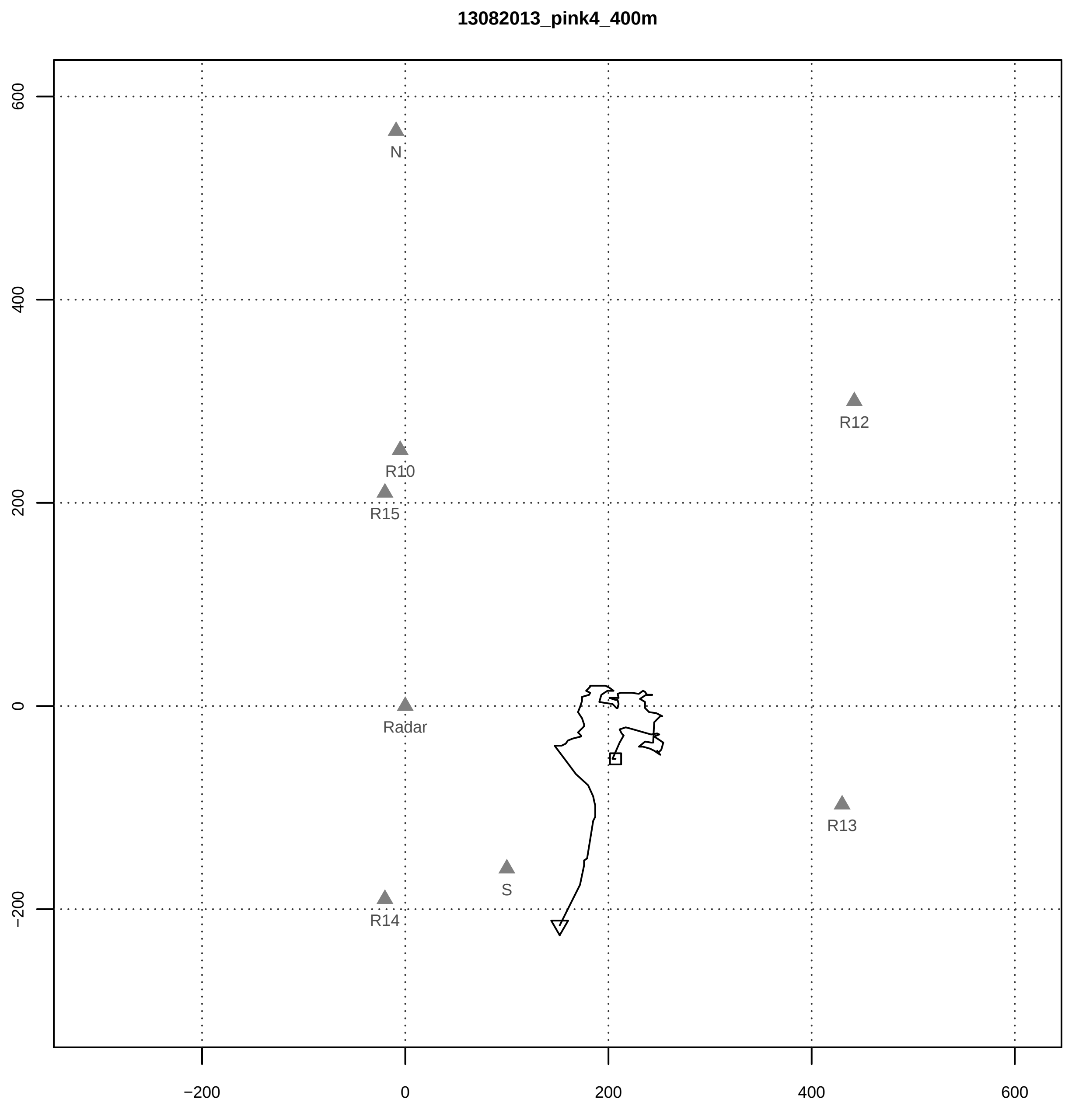

13082013_pink4_400m
600
400
200
0
−200
−200
0
200
400
600
N
R12
R10
R15
Radar
R13
S
R14

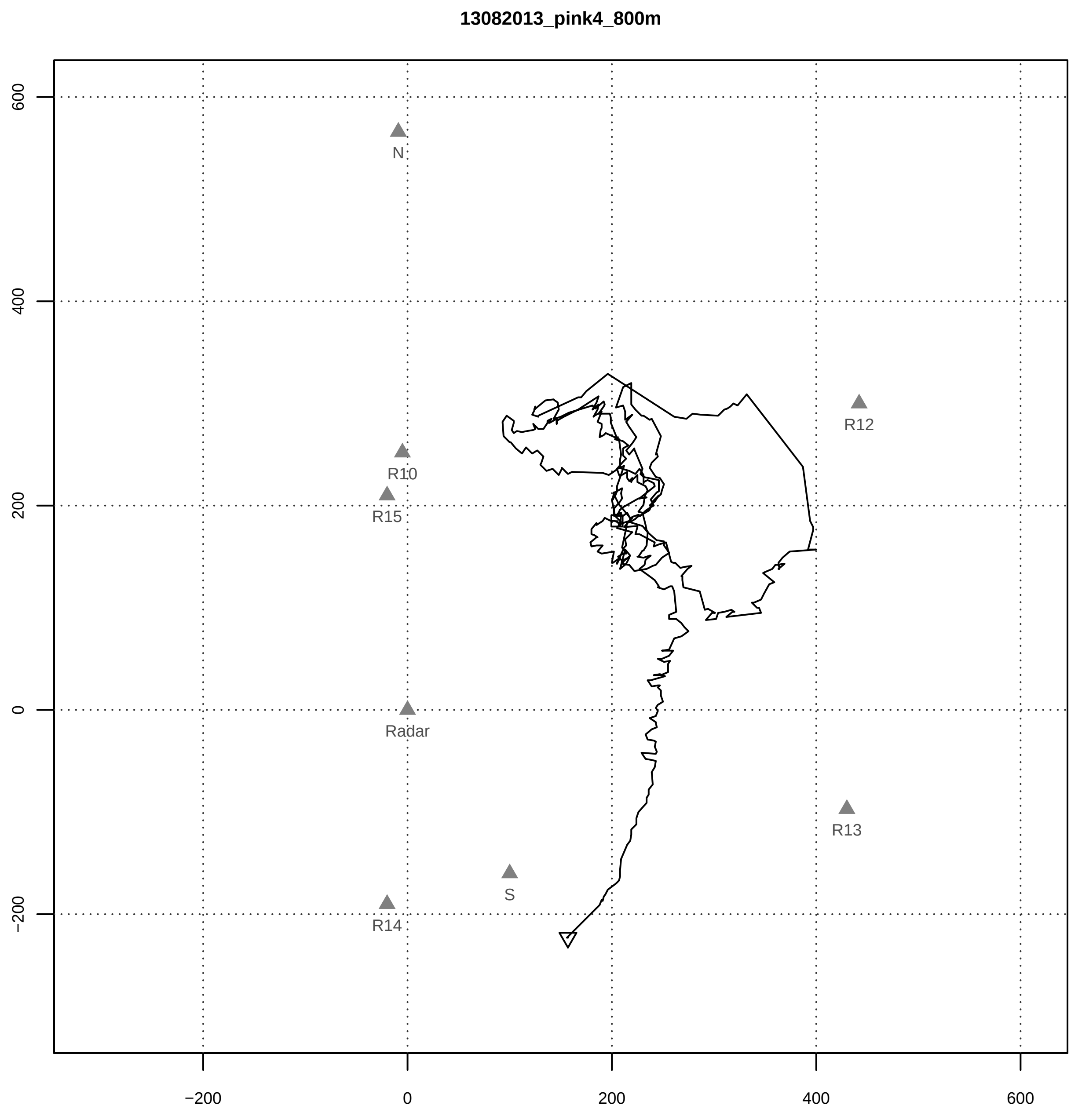

13082013_pink4_800m
N
R10
R15
R12
Radar
R13
S
R14
600
400
200
0
−200
−200
0
200
400
600

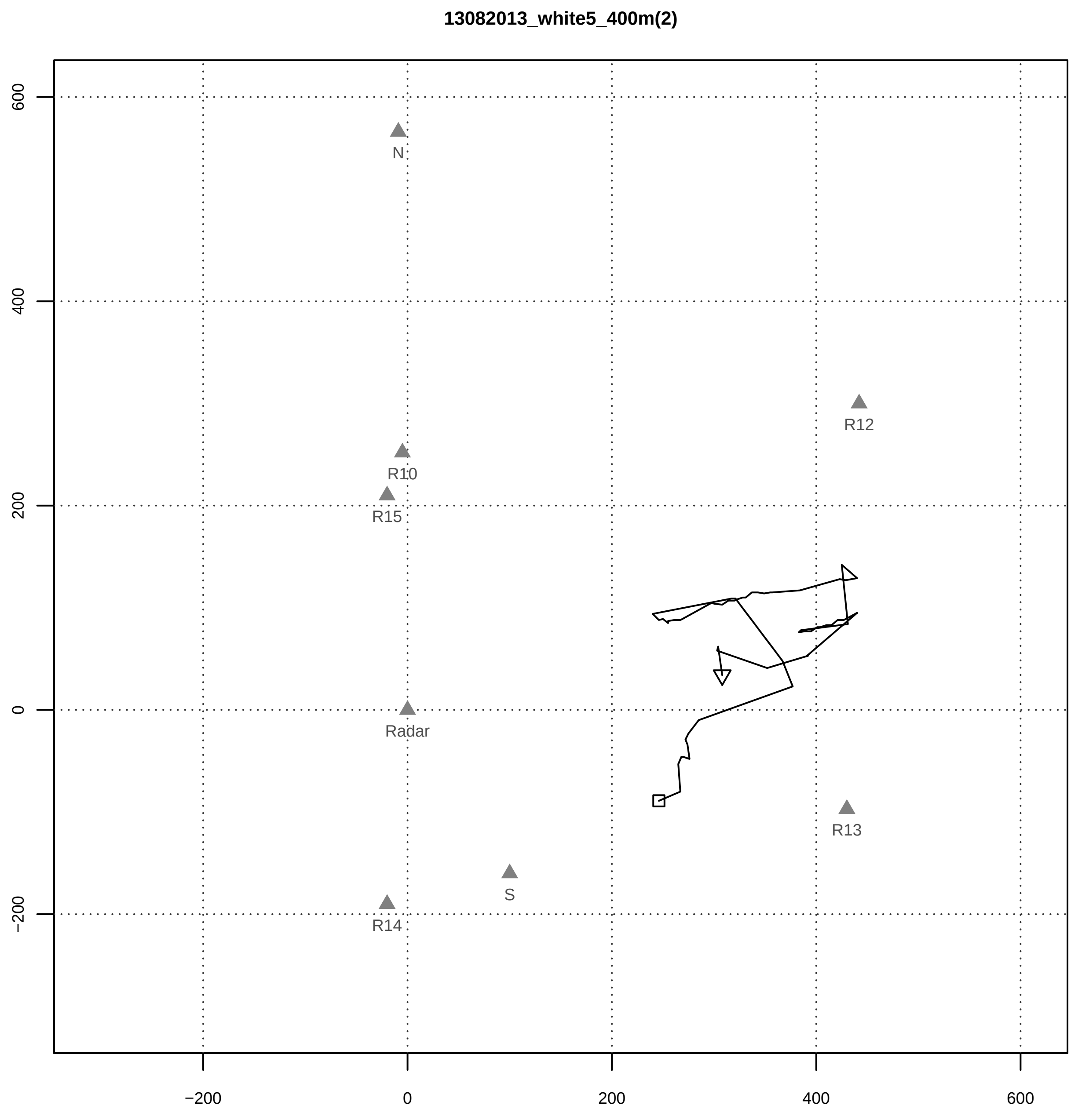
13082013_white5_400m(2)
N
R12
R10
R15
Radar
R13
S
R14
−200
0
200
400
600

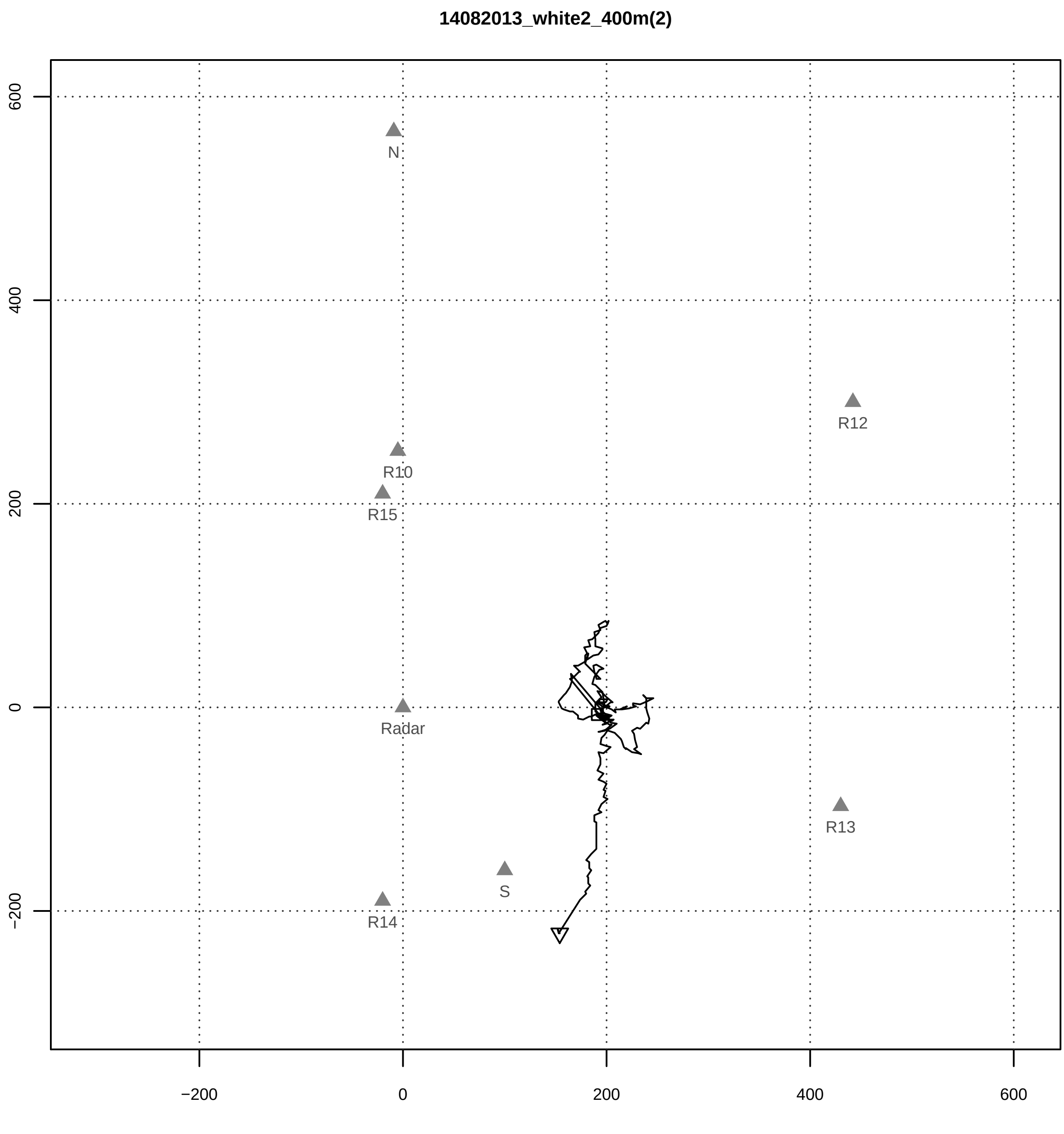

14082013_white2_400m(2)
N
R12
R10
R15
Radar
R13
S
R14
600
400
200
0
−200
−200
0
200
400
600

14082013_white7_400m

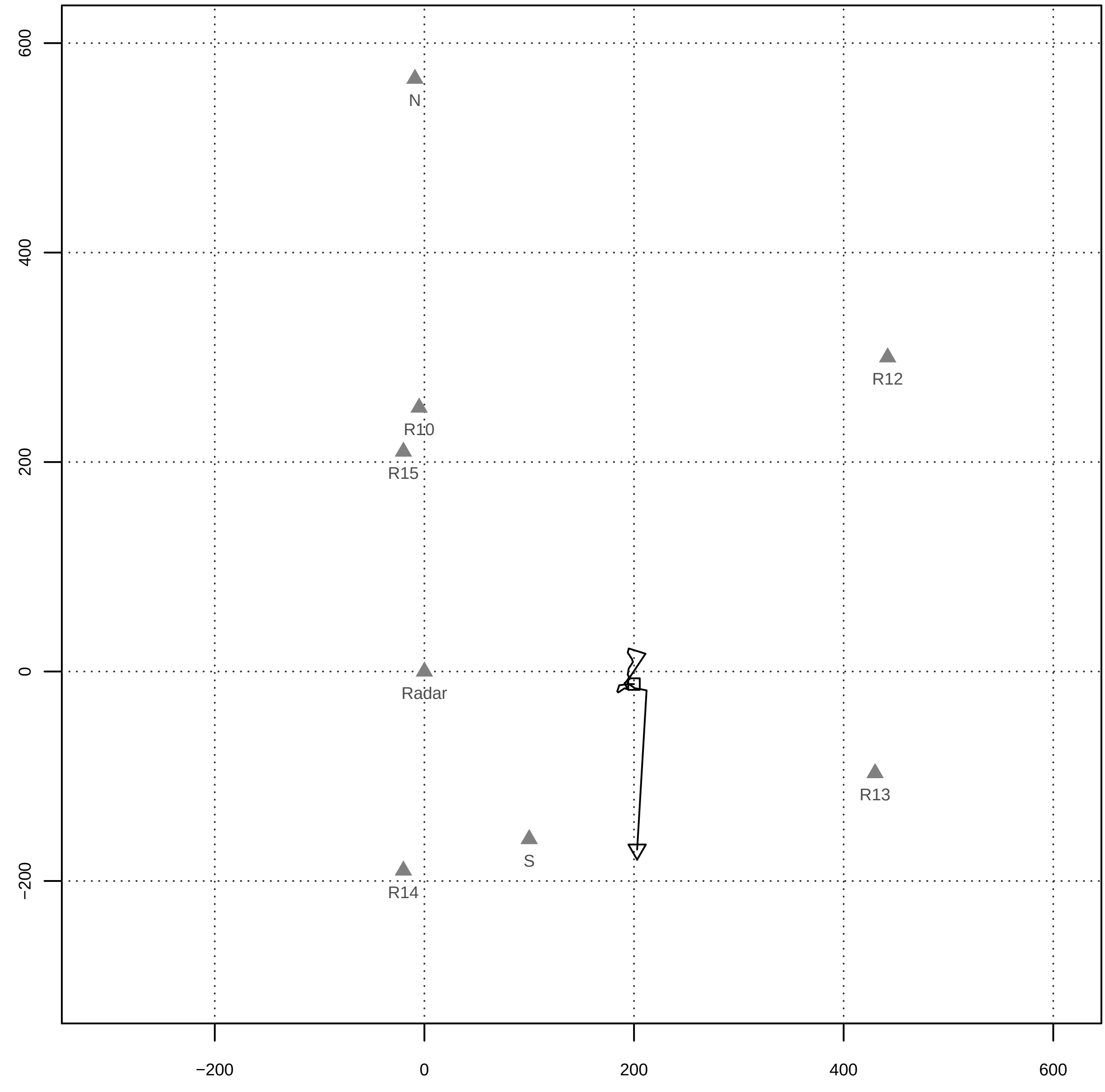

14082013_yellow2_400m

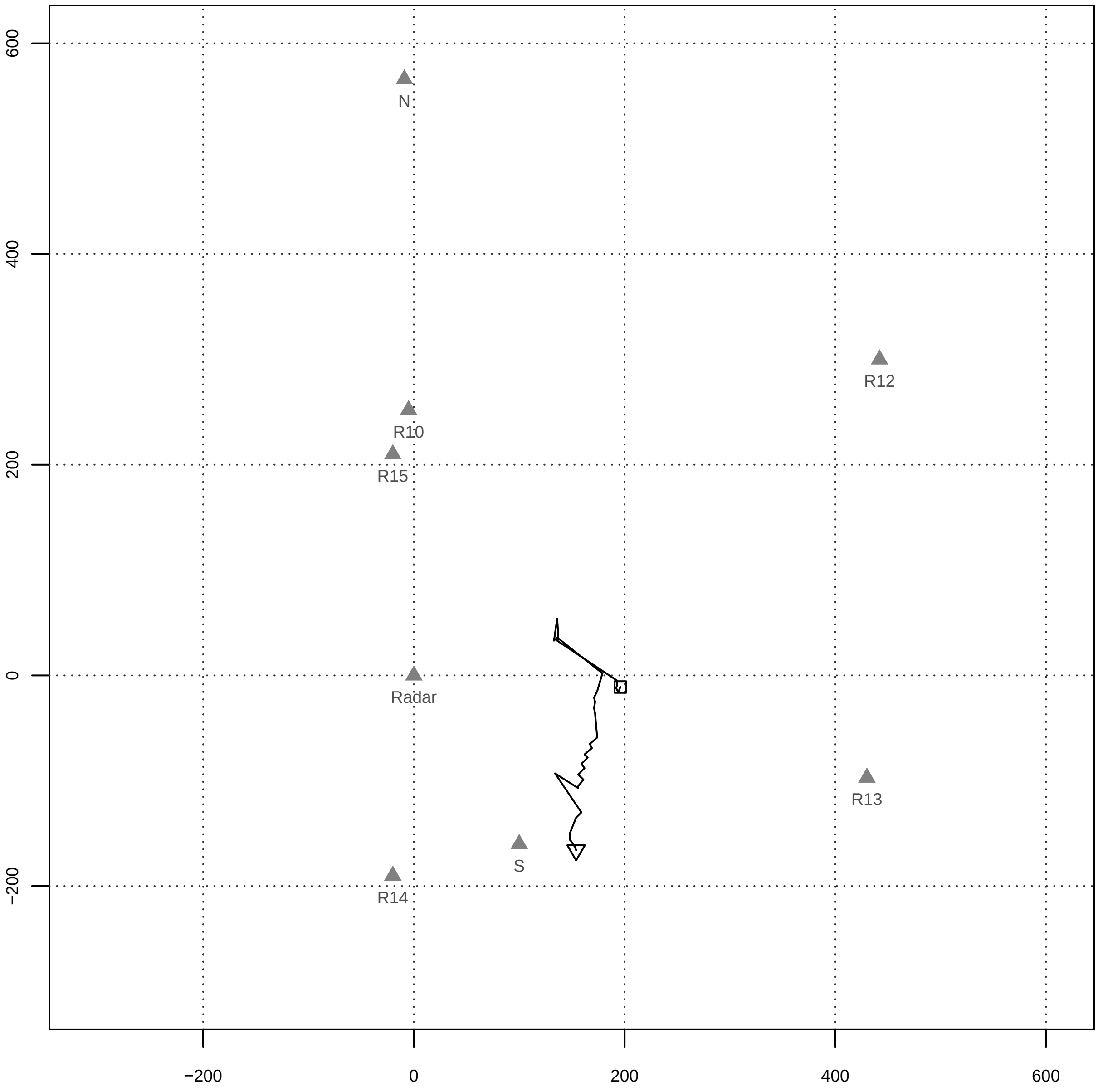

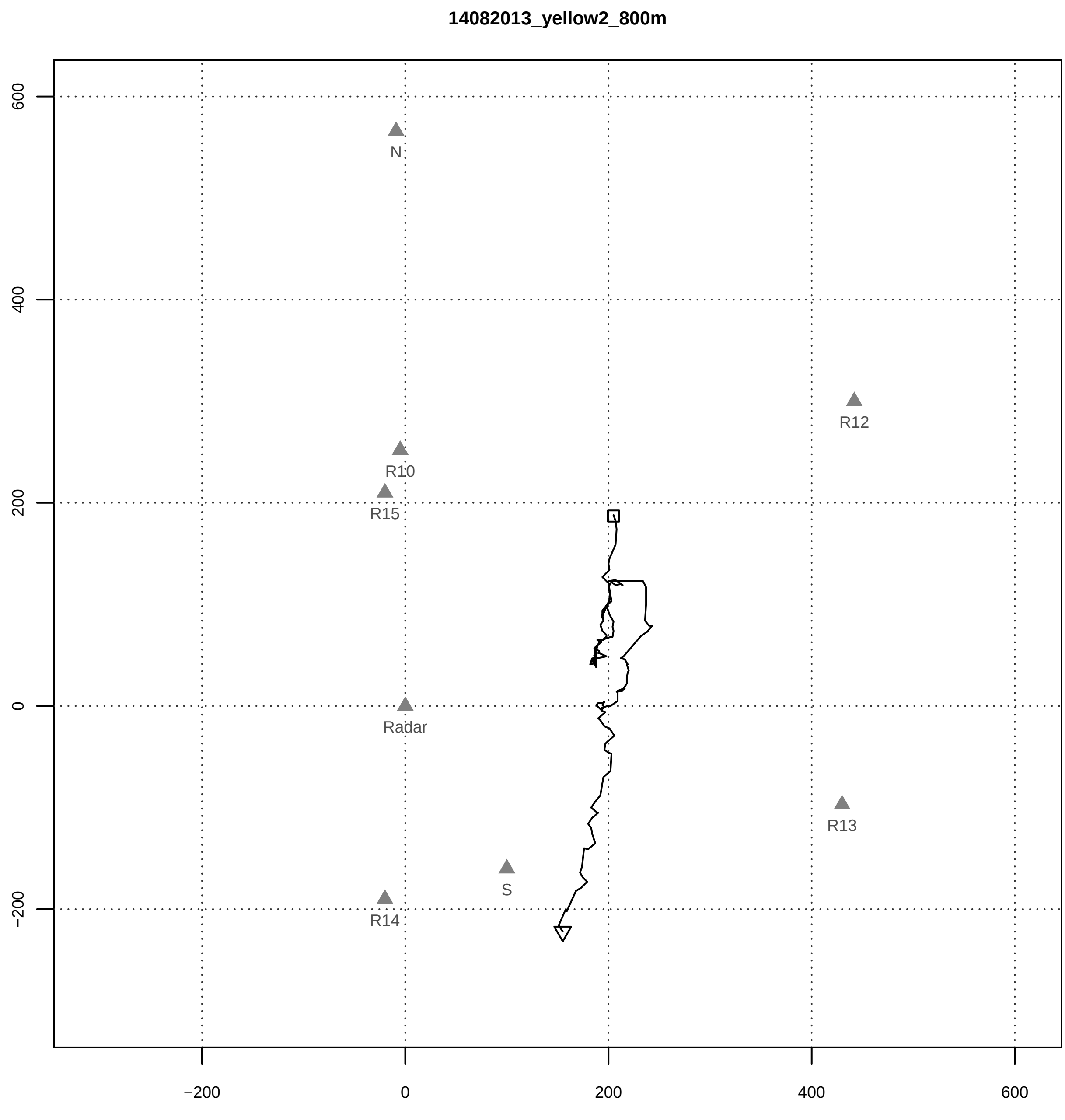
14082013_yellow2_800m
N
R12
R10
R15
Radar
R13
S
R14
−200
0
200
400
600
−200
0
200
400
600

14082013_yellow10_400m(2)

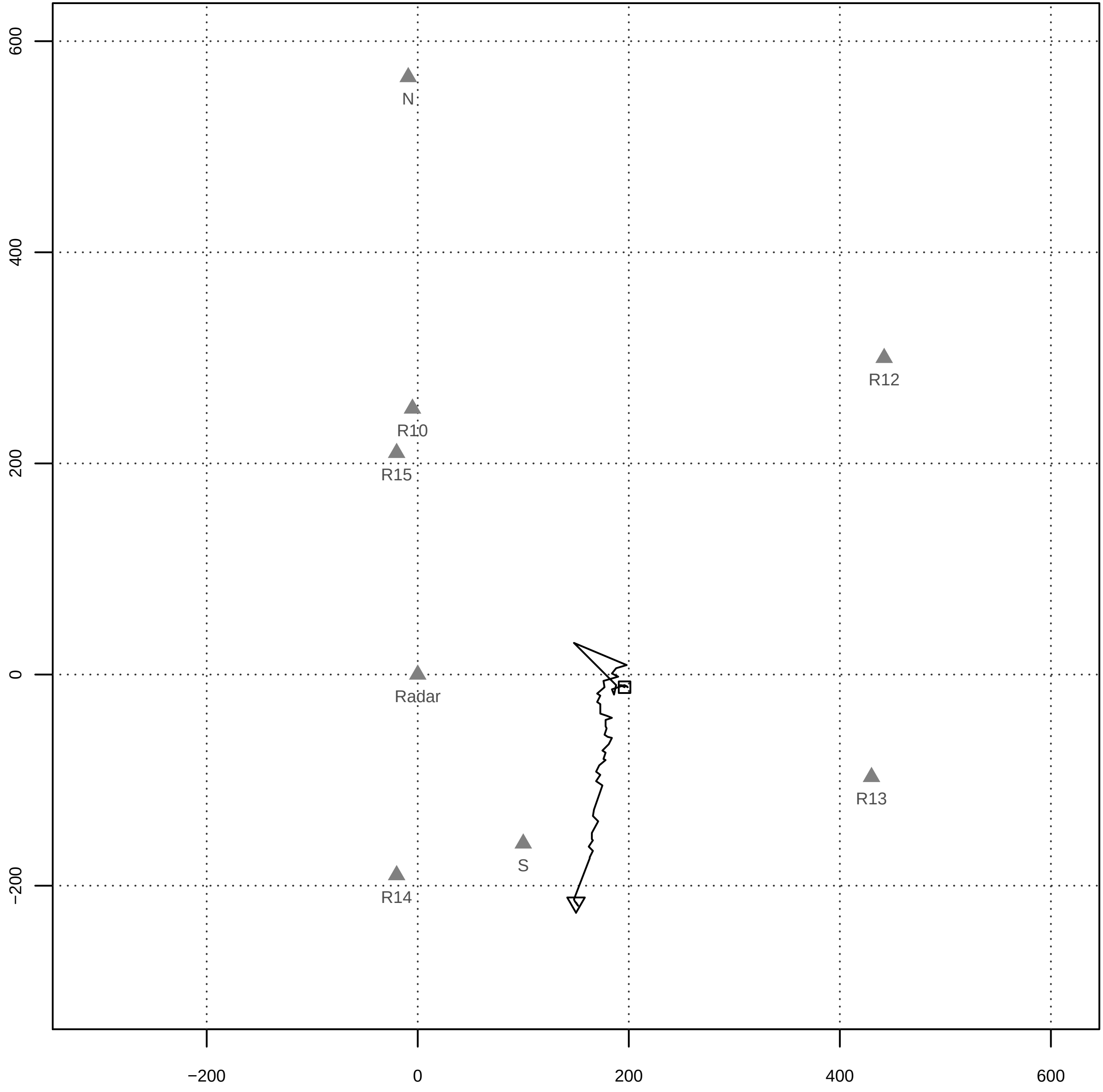

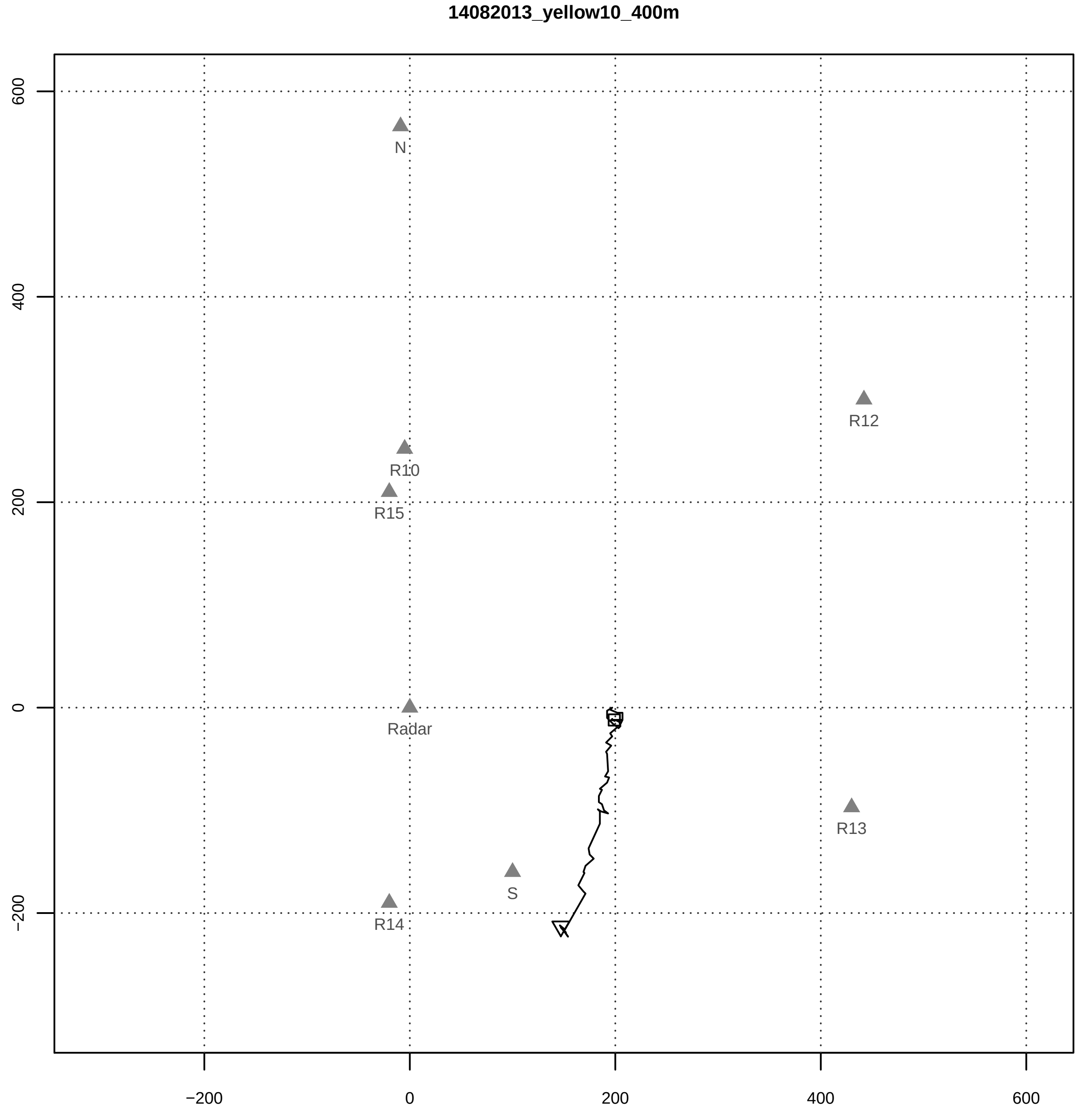

14082013_yellow10_400m
N
R12
R10
R15
Radar
R13
S
R14
−200
0
200
400
600

14082013_yellow11_400m

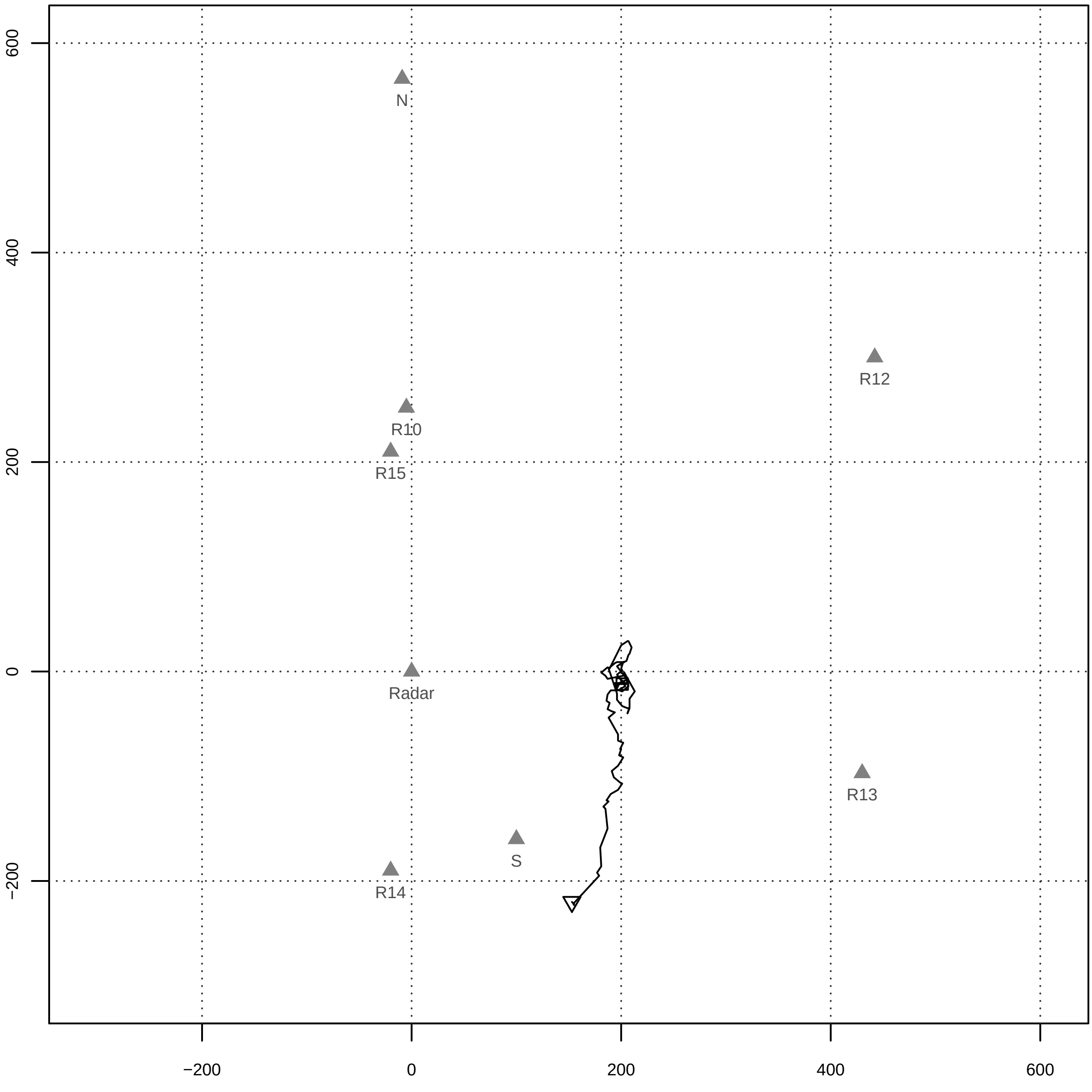

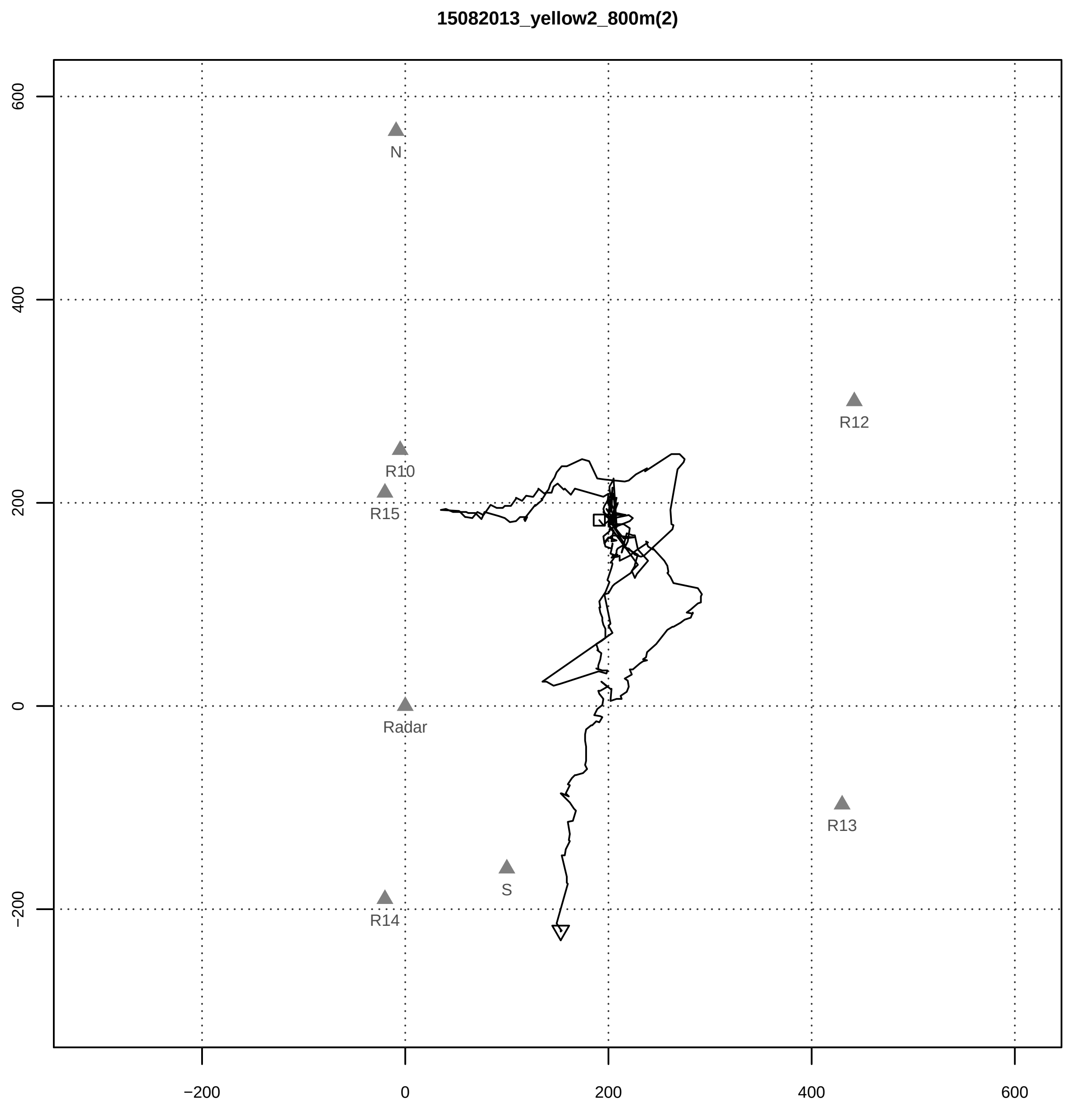
15082013_yellow2_800m(2)
N
R10
R15
R12
Radar
R13
S
R14
600
400
200
0
−200
−200
0
200
400
600

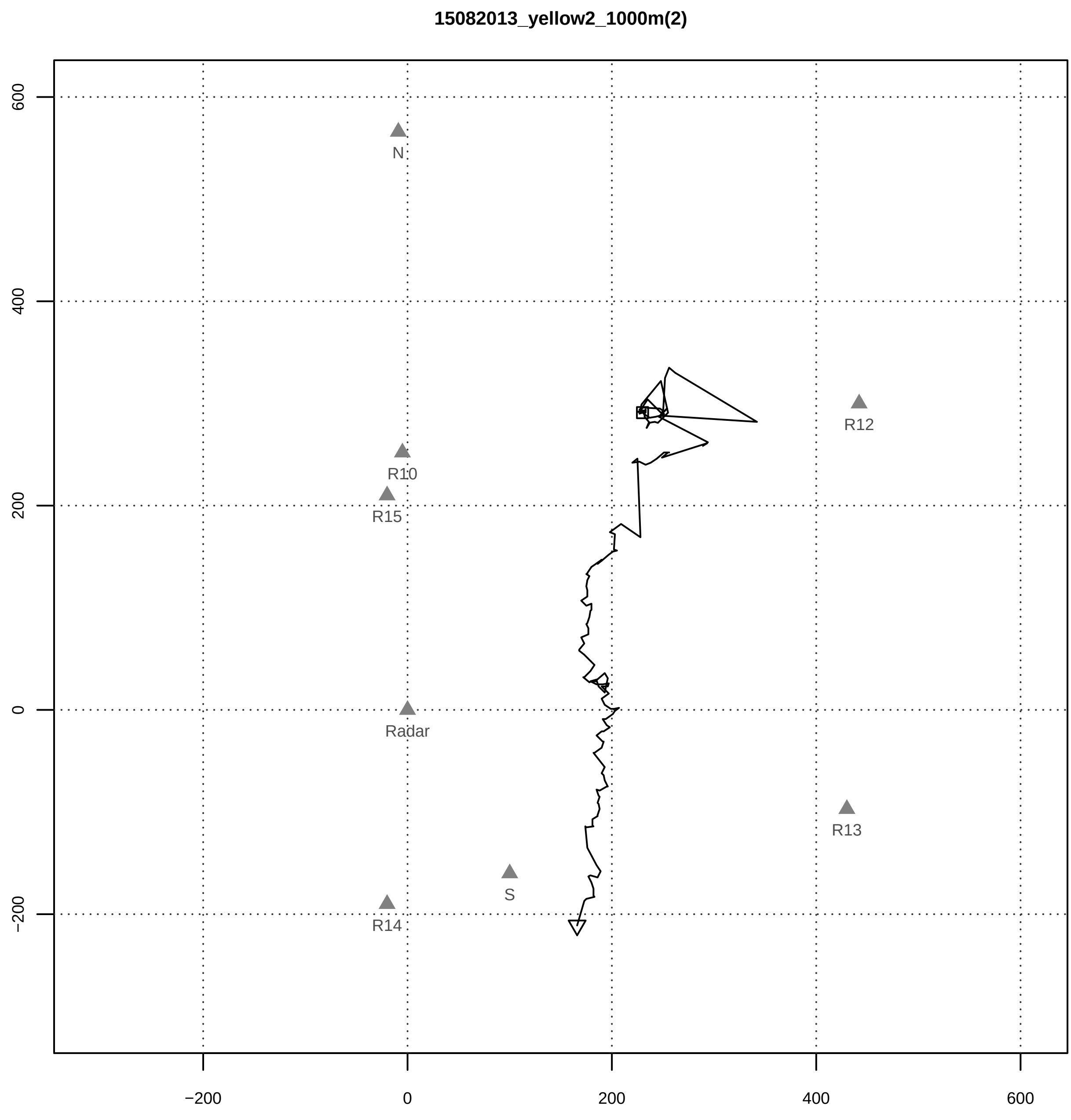
15082013_yellow2_1000m(2)
N
R10
R15
Radar
R12
R13
S
R14
600
400
200
0
−200
−200
0
200
400
600

15082013_yellow2_1000m

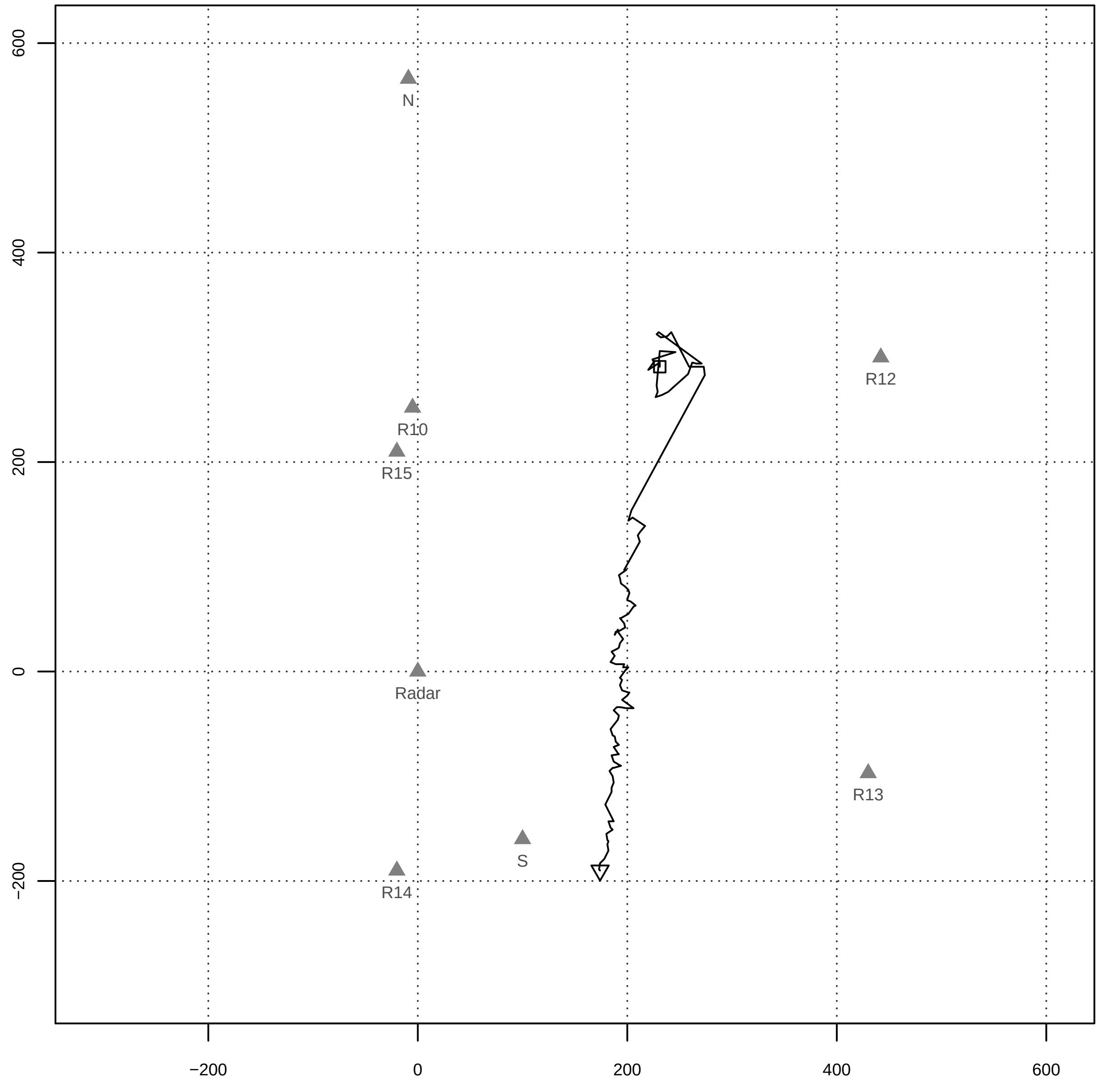

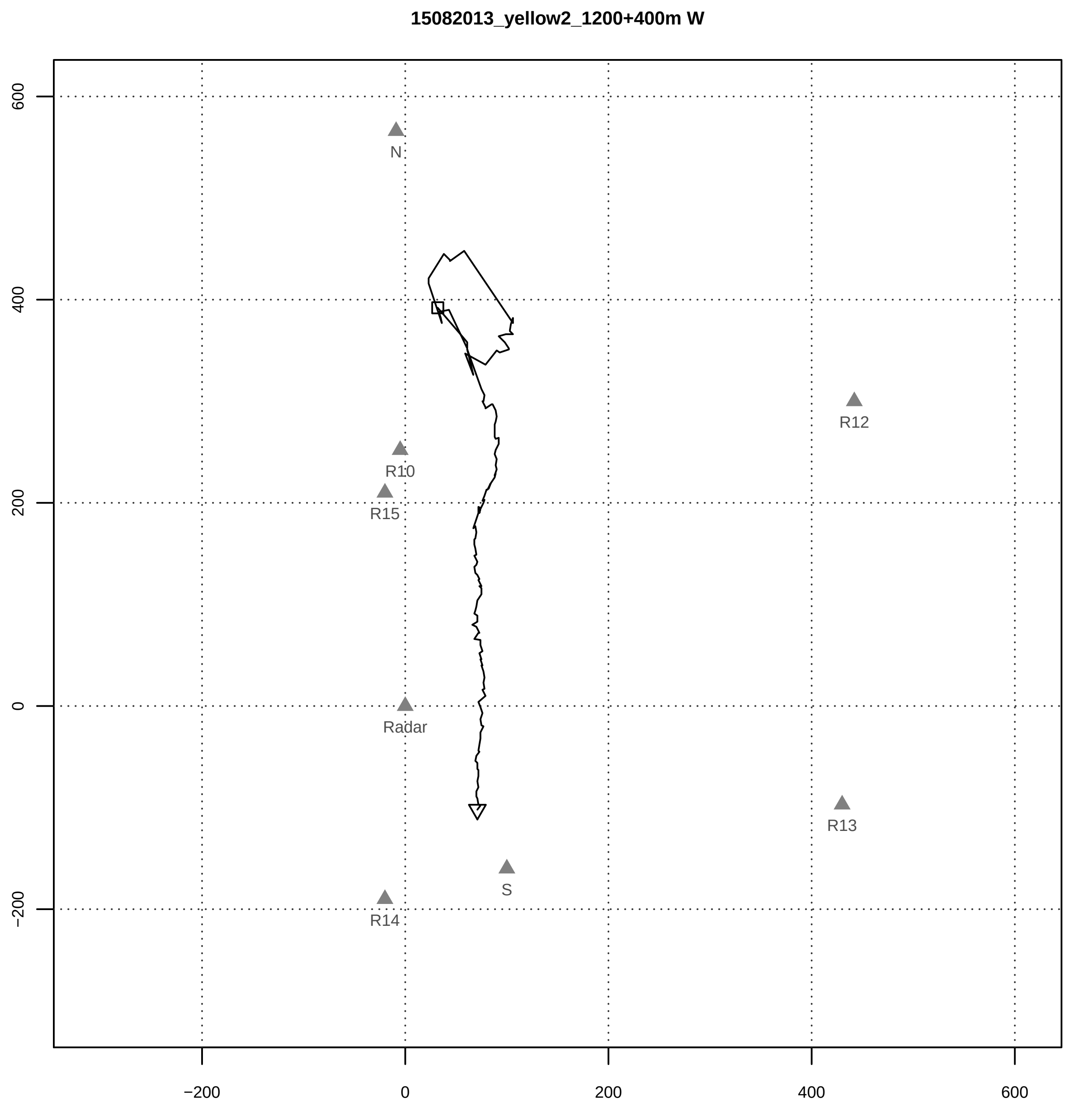
15082013_yellow2_1200+400m W
N
R10
R15
R12
Radar
R13
S
R14
−200
0
200
400
600
−200
0
200
400
600

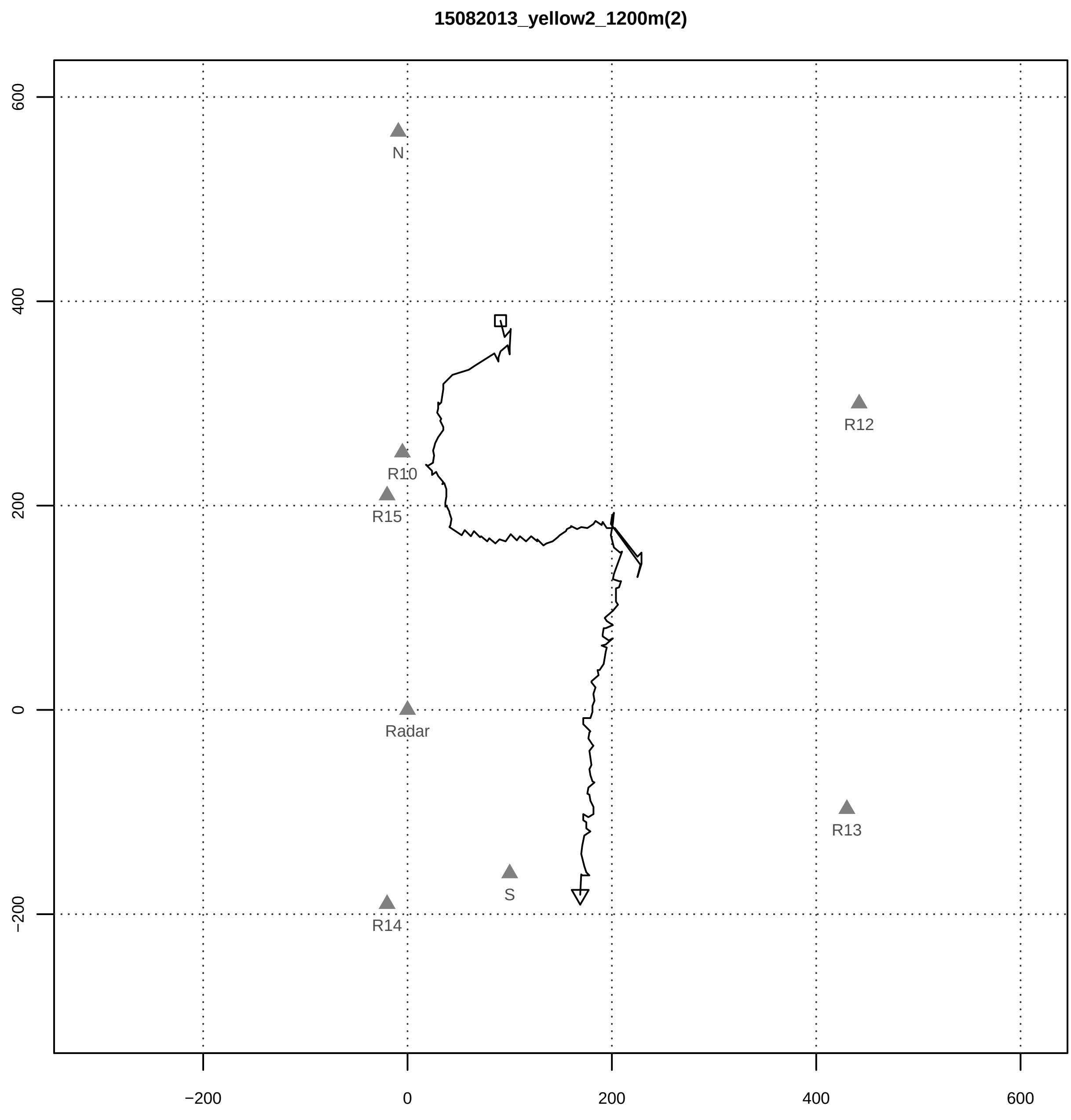
15082013_yellow2_1200m(2)
N
R10
R15
R12
Radar
R13
S
R14
600
400
200
0
−200
−200
0
200
400
600

15082013_yellow2_1200m

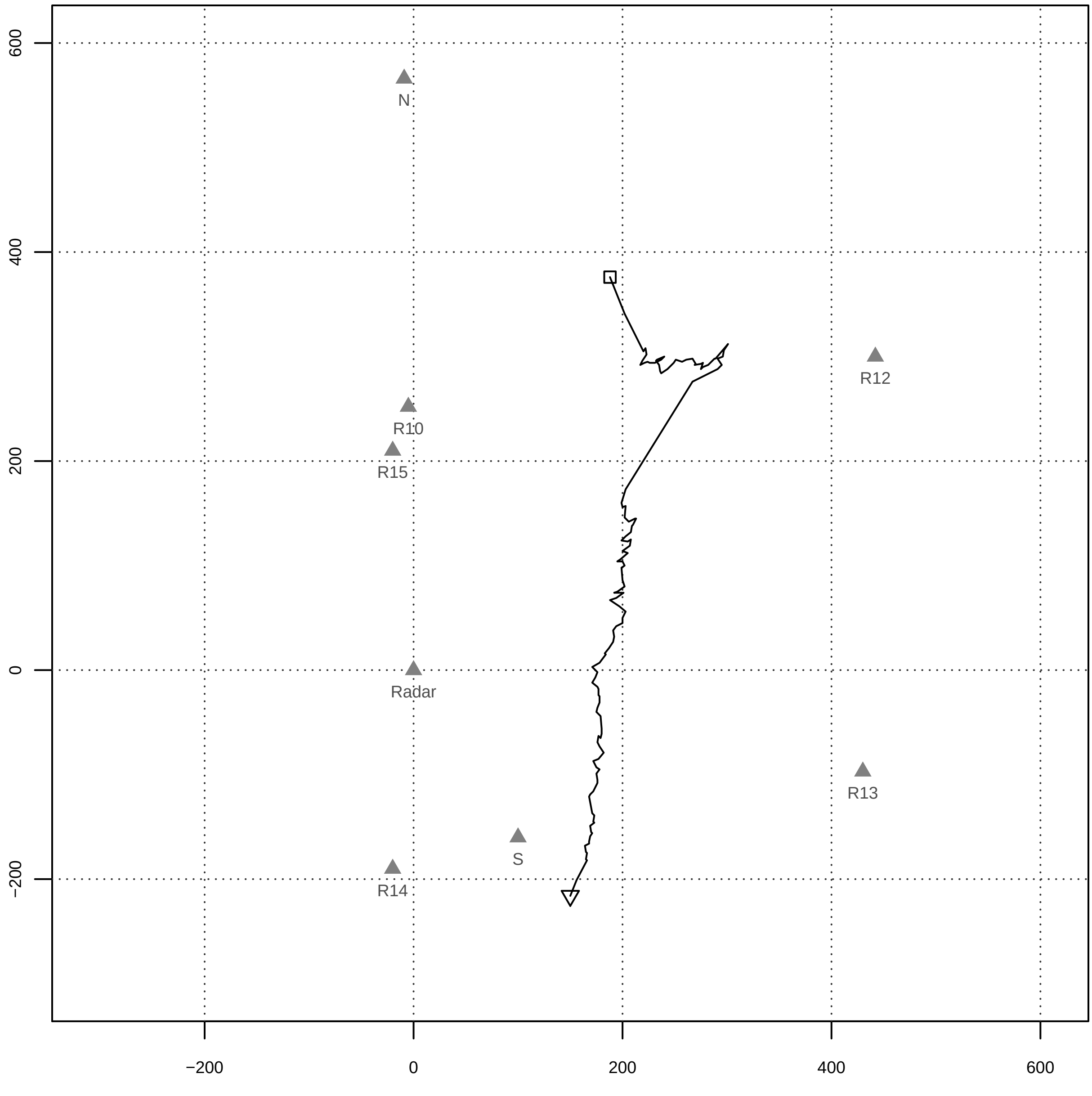

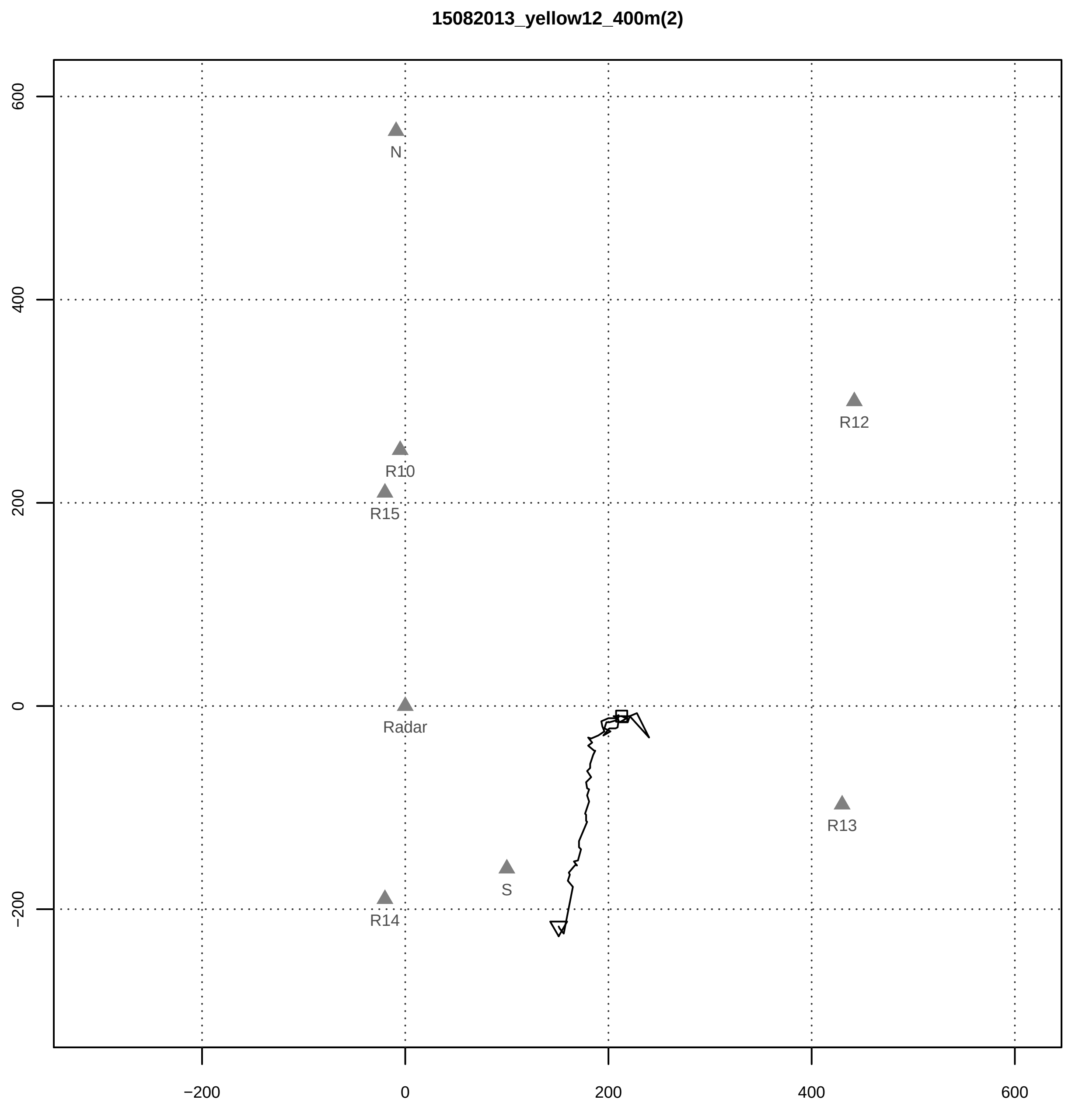
15082013_yellow12_400m(2)
N
R12
R10
R15
Radar
R13
S
R14
600
400
200
0
−200
−200
0
200
400
600

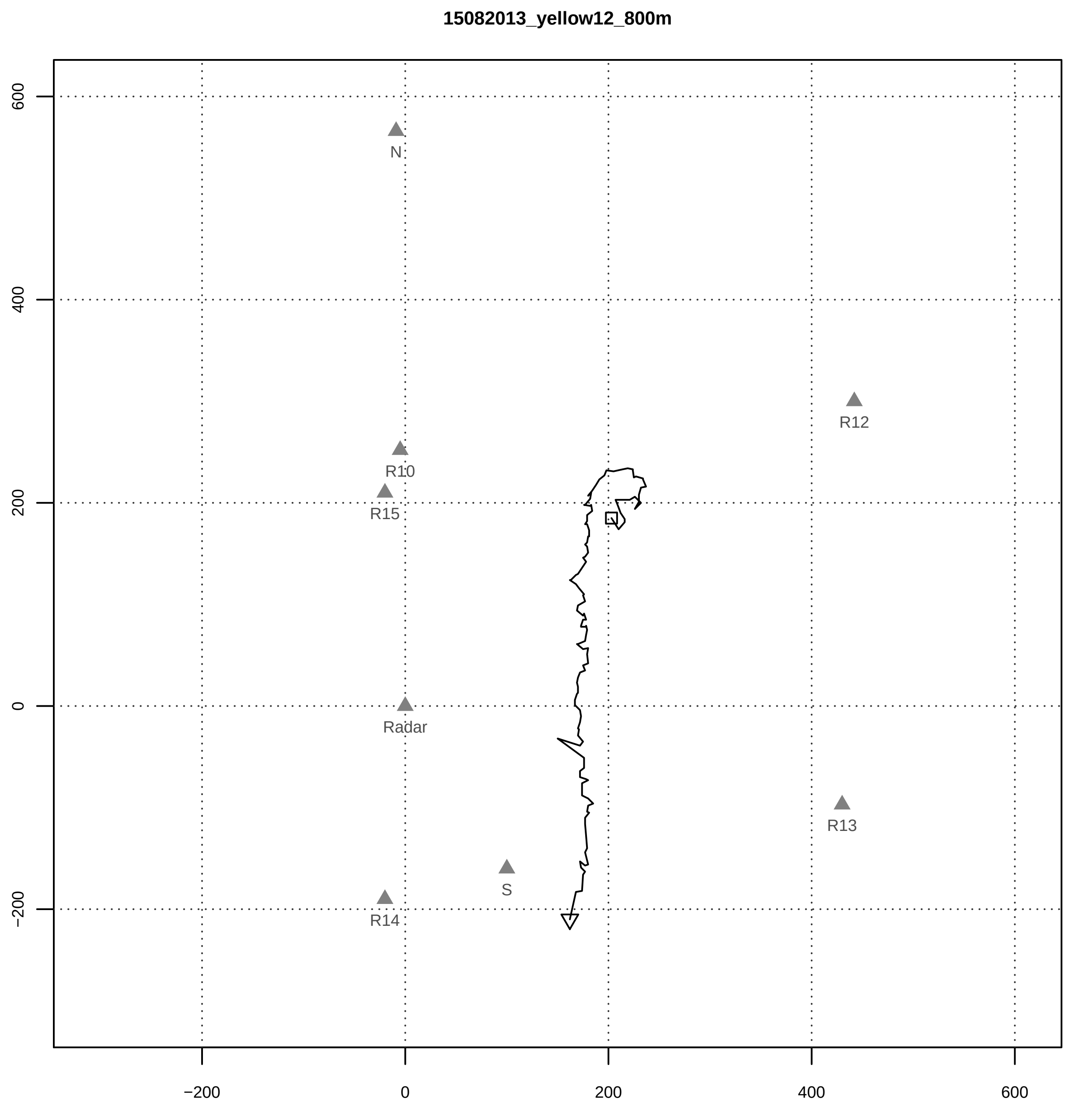

15082013_yellow12_800m
N
R10
R15
R12
Radar
R13
S
R14
600
400
200
0
−200
−200
0
200
400
600

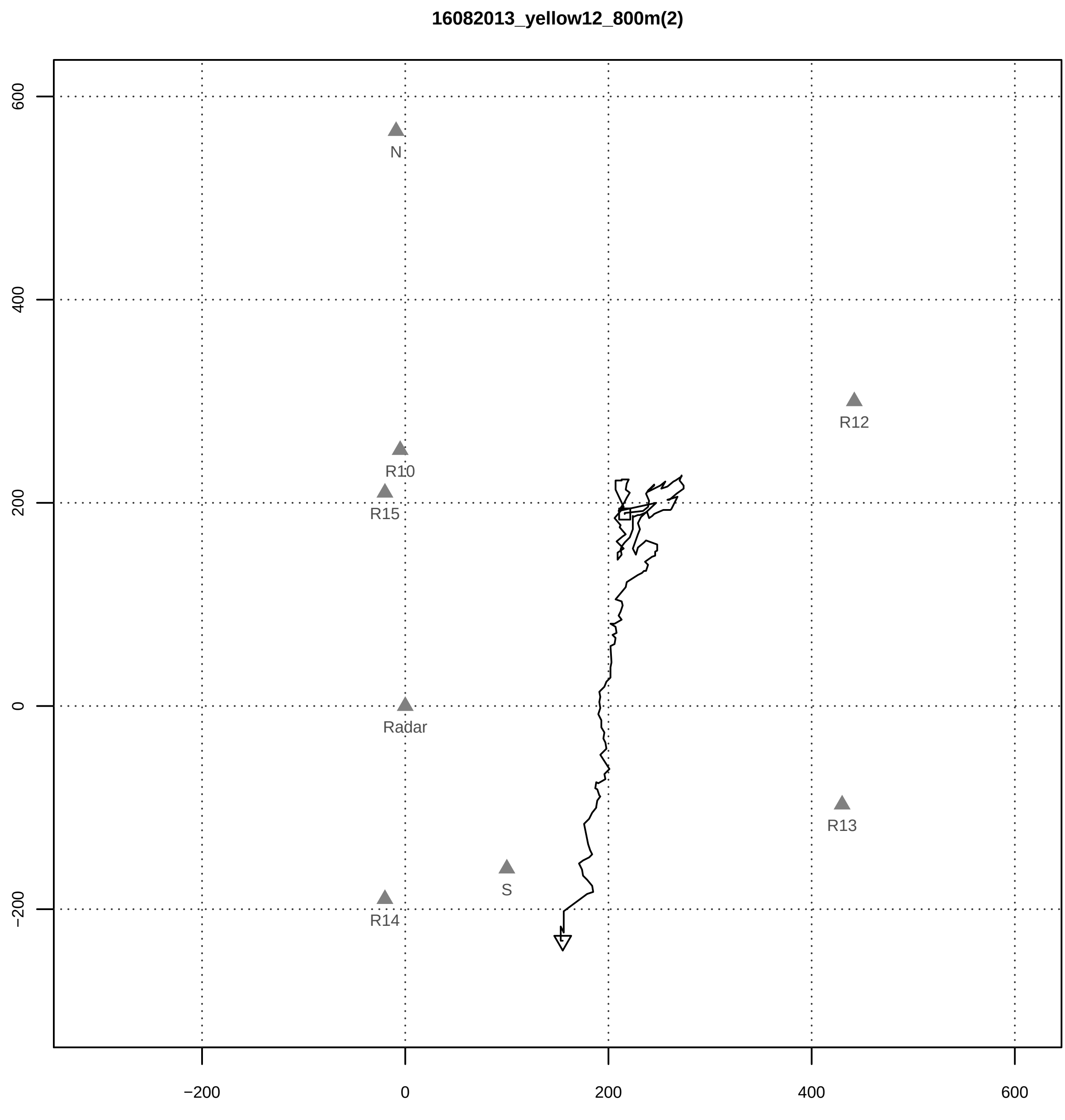
16082013_yellow12_800m(2)
N
R12
R10
R15
Radar
R13
S
R14
600
400
200
0
−200
−200
0
200
400
600

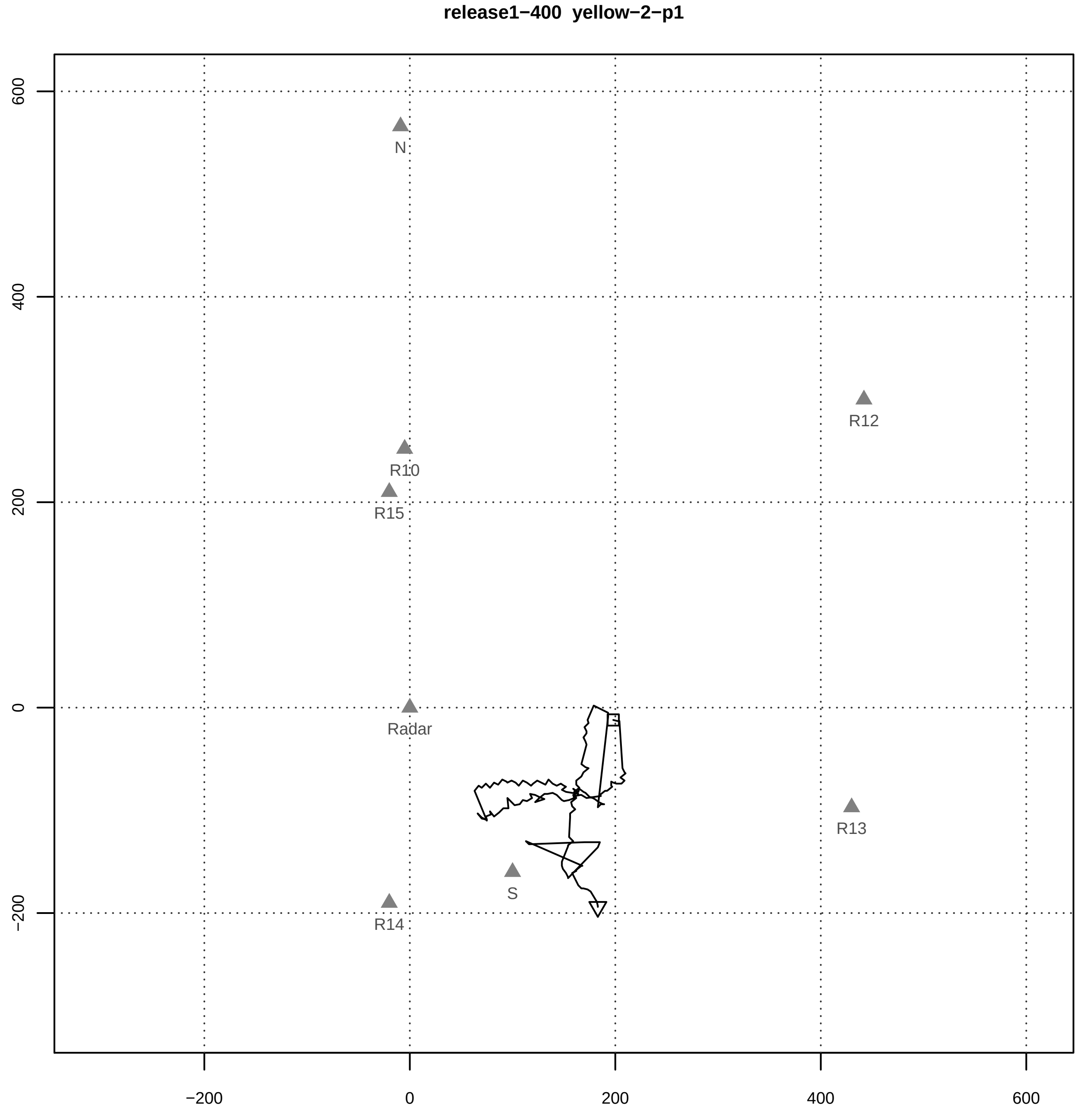

release1−400 yellow−2−p1
N
R12
R10
R15
Radar
R13
S
R14
600
400
200
0
−200
−200
0
200
400
600

release1−1200 blue−3−p1

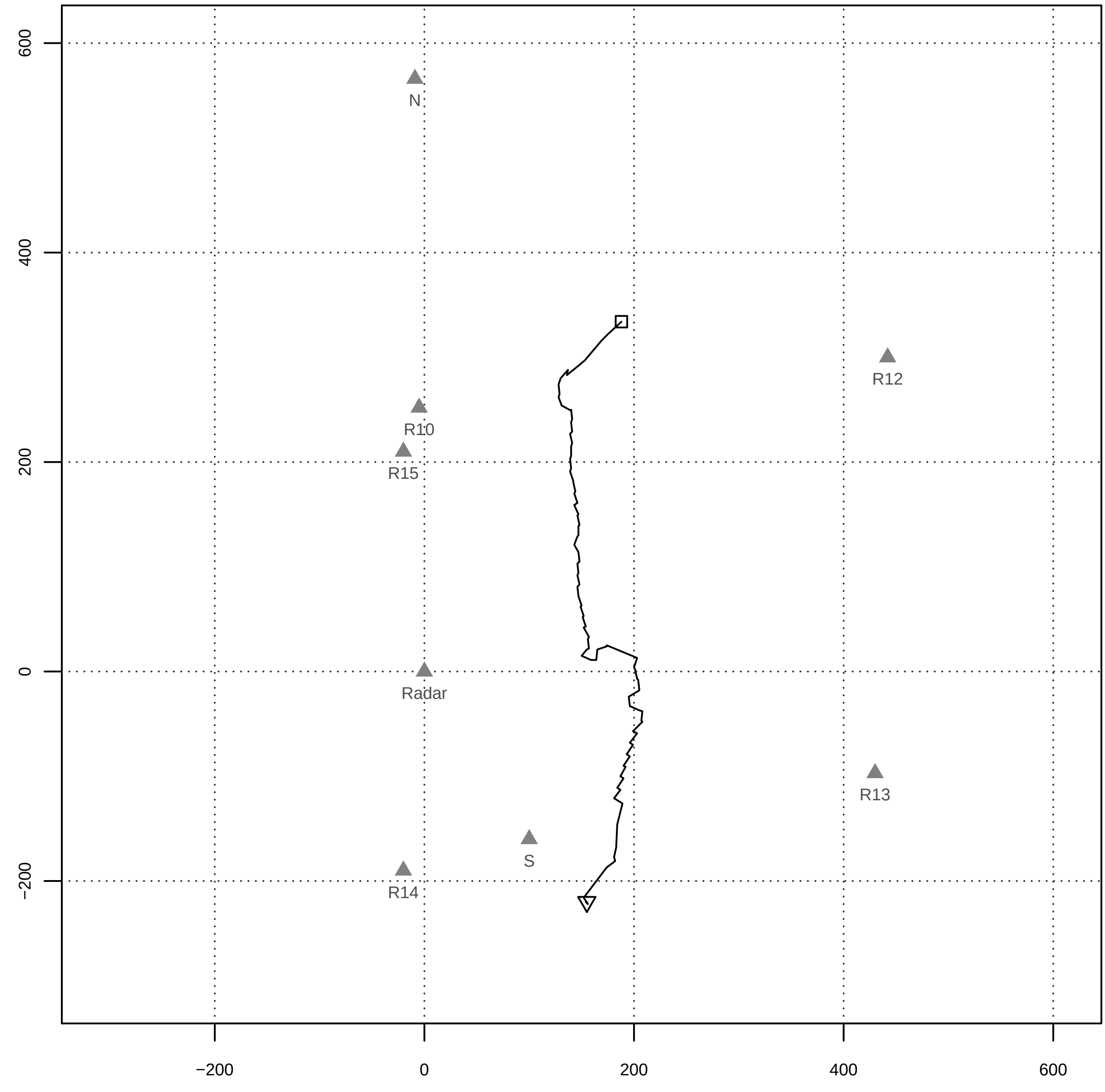

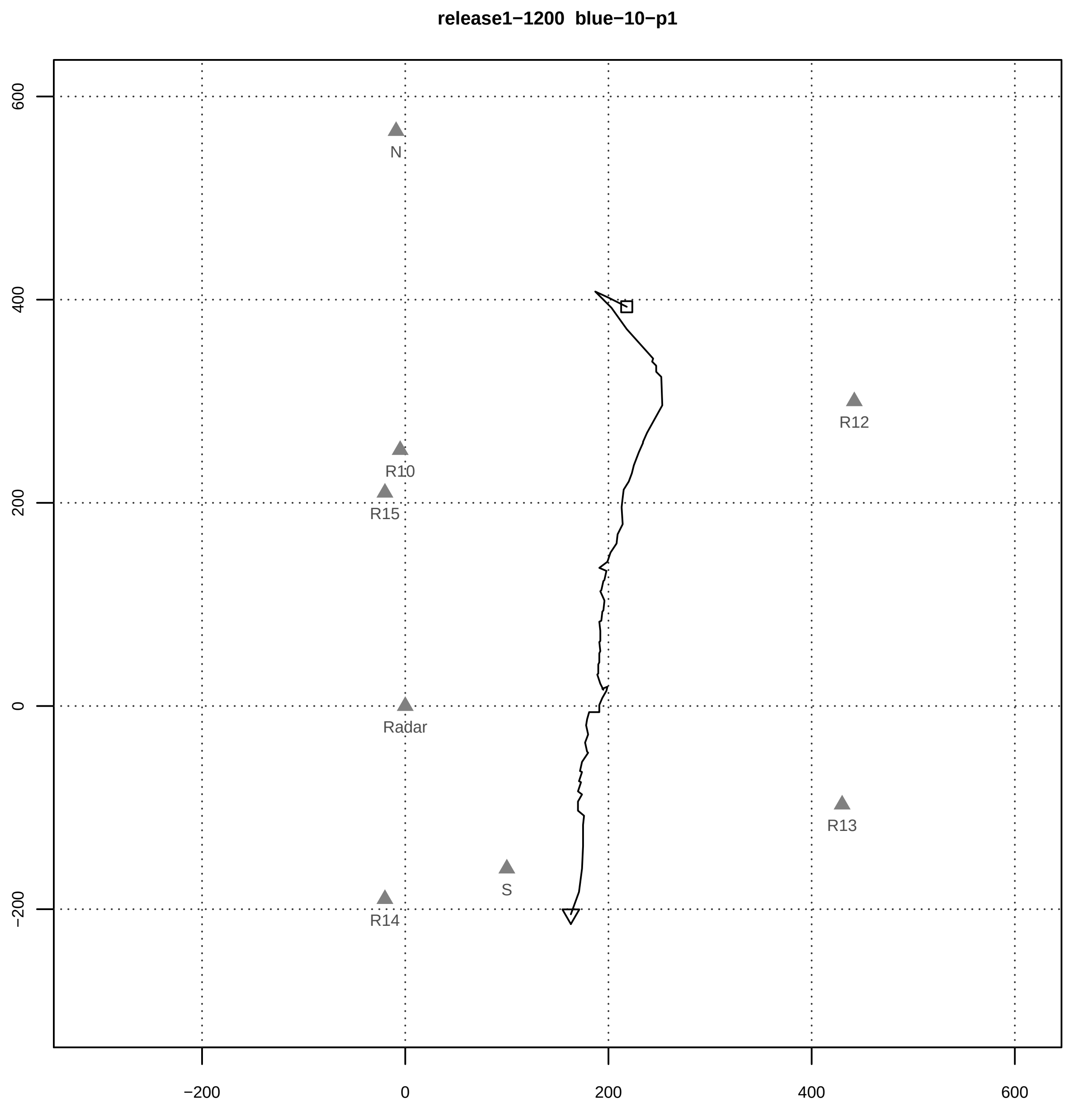

release1−1200 blue−10−p1
600
400
200
0
−200
−200
0
200
400
600
N
R12
R10
R15
Radar
R13
S
R14

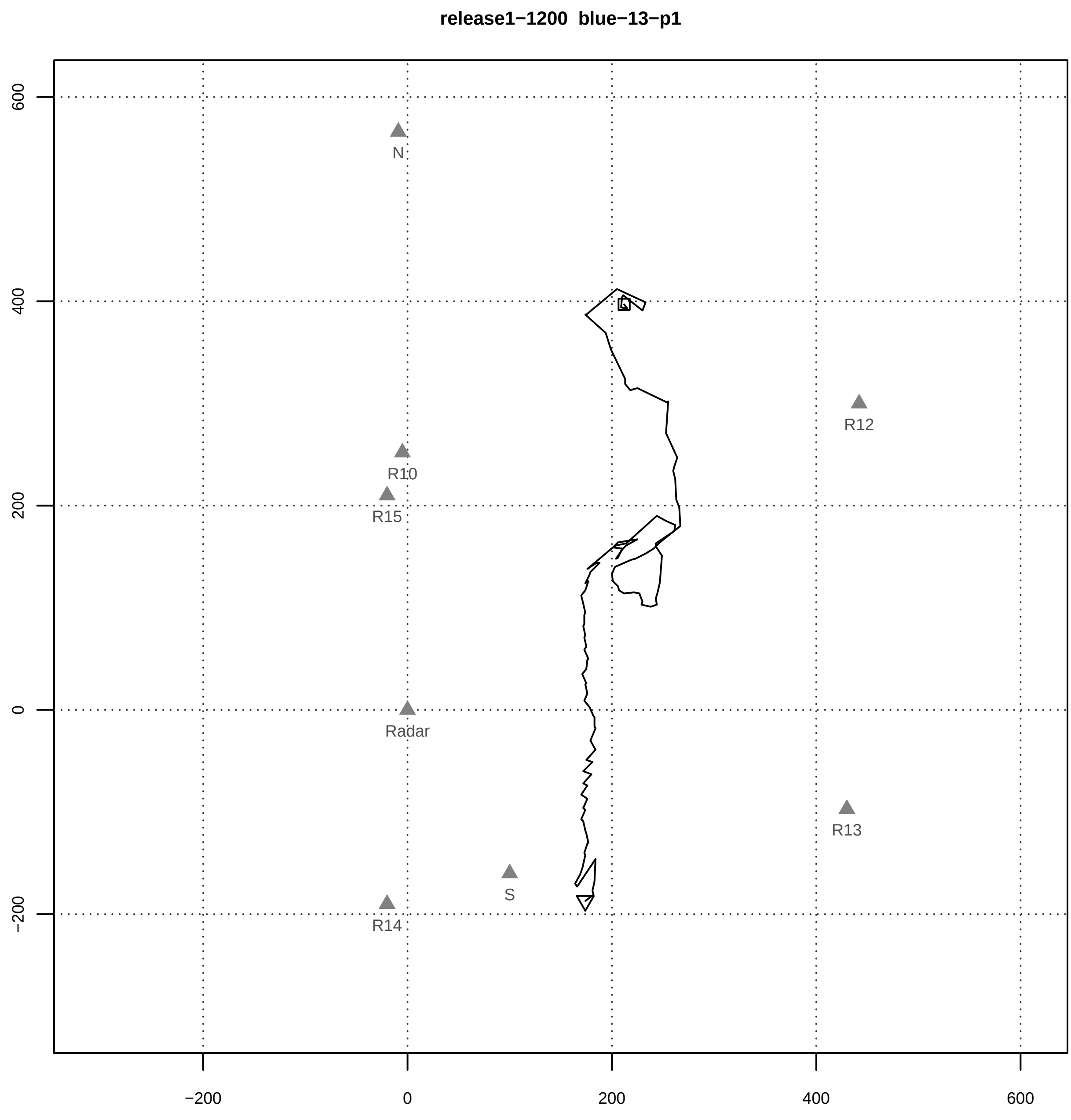
release1−1200 blue−13−p1
N
R12
R10
R15
Radar
R13
S
R14
−200
0
200
400
600
−200
0
200
400
600

release2−400 yellow−2−p1

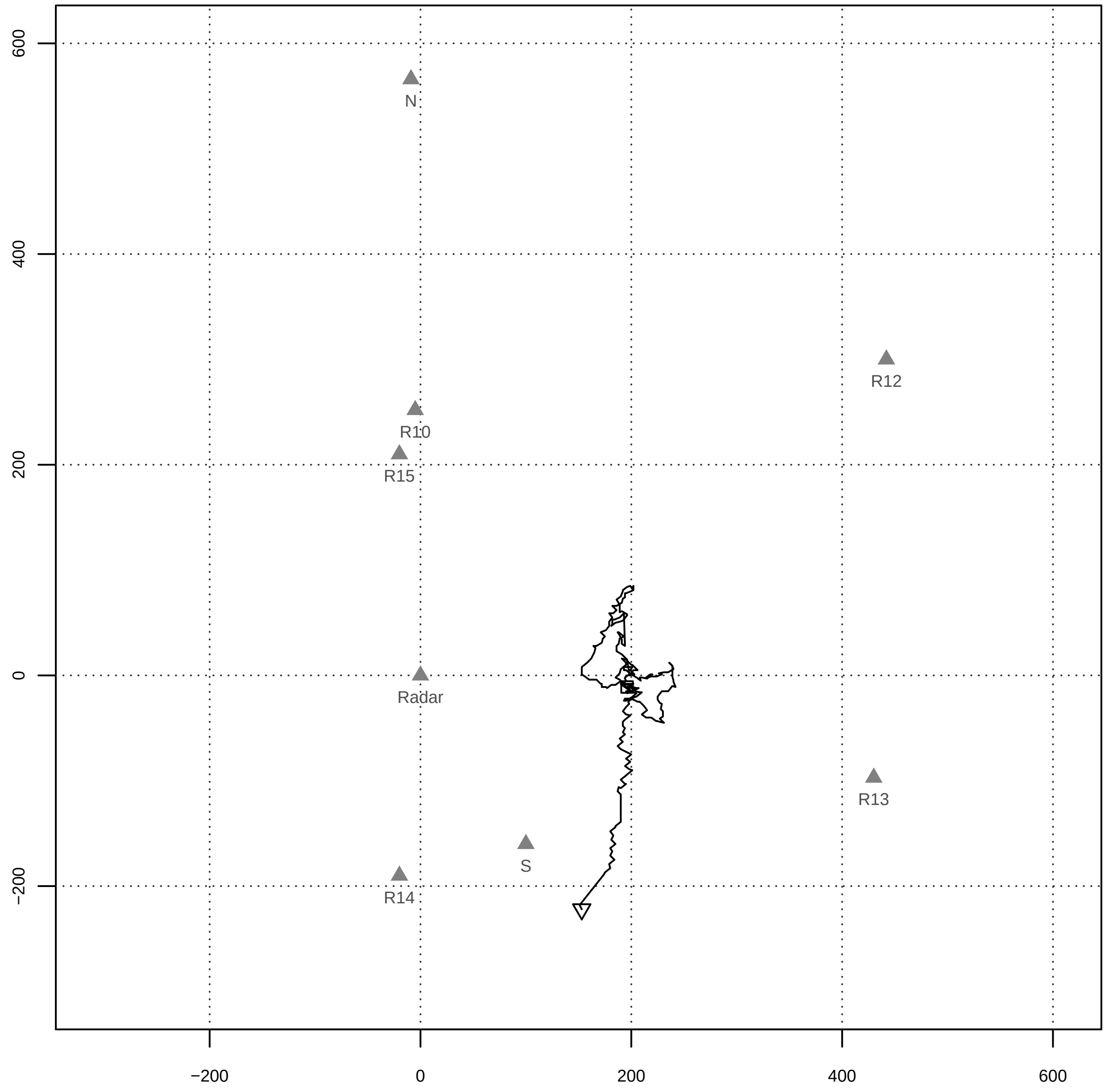

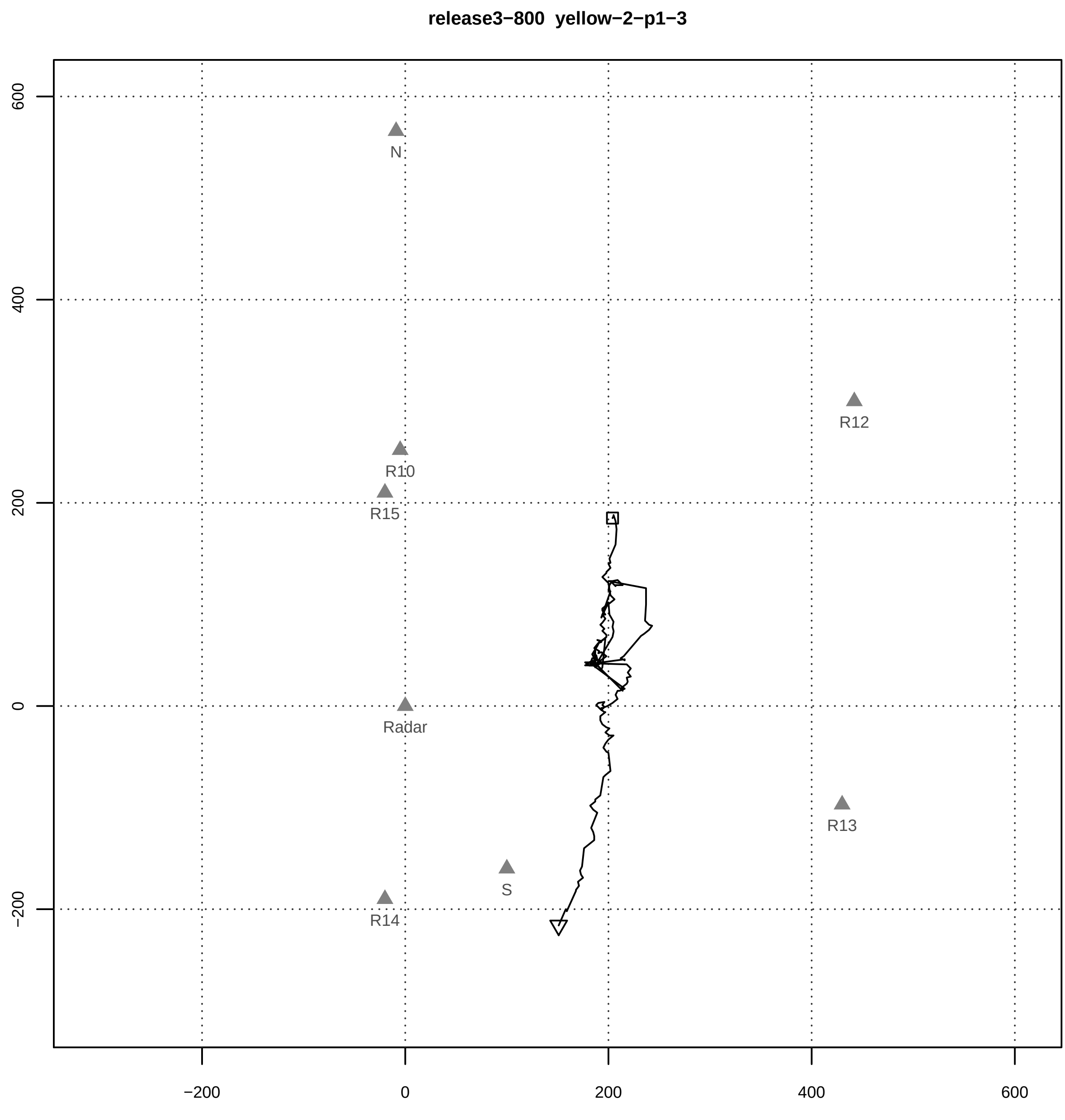
release3−800 yellow−2−p1−3
N
R12
R10
R15
Radar
R13
S
R14
−200
0
200
400
600

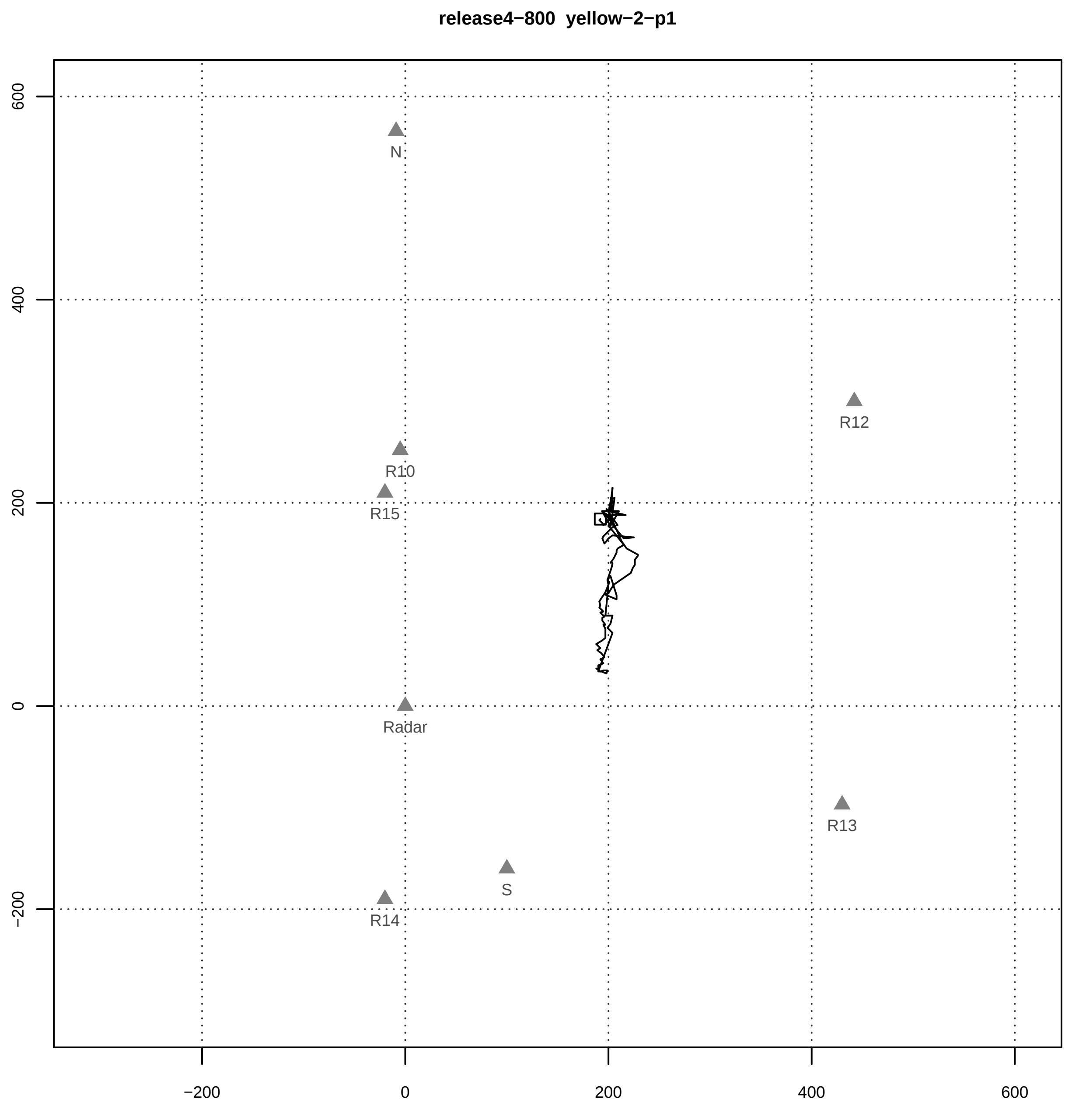
release4−800 yellow−2−p1
N
R10
R15
R12
Radar
R13
S
R14
600
400
200
0
−200
−200
0
200
400
600

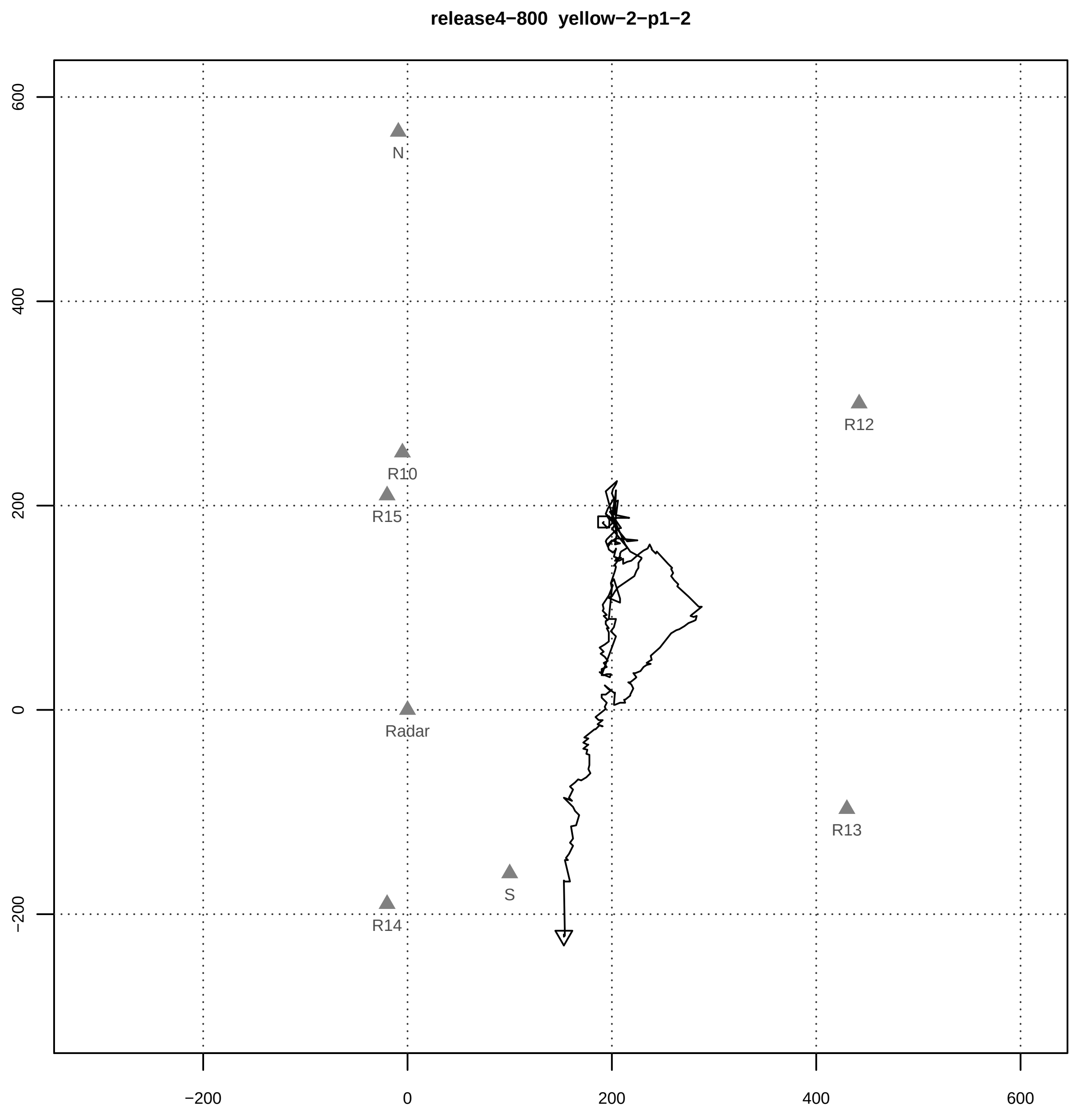
release4−800 yellow−2−p1−2
N
R12
R10
R15
Radar
R13
S
R14
600
400
200
0
−200
−200
0
200
400
600

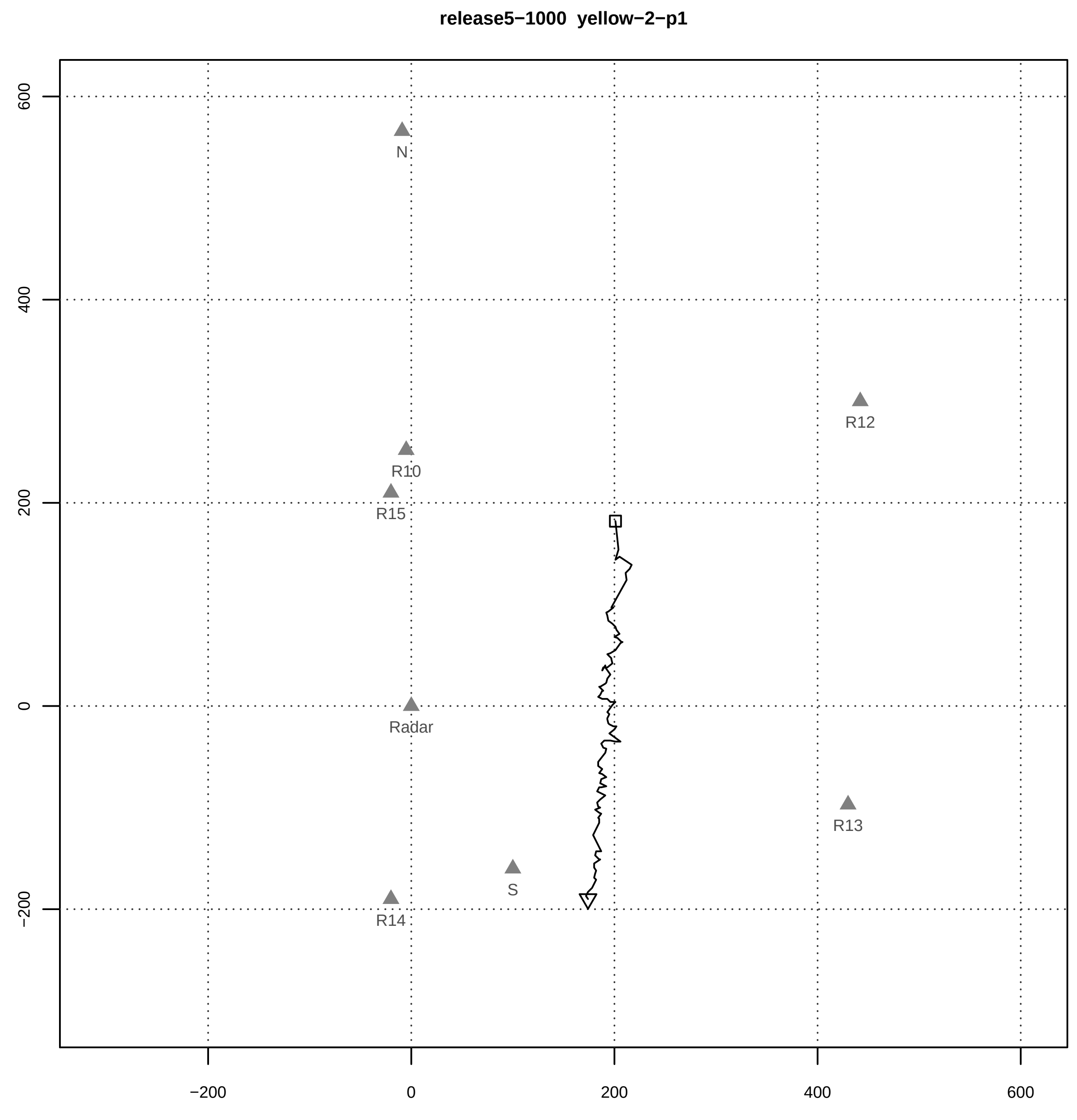
release5−1000 yellow−2−p1
N
R12
R10
R15
Radar
R13
S
R14
600
400
200
0
−200
−200
0
200
400
600

release6−1000 yellow−2−p1

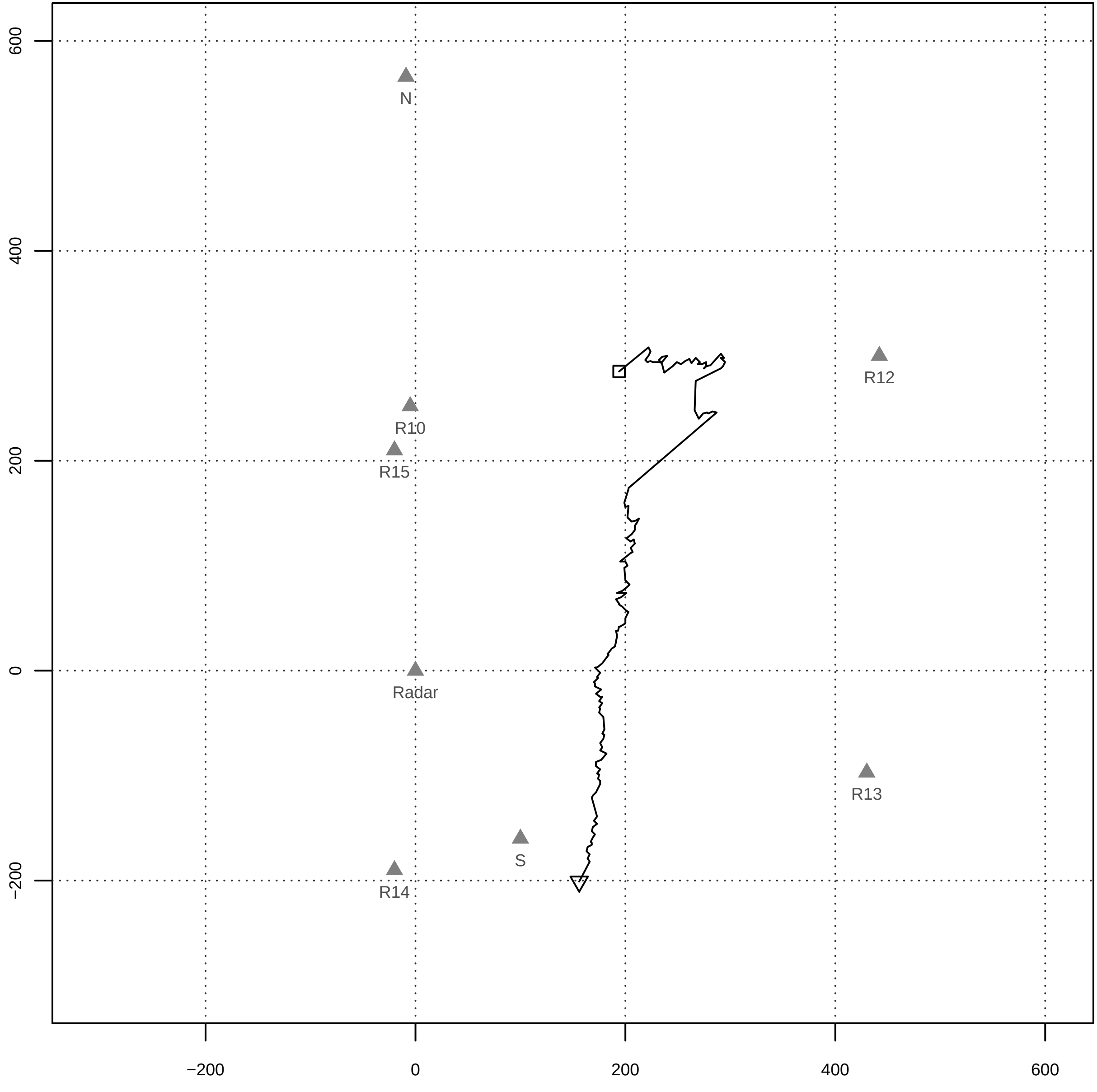

release7−1200 yellow−2−p1

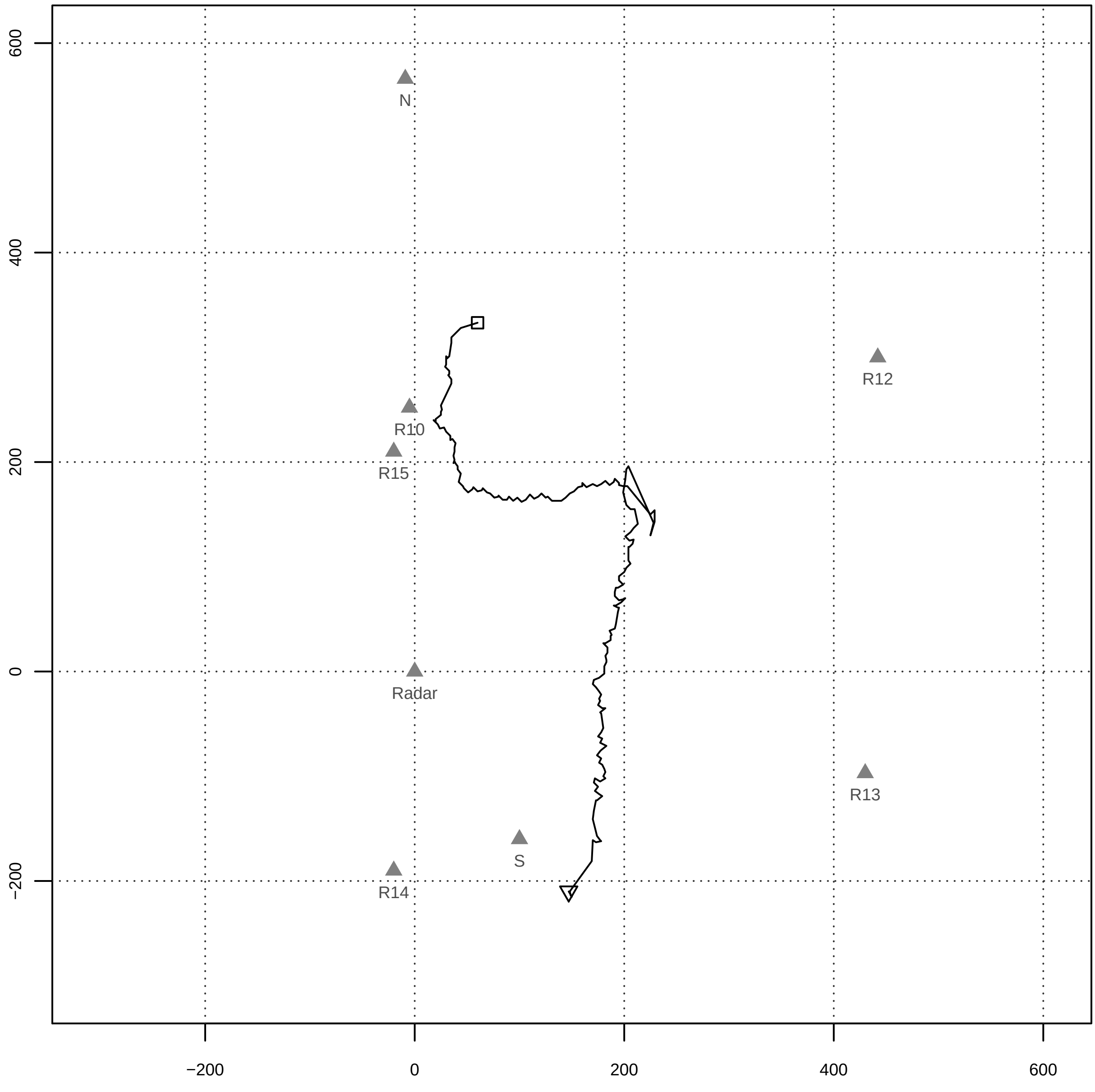

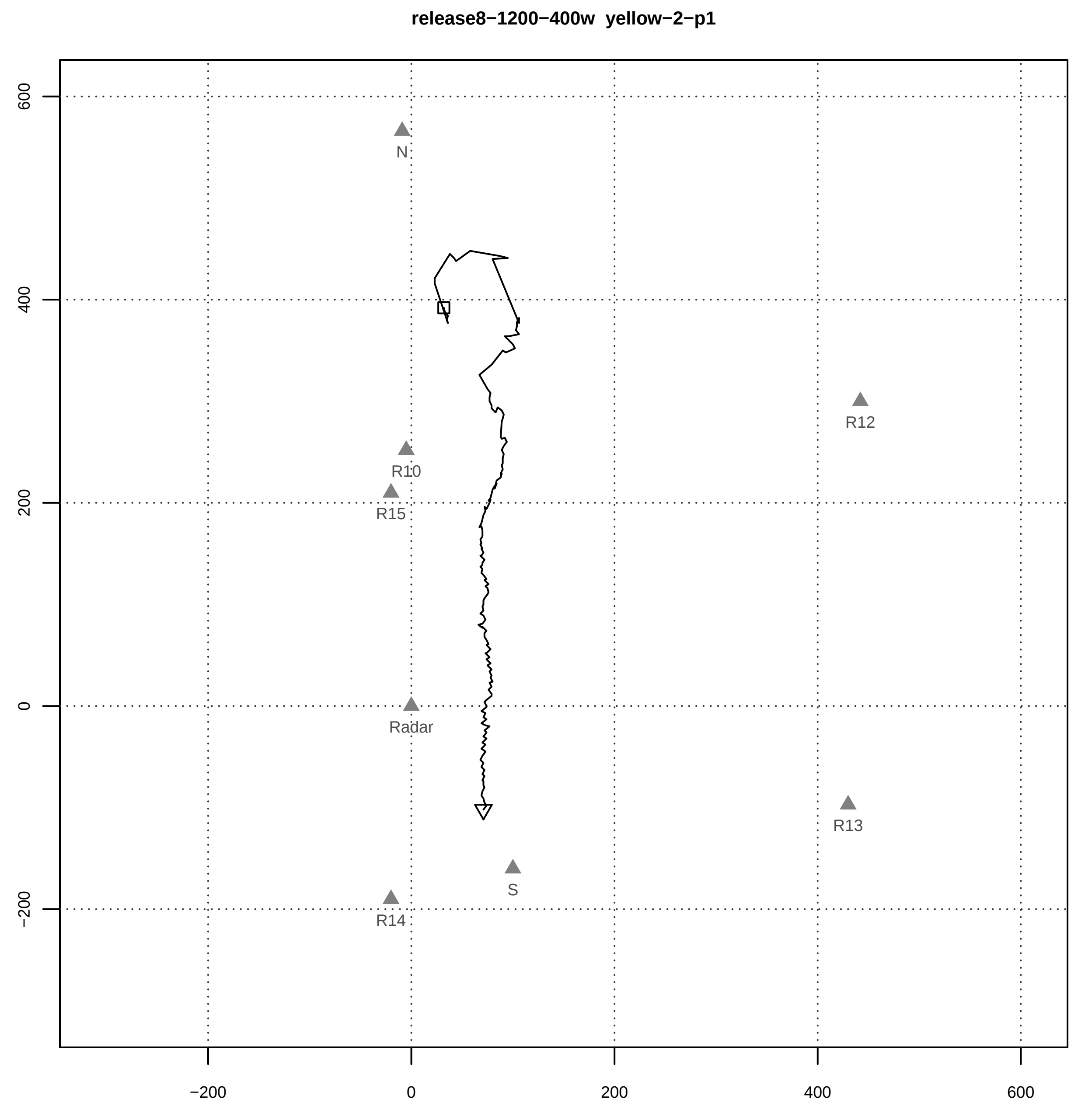
release8−1200−400w yellow−2−p1
N
R12
R10
R15
Radar
R13
S
R14
600
400
200
0
−200
−200
0
200
400
600

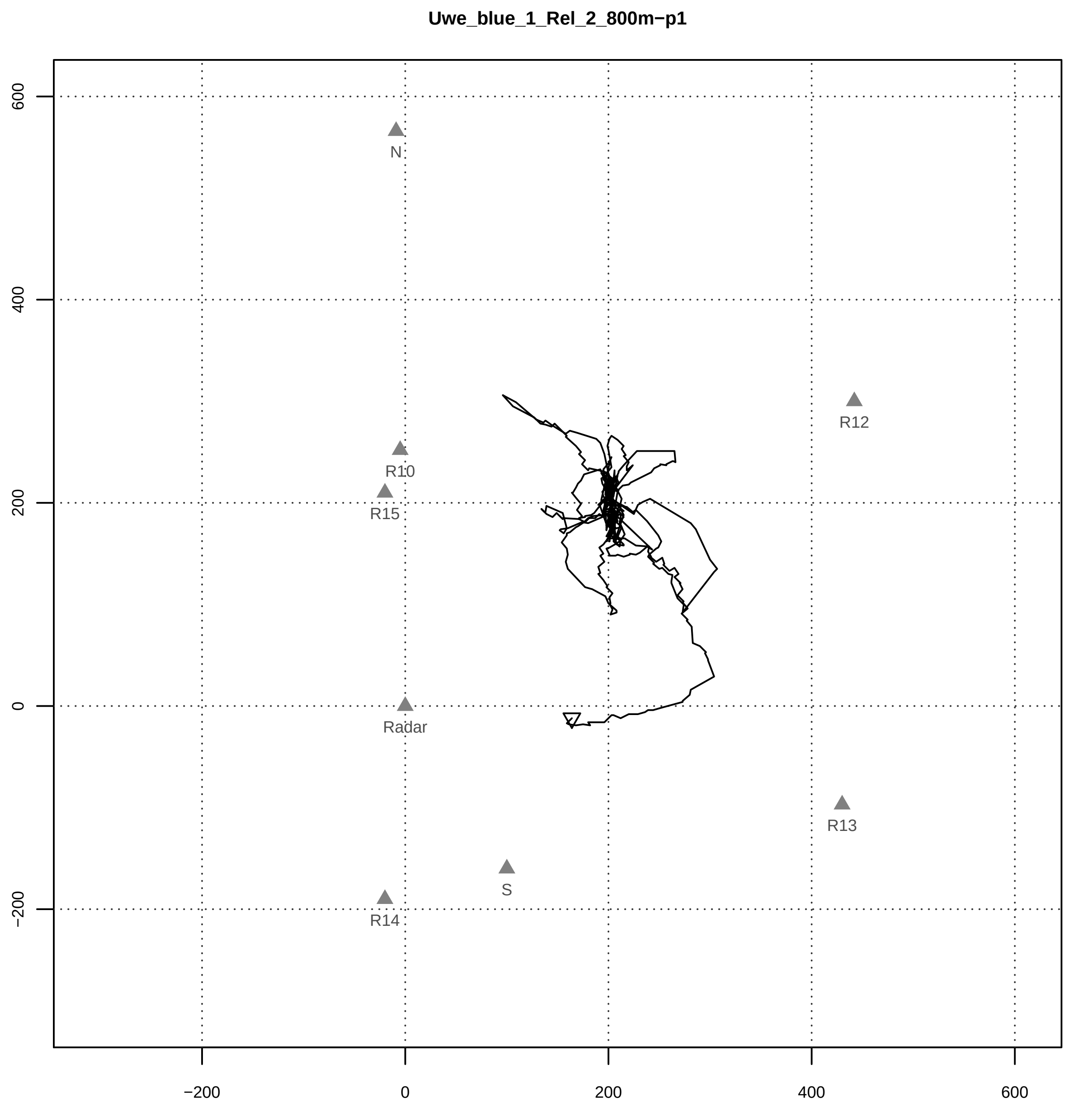

Uwe_blue_1_Rel_2_800m−p1
600
400
200
0
−200
−200
0
200
400
600
N
R10
R15
R12
Radar
R13
S
R14

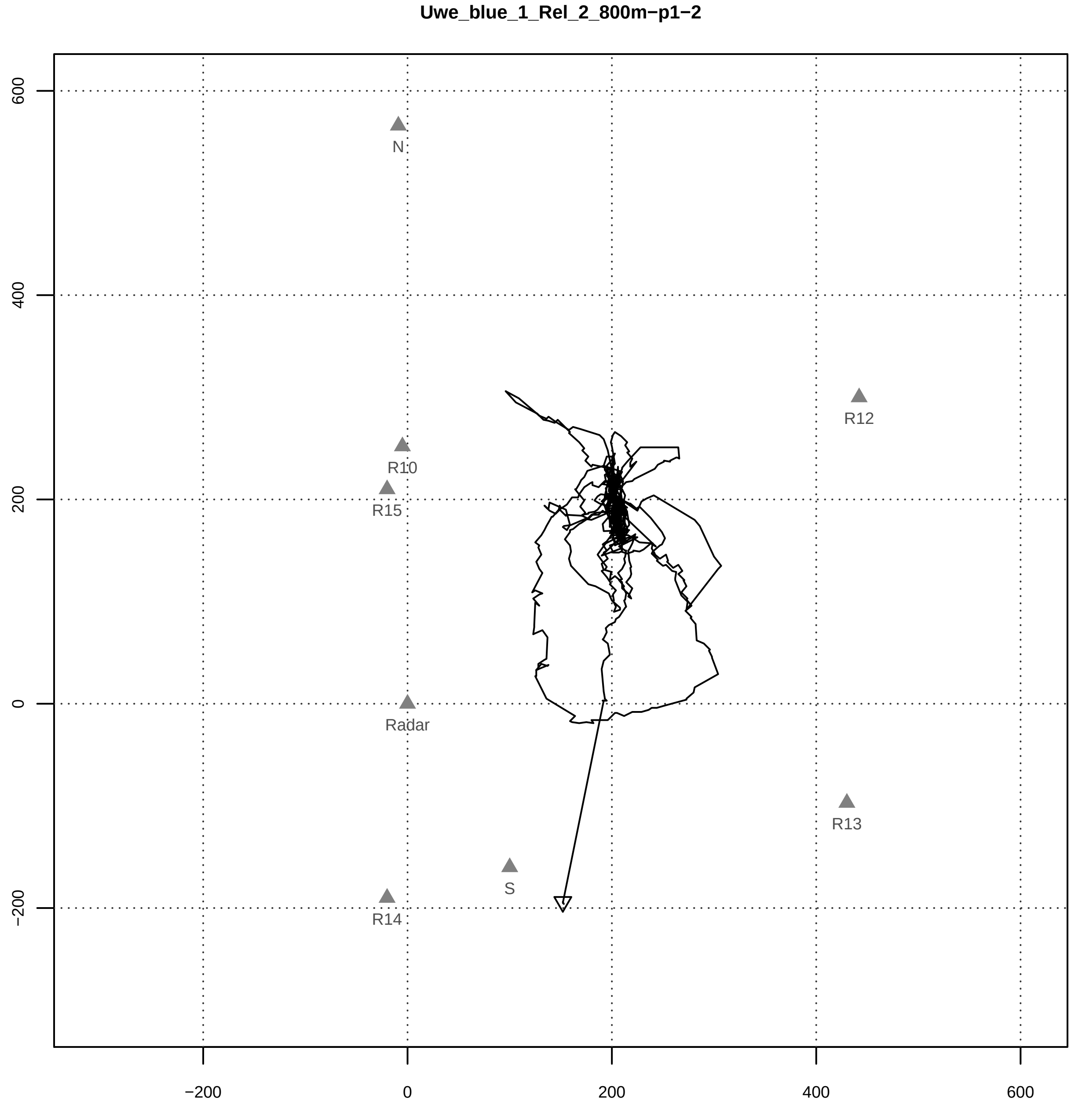

Uwe_blue_1_Rel_2_800m−p1−2
600
400
200
0
−200
−200
0
200
400
600
N
R10
R15
R12
Radar
R13
S
R14

Uwe_blue_3_Rel_1_800m

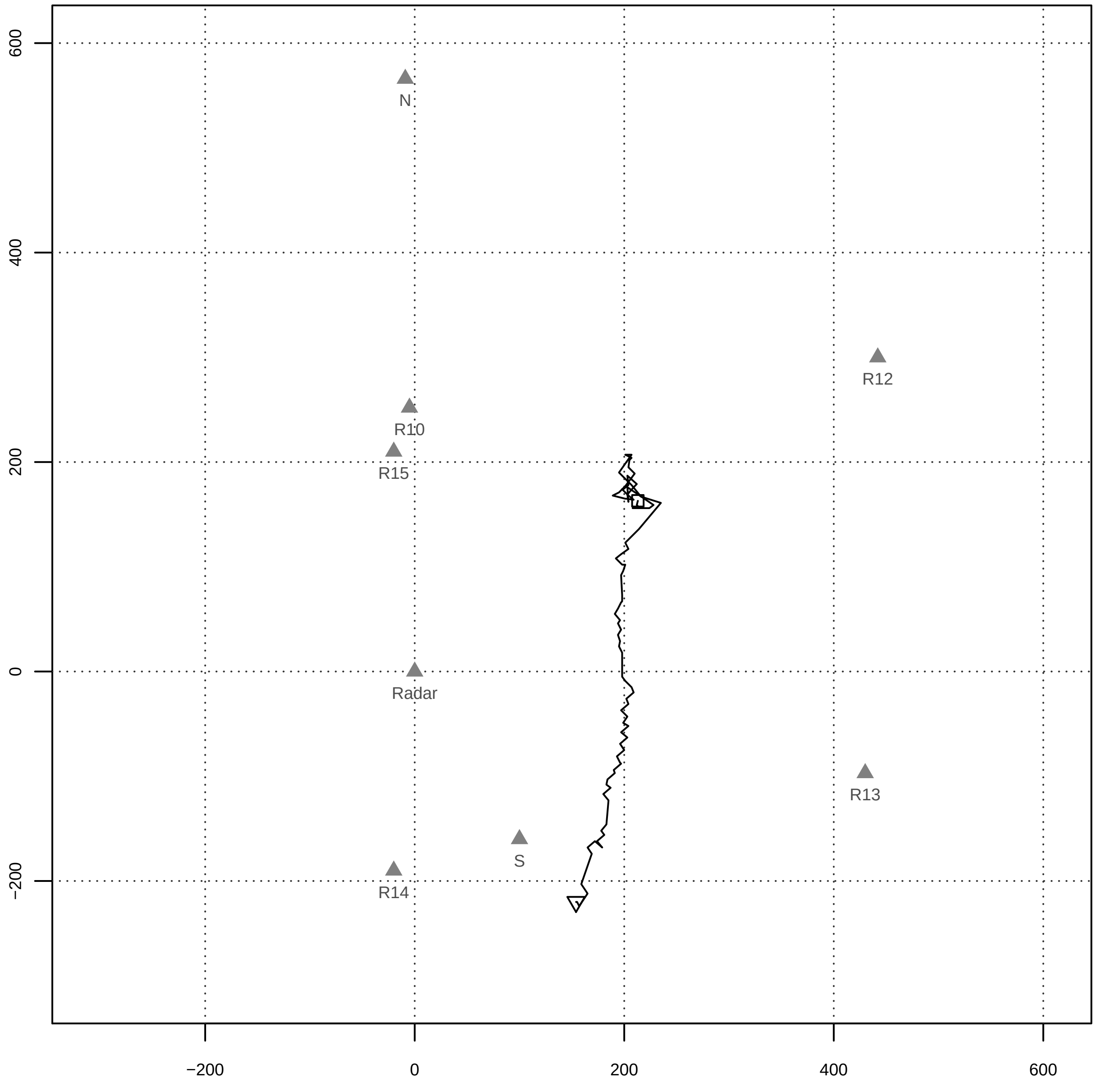

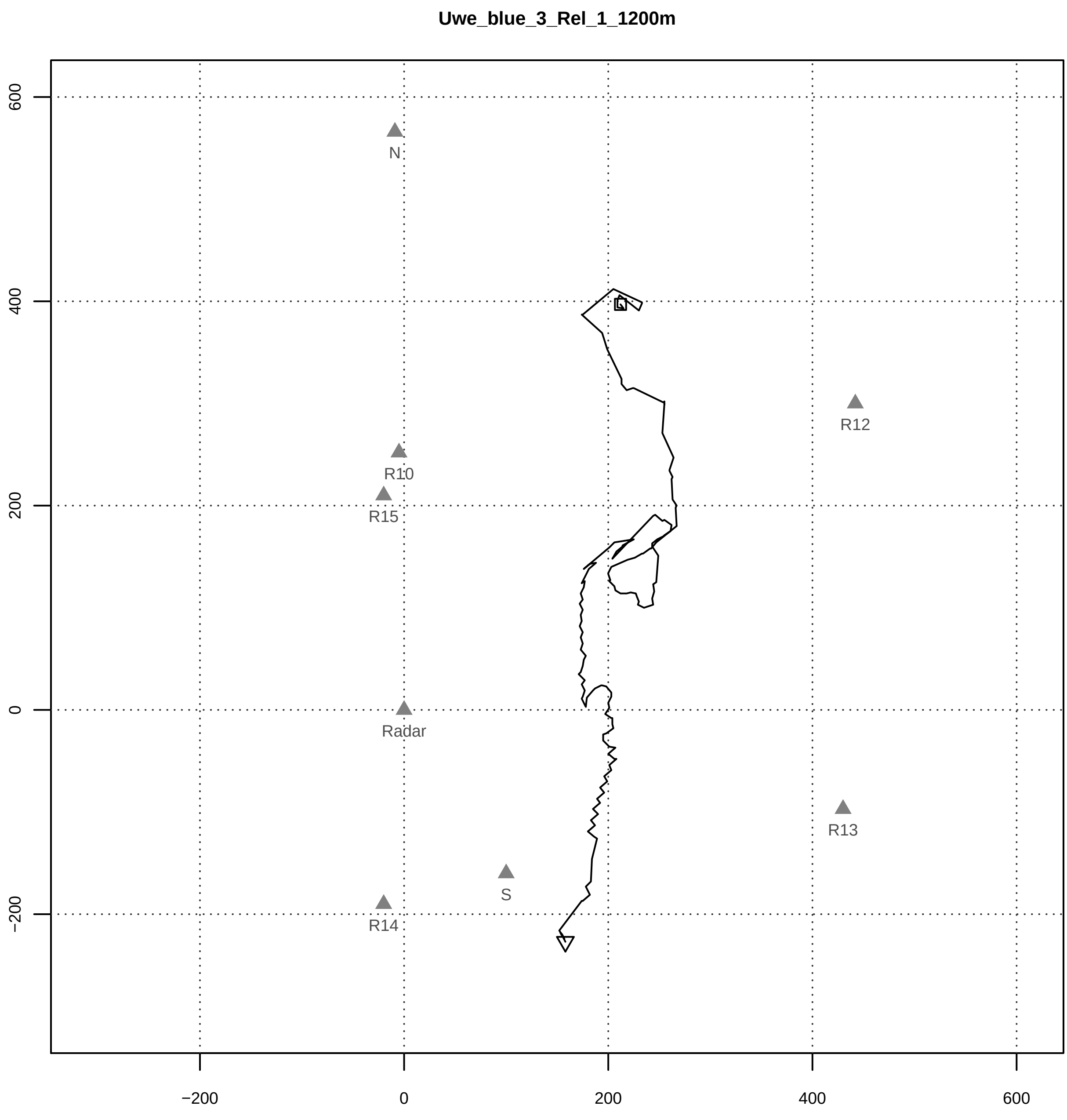
Uwe_blue_3_Rel_1_1200m
N
R10
R15
R12
Radar
R13
S
R14
600
400
200
0
−200
−200
0
200
400
600

Uwe_blue_10_Rel_1_1200m

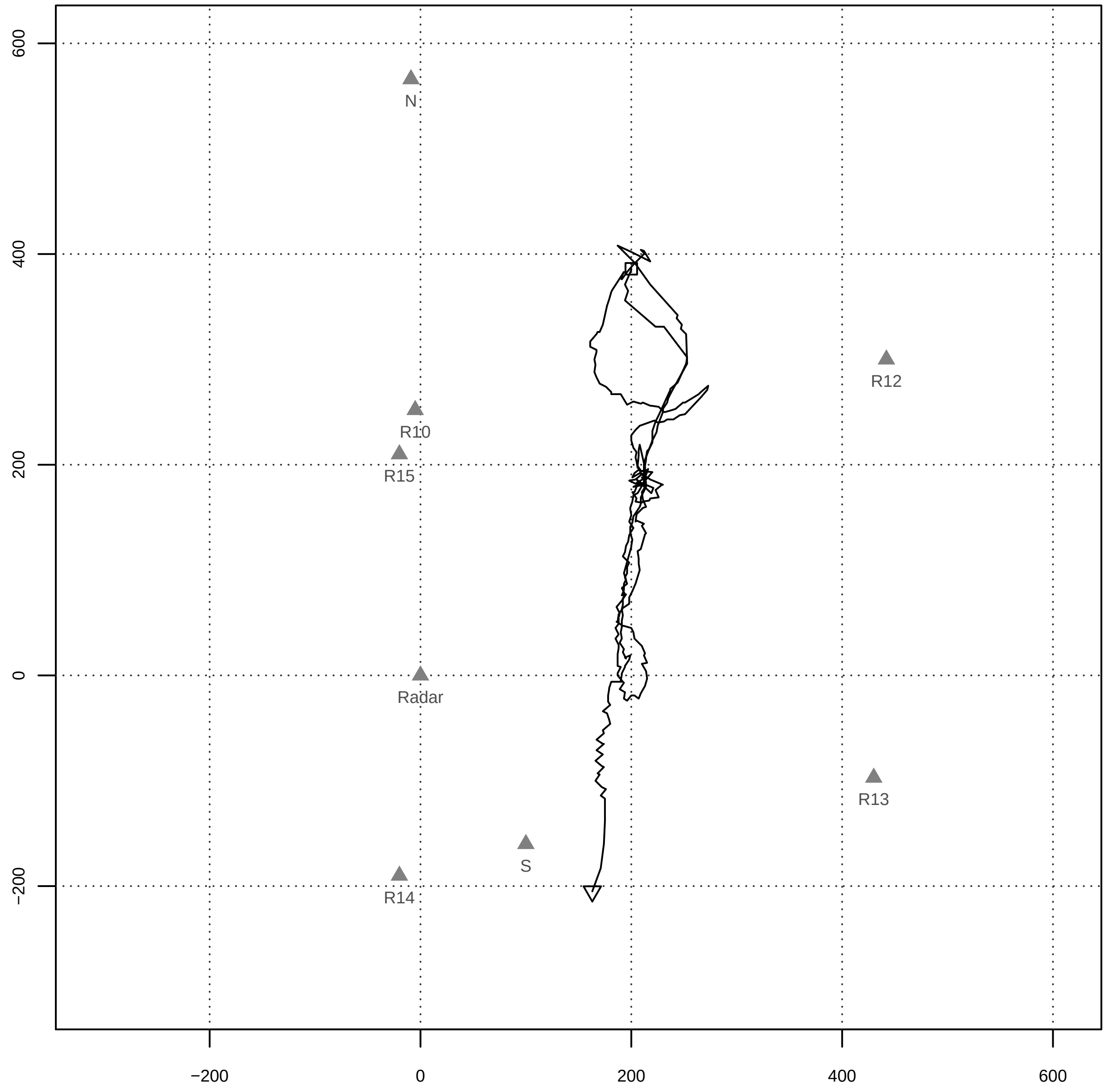

Uwe_blue_10_Rel_1_1200m−p1

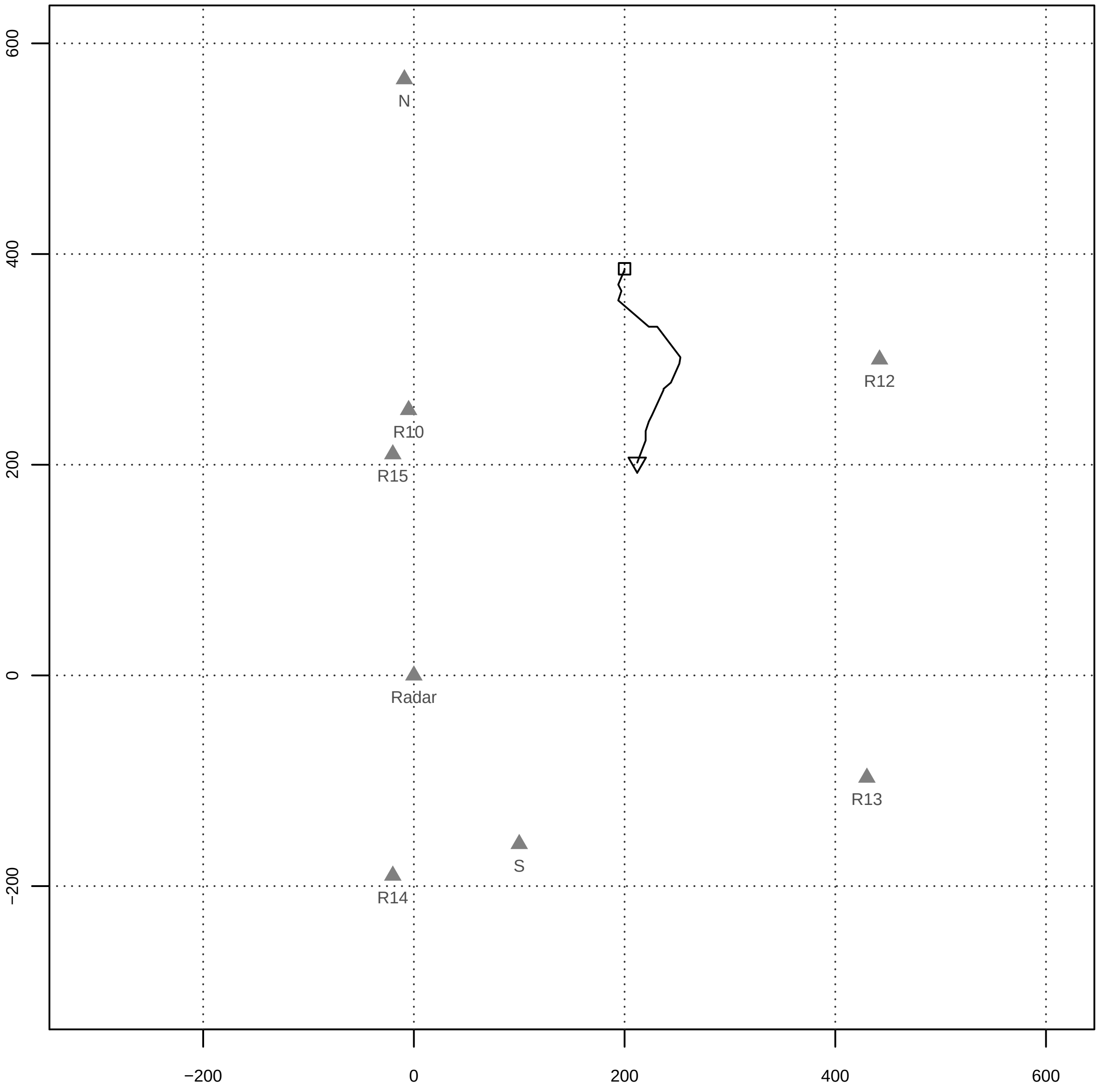

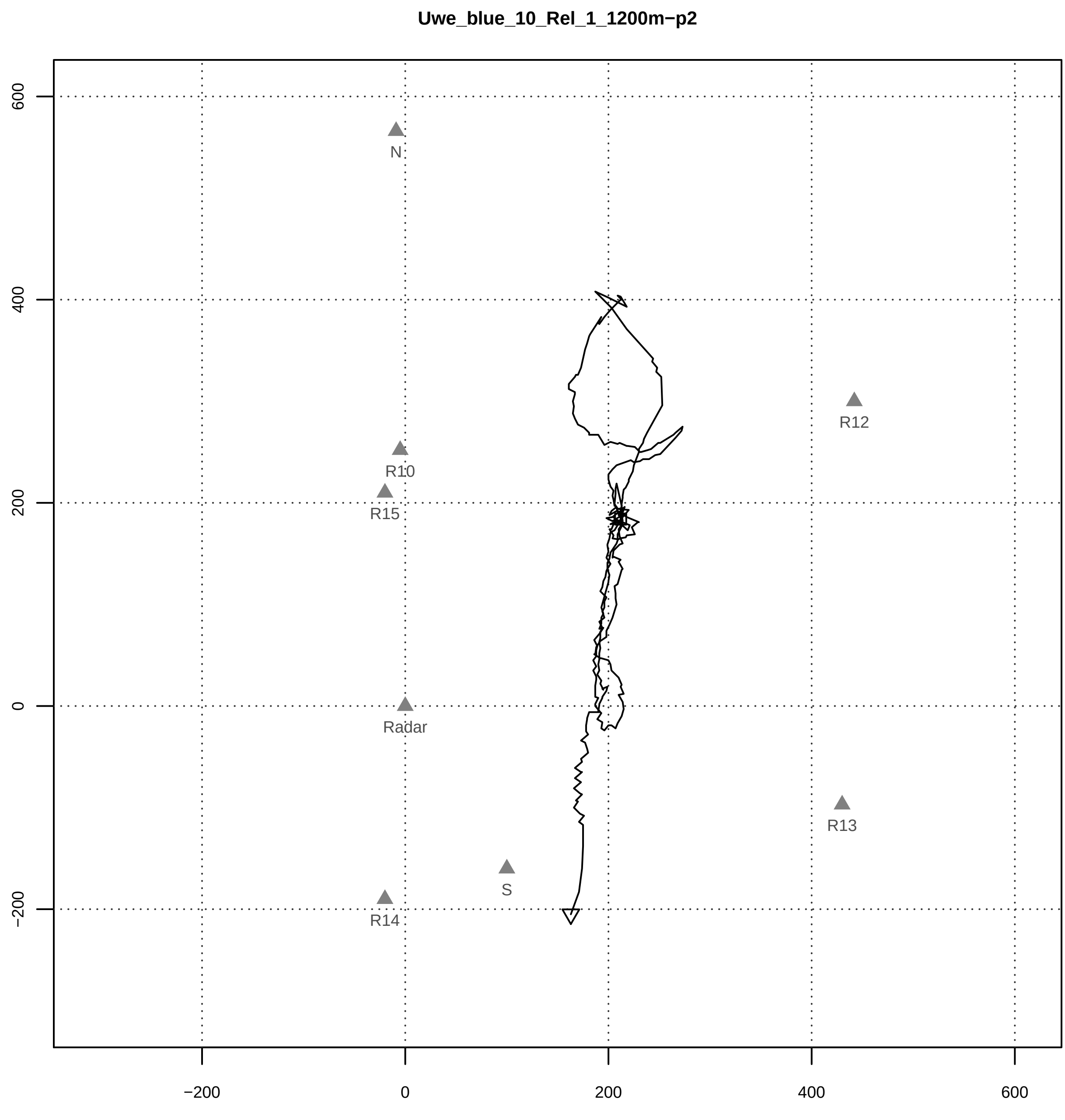

Uwe_blue_10_Rel_1_1200m−p2
600
400
200
0
−200
−200
0
200
400
600
N
R10
R15
Radar
R12
R13
S
R14

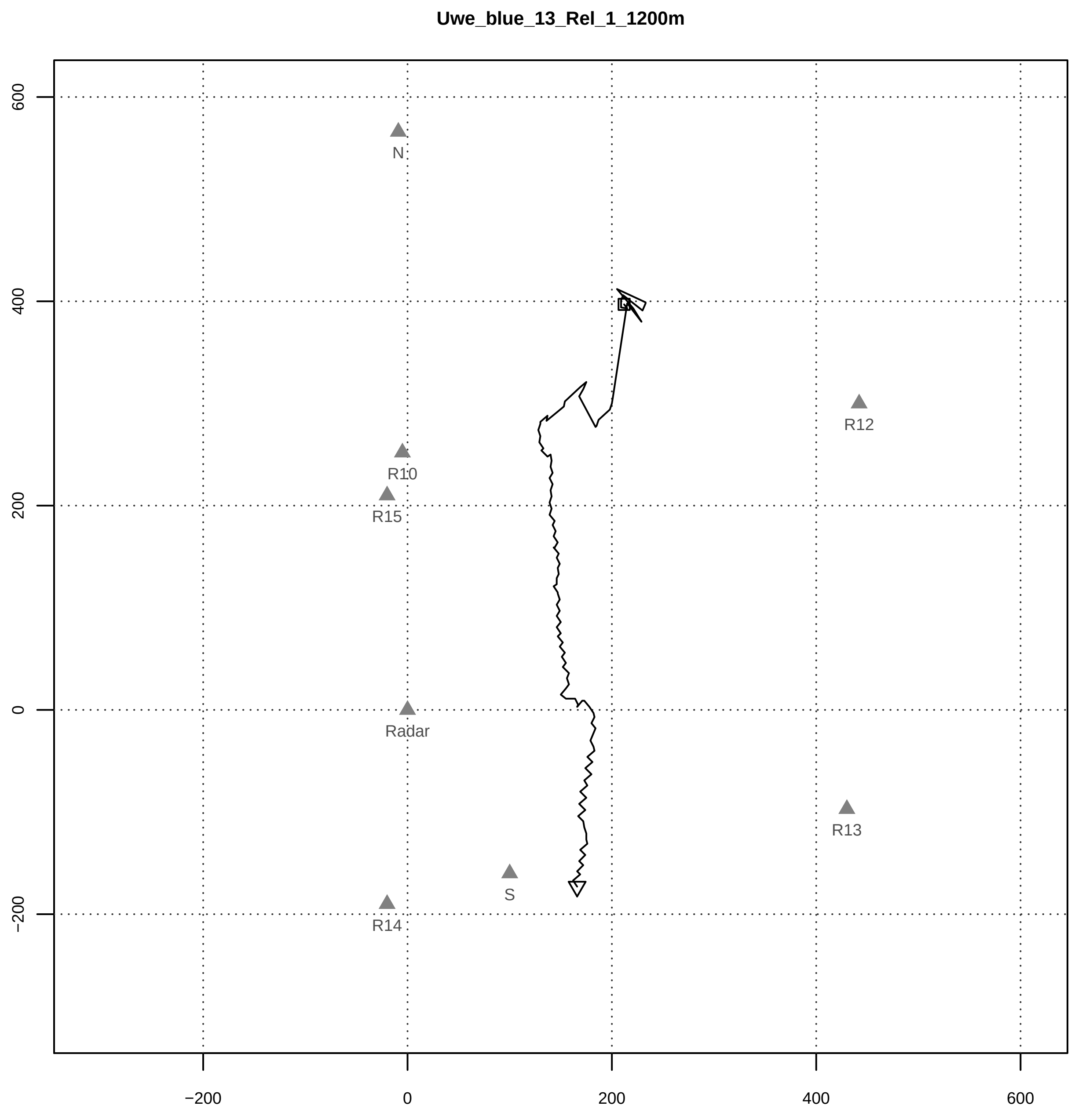
Uwe_blue_13_Rel_1_1200m
N
R10
R15
R12
Radar
R13
S
R14
−200
0
200
400
600
−200
0
200
400
600

Uwe_blue_13_Rel_1_1700m_400m_West

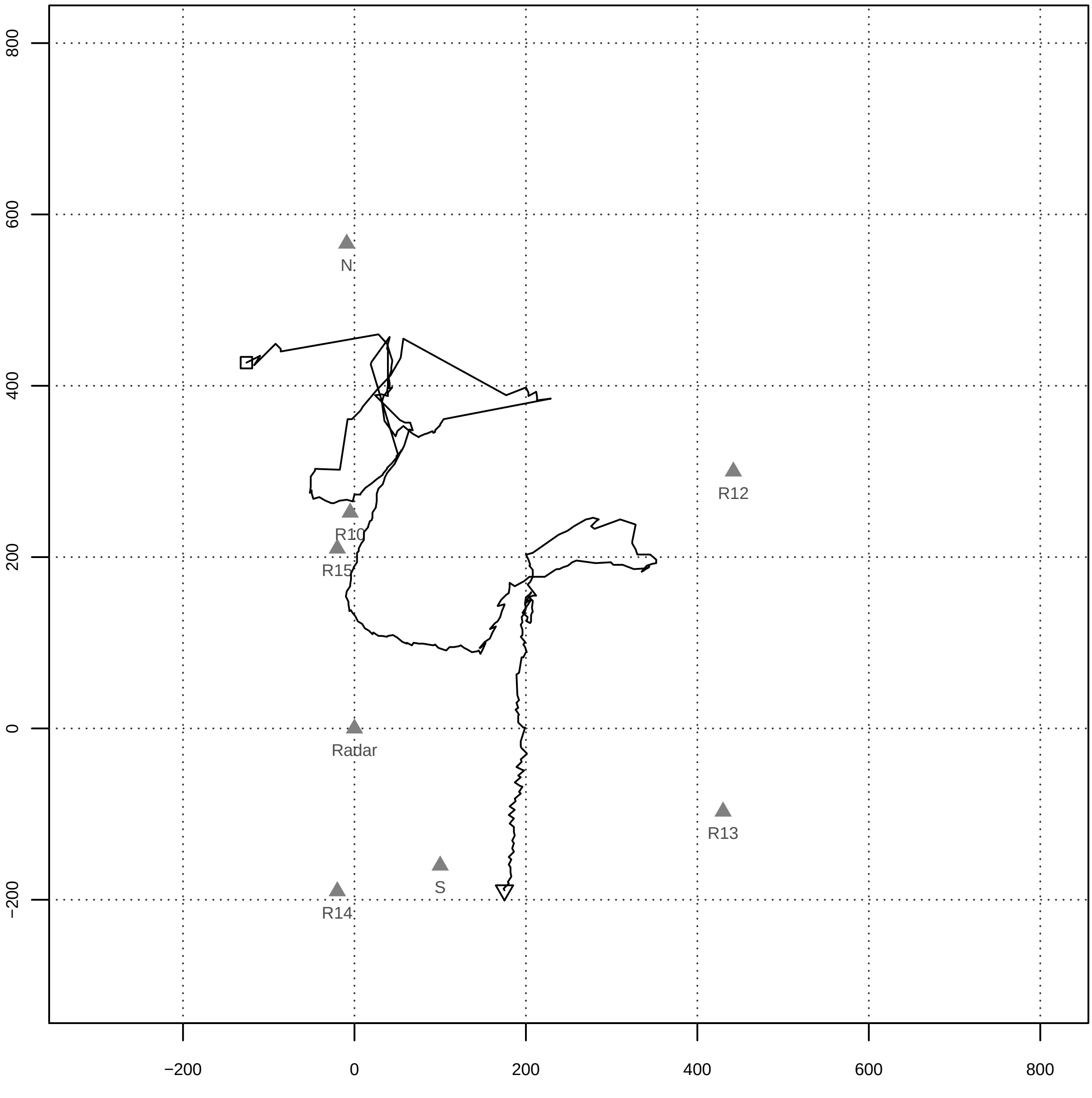

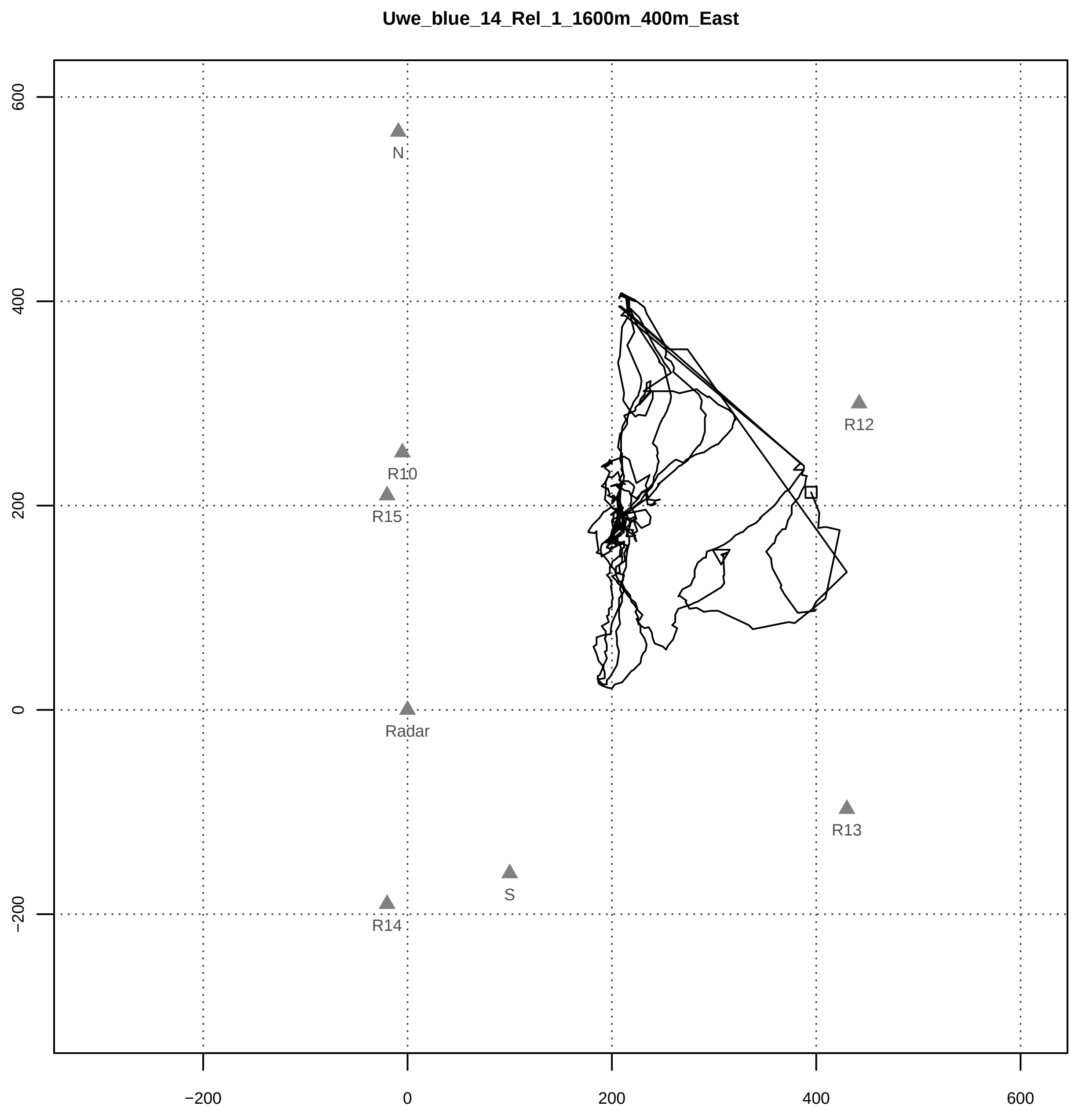

Uwe_blue_14_Rel_1_1600m_400m_East
N
R10
R15
R12
Radar
R13
S
R14
−200
0
200
400
600

Uwe_neon_blue_78_Rel_1_400m

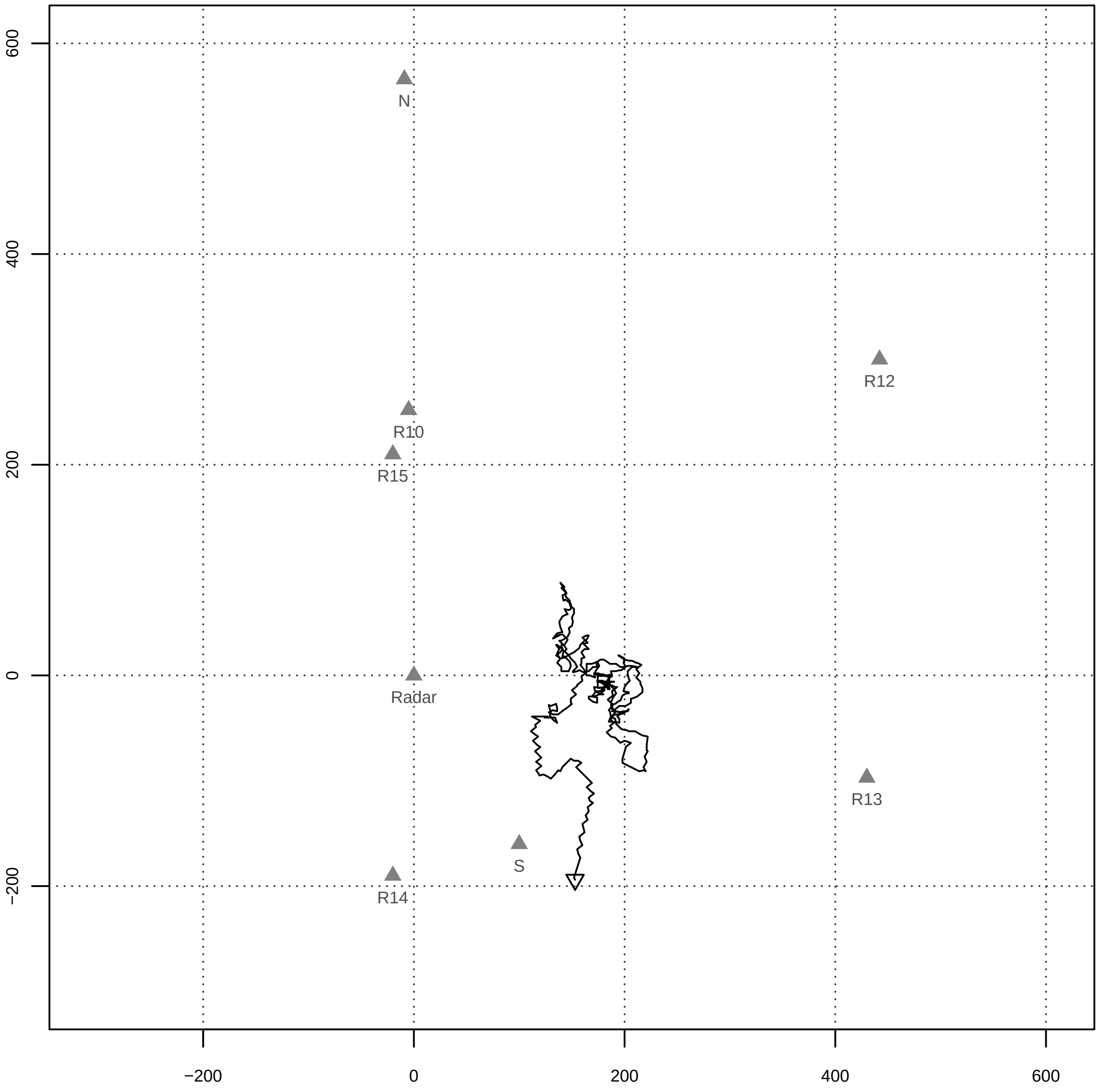

Uwe_neon_blue_78_Rel_1_800m

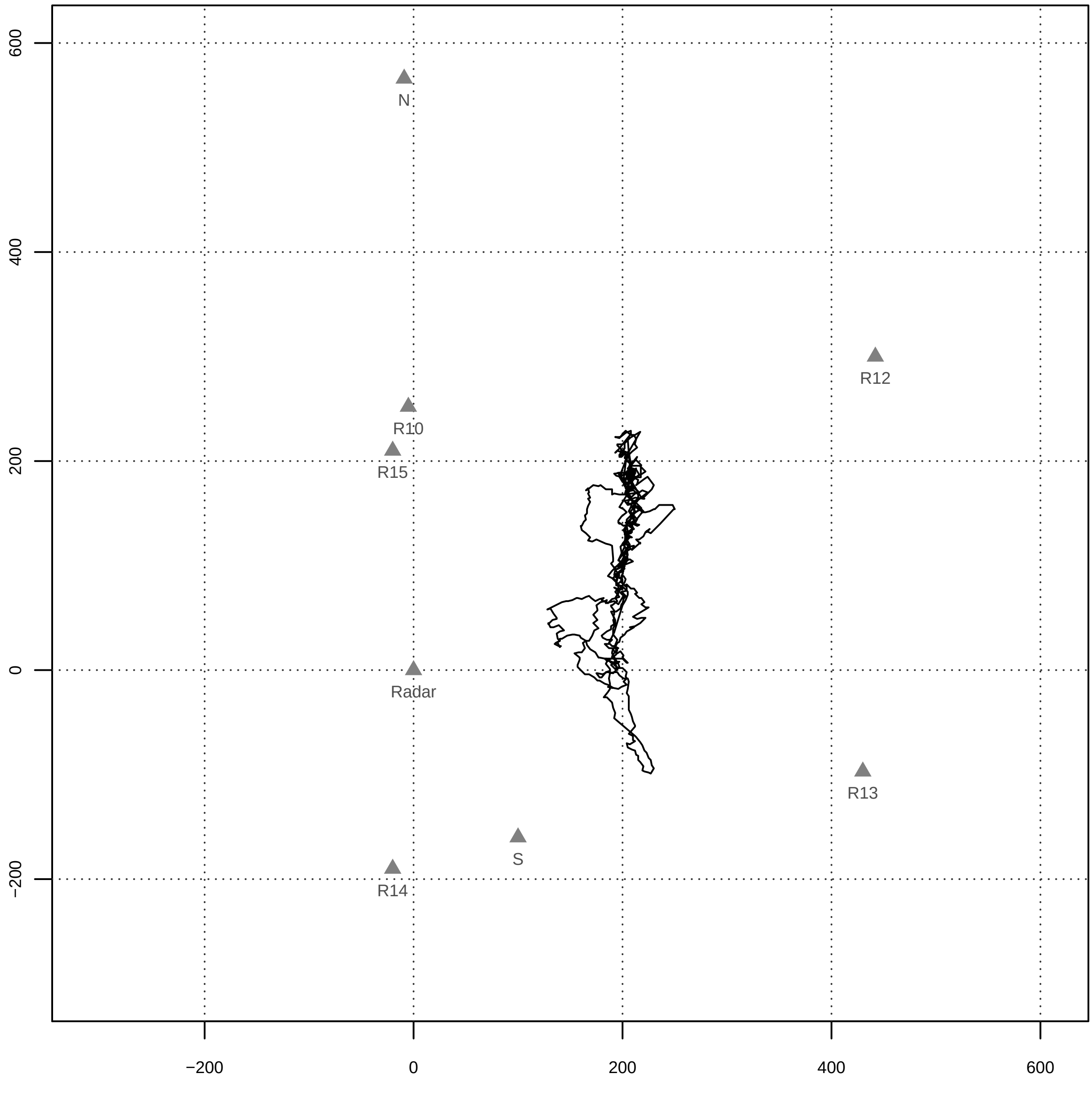

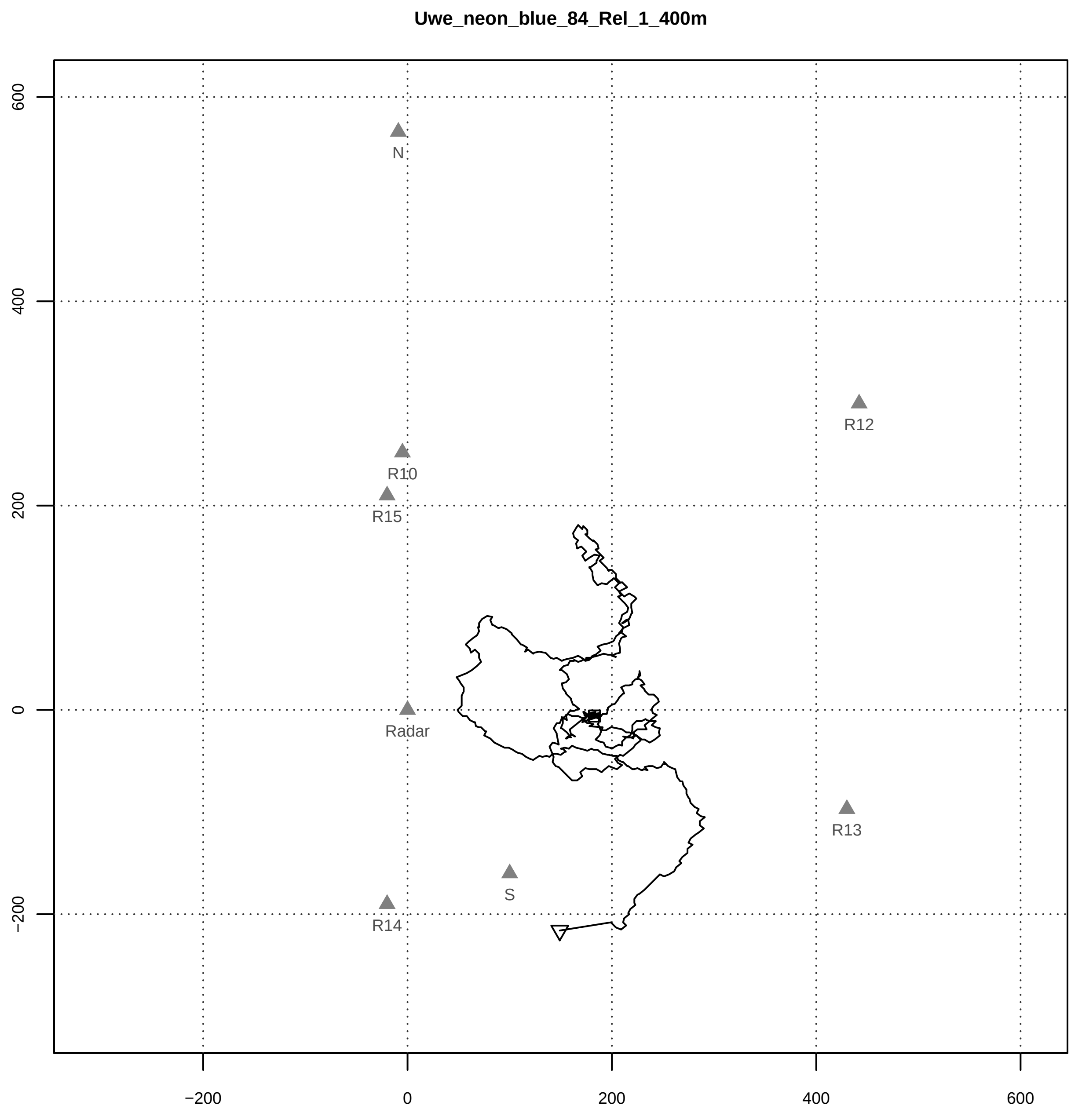

Uwe_neon_blue_84_Rel_1_400m
N
R12
R10
R15
Radar
R13
S
R14
−200
0
200
400
600

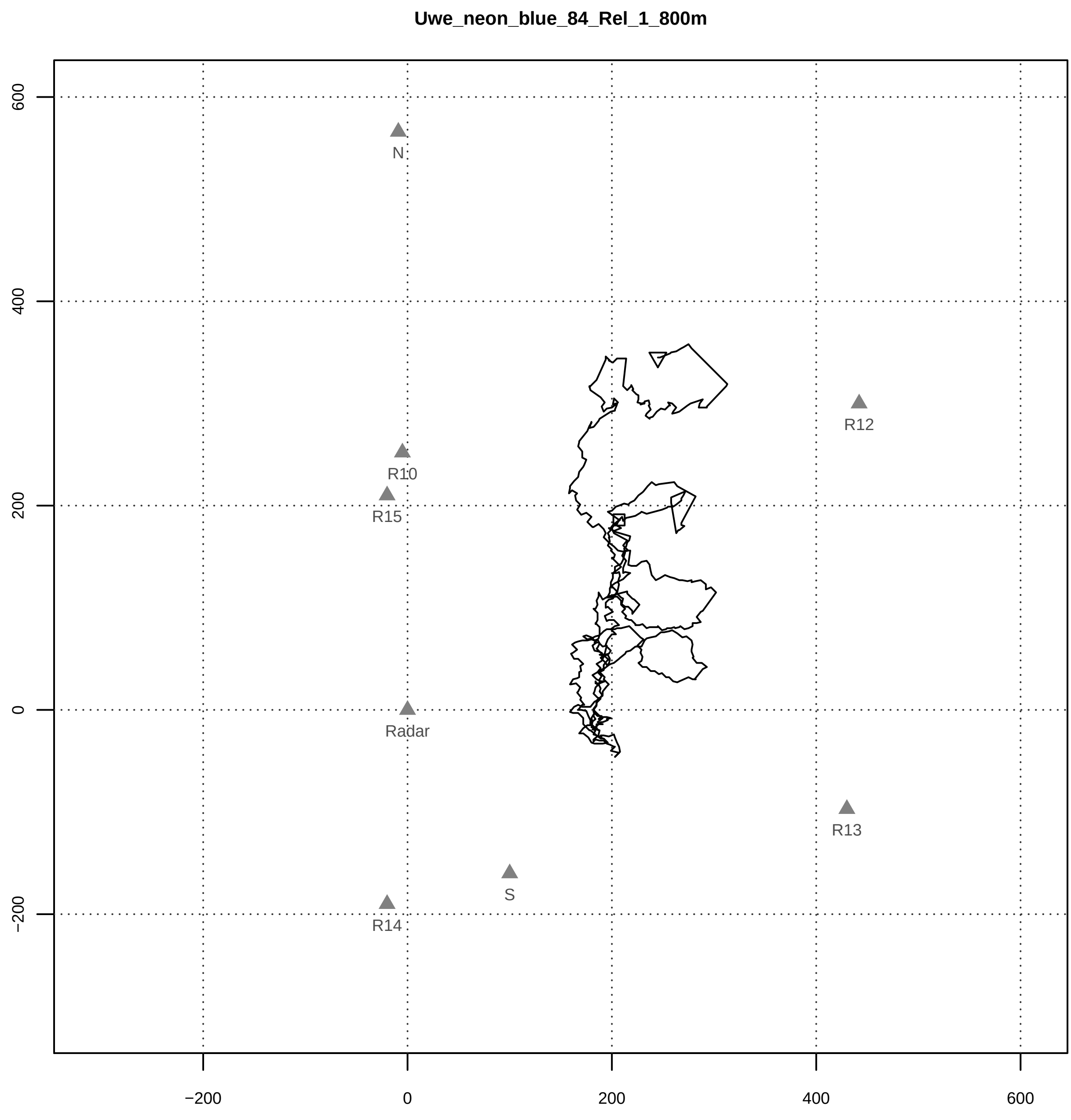

Uwe_neon_blue_84_Rel_1_800m
600
400
200
0
−200
N
R10
R15
R12
Radar
R13
S
R14
−200
0
200
400
600

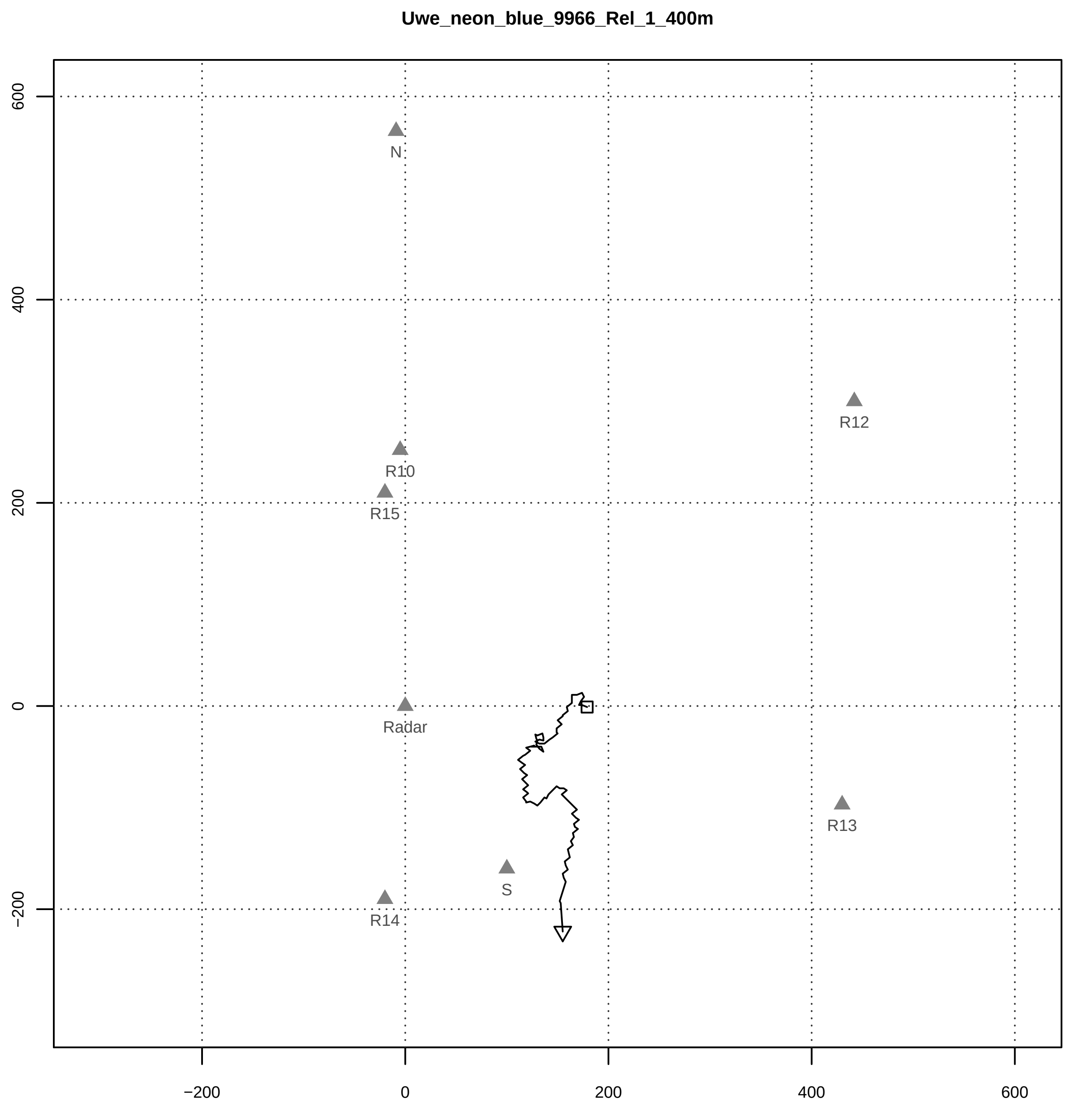

Uwe_neon_blue_9966_Rel_1_400m
N
R12
R10
R15
Radar
R13
S
R14
600
400
200
0
−200
−200
0
200
400
600

Uwe_neon_green_1_Rel_1_1200m

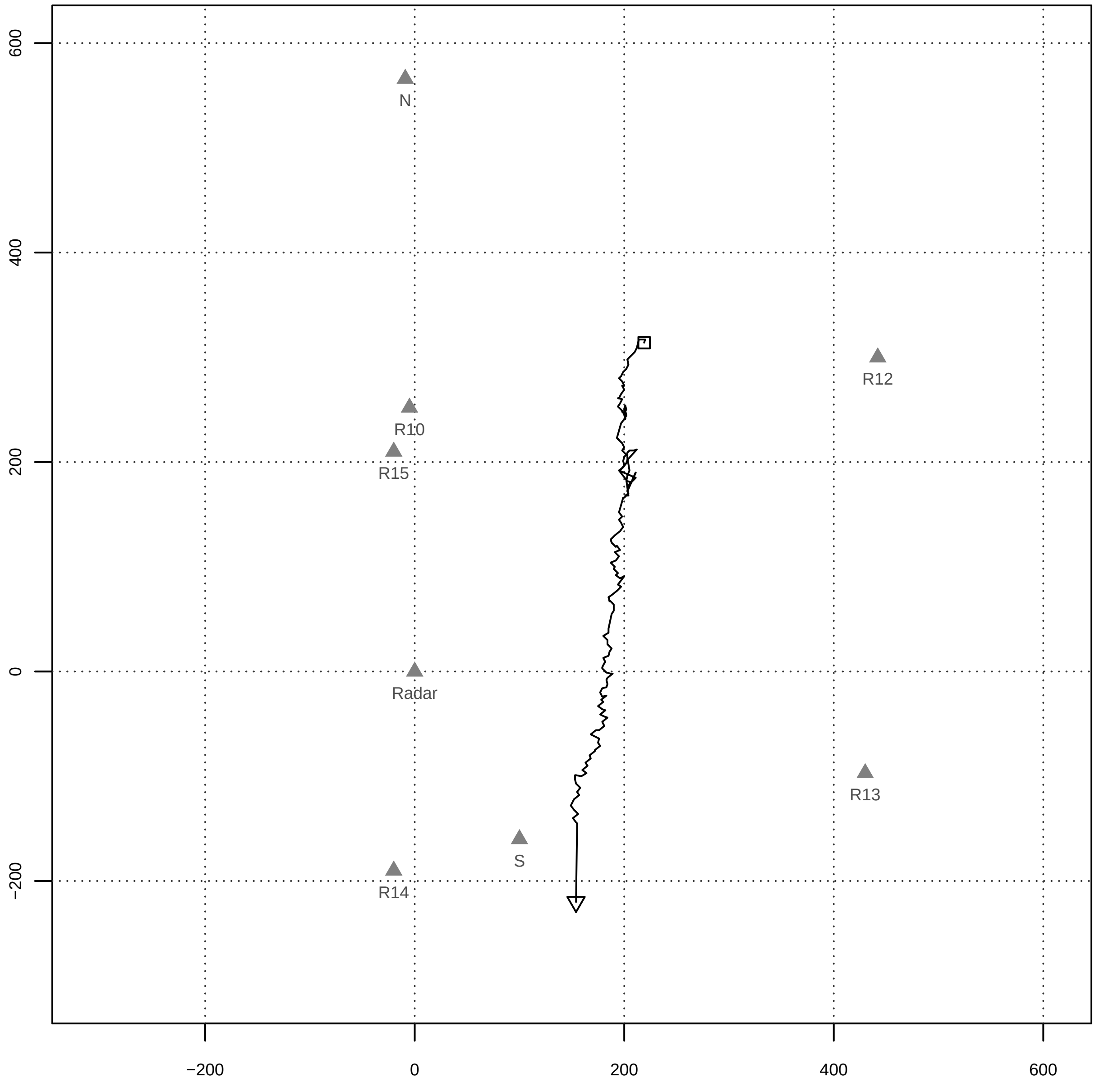

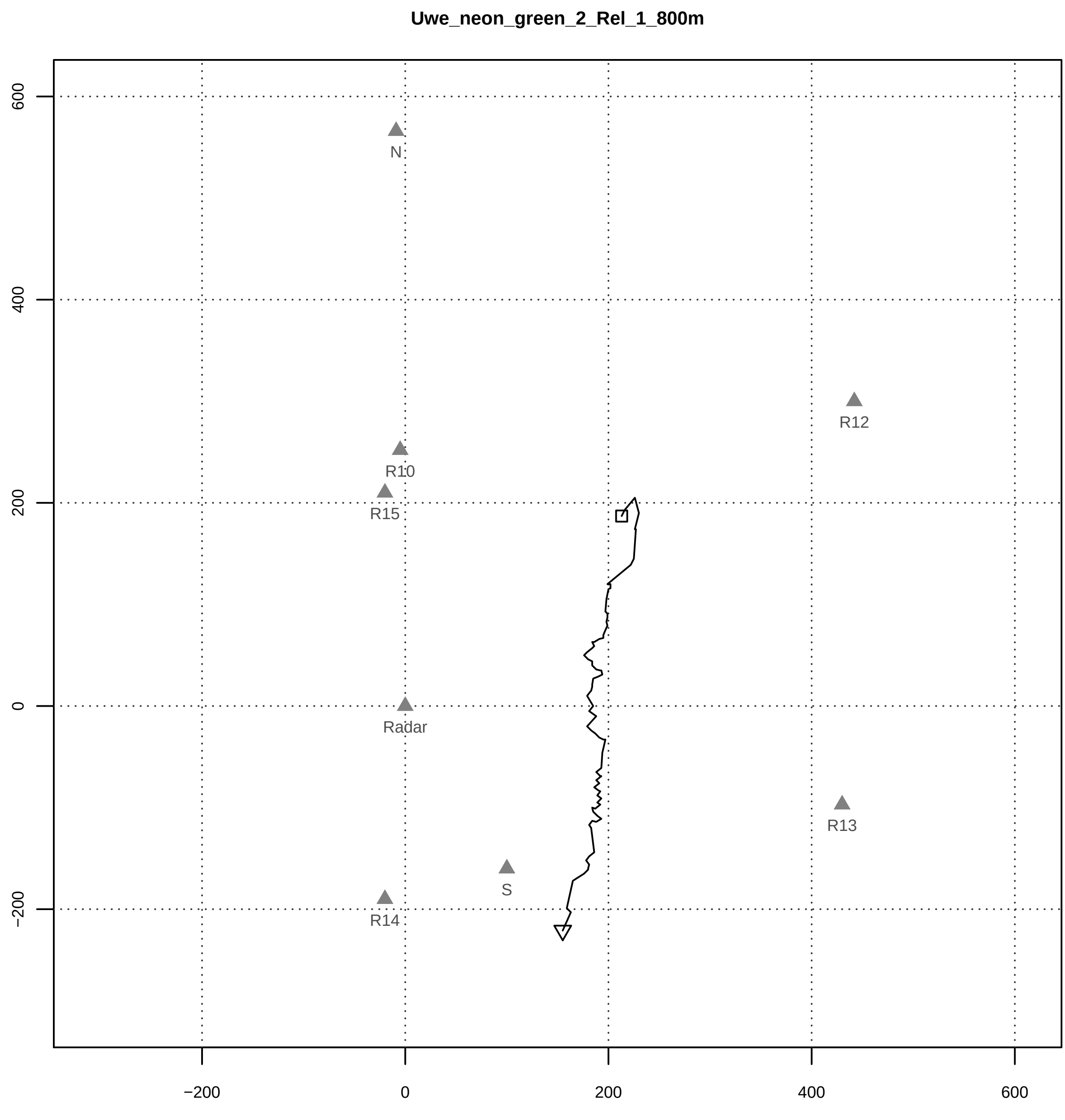
Uwe_neon_green_2_Rel_1_800m
N
R12
R10
R15
Radar
R13
S
R14
−200
0
200
400
600
−200
0
200
400
600

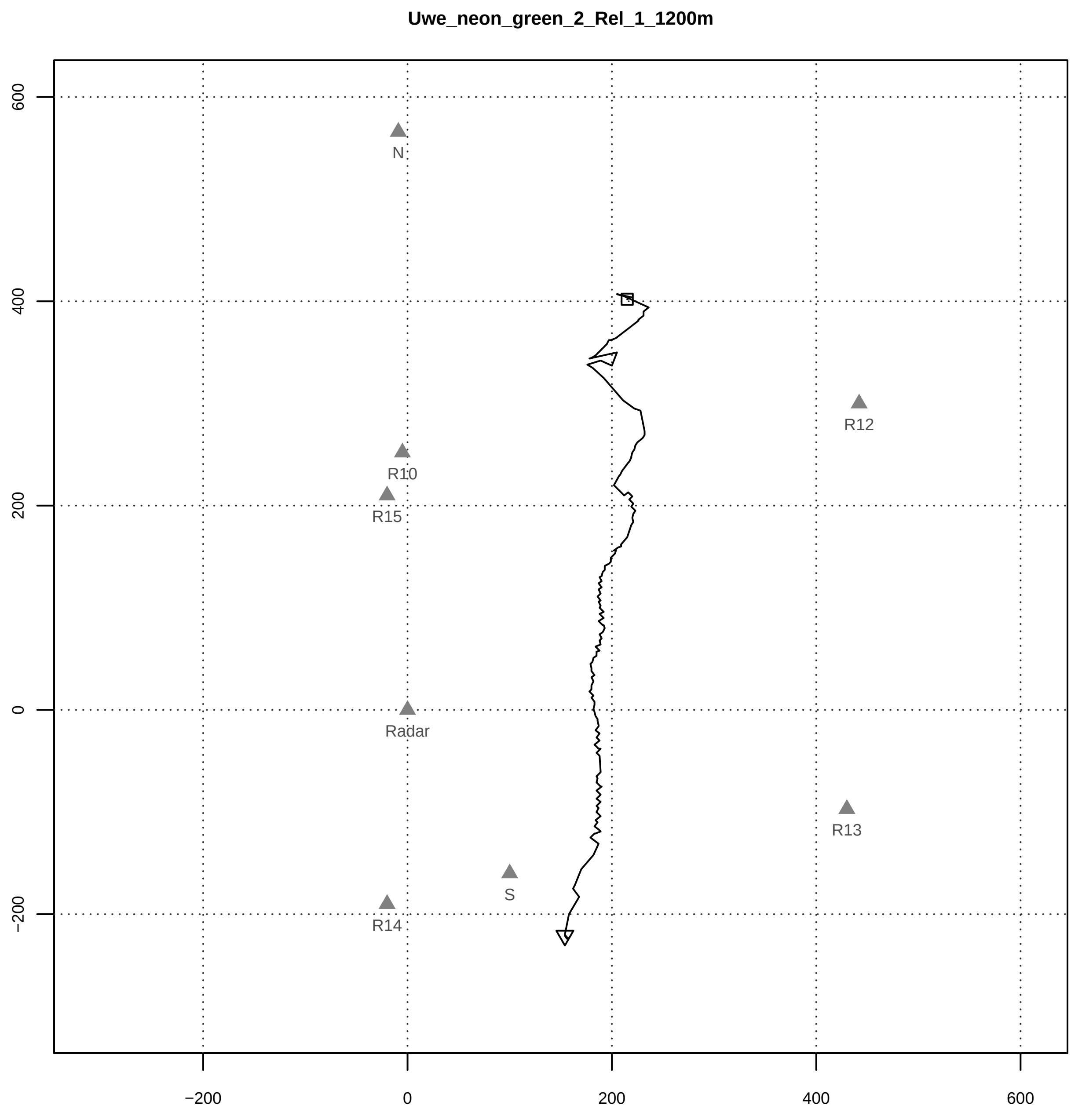
Uwe_neon_green_2_Rel_1_1200m
600
400
200
0
−200
−200
0
200
400
600
N
R10
R15
Radar
S
R14
R12
R13

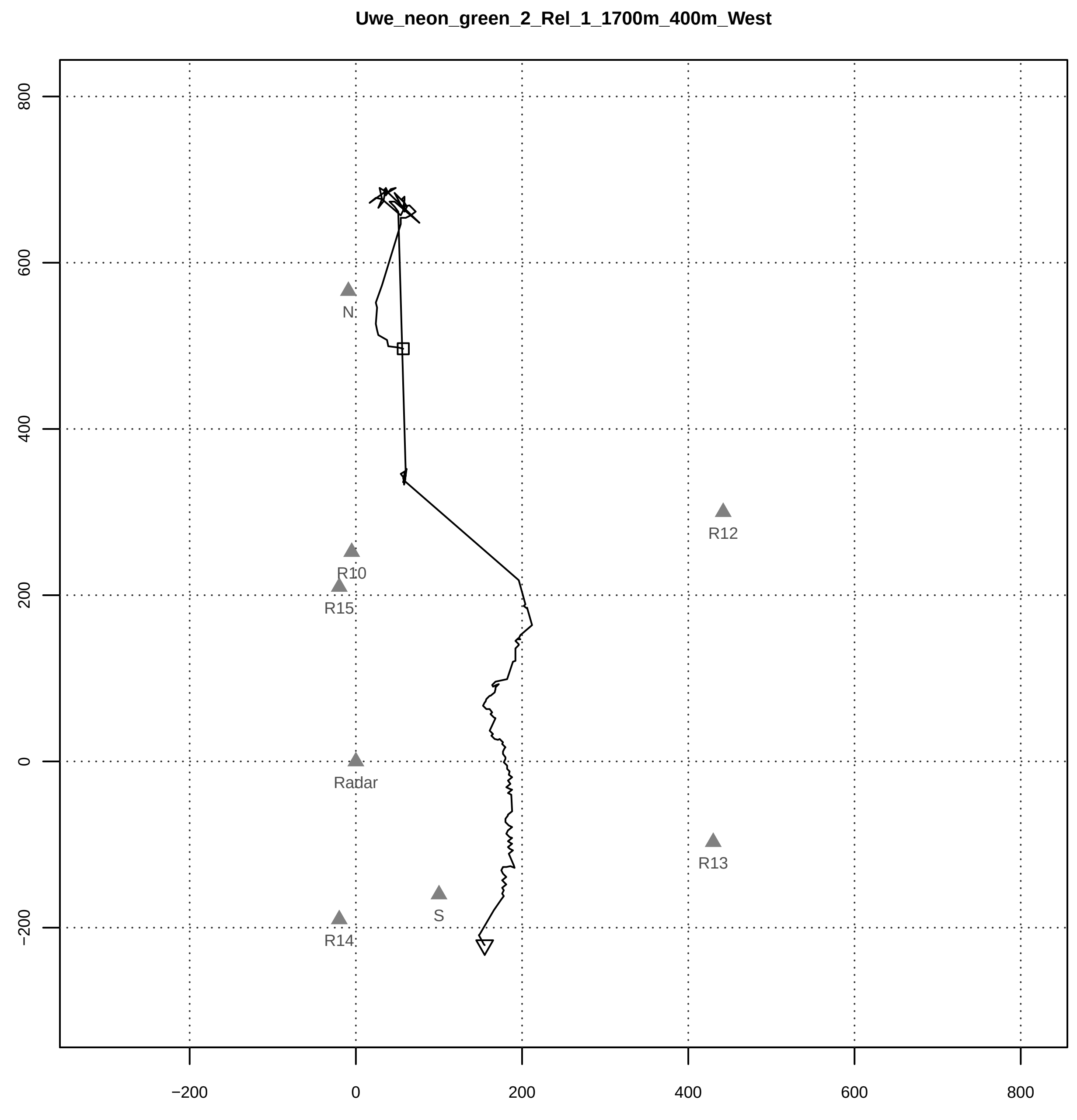

Uwe_neon_green_2_Rel_1_1700m_400m_West
N
R10
R15
R12
Radar
R13
S
R14
800
600
400
200
0
−200
−200
0
200
400
600
800

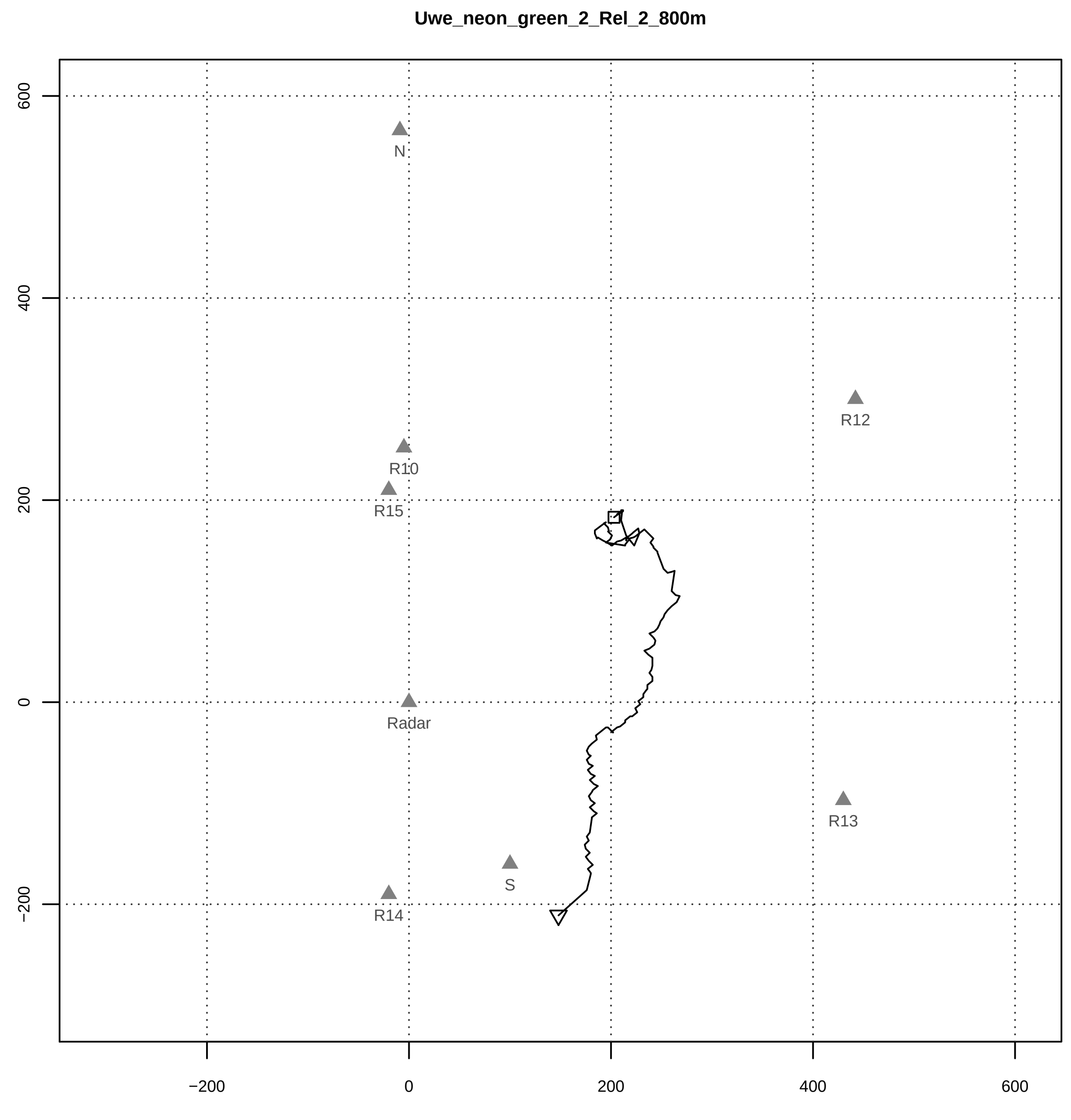

Uwe_neon_green_2_Rel_2_800m
N
R12
R10
R15
Radar
R13
S
R14
−200
0
200
400
600

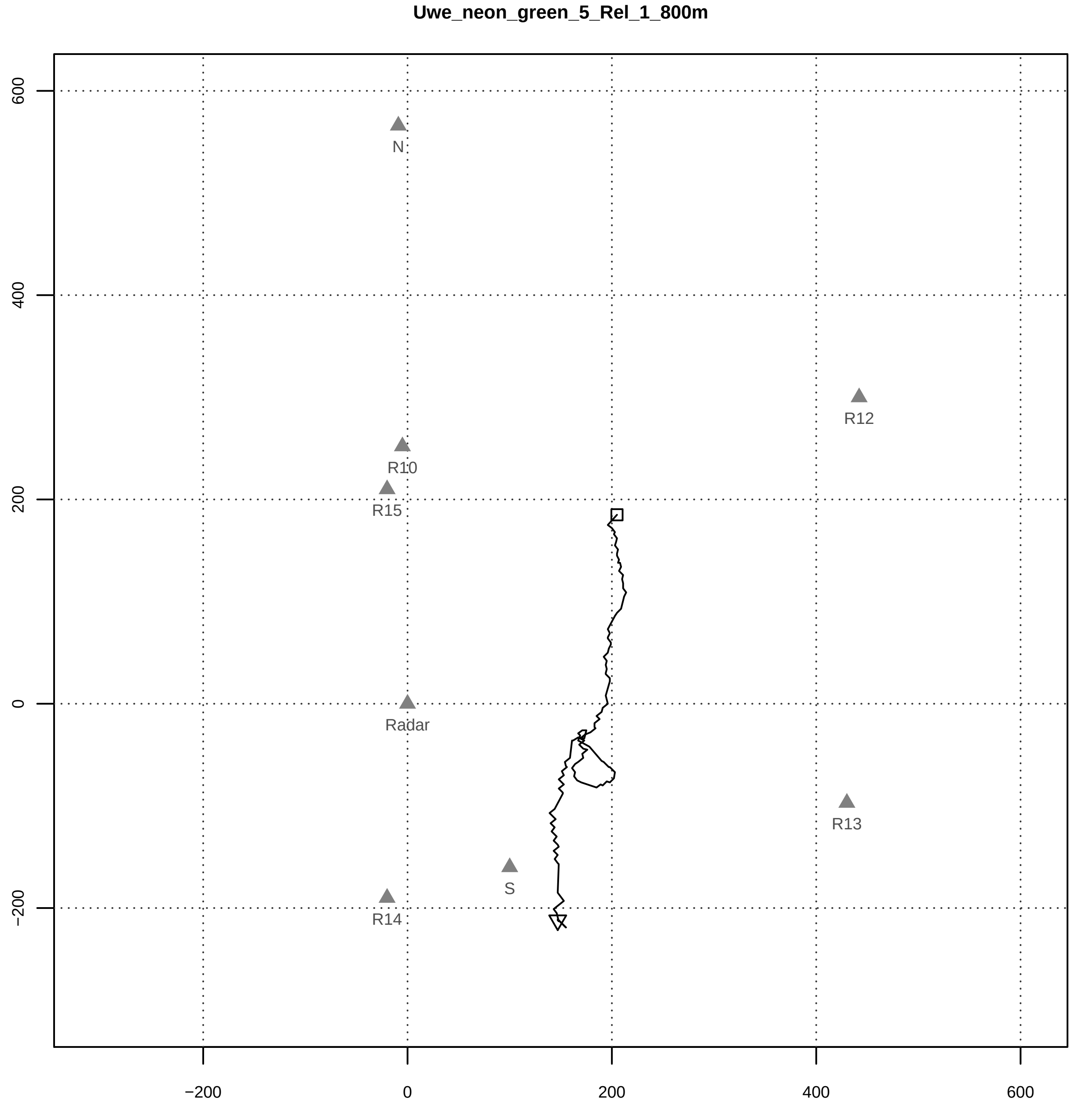

Uwe_neon_green_5_Rel_1_800m
N
R10
R15
R12
Radar
R13
S
R14
−200
0
200
400
600
−200
0
200
400
600

Uwe_neon_green_8_Rel_1_800m

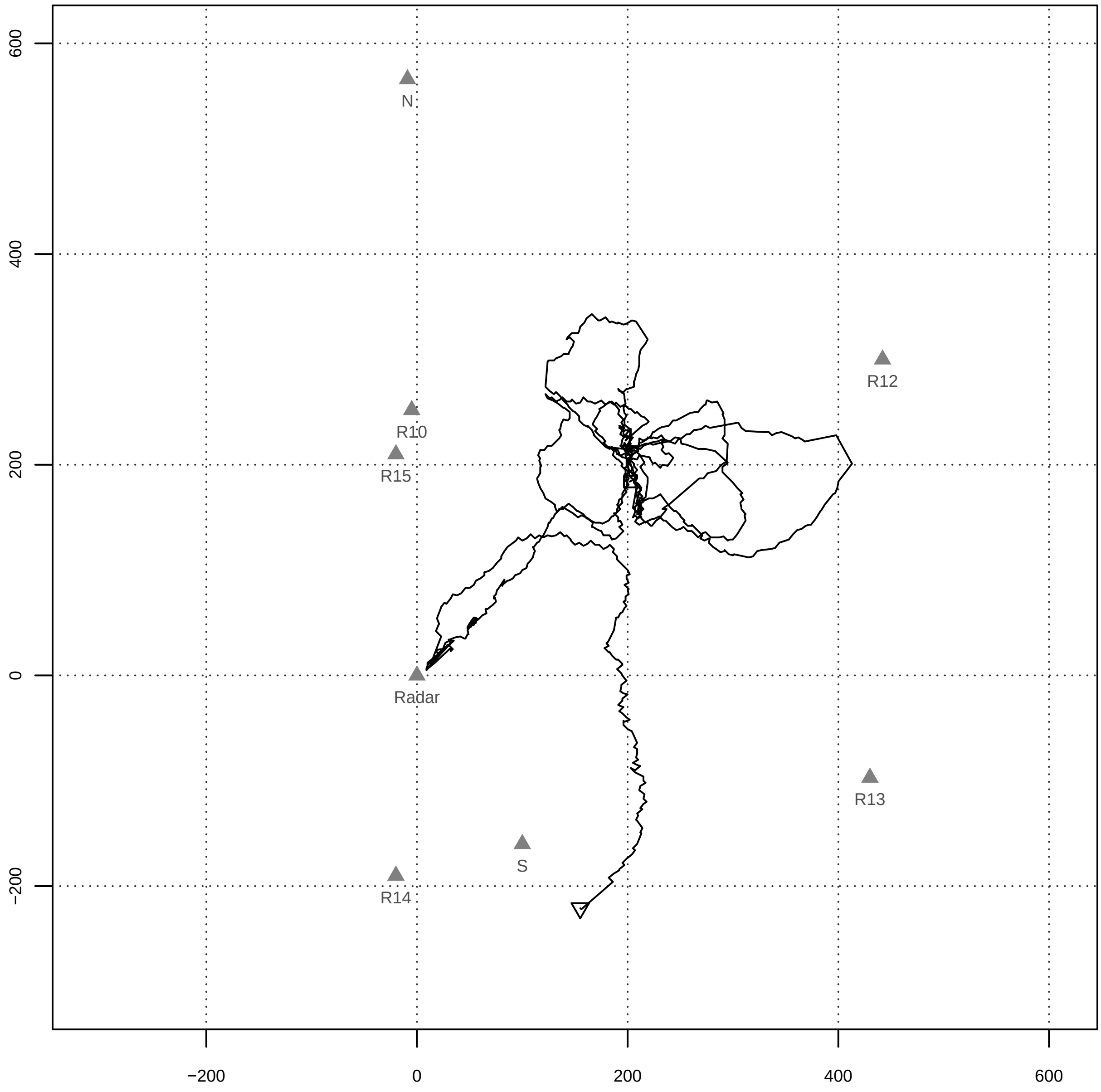

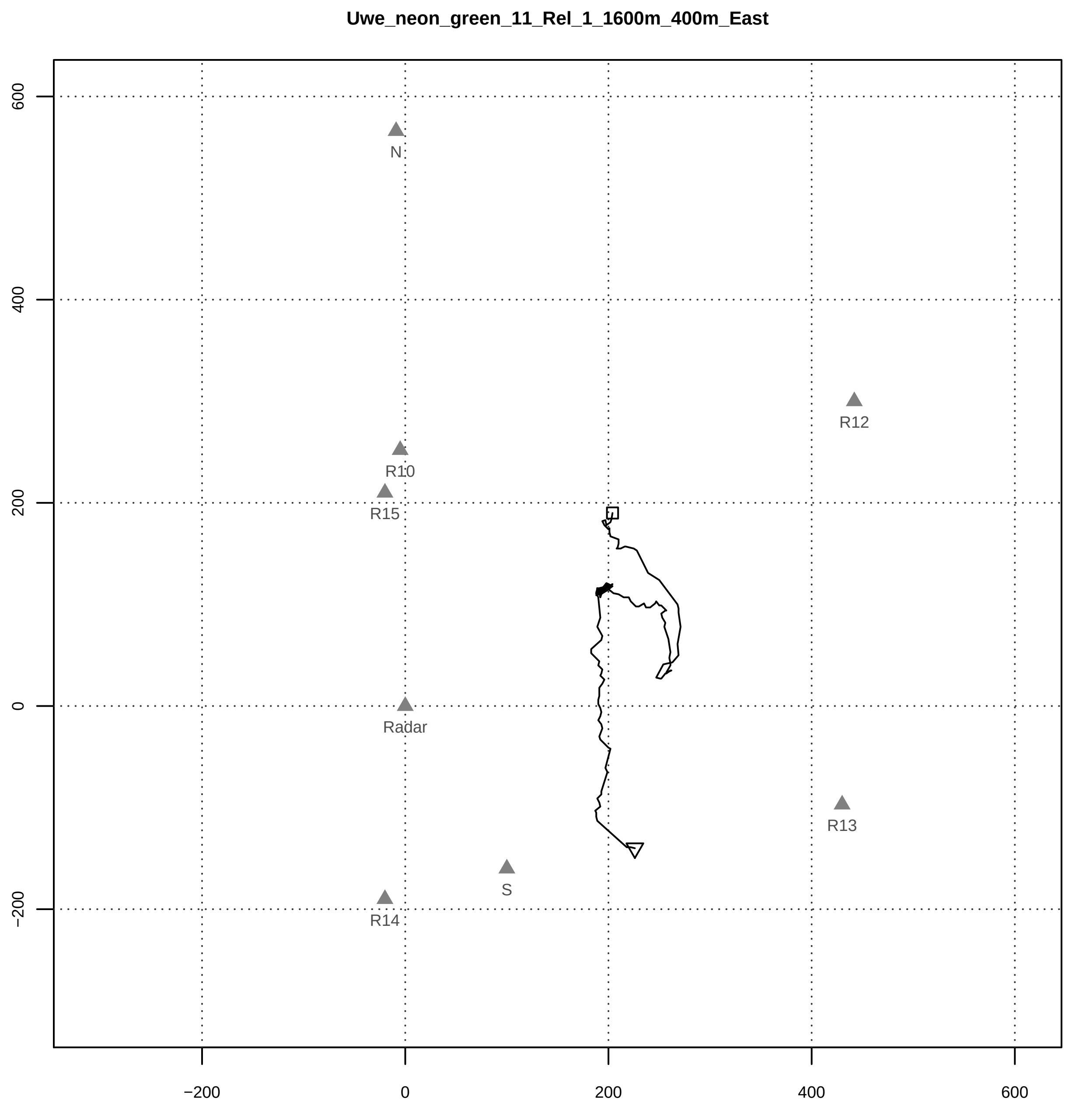

Uwe_neon_green_11_Rel_1_1600m_400m_East
N
R12
R10
R15
Radar
R13
S
R14
600
400
200
0
−200
−200
0
200
400
600

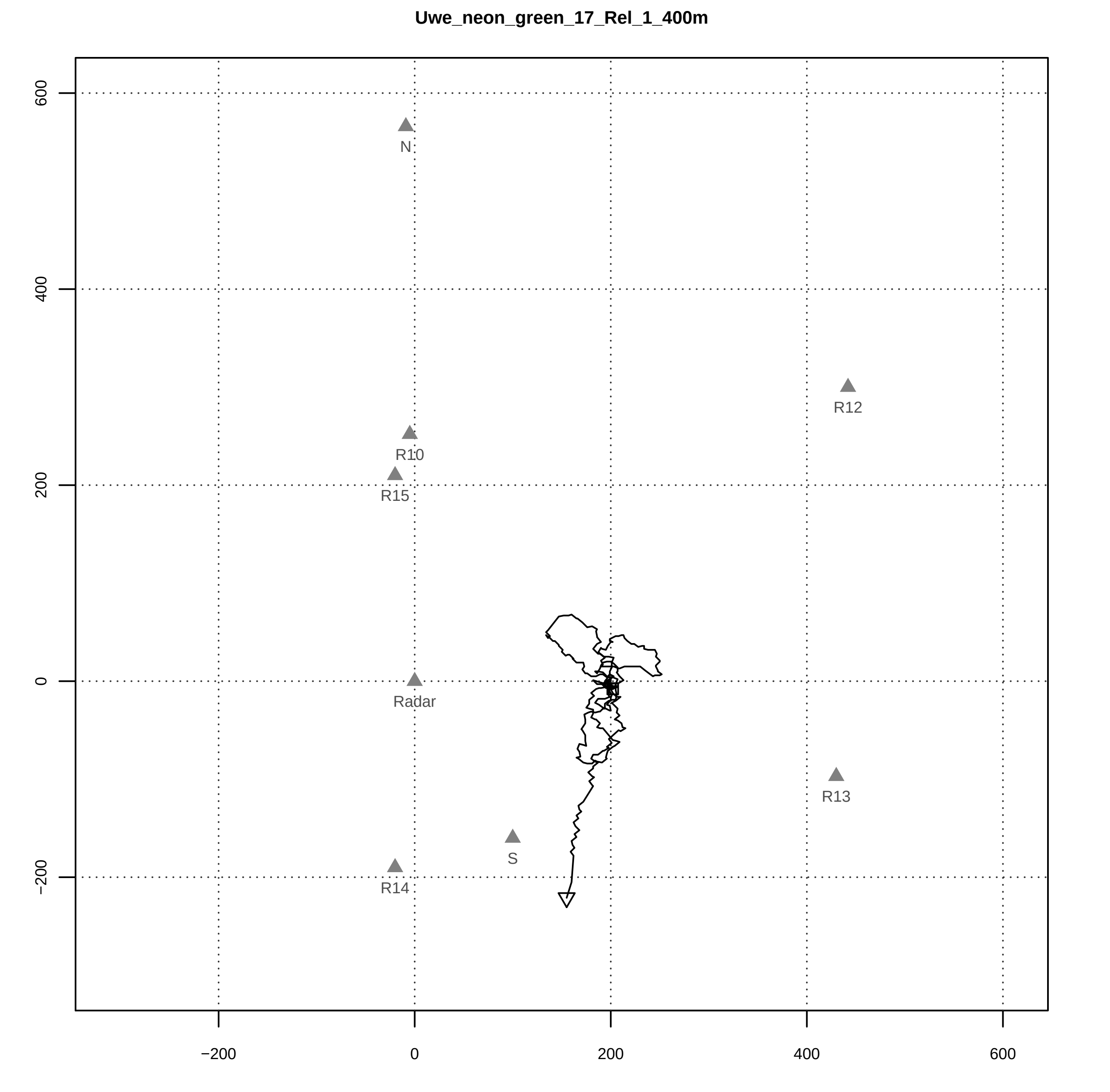
Uwe_neon_green_17_Rel_1_400m
600
400
200
0
−200
−200
0
200
400
600
N
R12
R10
R15
Radar
R13
S
R14

Uwe_neon_green_47_Rel_1_800m

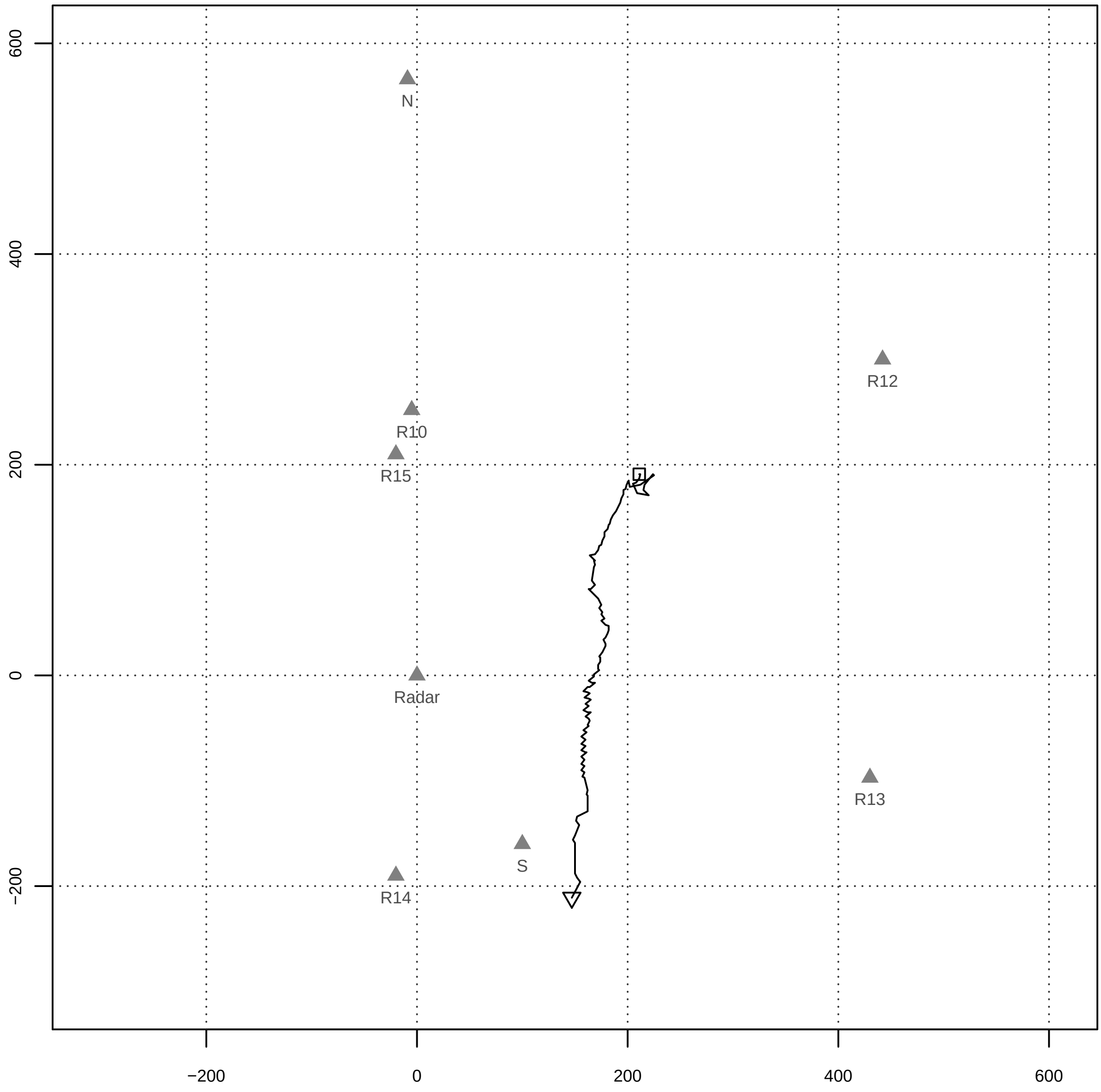

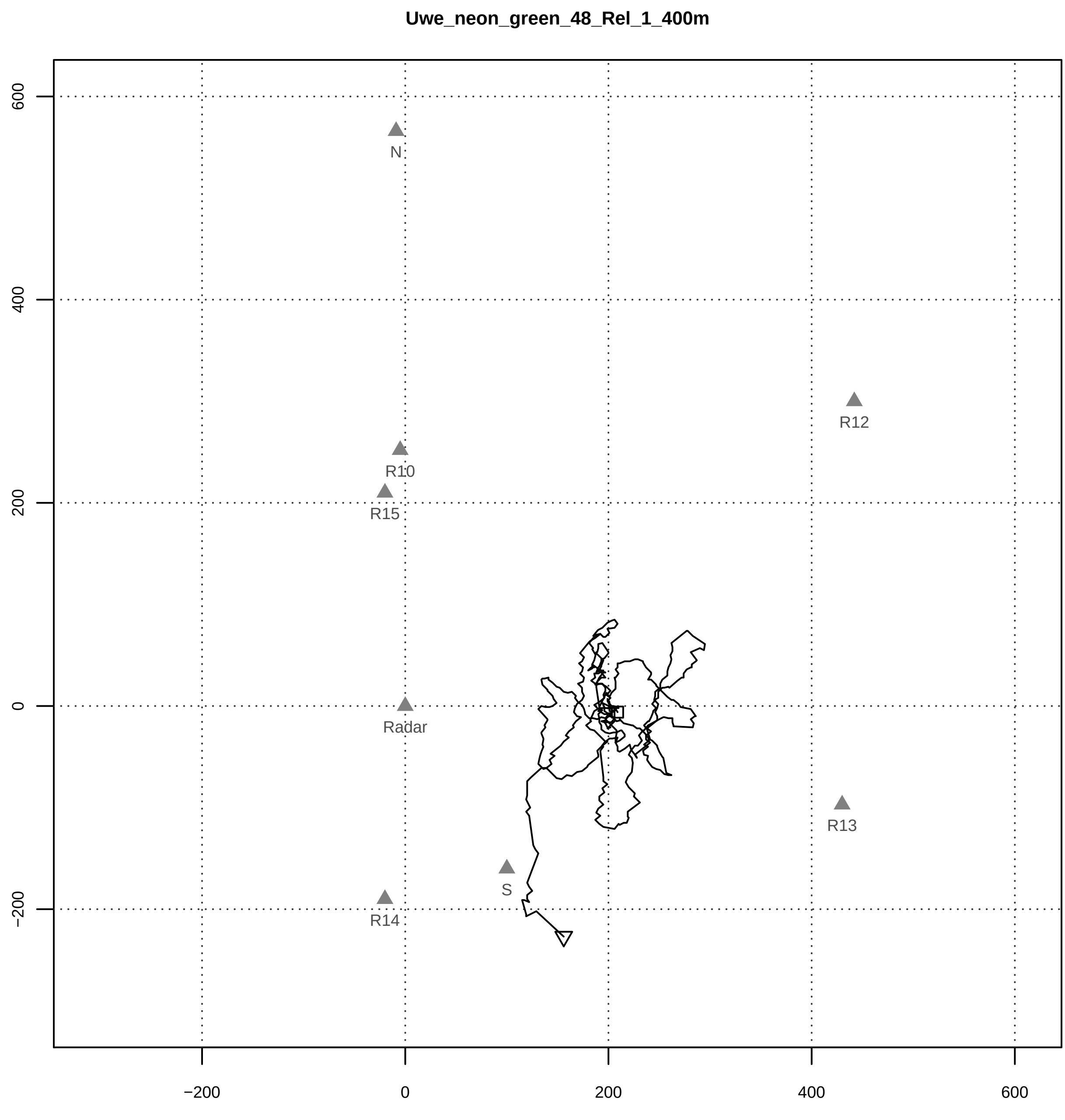
Uwe_neon_green_48_Rel_1_400m
N
R12
R10
R15
Radar
R13
S
R14
600
400
200
0
−200
−200
0
200
400
600

Uwe_neon_green_58_Rel_1_400m

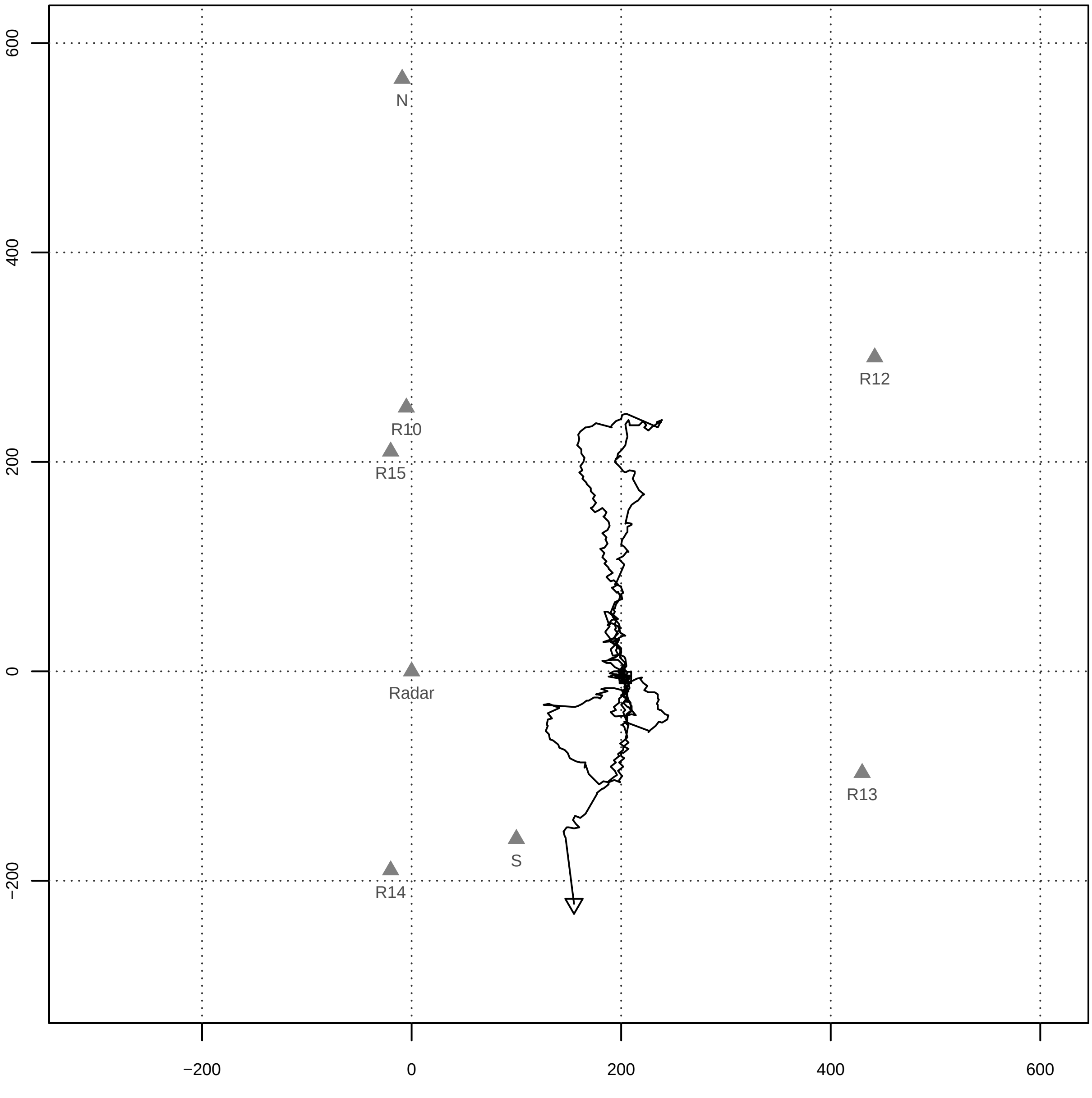

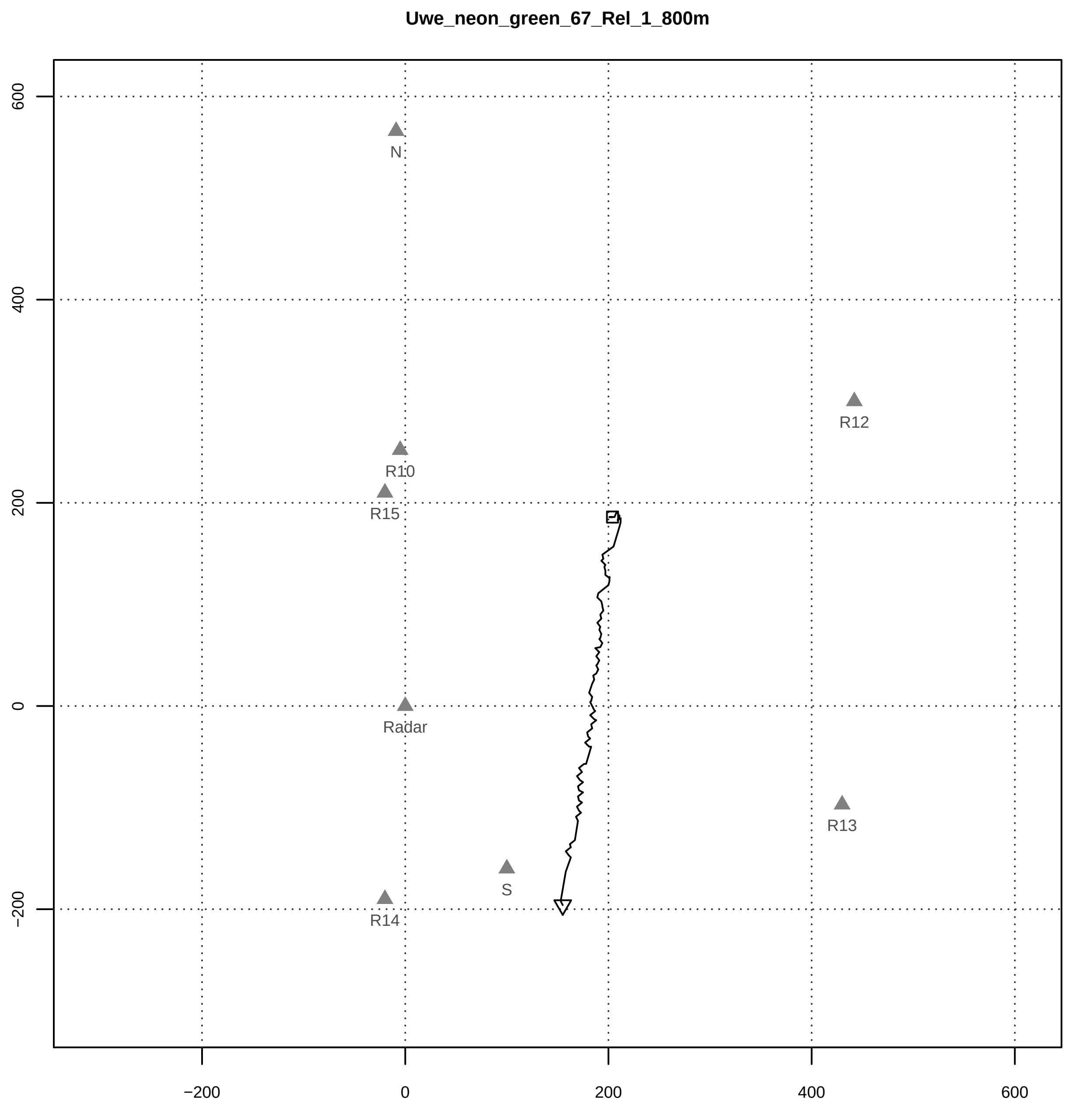
Uwe_neon_green_67_Rel_1_800m
N
R12
R10
R15
Radar
R13
S
R14
−200
0
200
400
600

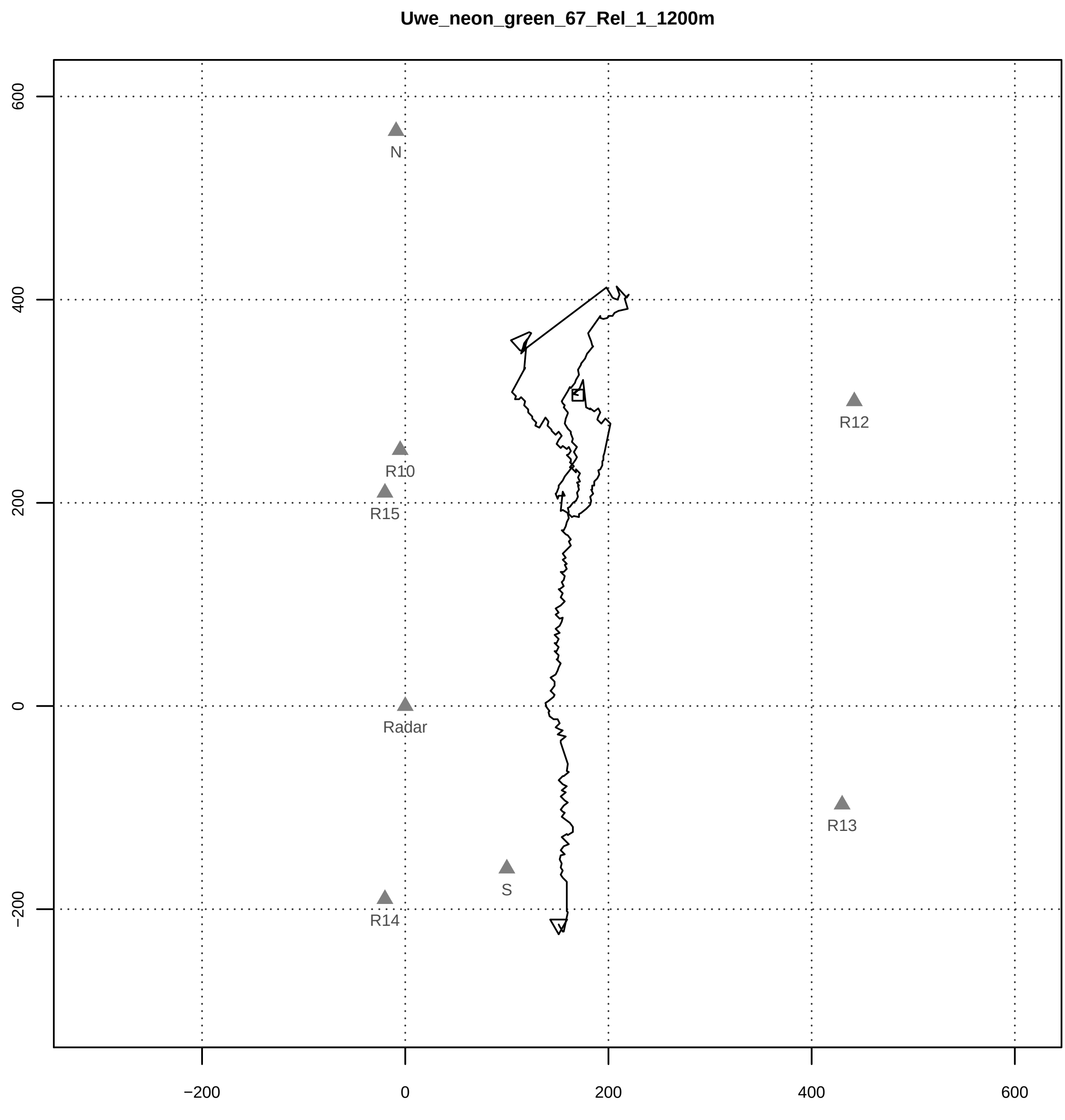

Uwe_neon_green_67_Rel_1_1200m
N
R10
R15
R12
Radar
R13
S
R14
−200
0
200
400
600

Uwe_neon_pink_95_Rel_1_800m

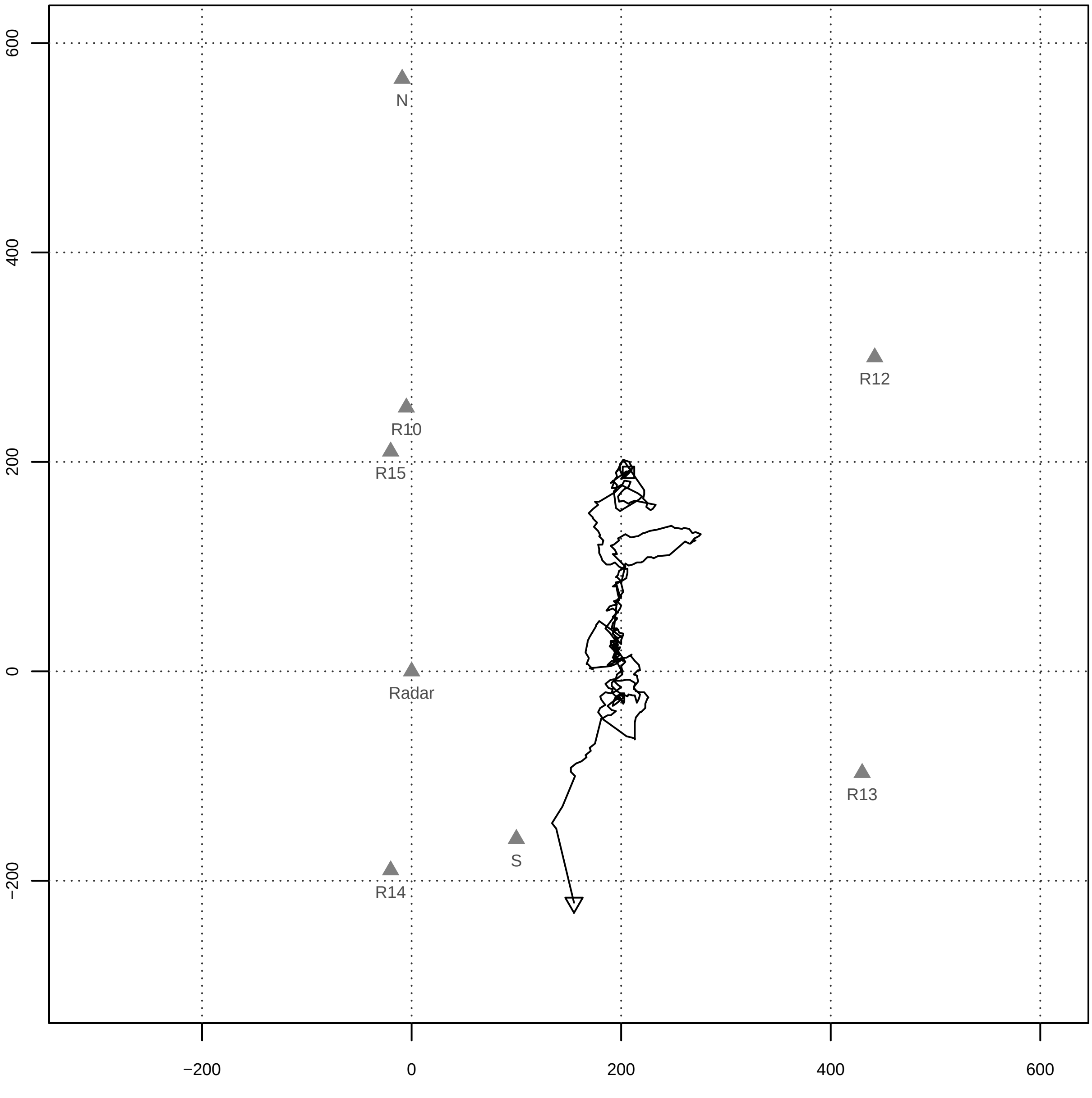

Uwe_neon_pink_96_Rel_2_400m

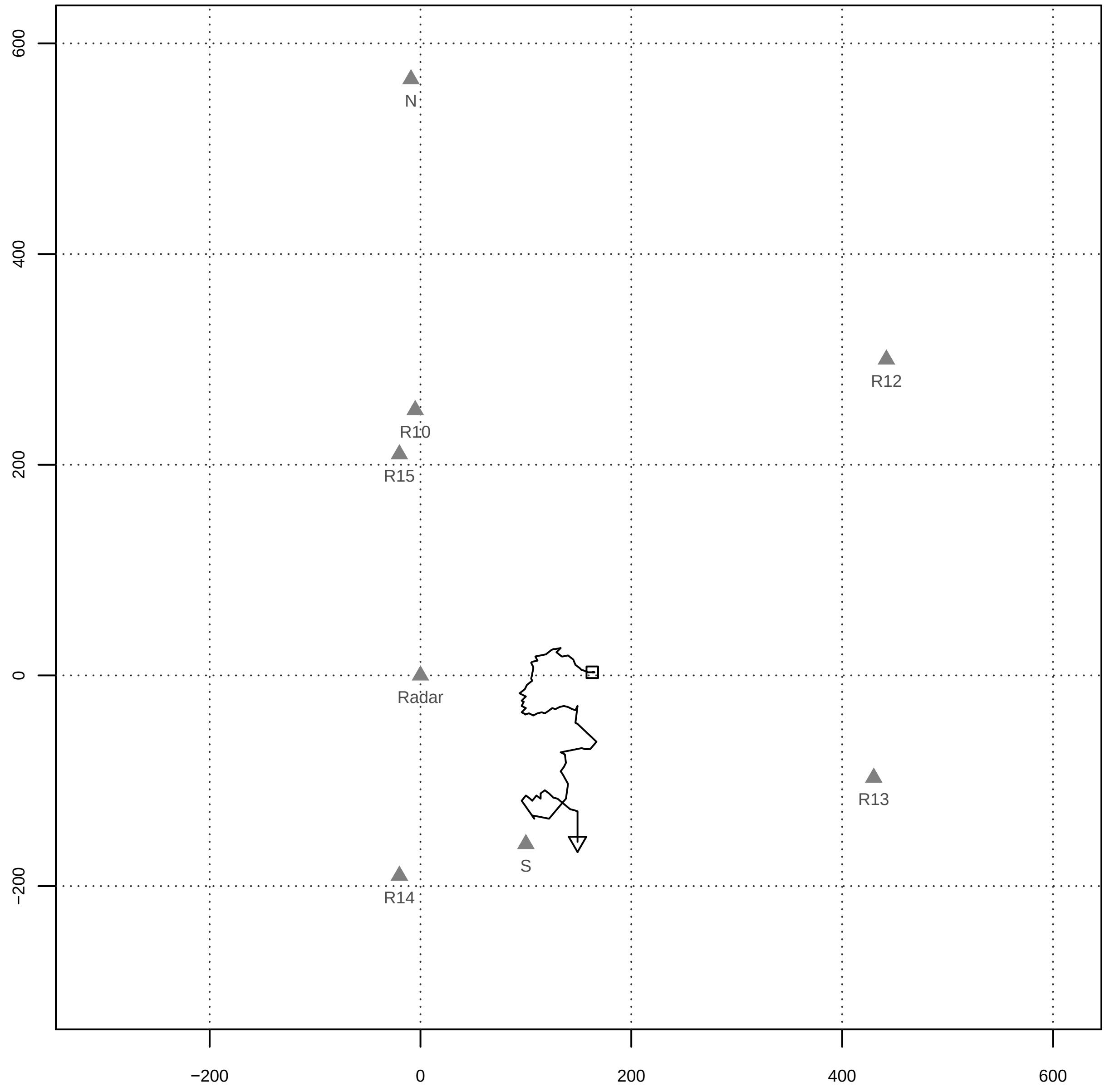

Uwe_neon_yellow_2_Rel_1_1600m_400m_East

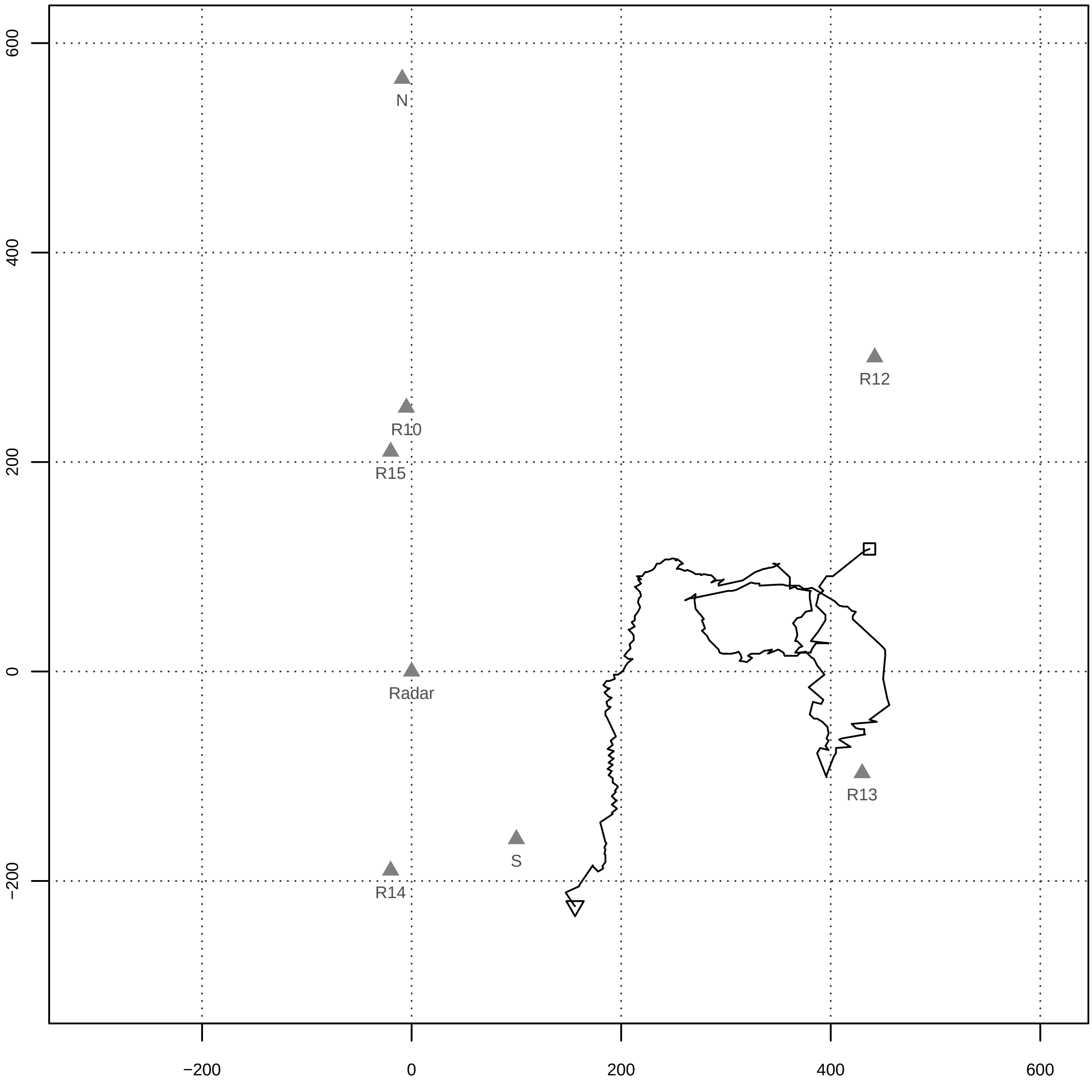

Uwe_neon_yellow_6_Rel_1_400m

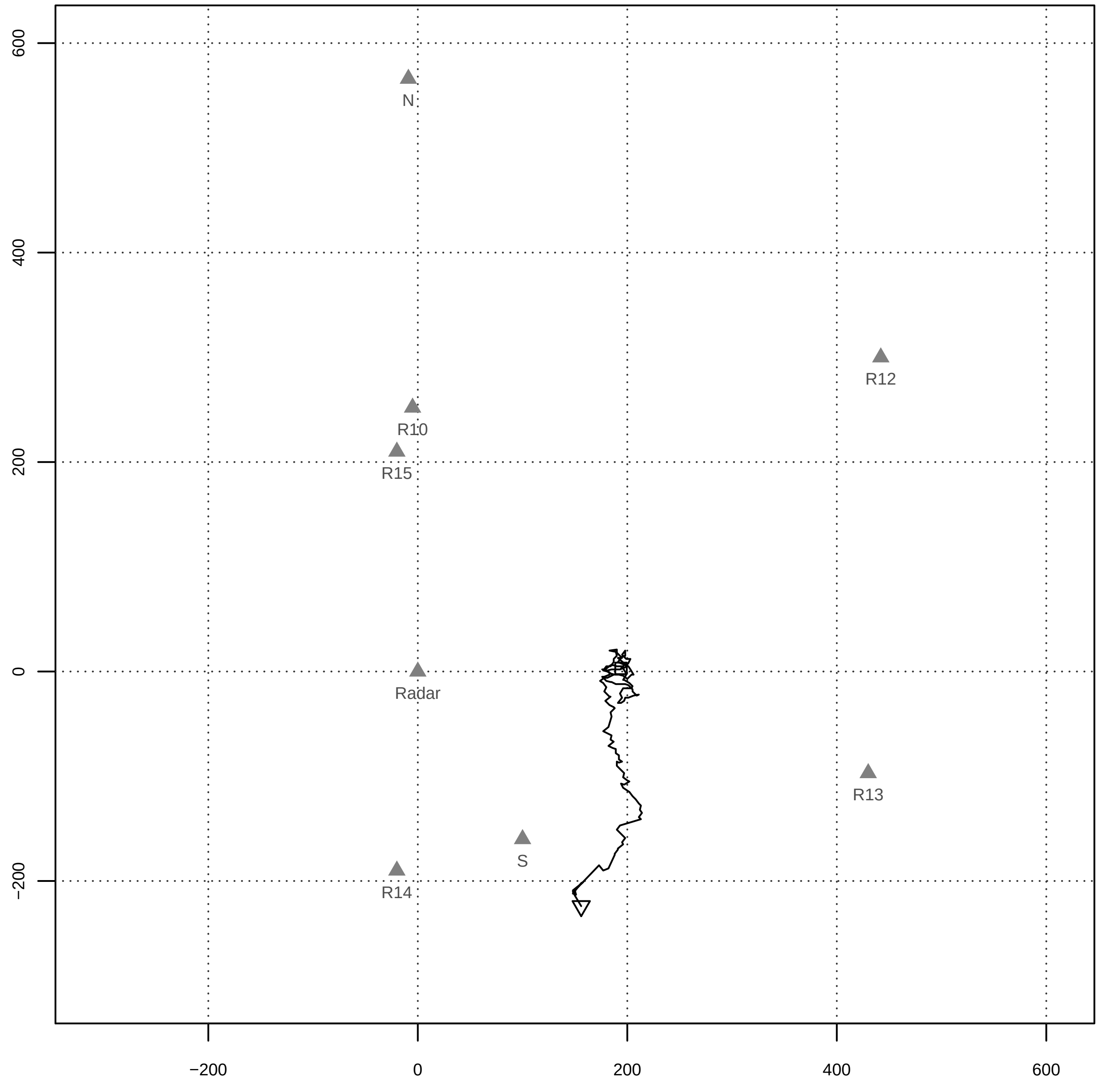

Uwe_neon_yellow_8_Rel_1_400m

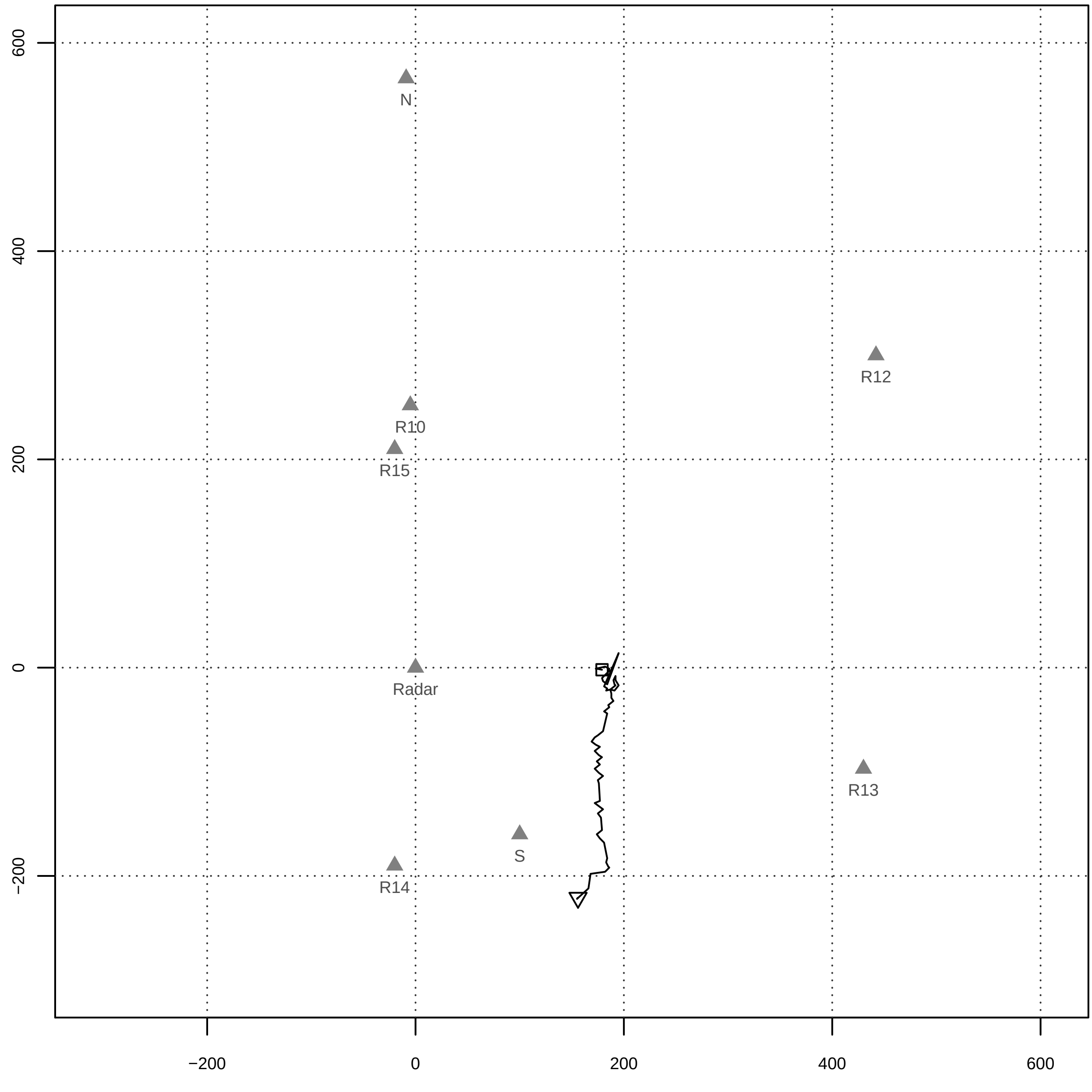

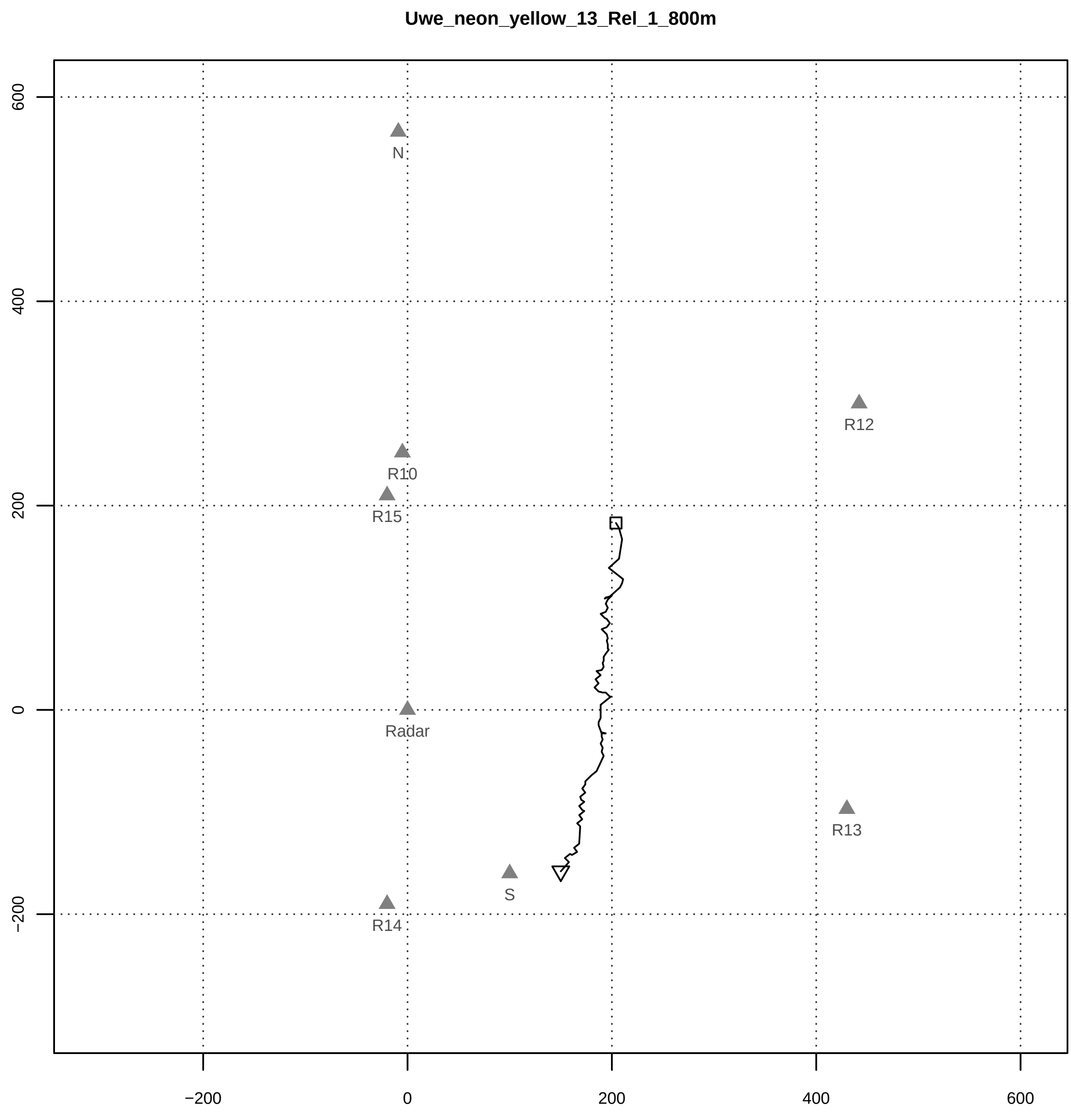
Uwe_neon_yellow_13_Rel_1_800m
N
R12
R10
R15
Radar
R13
S
R14
600
400
200
0
−200
−200
0
200
400
600

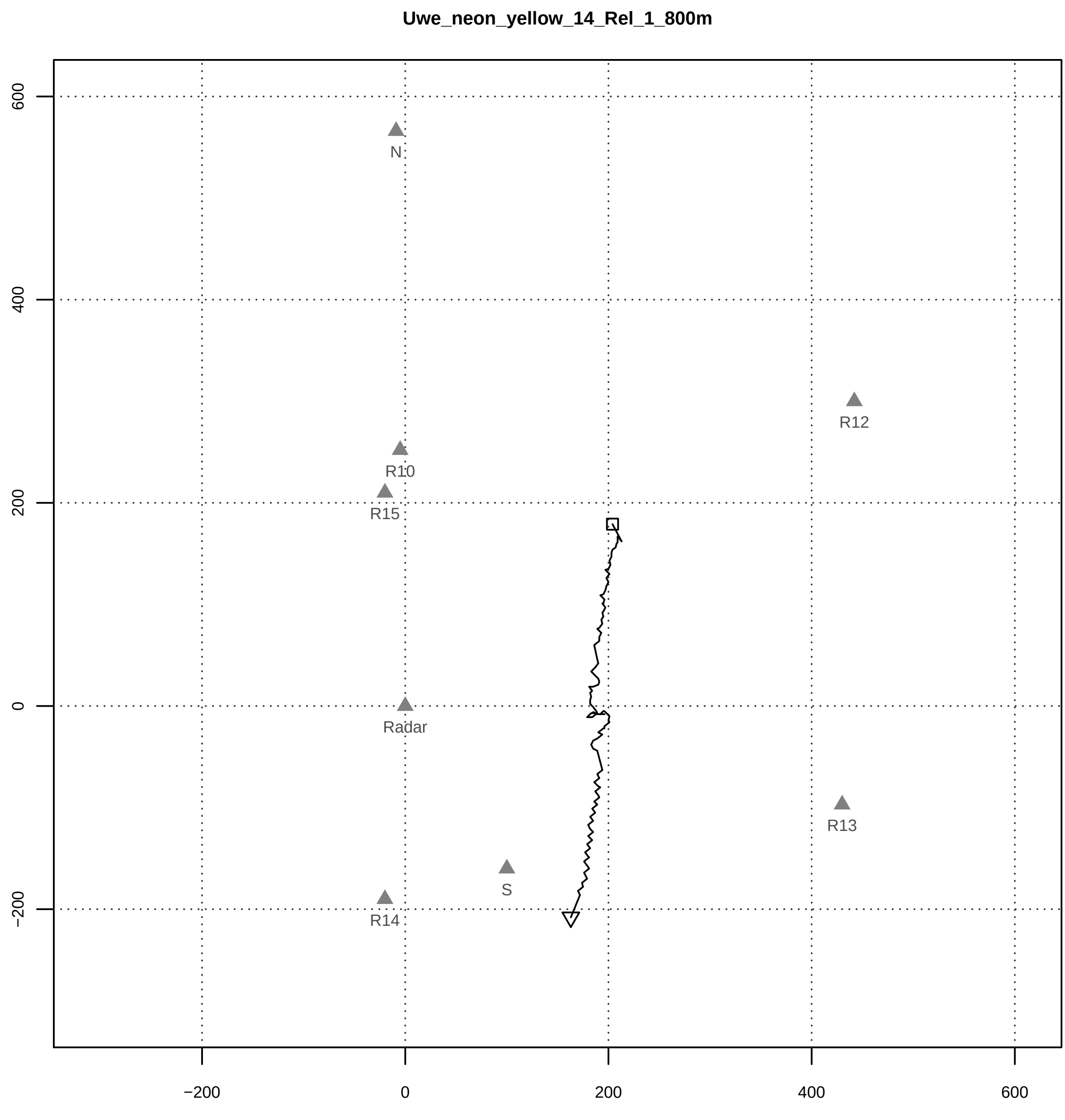

Uwe_neon_yellow_14_Rel_1_800m
N
R12
R10
R15
Radar
R13
S
R14
600
400
200
0
−200
−200
0
200
400
600

Uwe_neon_yellow_17_Rel_1_1200m

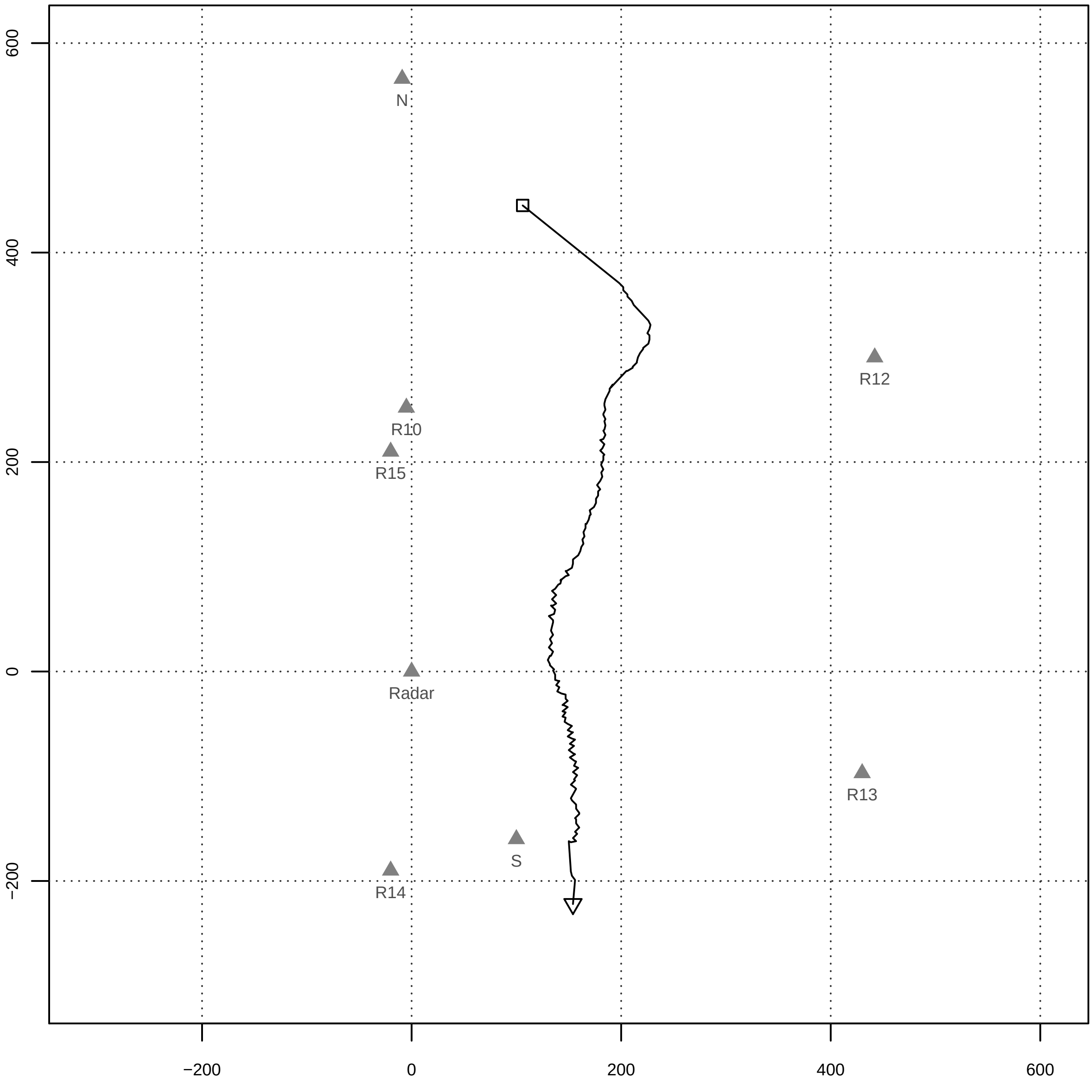

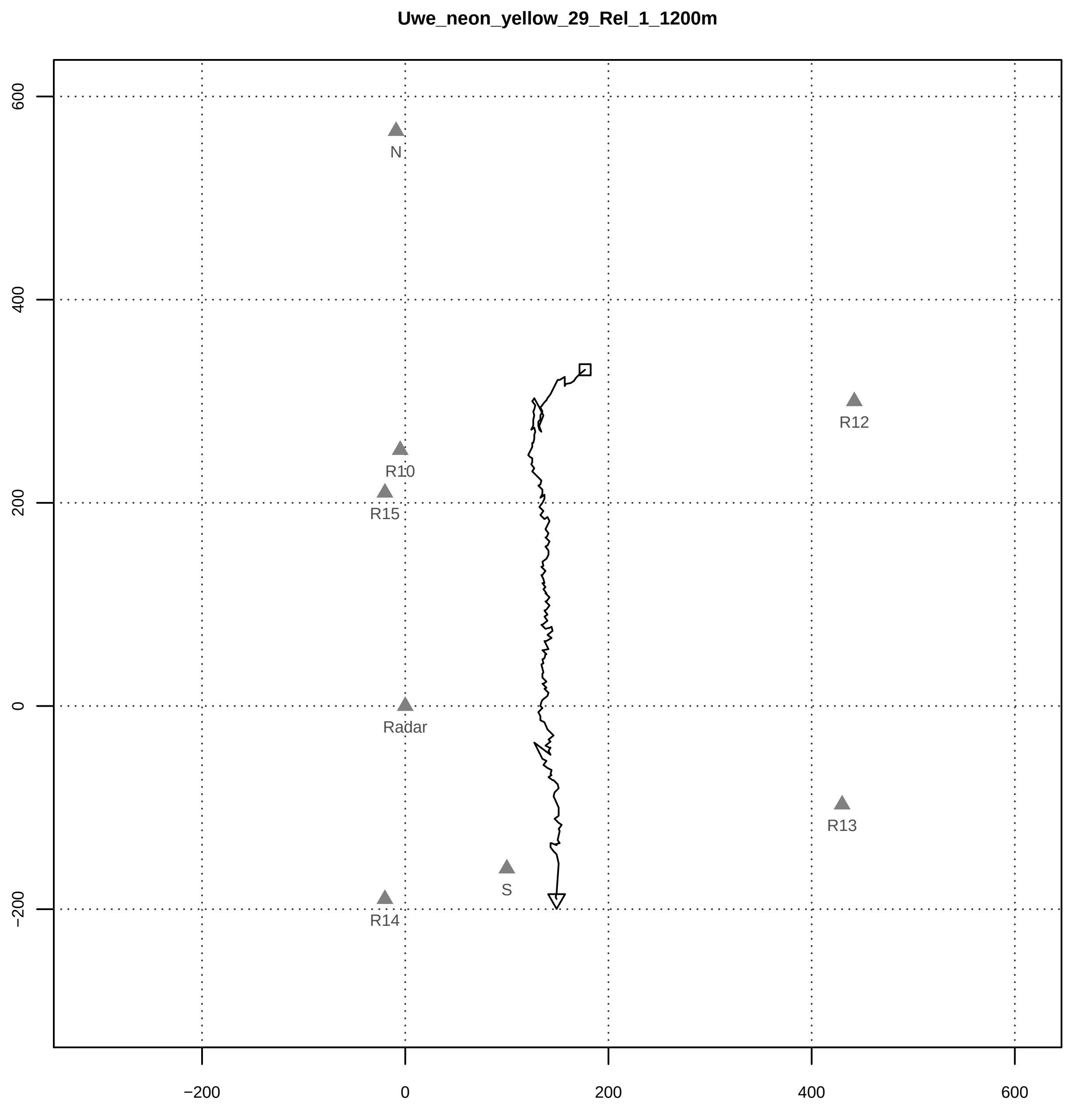
Uwe_neon_yellow_29_Rel_1_1200m
N
R12
R10
R15
Radar
R13
S
R14
600
400
200
0
−200
−200
0
200
400
600

Uwe_neon_yellow_38_Rel_1_1200m

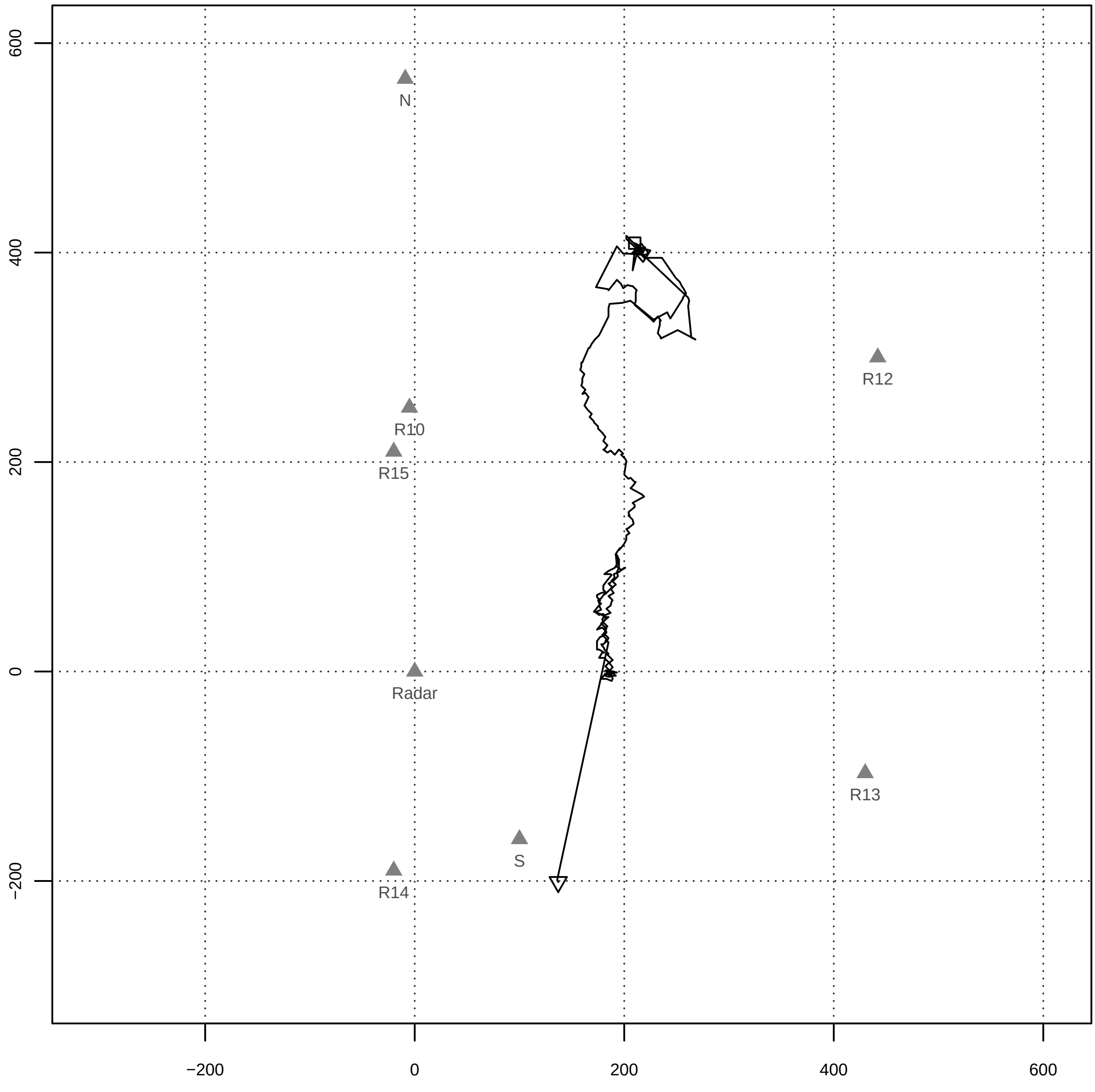

Uwe_neon_yellow_40_Rel_1_1200m

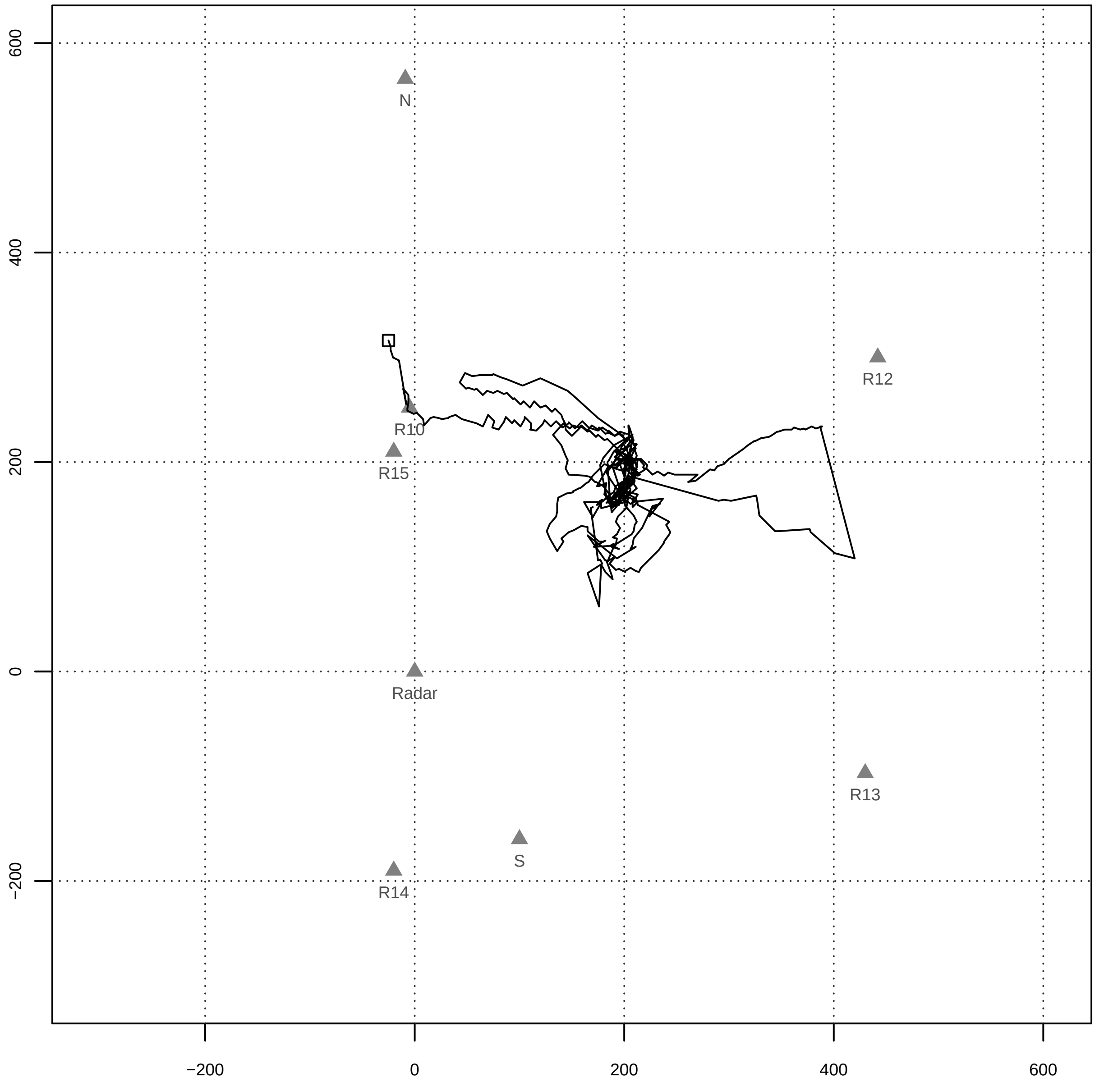

Uwe_neon_yellow_40_Rel_1_1200m−p1−2

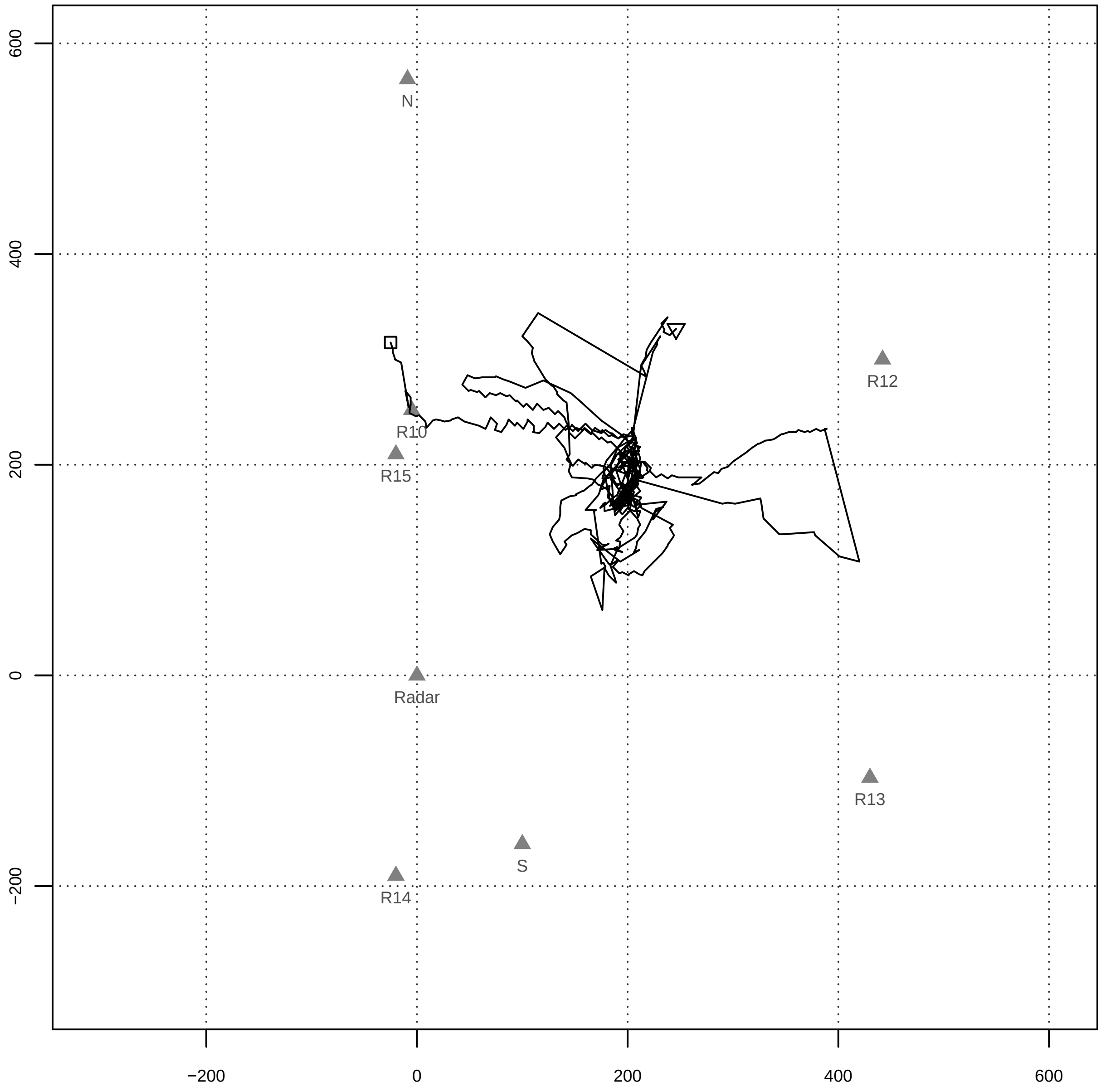

Uwe_neon_yellow_47_Rel_1_400m

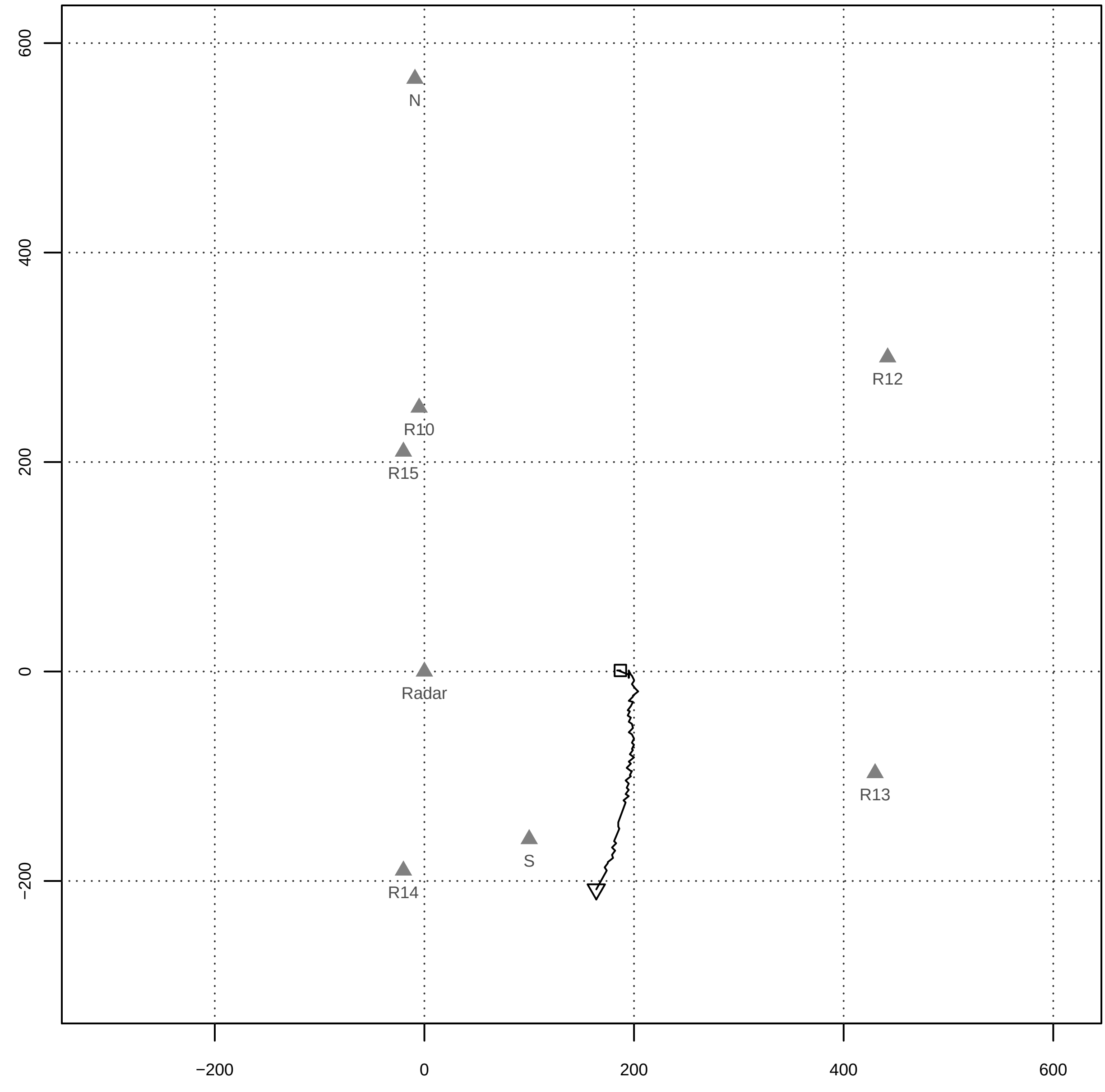

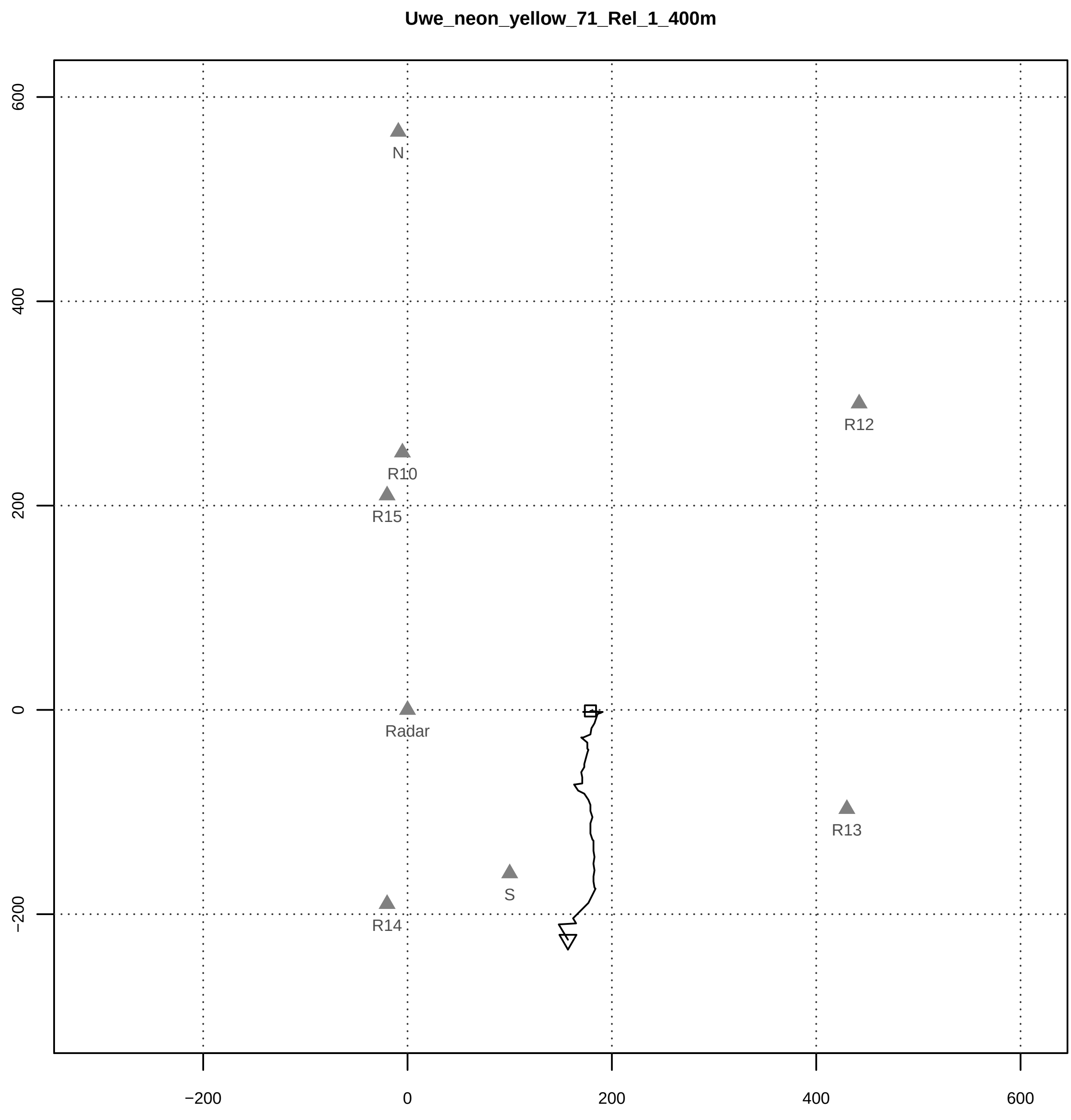

Uwe_neon_yellow_71_Rel_1_400m
N
R12
R10
R15
Radar
R13
S
R14
600
400
200
0
−200
−200
0
200
400
600

Uwe_neon_yellow_95_Rel_1_400m

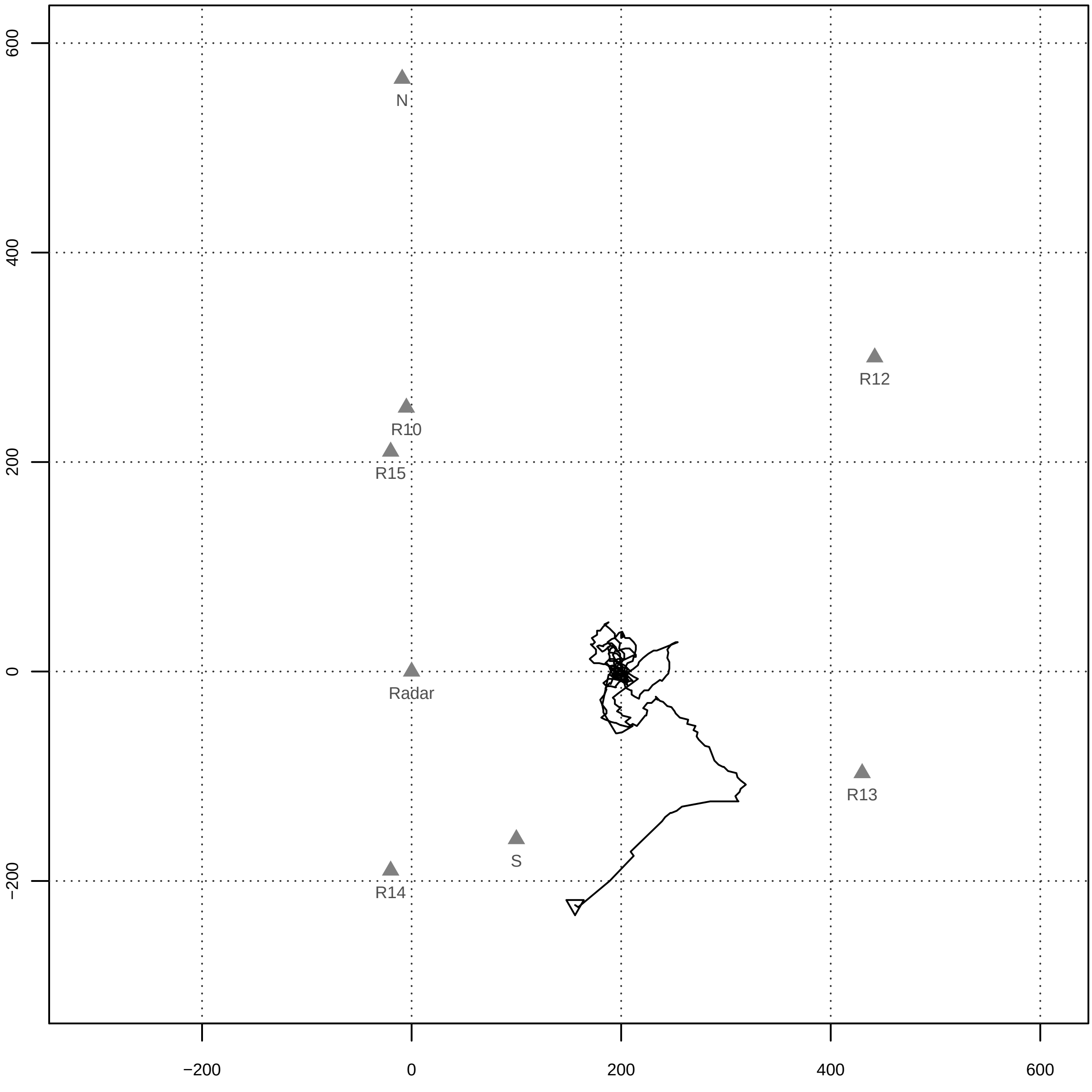

Uwe_neon_yellow_96_Rel_1_400m

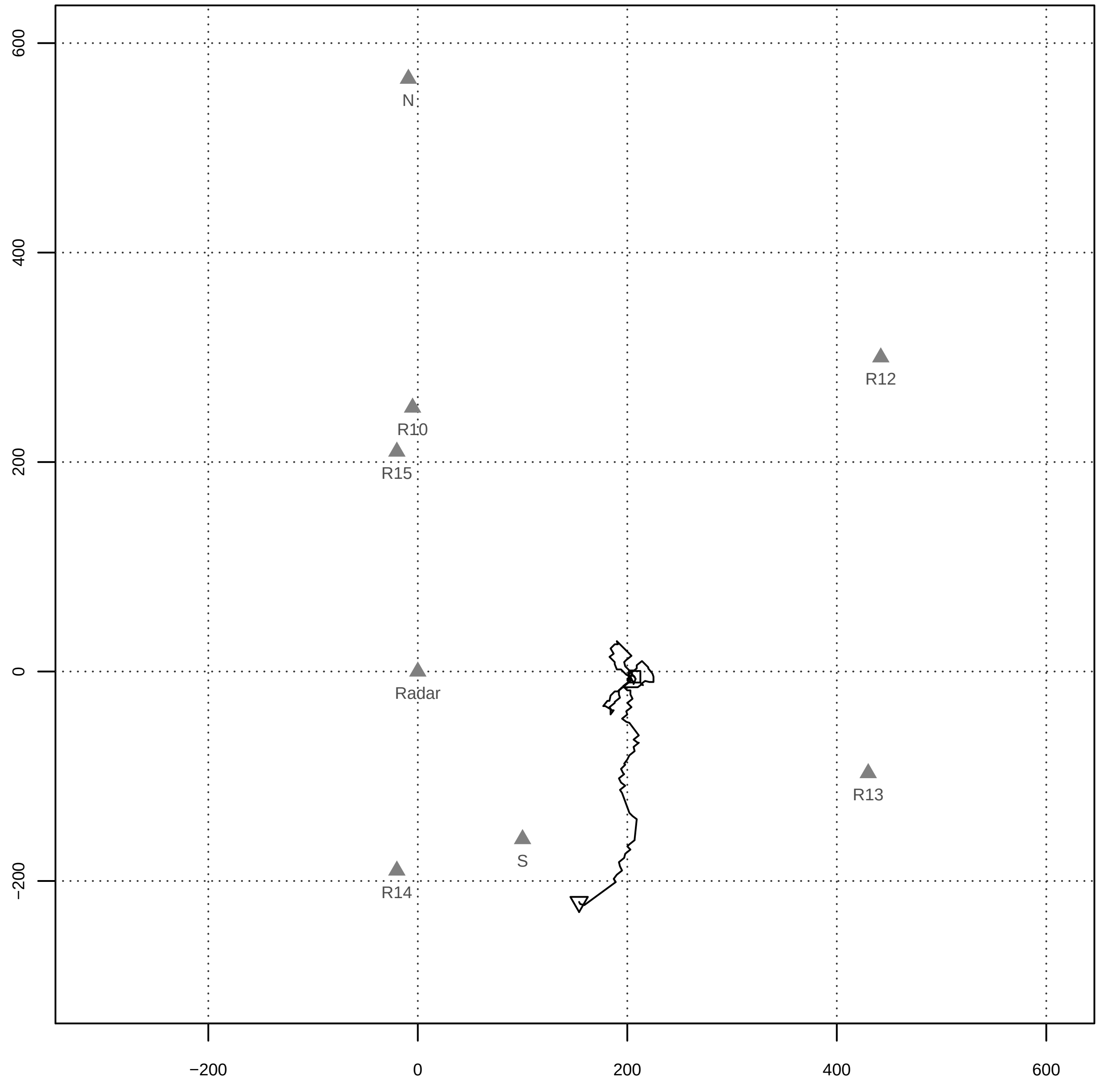

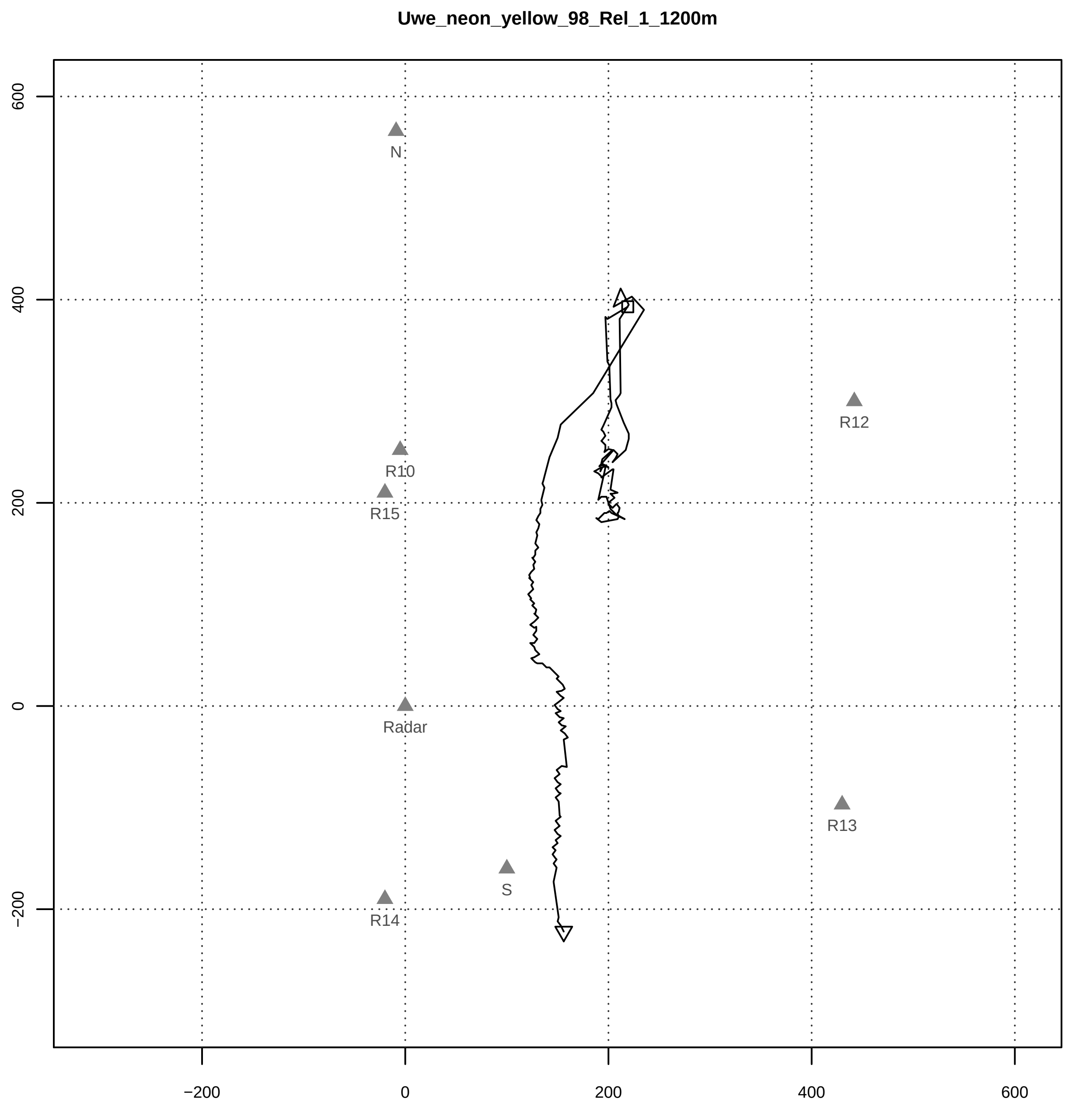

Uwe_neon_yellow_98_Rel_1_1200m
600
400
200
0
−200
−200
0
200
400
600
N
R10
R15
R12
Radar
R13
S
R14

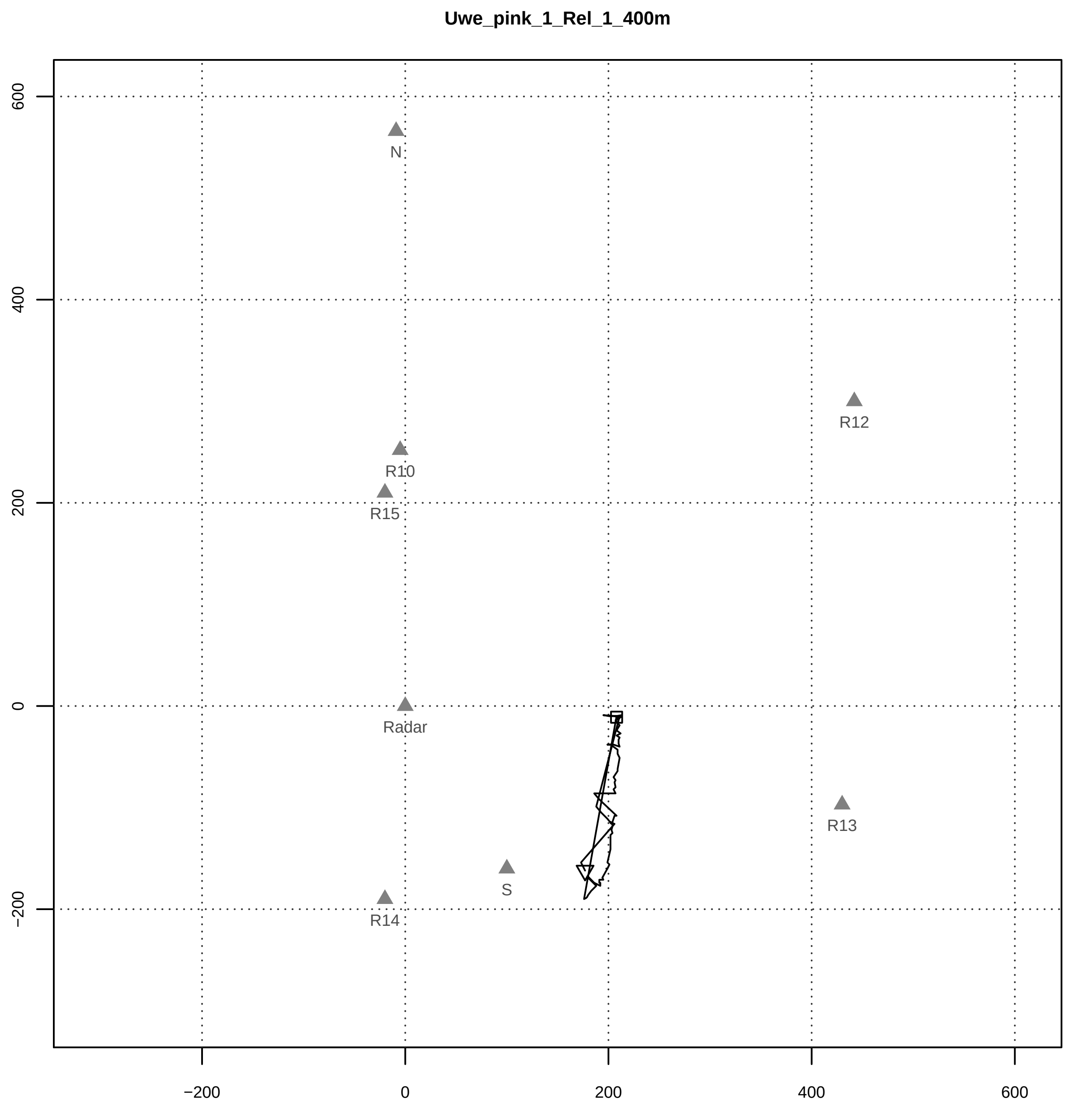

Uwe_pink_1_Rel_1_400m
N
R10
R15
R12
Radar
R13
S
R14
600
400
200
0
−200
−200
0
200
400
600

Uwe_pink_4_Rel_1_400m

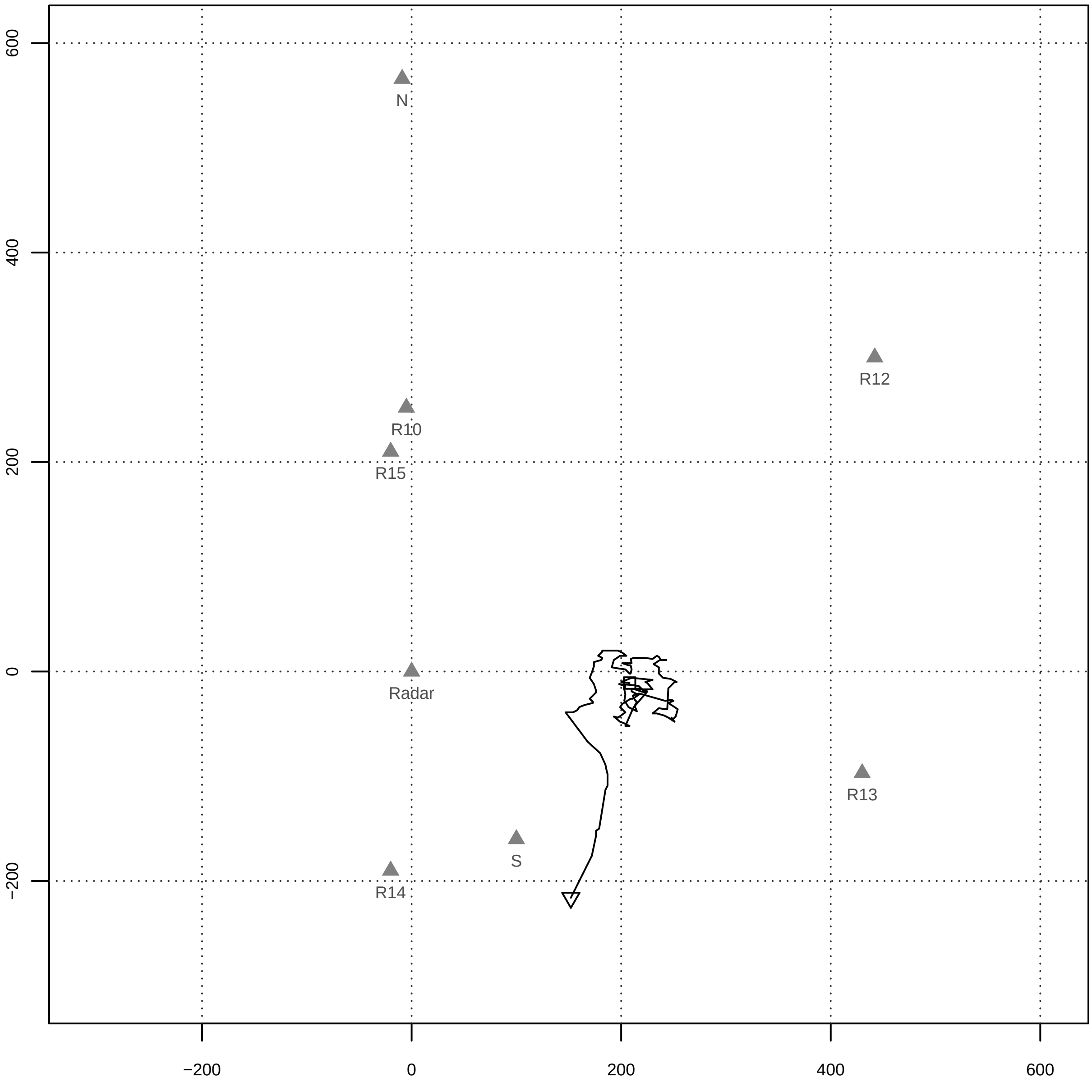

Uwe_pink_4_Rel_1_800m

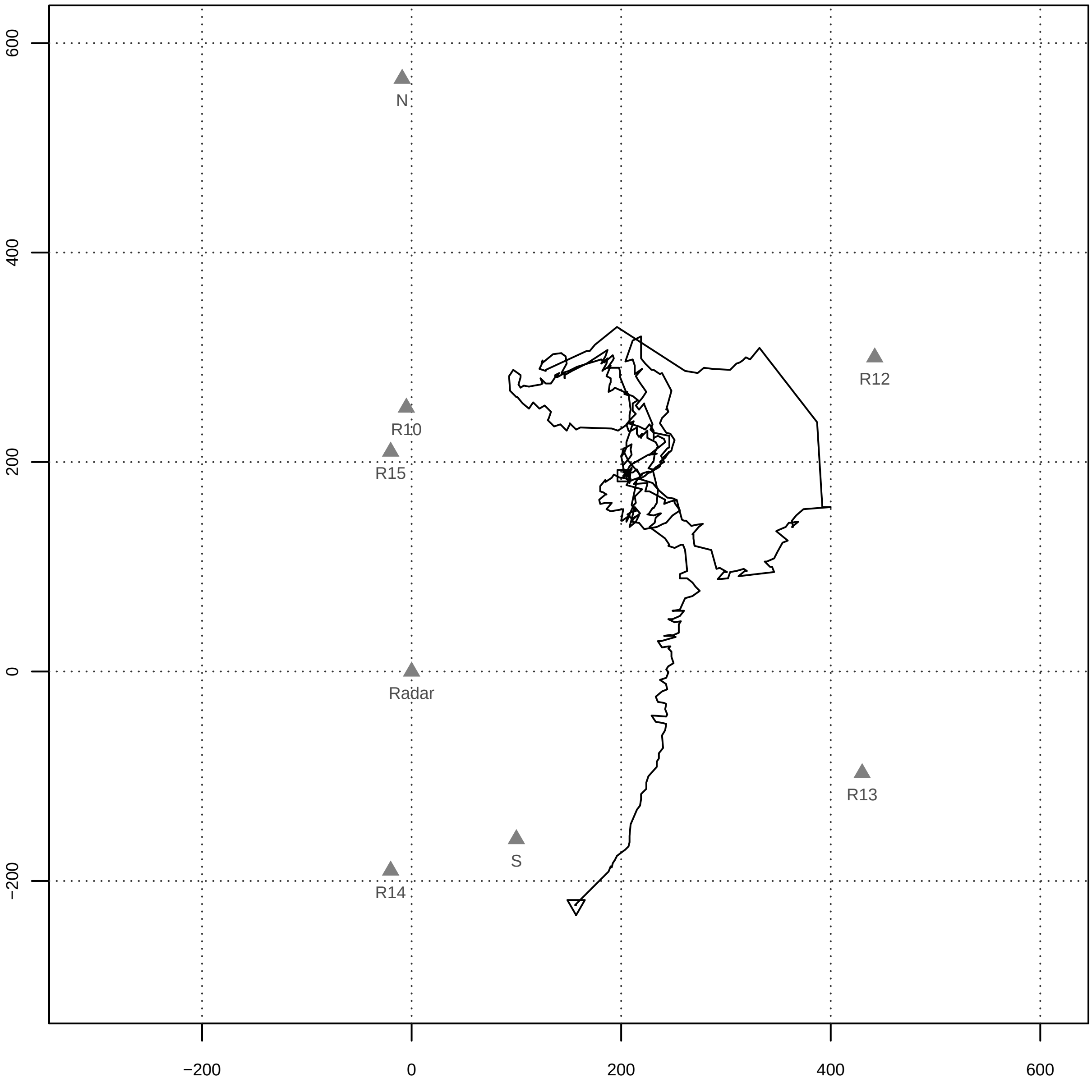

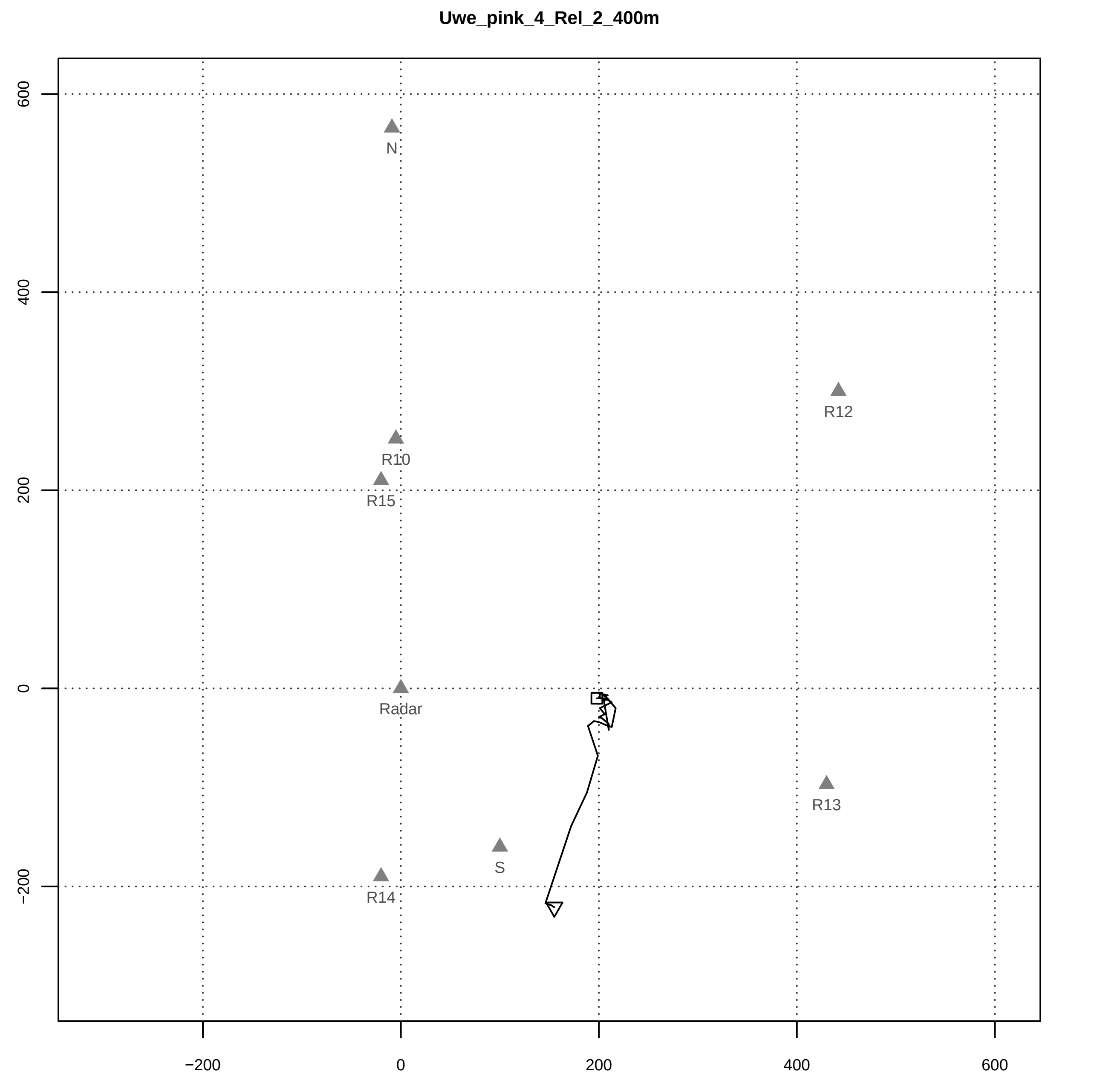

Uwe_pink_4_Rel_2_400m
N
R12
R10
R15
Radar
R13
S
R14
600
400
200
0
−200
−200
0
200
400
600

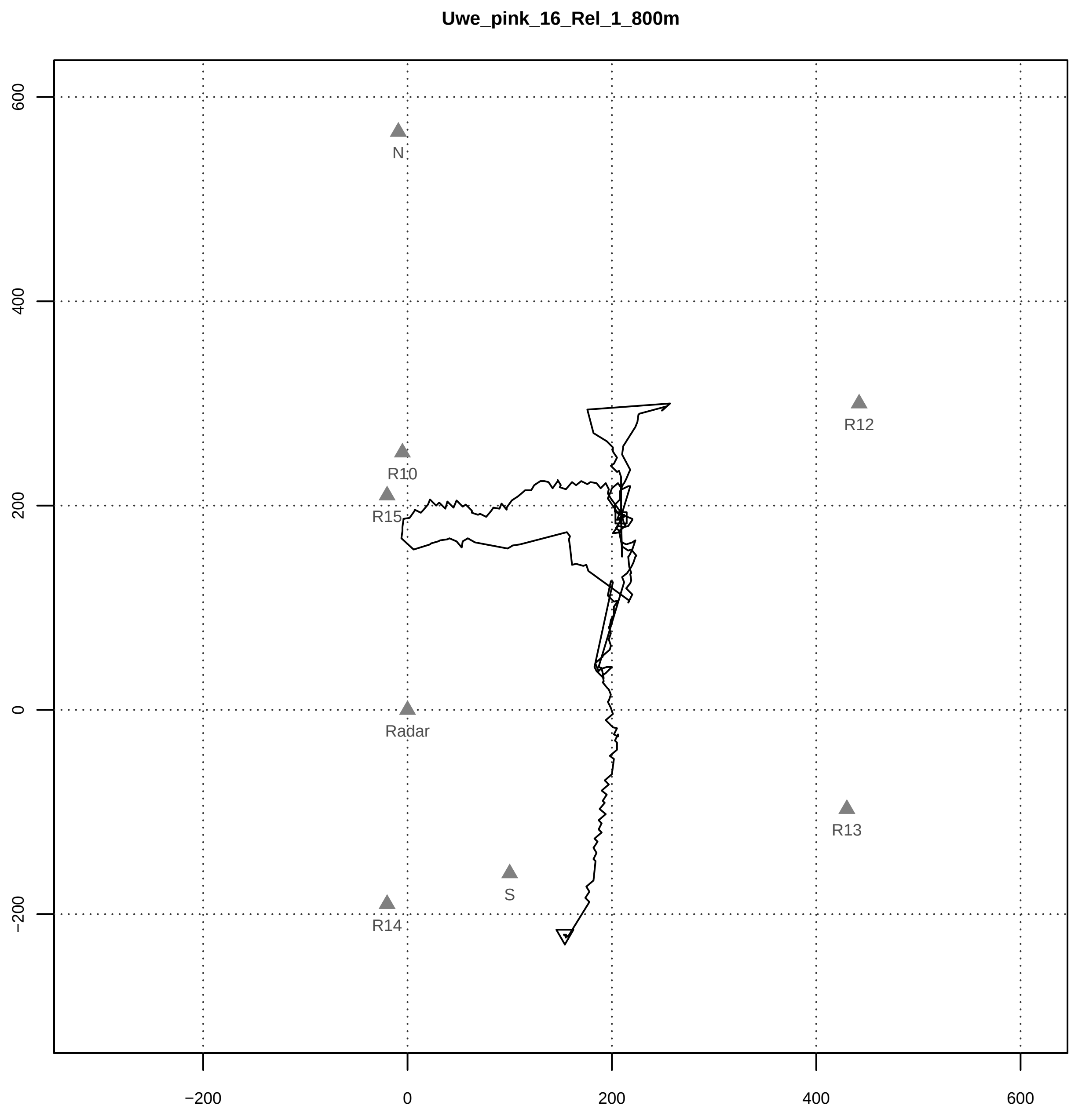

Uwe_pink_16_Rel_1_800m
N
R12
R10
R15
Radar
R13
S
R14
−200
0
200
400
600
−200
0
200
400
600

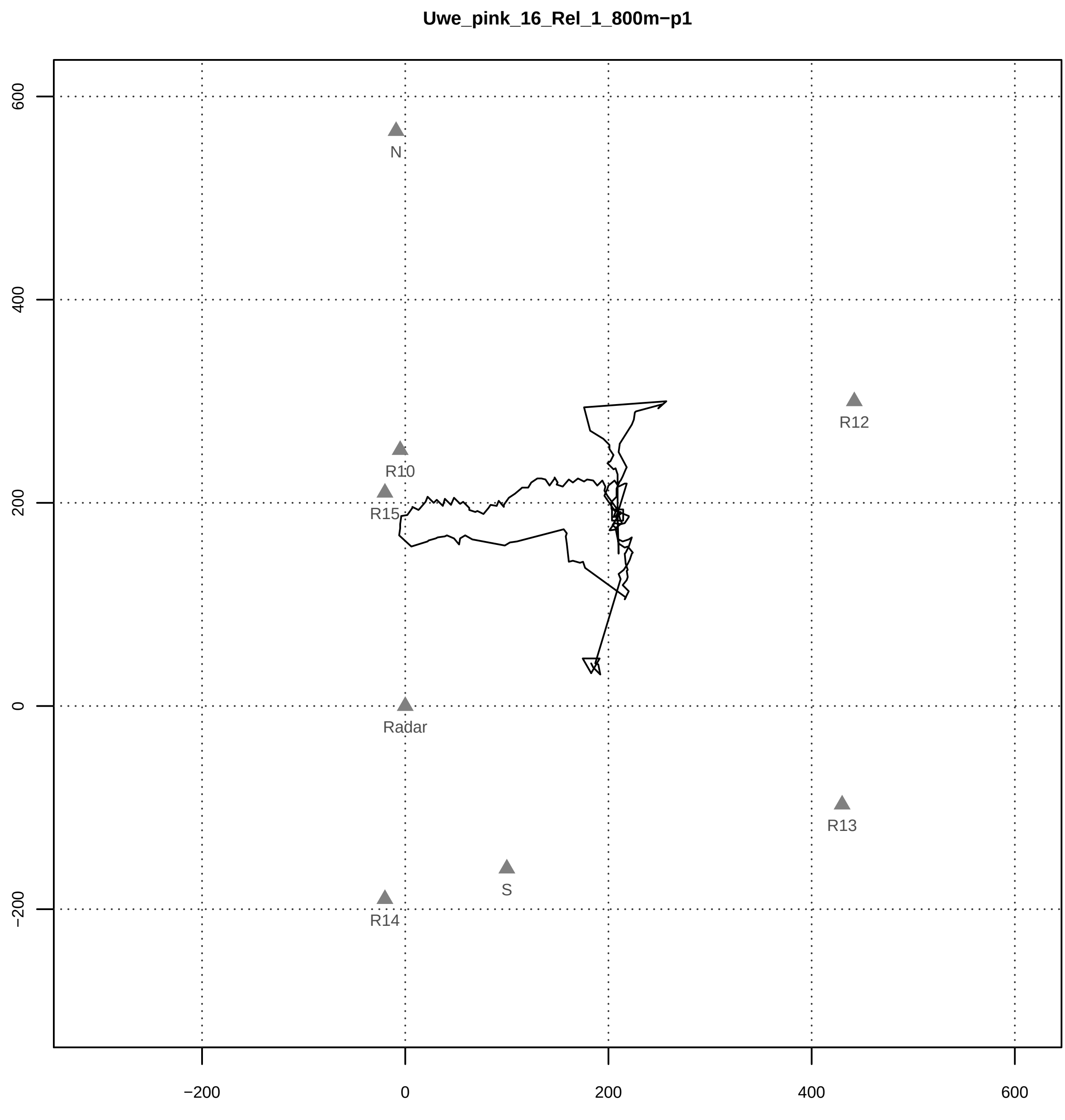
Uwe_pink_16_Rel_1_800m−p1
N
R10
R15
R12
Radar
R13
S
R14
−200
0
200
400
600
−200
0
200
400
600

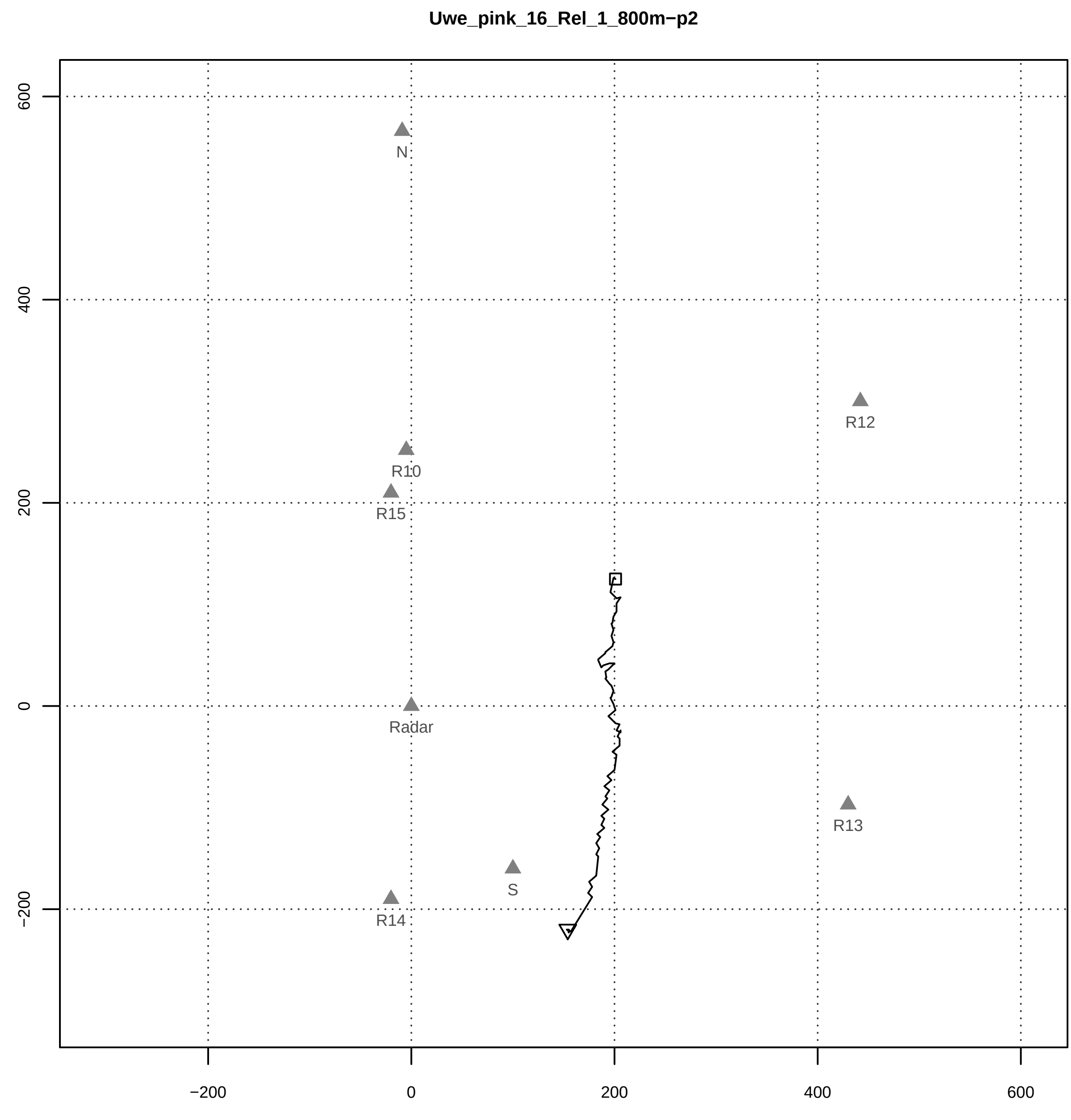

Uwe_pink_16_Rel_1_800m−p2
N
R12
R10
R15
Radar
R13
S
R14
−200
0
200
400
600

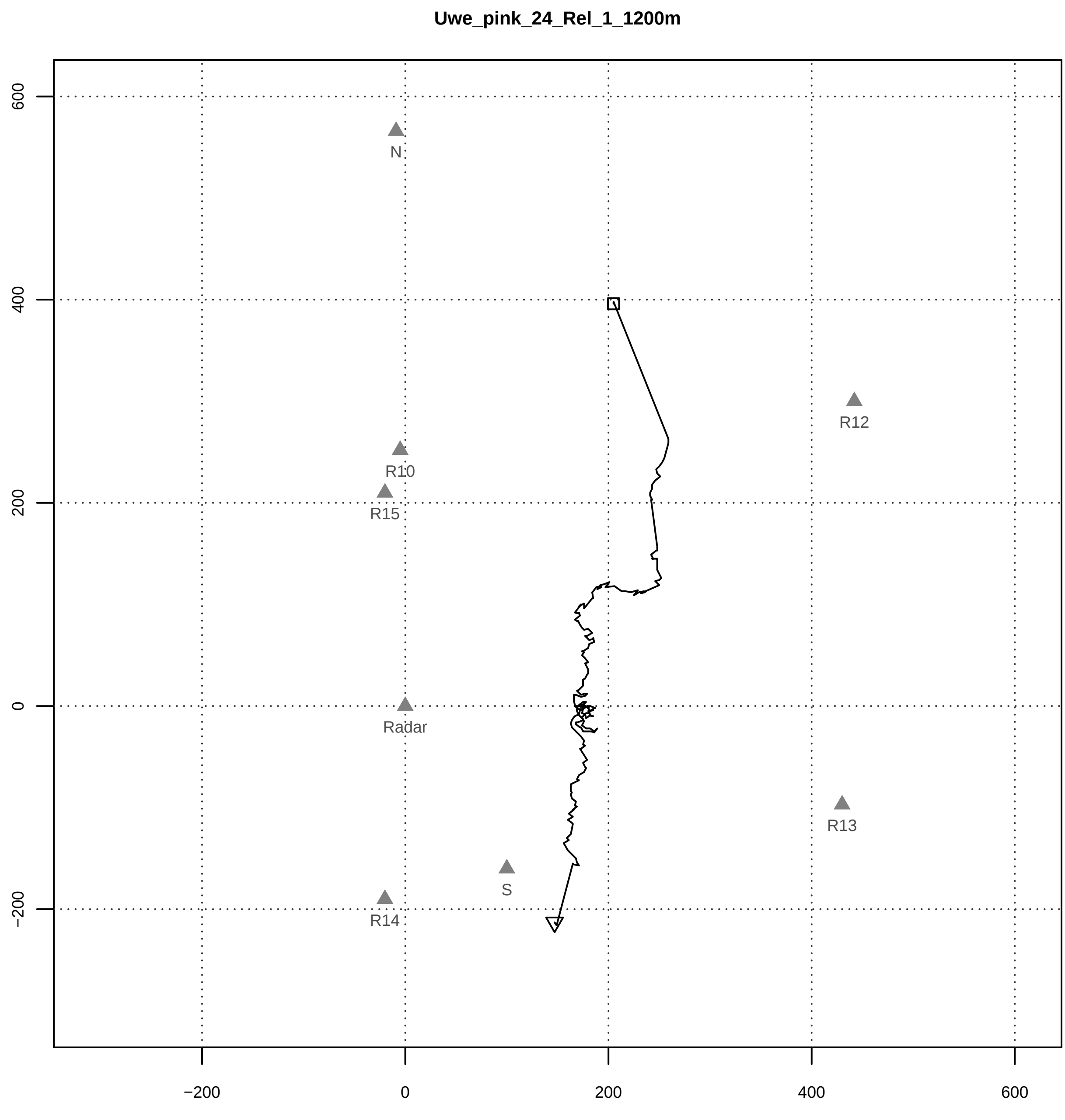
Uwe_pink_24_Rel_1_1200m
N
R10
R15
R12
Radar
R13
S
R14
600
400
200
0
−200
−200
0
200
400
600

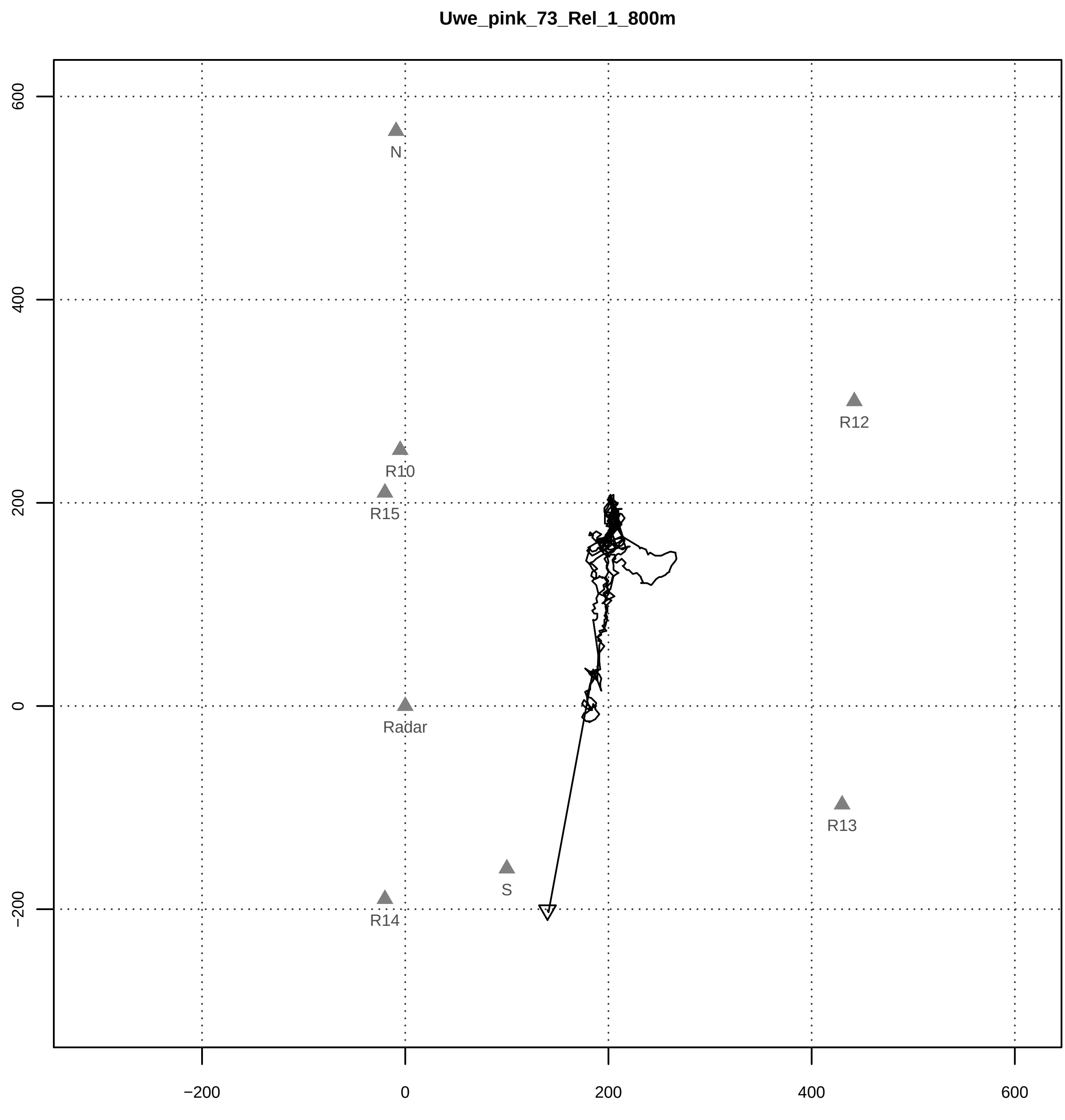
Uwe_pink_73_Rel_1_800m
N
R12
R10
R15
Radar
R13
S
R14
−200
0
200
400
600
−200
0
200
400
600

Uwe_pink_73_Rel_2_800m

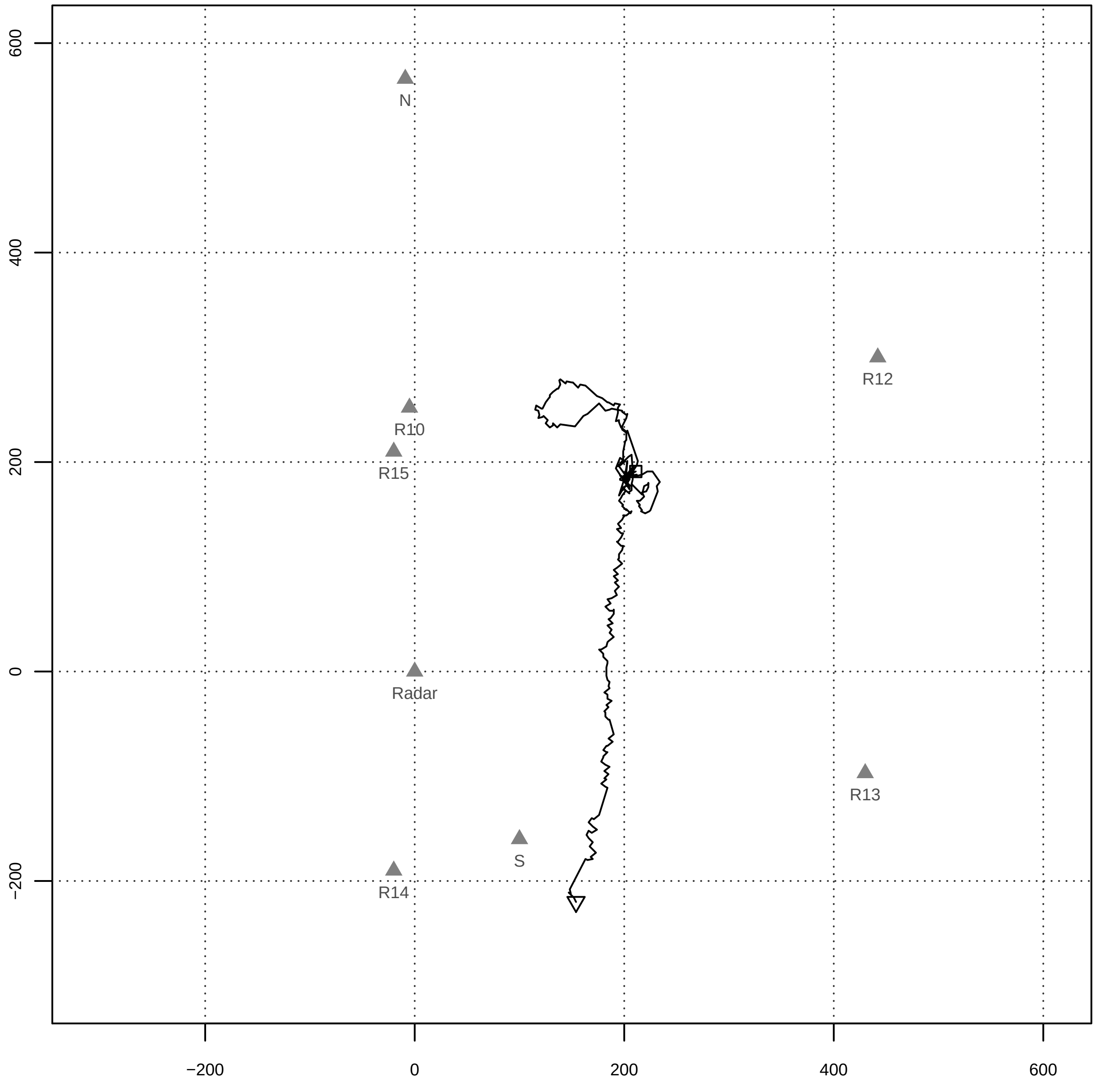

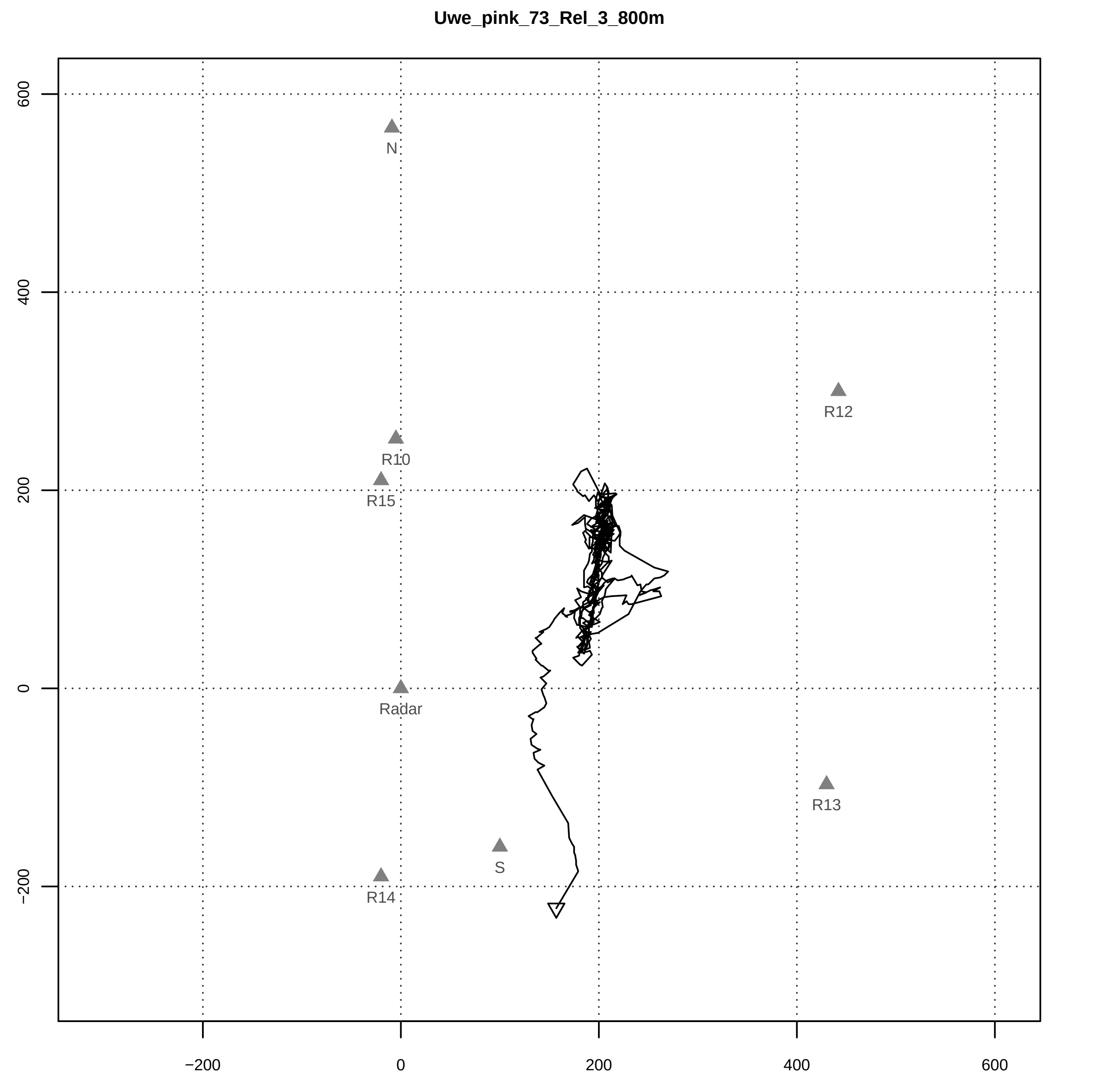

Uwe_pink_73_Rel_3_800m
N
R10
R15
R12
Radar
R13
S
R14
600
400
200
0
−200
−200
0
200
400
600

Uwe_pink_73_Rel_3_800m−p1

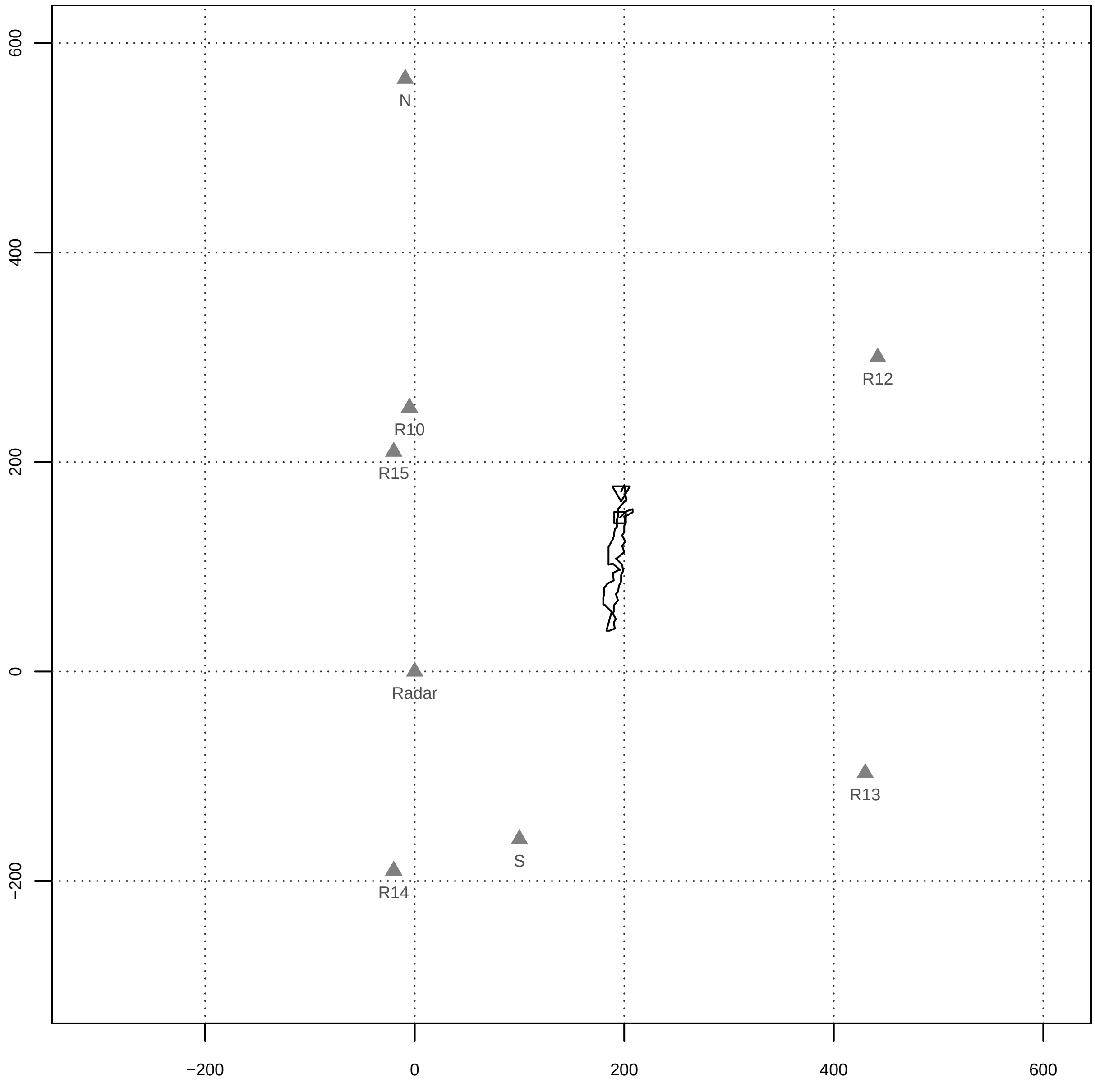

Uwe_pink_73_Rel_3_800m−p2

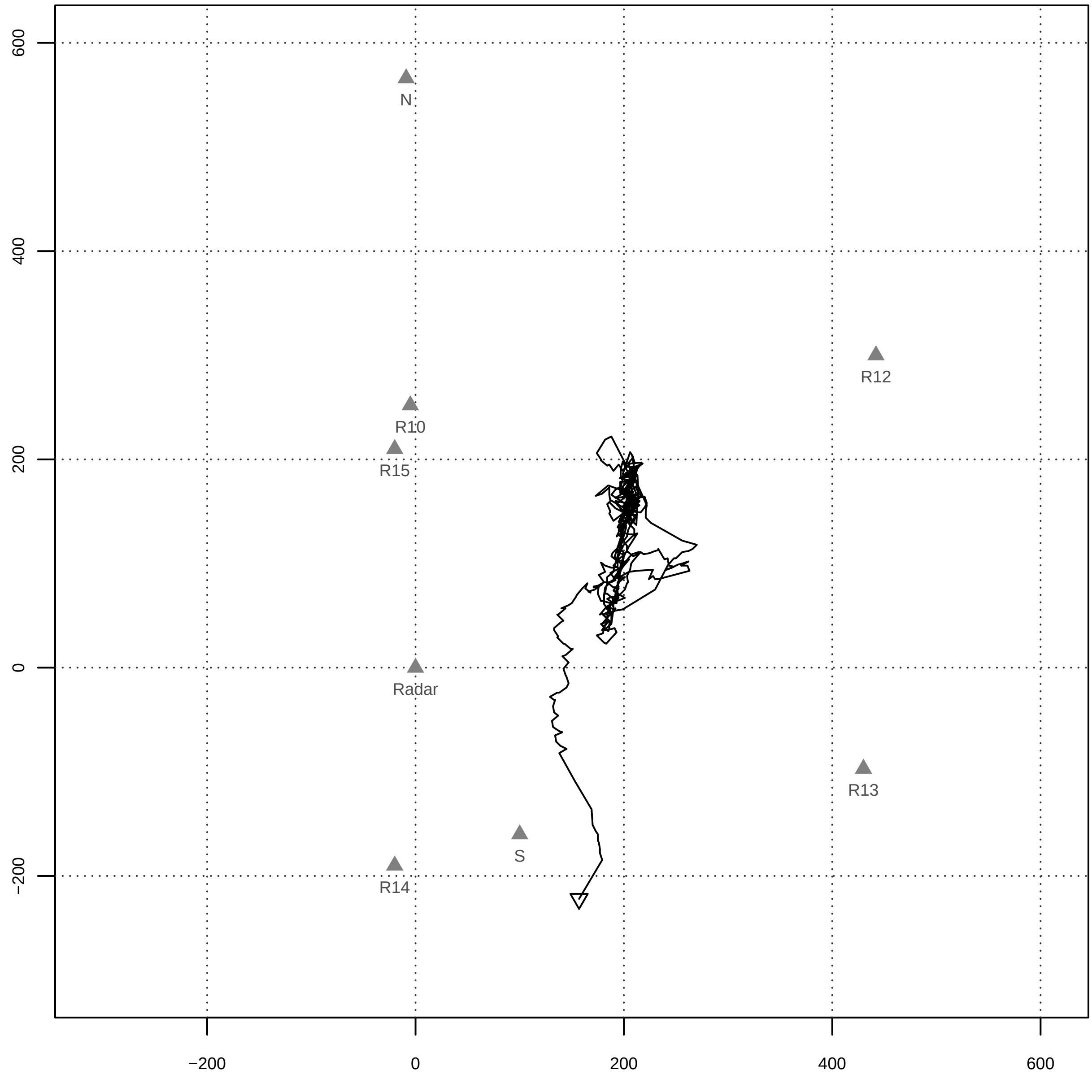

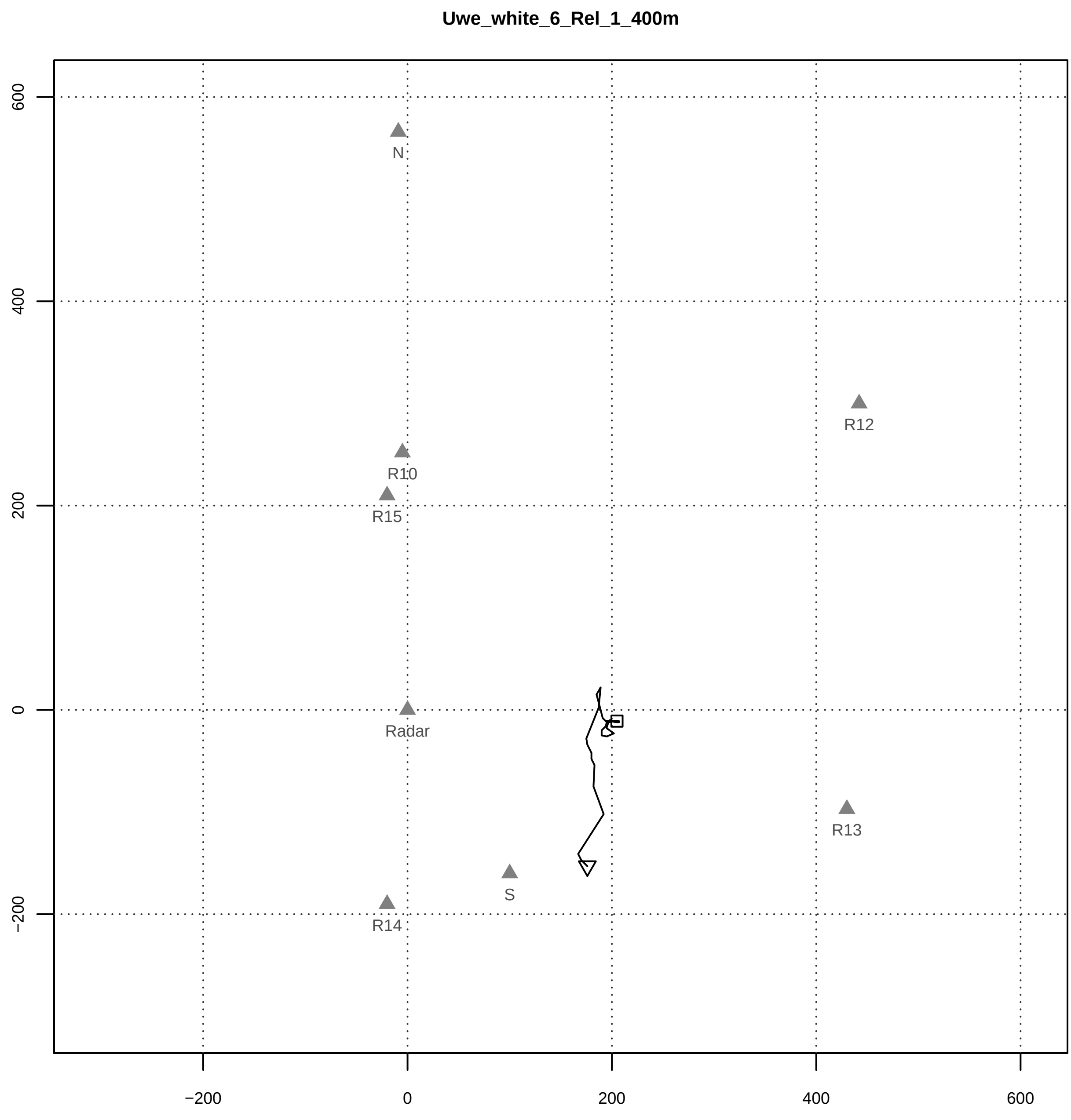

Uwe_white_6_Rel_1_400m
N
R12
R10
R15
Radar
R13
S
R14
−200
0
200
400
600

Uwe_white_6_Rel_2_400m

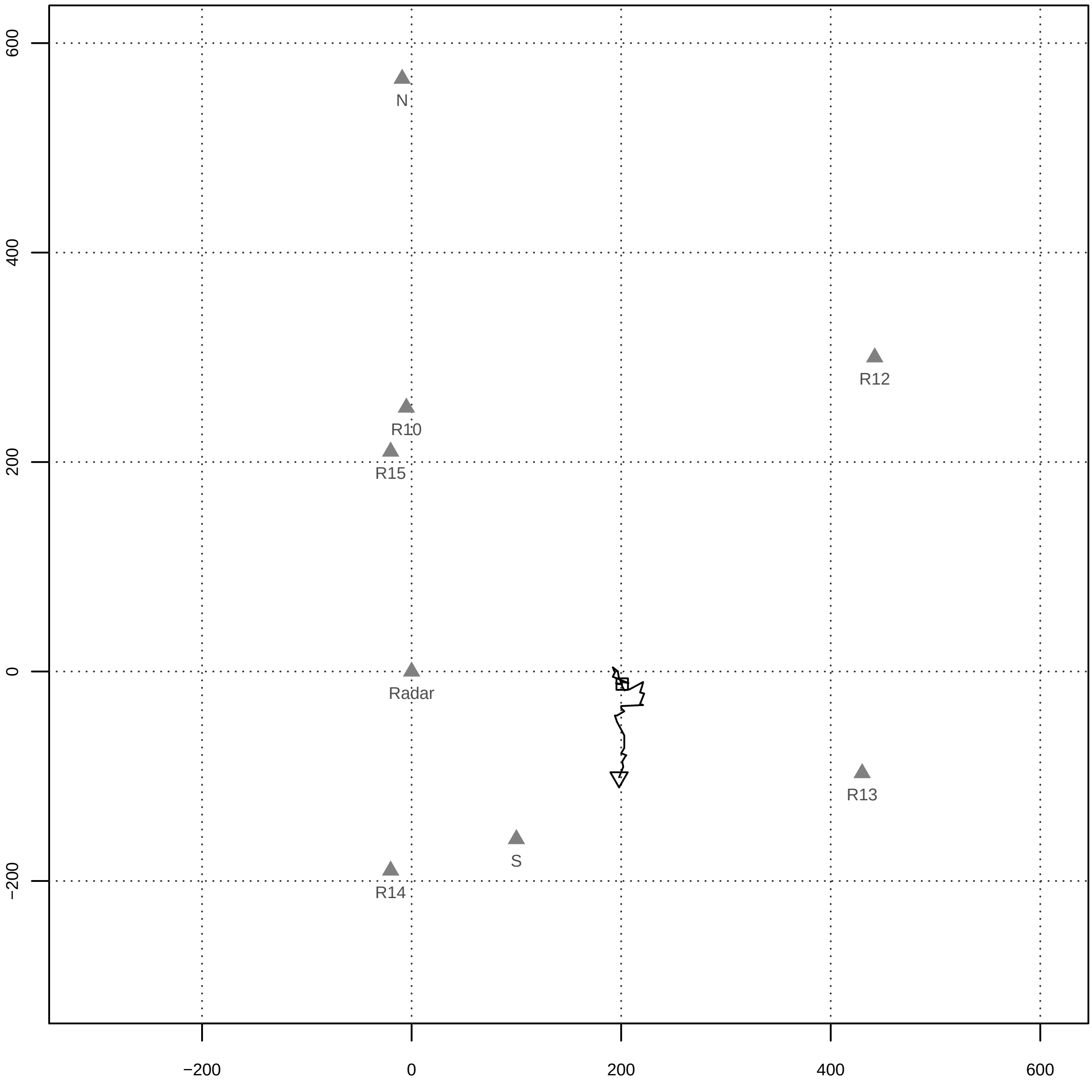

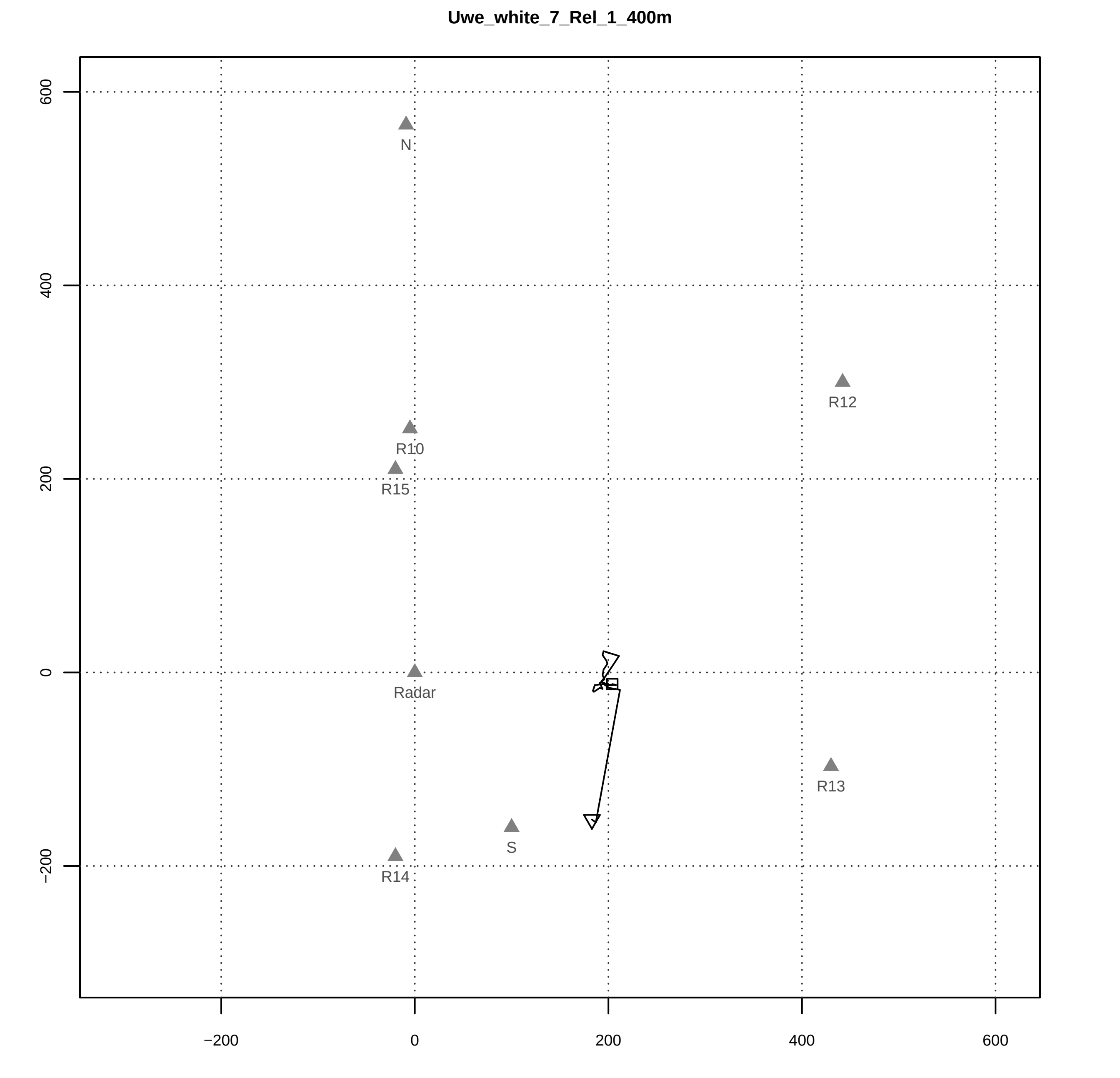

Uwe_white_7_Rel_1_400m
N
R12
R10
R15
Radar
R13
S
R14
600
400
200
0
−200
−200
0
200
400
600

Uwe_white_10_Rel_1_400m

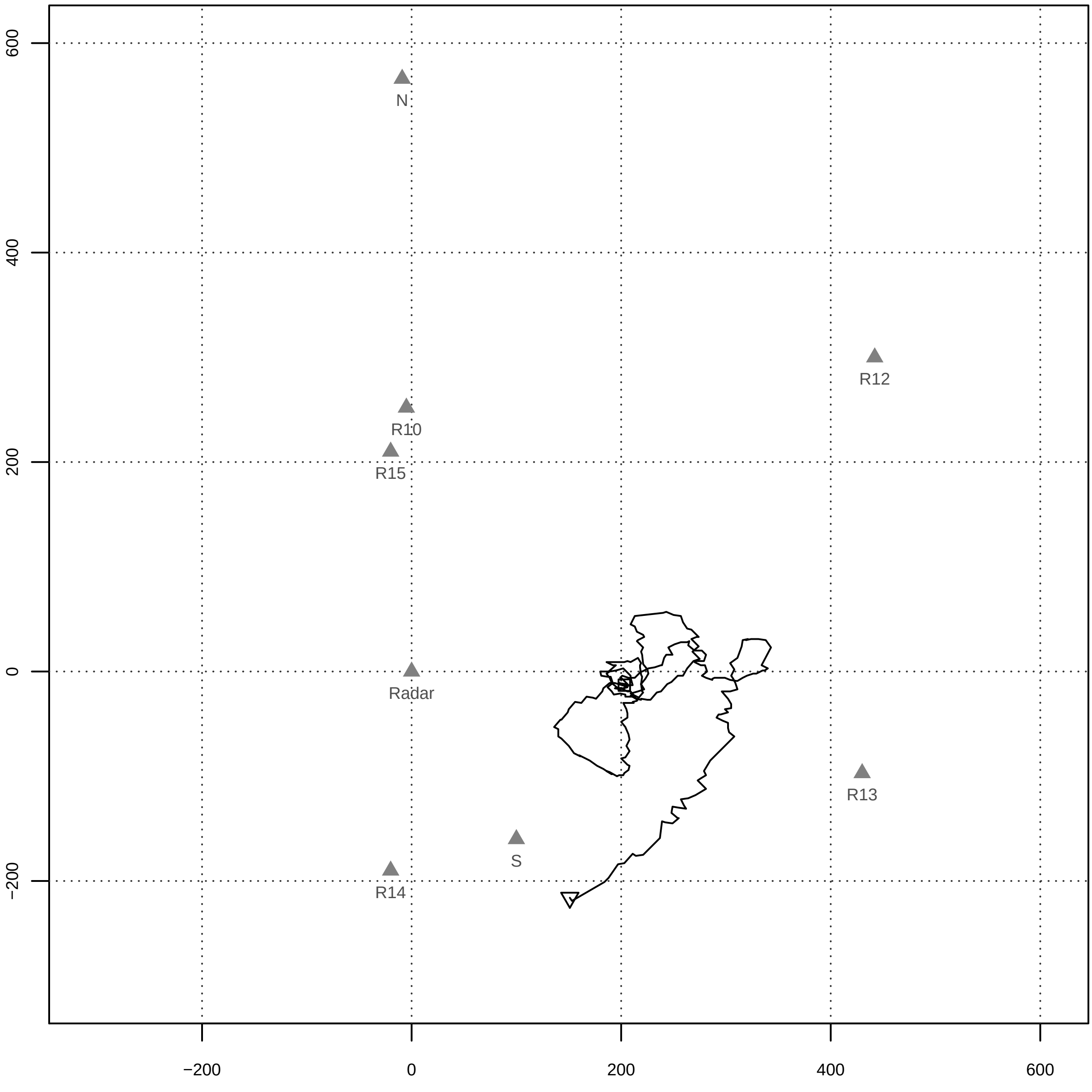

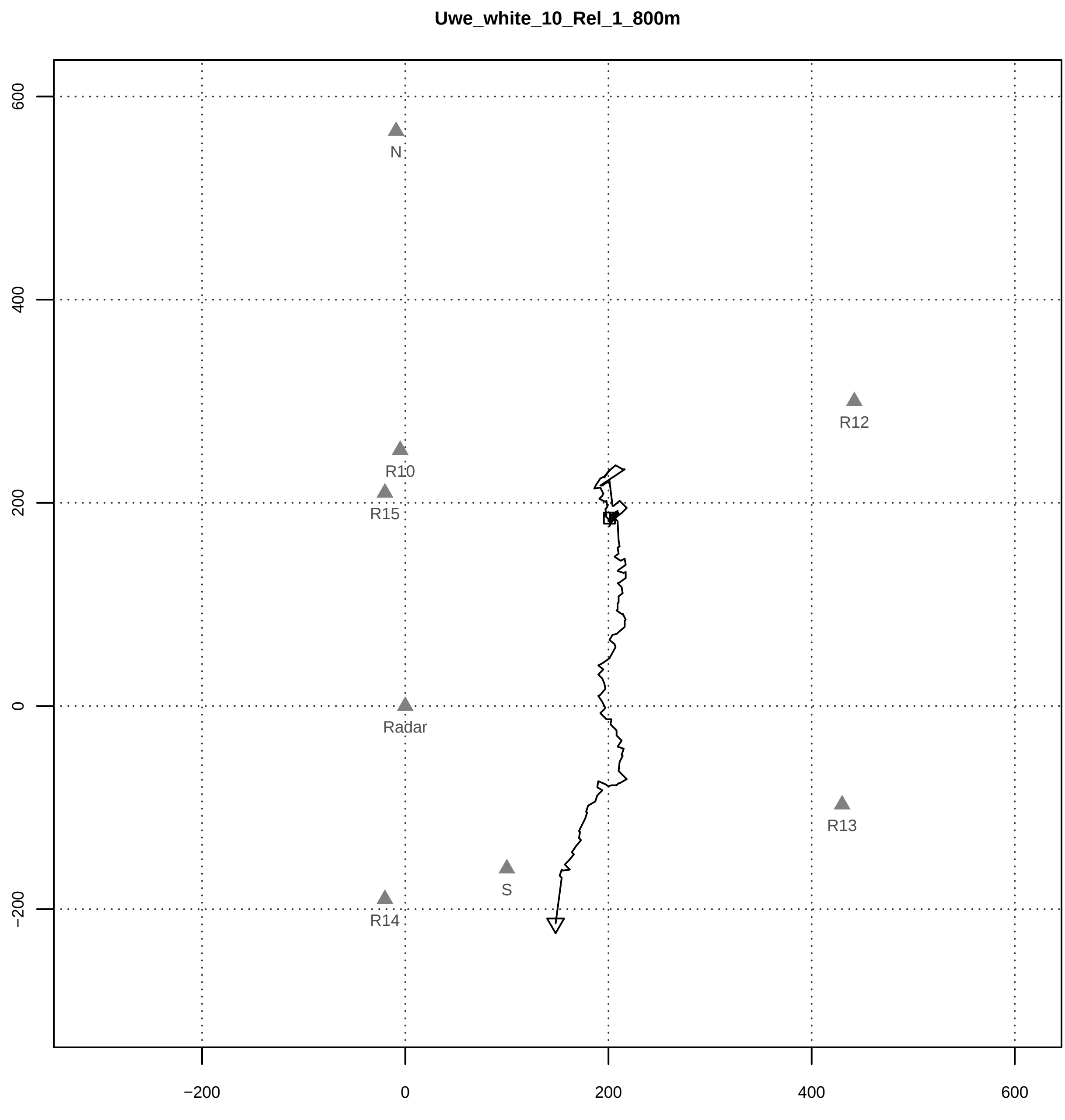

Uwe_white_10_Rel_1_800m
600
400
200
0
−200
−200
0
200
400
600
N
R12
R10
R15
Radar
R13
S
R14

Uwe_white_10_Rel_1_1000m

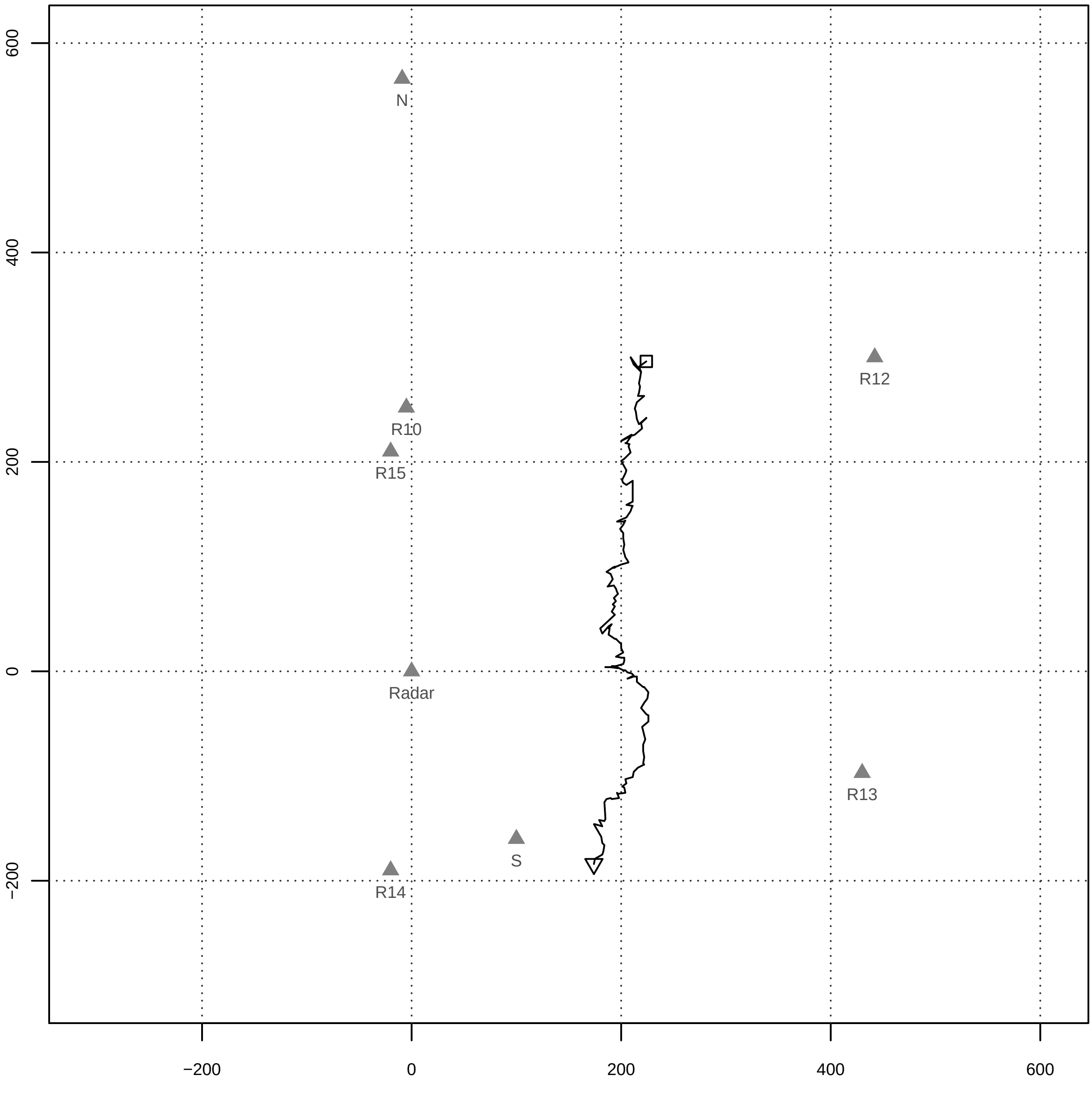

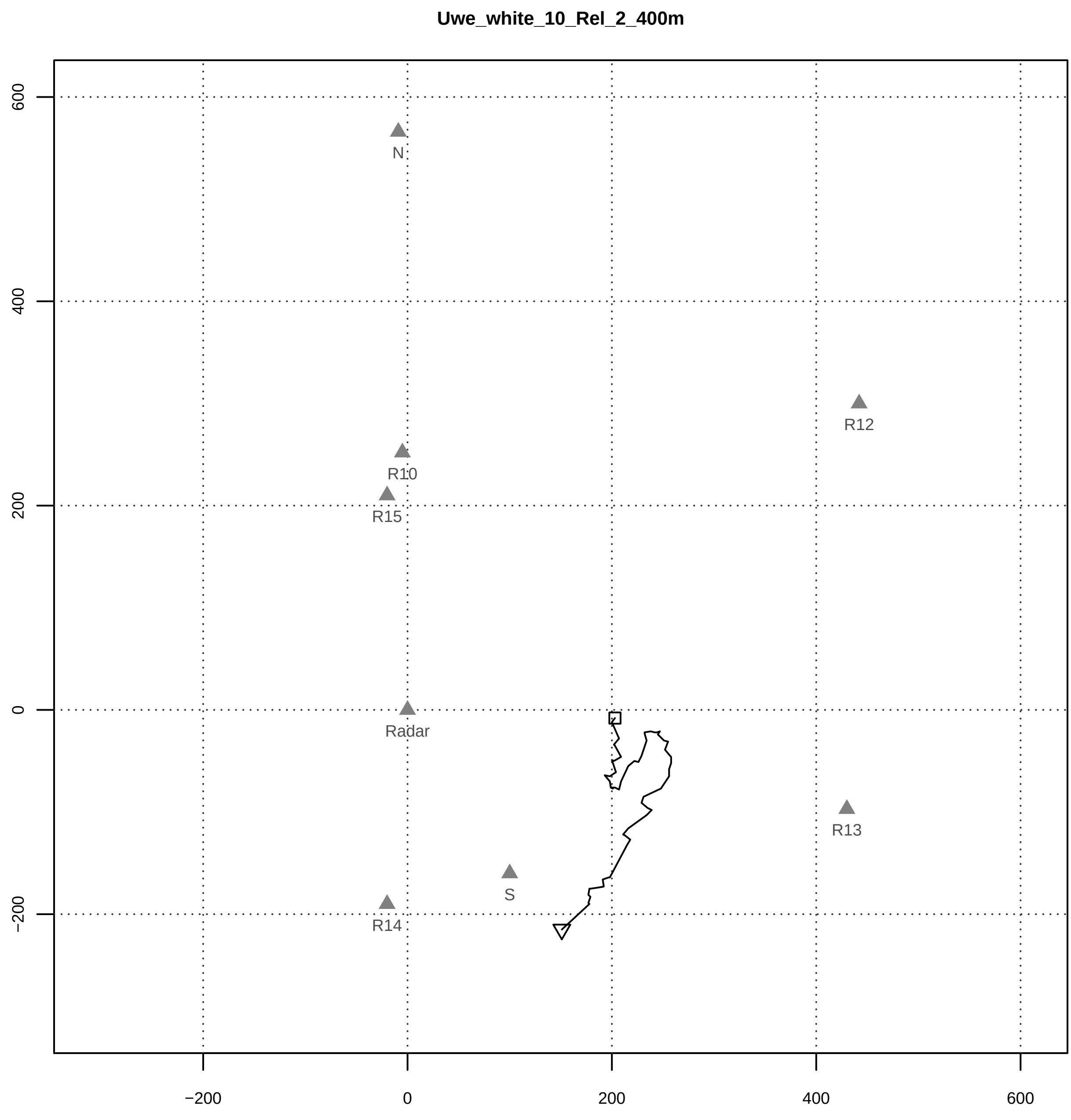
Uwe_white_10_Rel_2_400m
N
R12
R10
R15
Radar
R13
S
R14
600
400
200
0
−200
−200
0
200
400
600

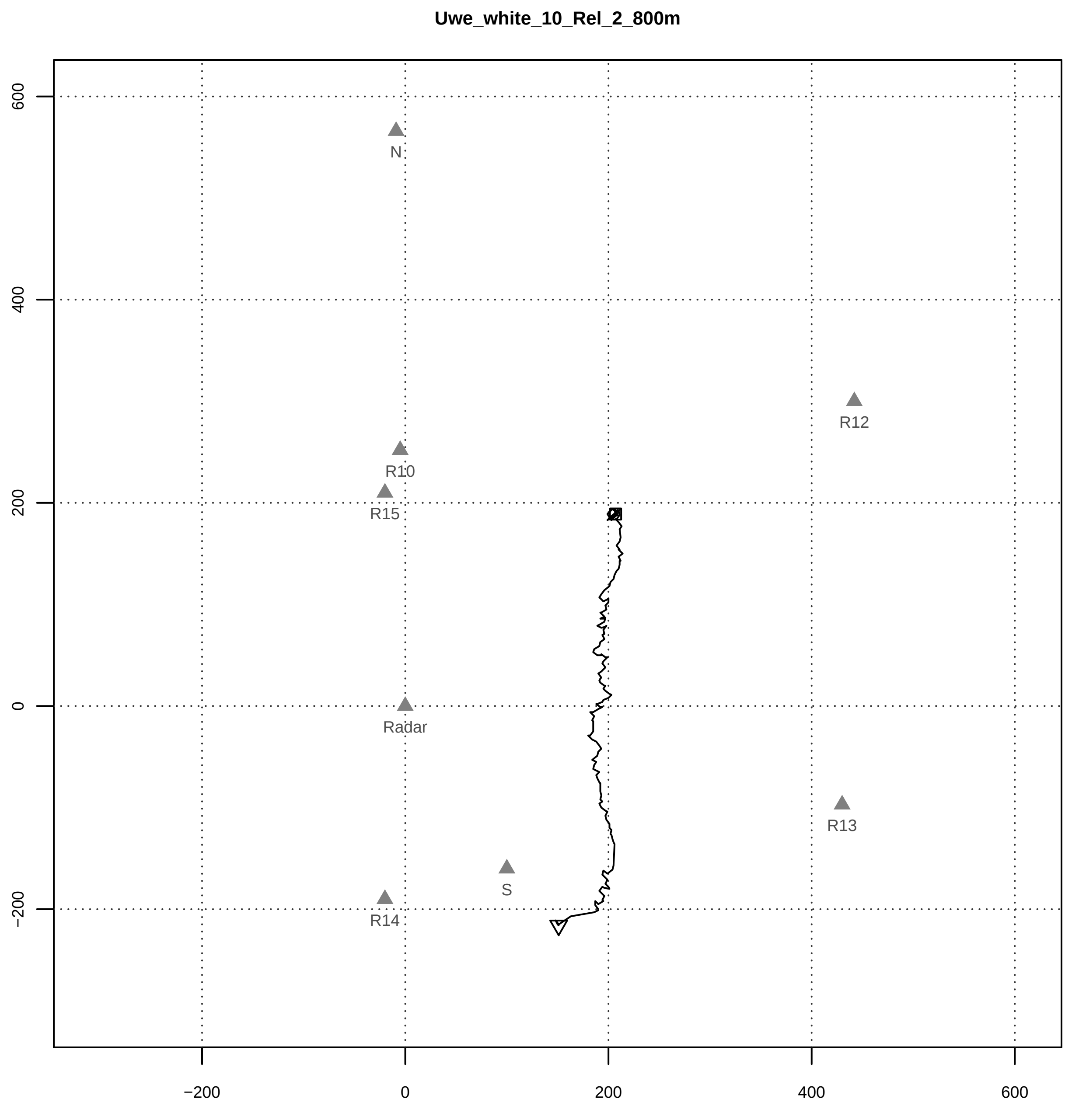
Uwe_white_10_Rel_2_800m
N
R12
R10
R15
Radar
R13
S
R14
600
400
200
0
−200
−200
0
200
400
600

Uwe_yellow_2_Rel_1_400m

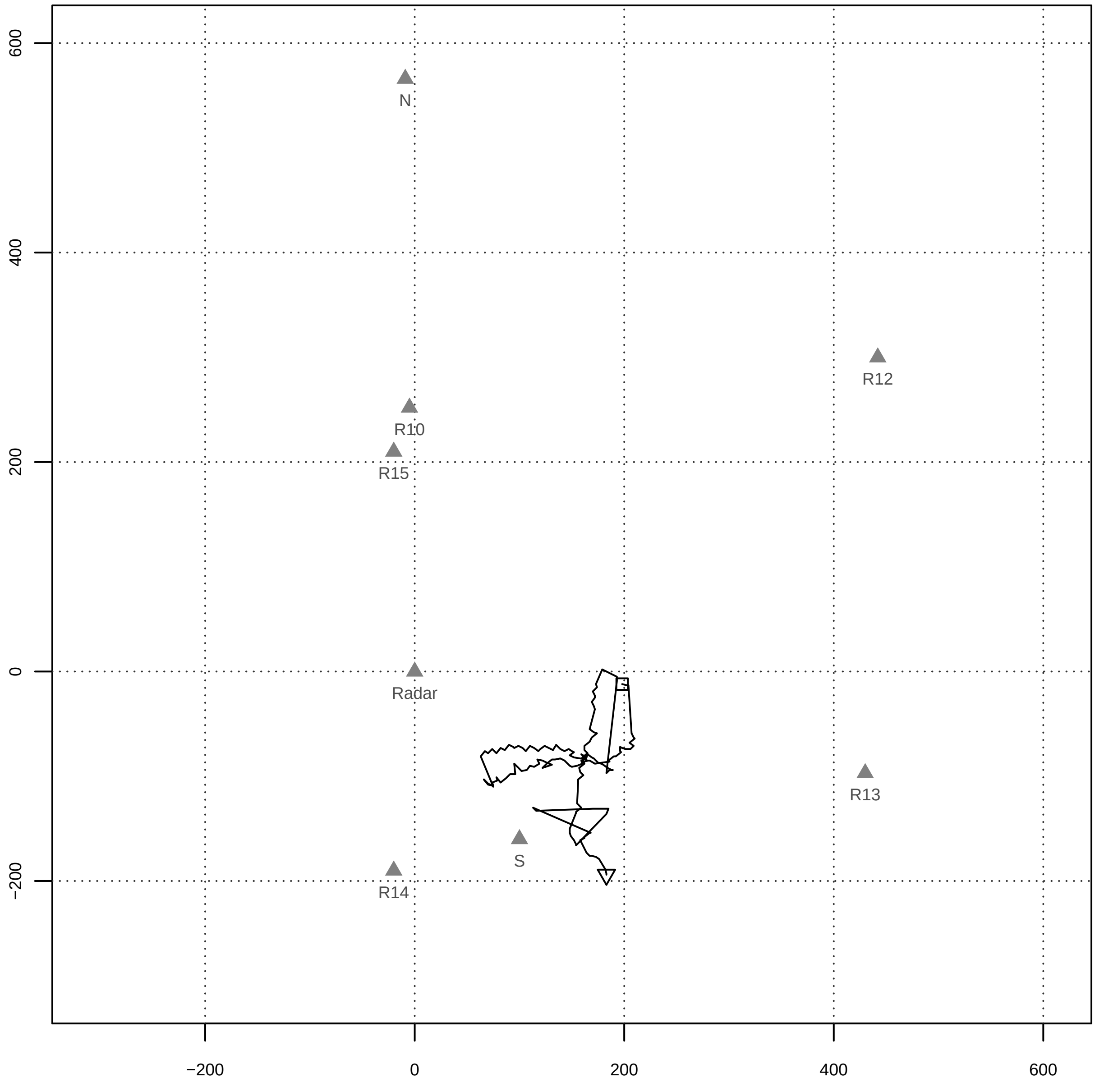

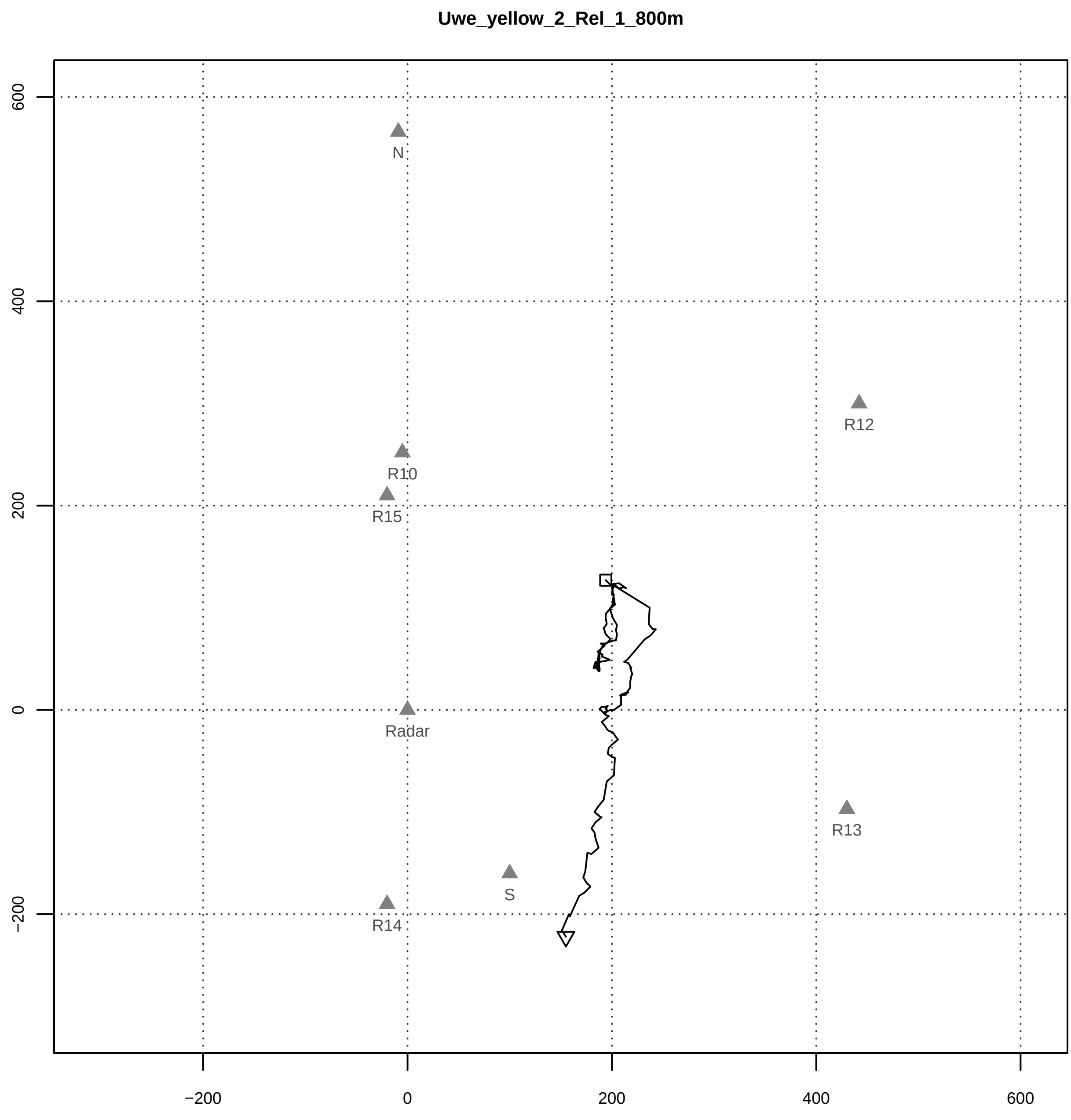

Uwe_yellow_2_Rel_1_800m
N
R12
R10
R15
Radar
R13
S
R14
−200
0
200
400
600

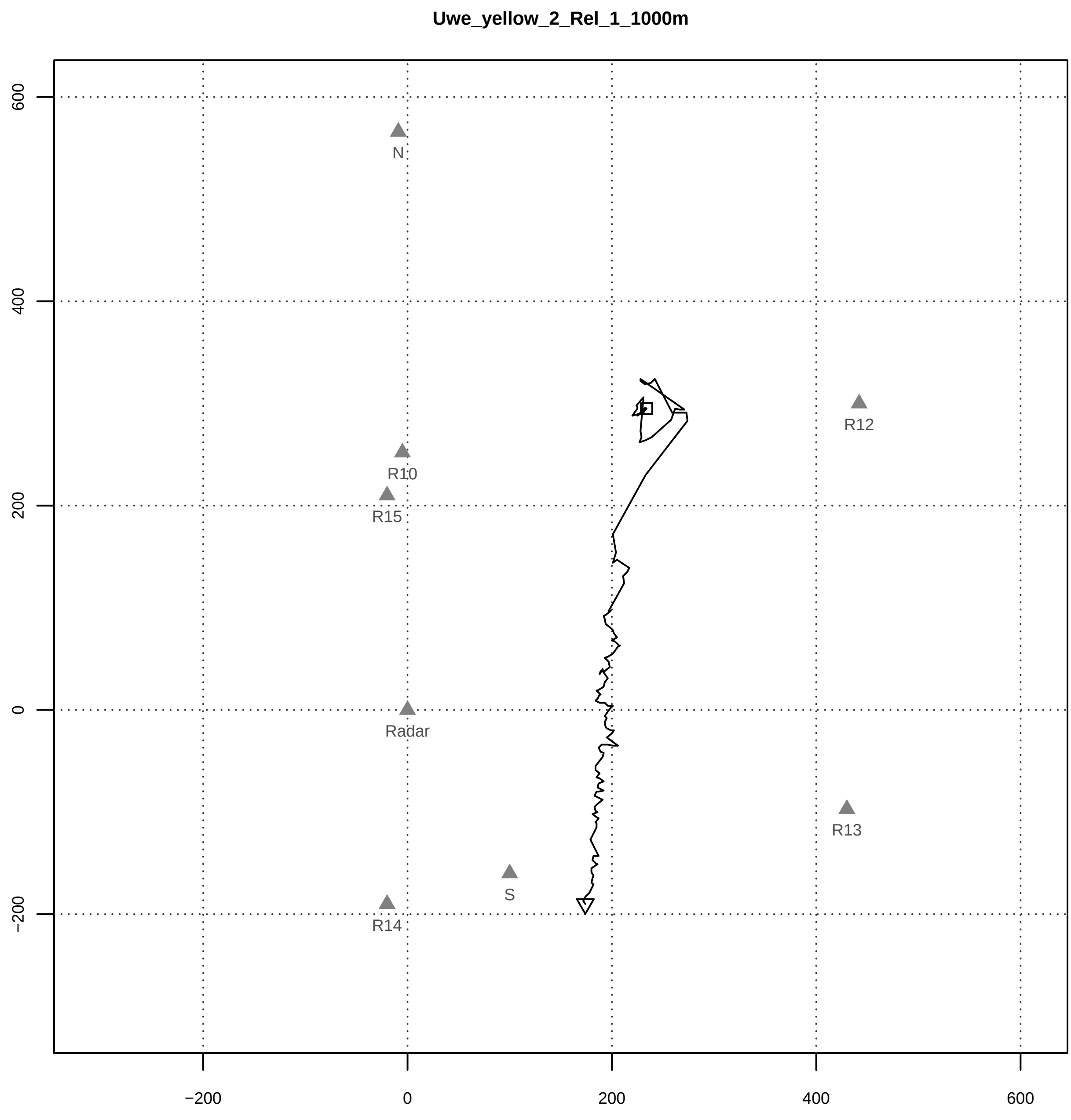

Uwe_yellow_2_Rel_1_1000m
600
400
200
0
−200
−200
0
200
400
600
N
R10
R15
Radar
S
R14
R12
R13

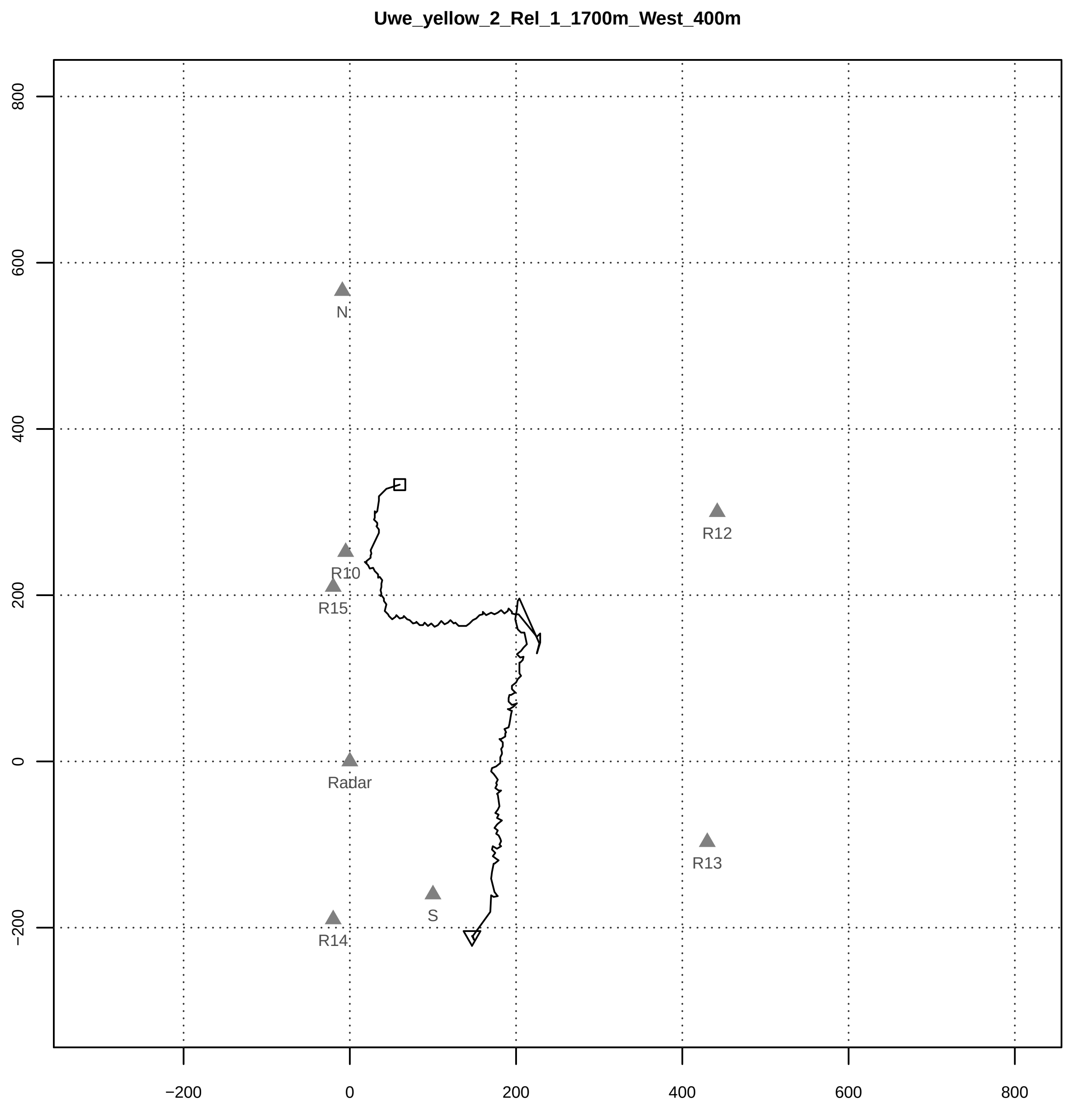

Uwe_yellow_2_Rel_1_1700m_West_400m
N
R10
R15
R12
Radar
R13
S
R14
−200
0
200
400
600
800

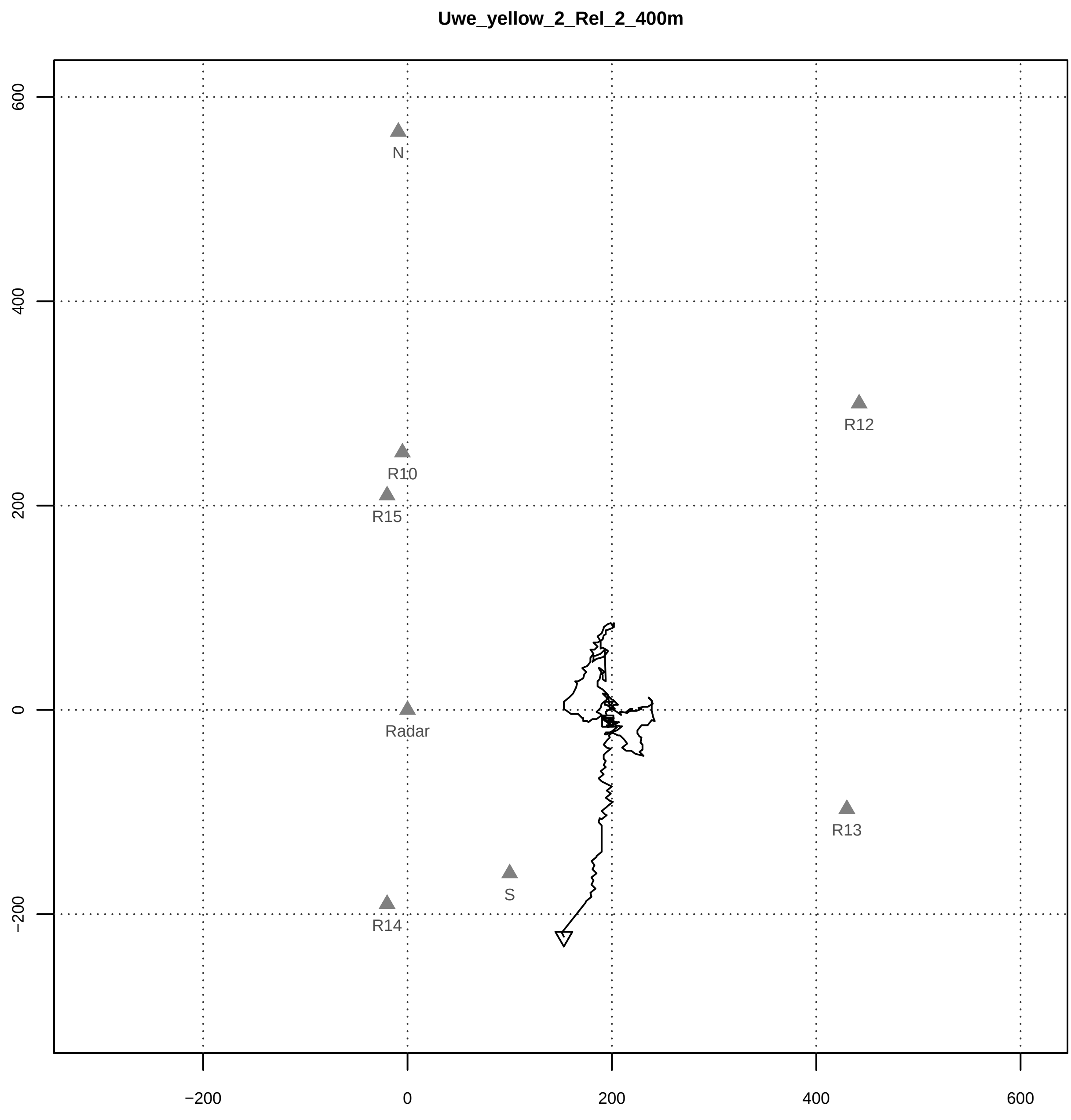

Uwe_yellow_2_Rel_2_400m
N
R12
R10
R15
Radar
R13
S
R14
600
400
200
0
−200
−200
0
200
400
600

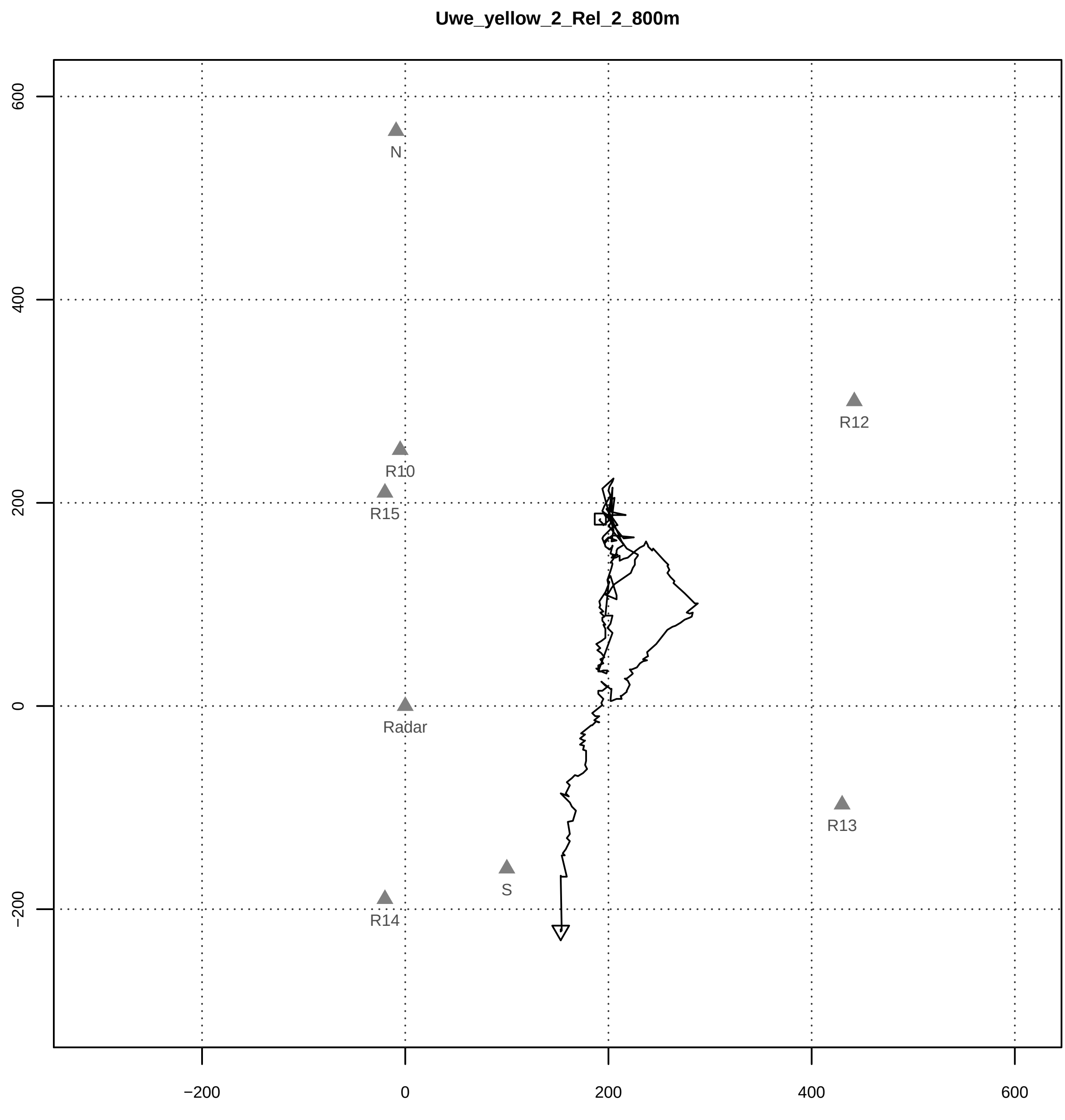

Uwe_yellow_2_Rel_2_800m
N
R12
R10
R15
Radar
R13
S
R14
600
400
200
0
−200
−200
0
200
400
600

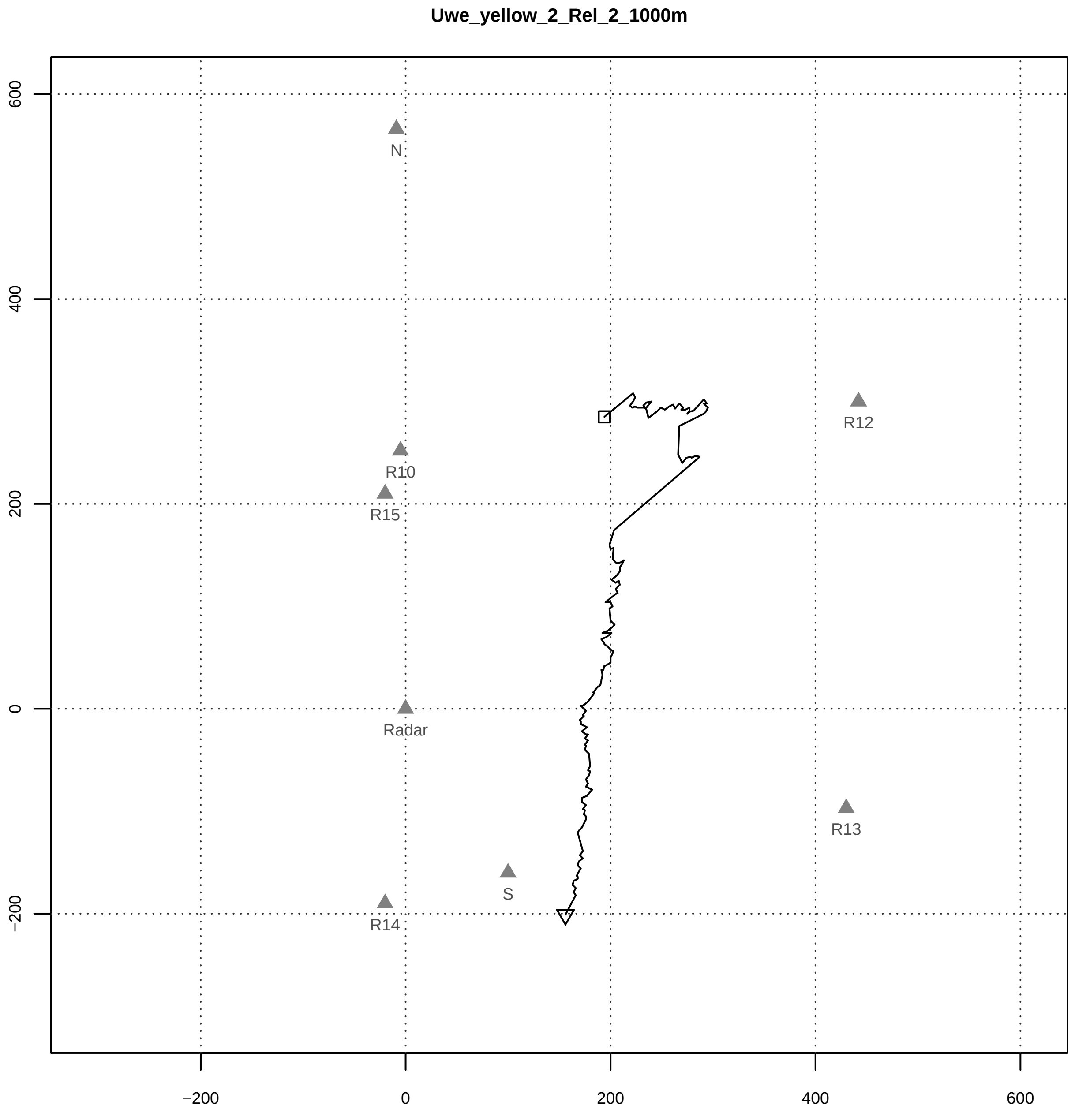

Uwe_yellow_2_Rel_2_1000m
N
R10
R15
R12
Radar
R13
S
R14
600
400
200
0
−200
−200
0
200
400
600

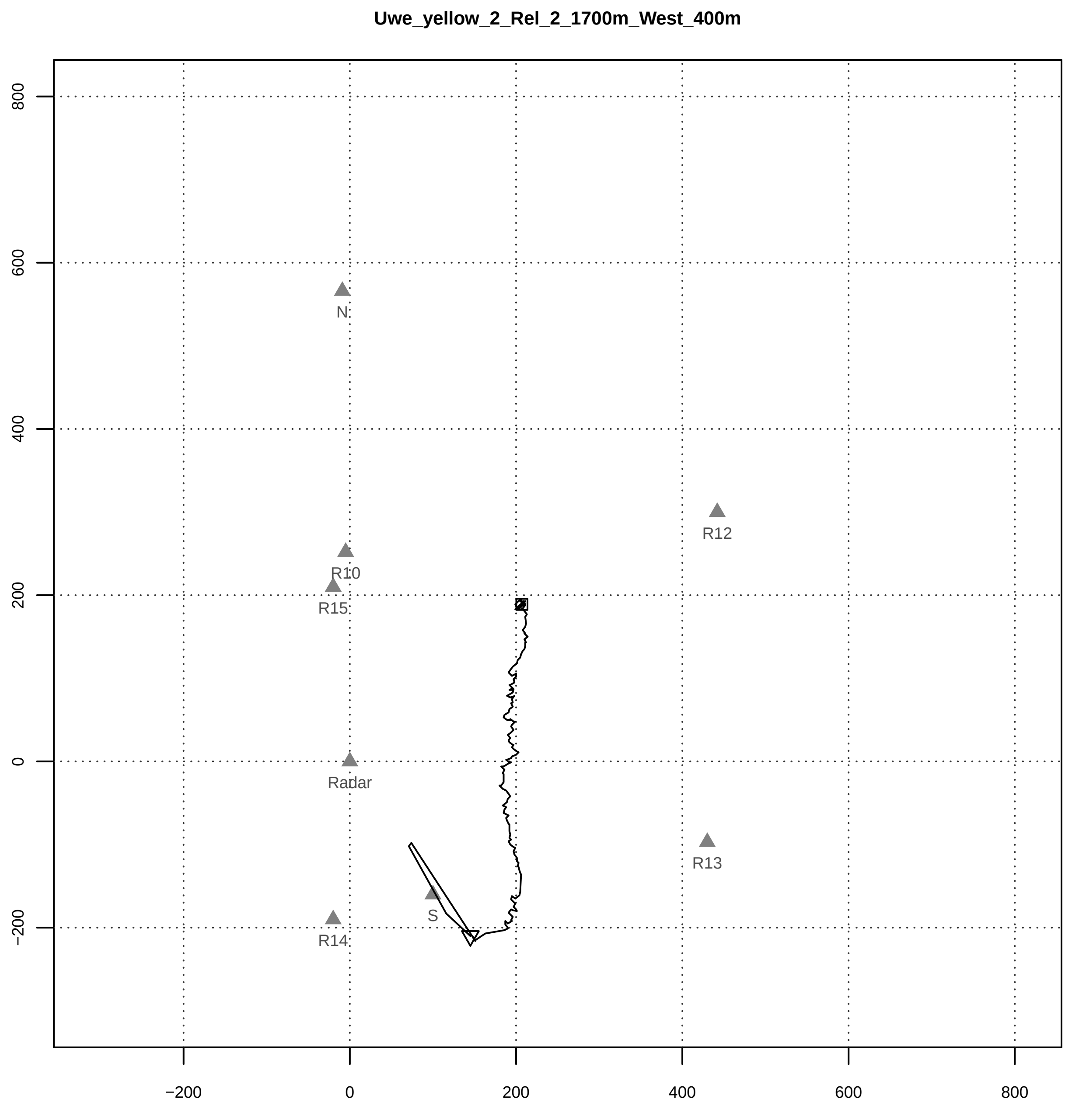

Uwe_yellow_2_Rel_2_1700m_West_400m
800
600
400
200
0
−200
−200
0
200
400
600
800
N
R12
R10
R15
Radar
R13
S
R14

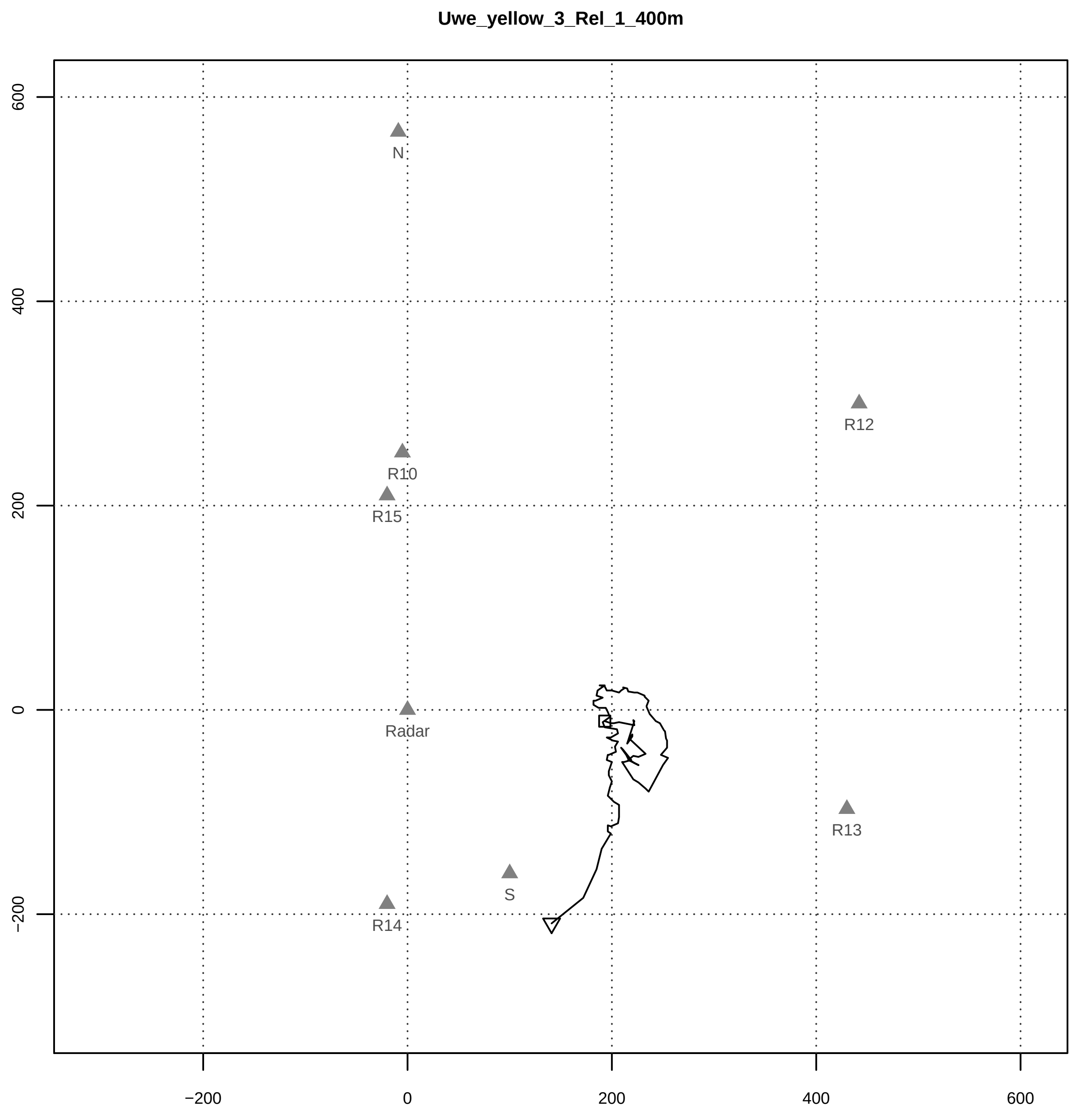

Uwe_yellow_3_Rel_1_400m
N
R12
R10
R15
Radar
R13
S
R14
600
400
200
0
−200
−200
0
200
400
600

Uwe_yellow_10_Rel_1_400m

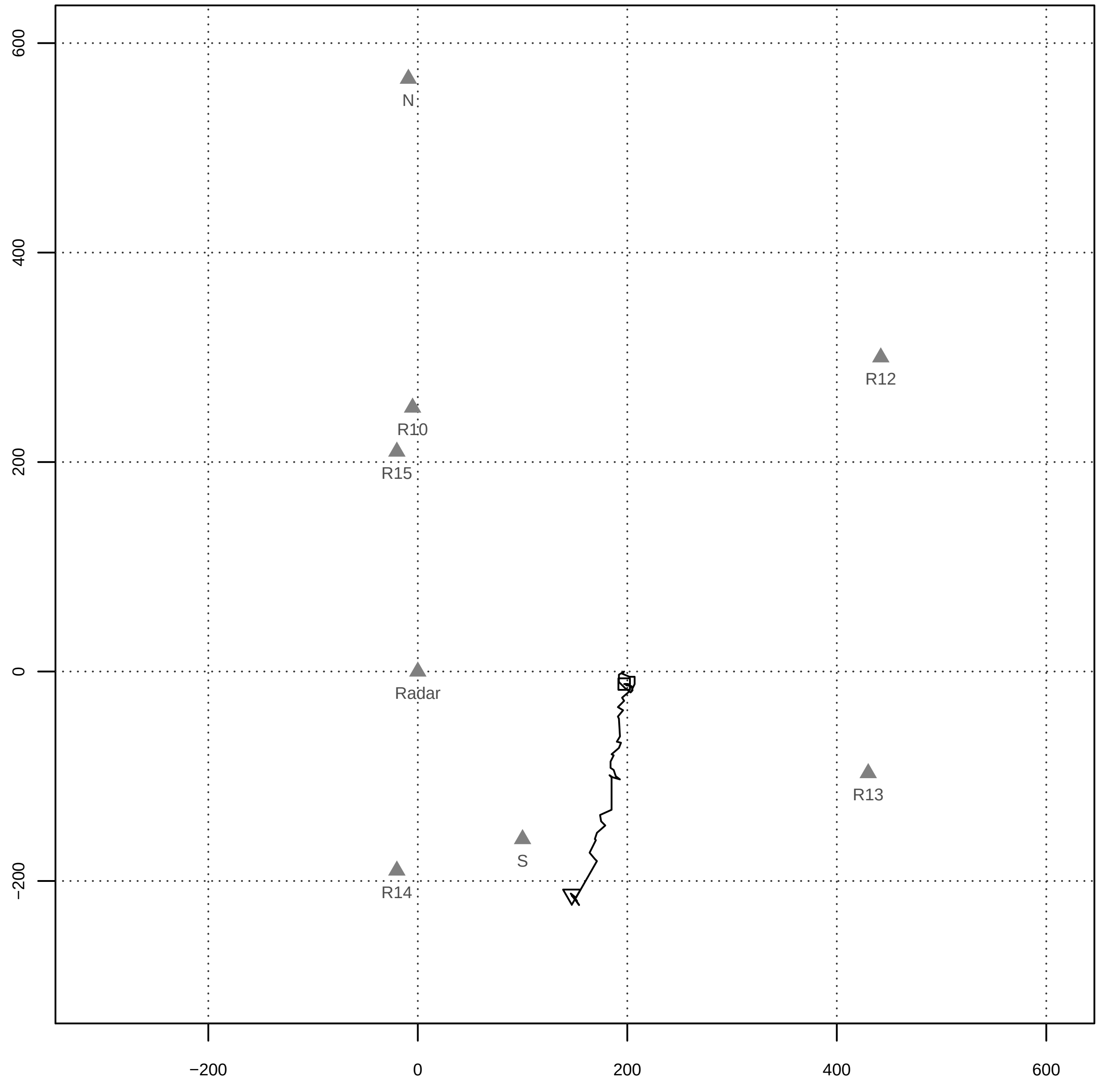

Uwe_yellow_10_Rel_2_400m

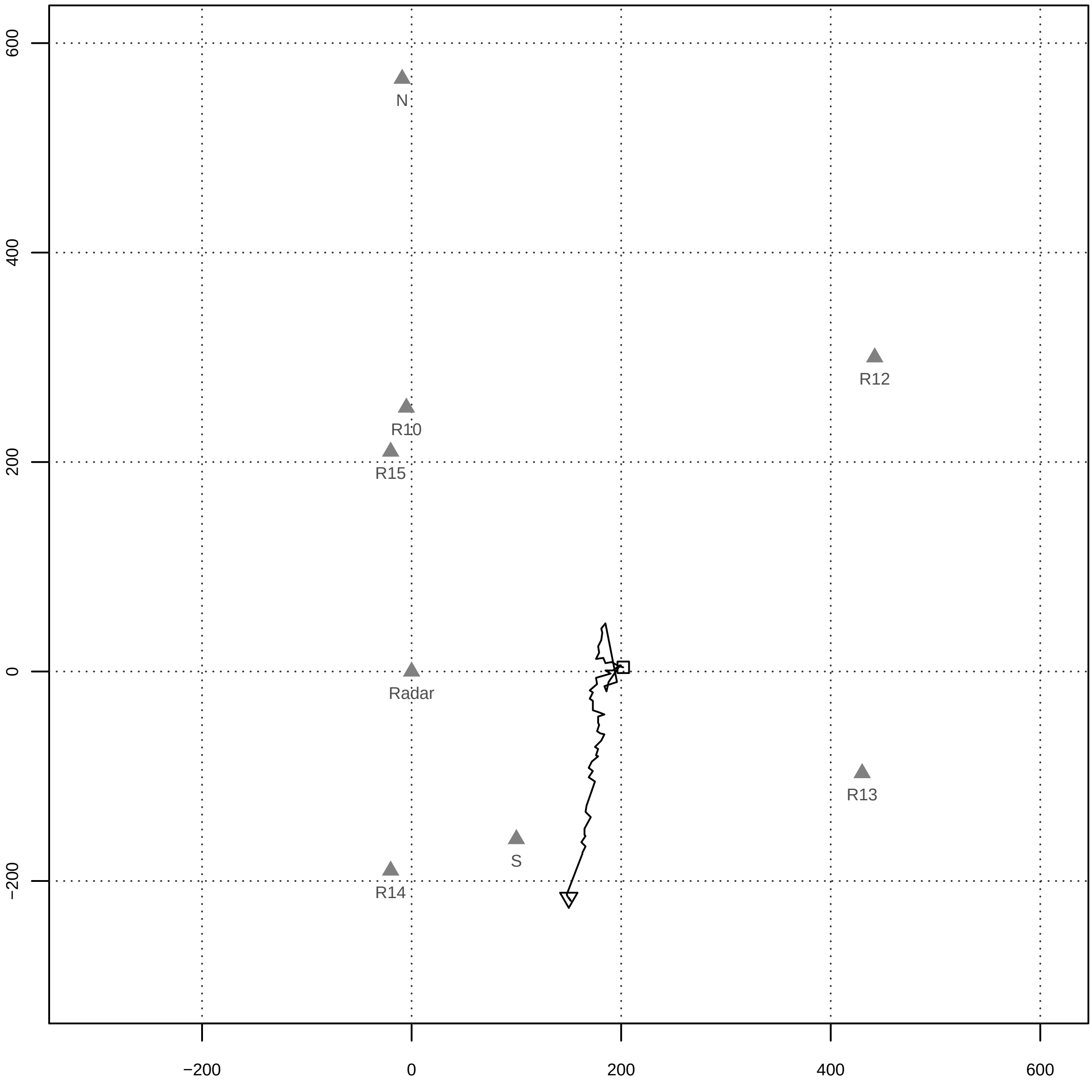

Uwe_yellow_11_Rel_1_400m

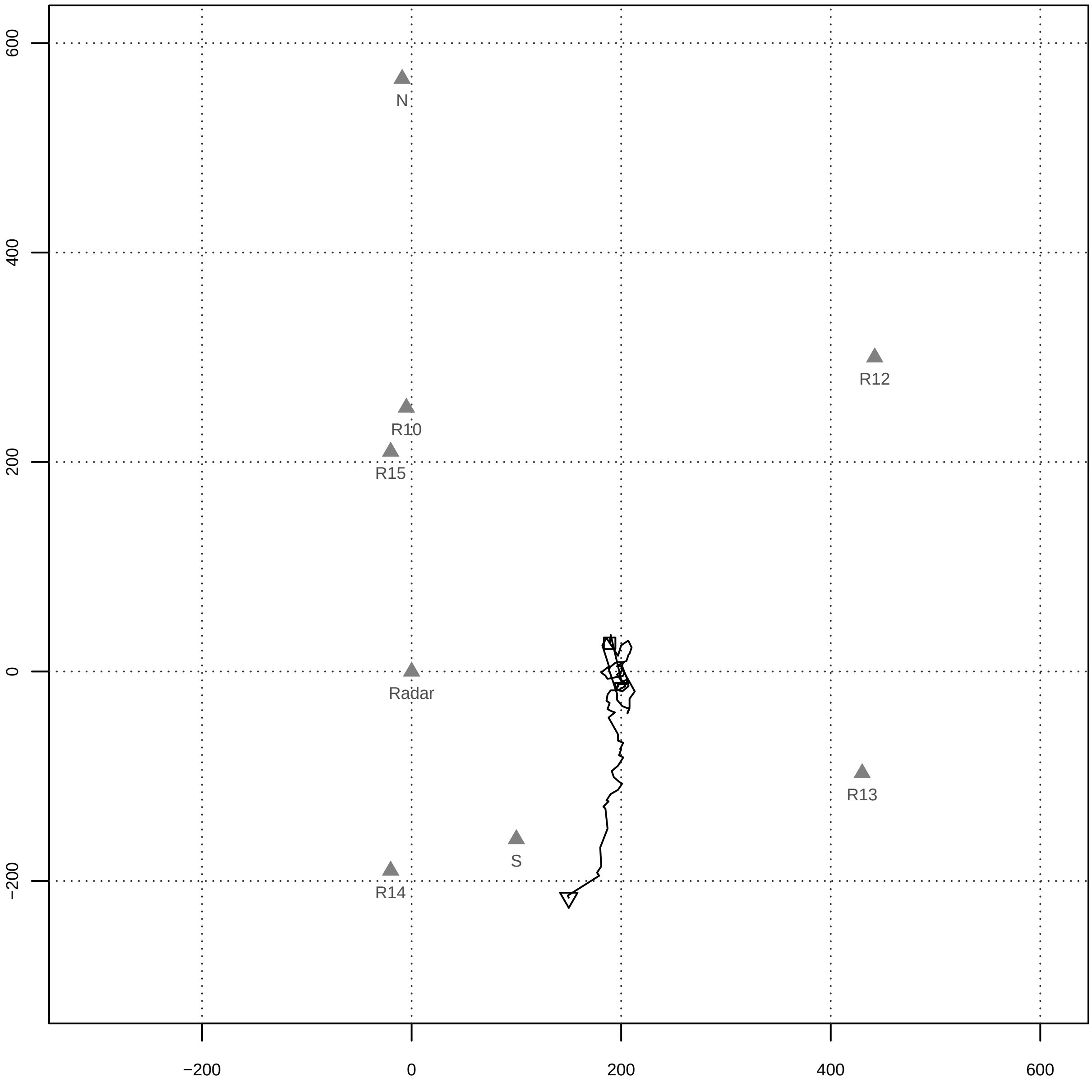

Uwe_yellow_12_Rel_1_400m

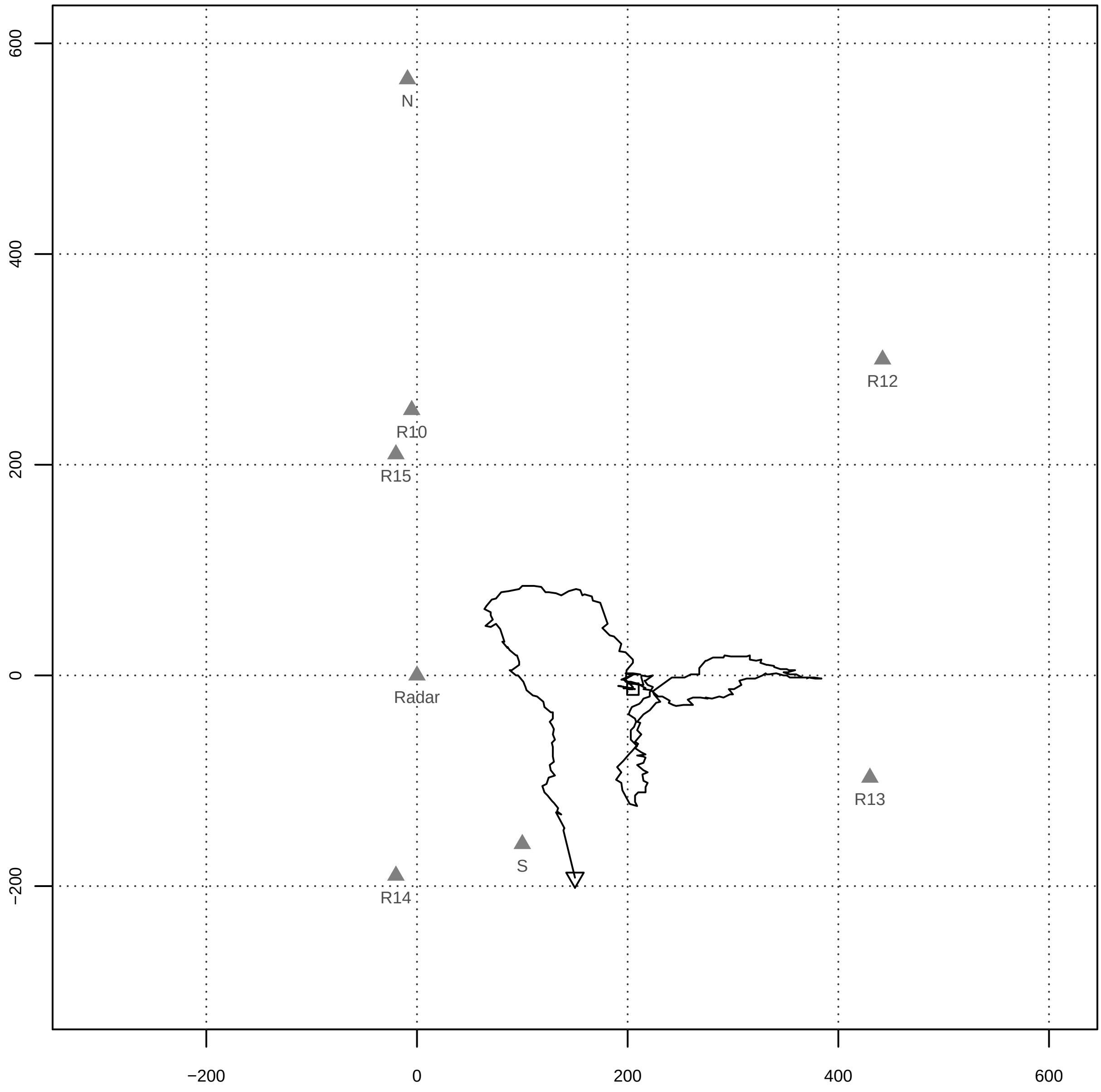

Uwe_yellow_12_Rel_1_800m

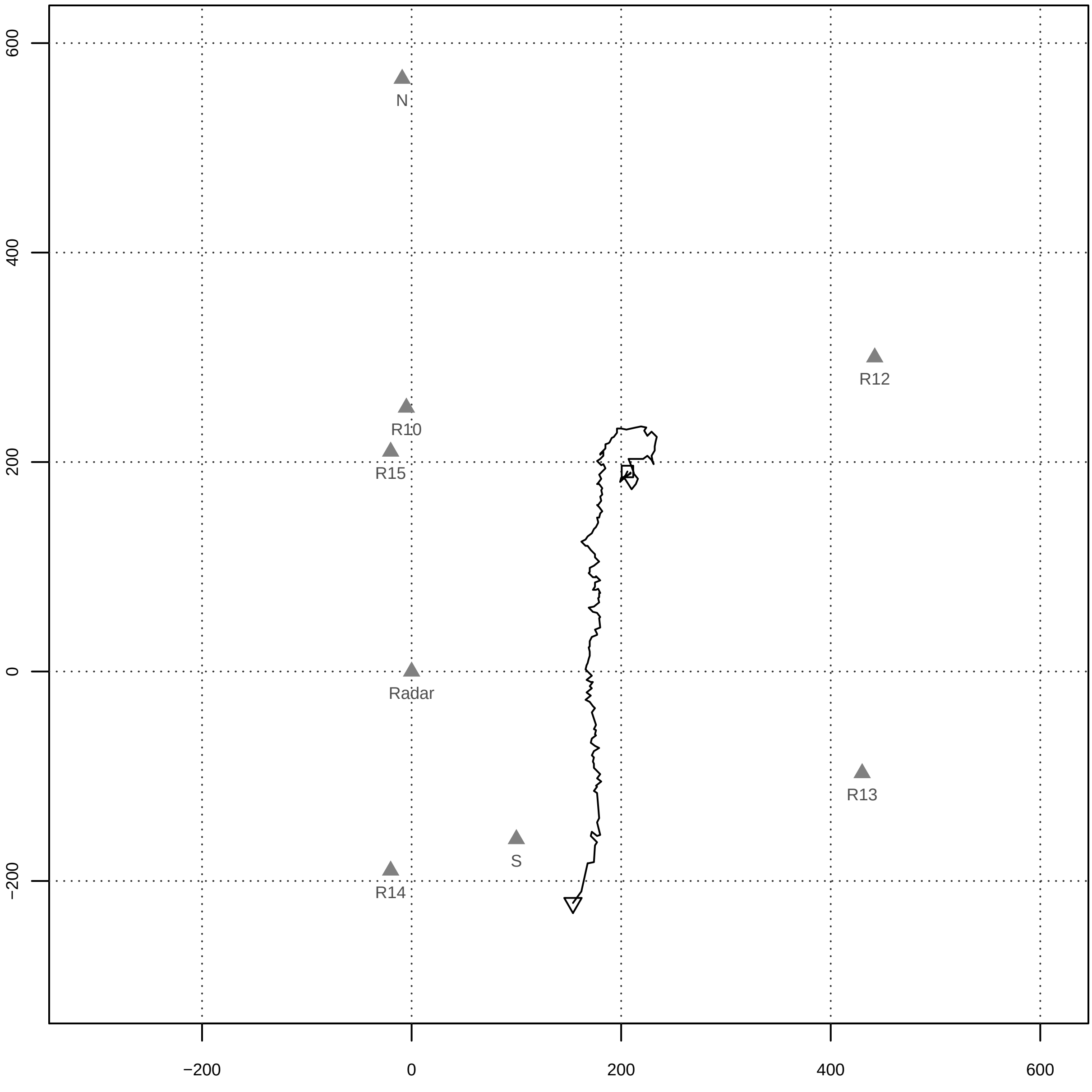

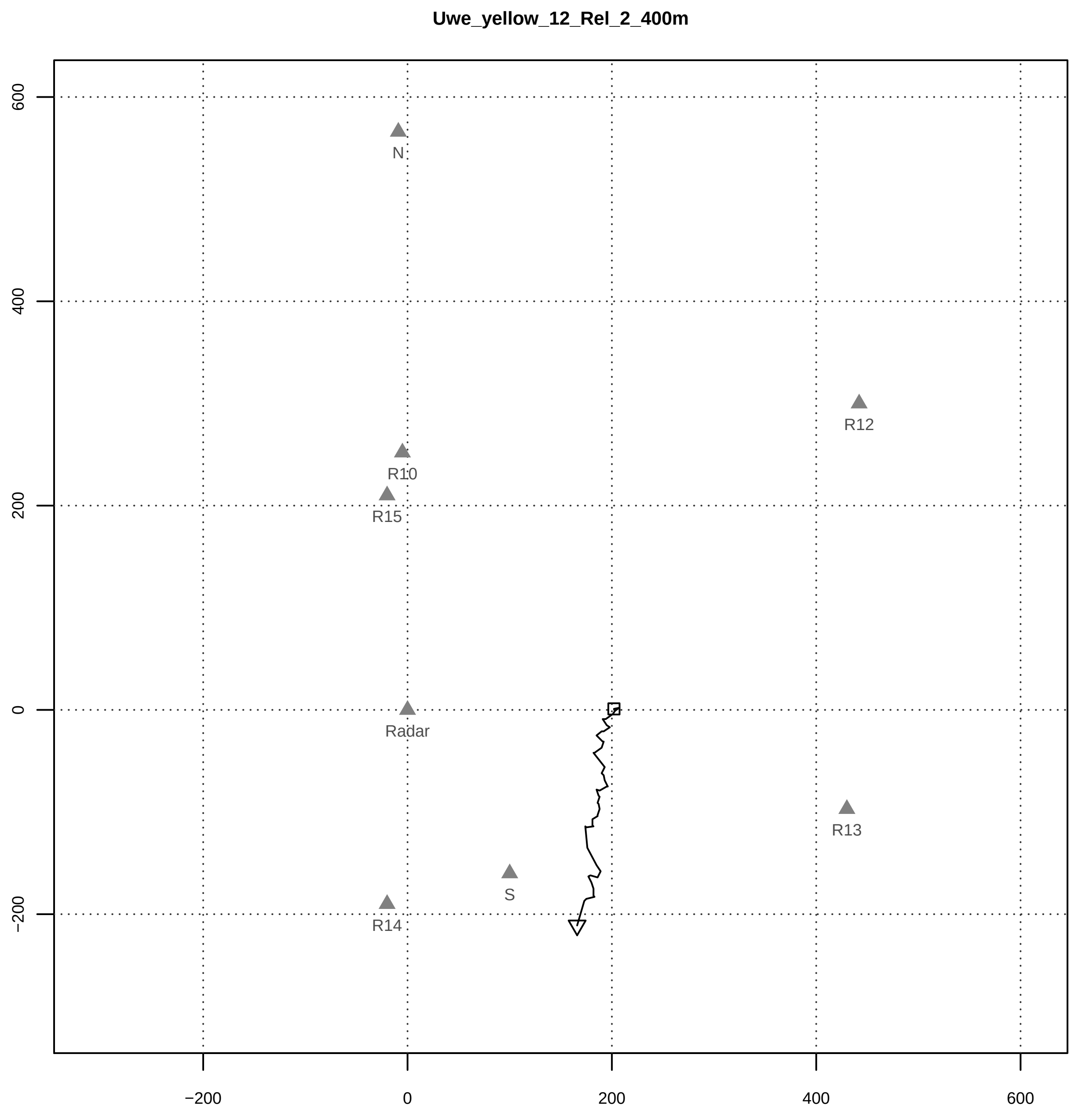
Uwe_yellow_12_Rel_2_400m
N
R12
R10
R15
Radar
R13
S
R14
600
400
200
0
−200
−200
0
200
400
600

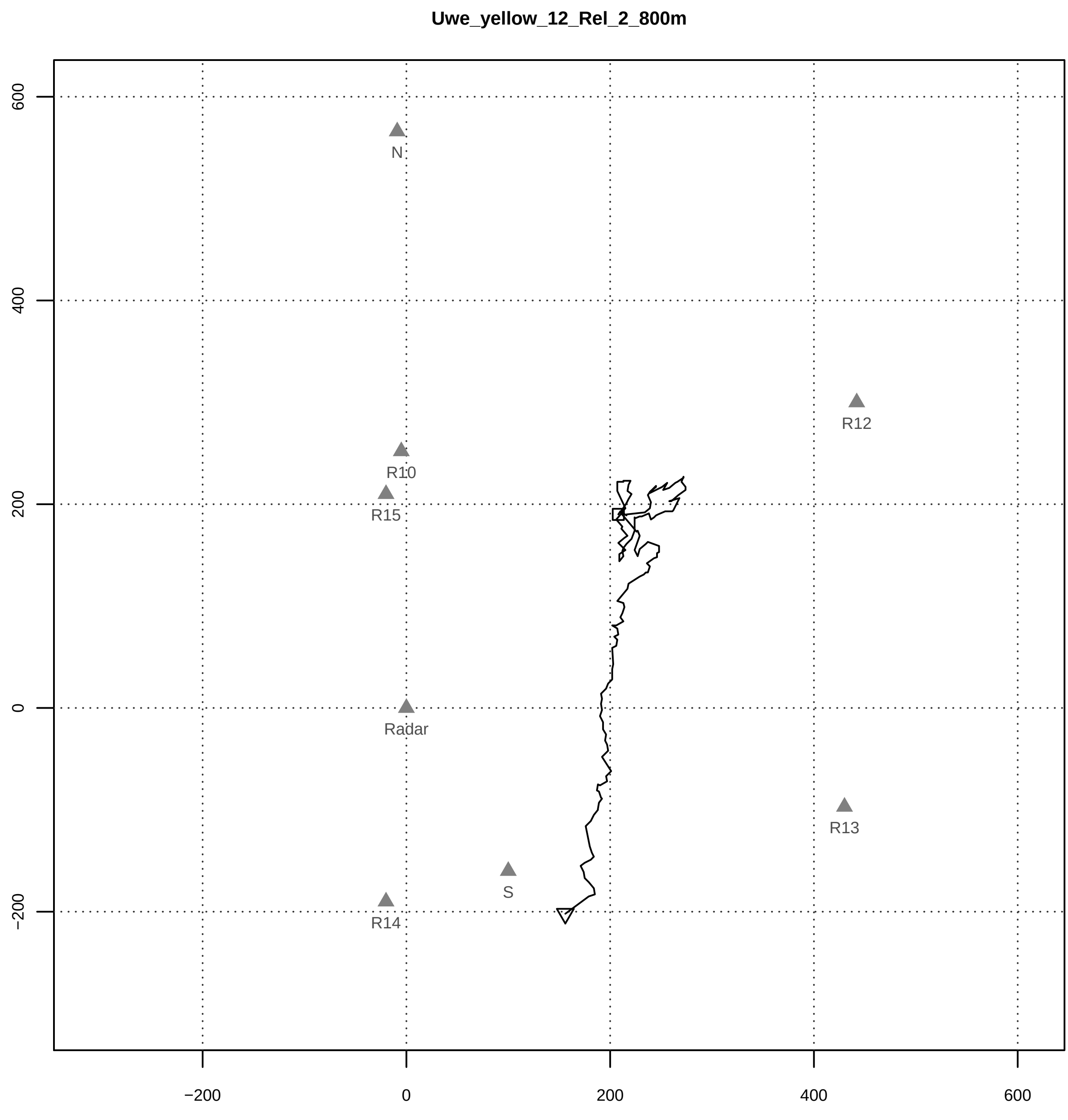
Uwe_yellow_12_Rel_2_800m
N
R12
R10
R15
Radar
R13
S
R14
600
400
200
0
−200
−200
0
200
400
600

Uwe_yellow_12_Rel_3_400m

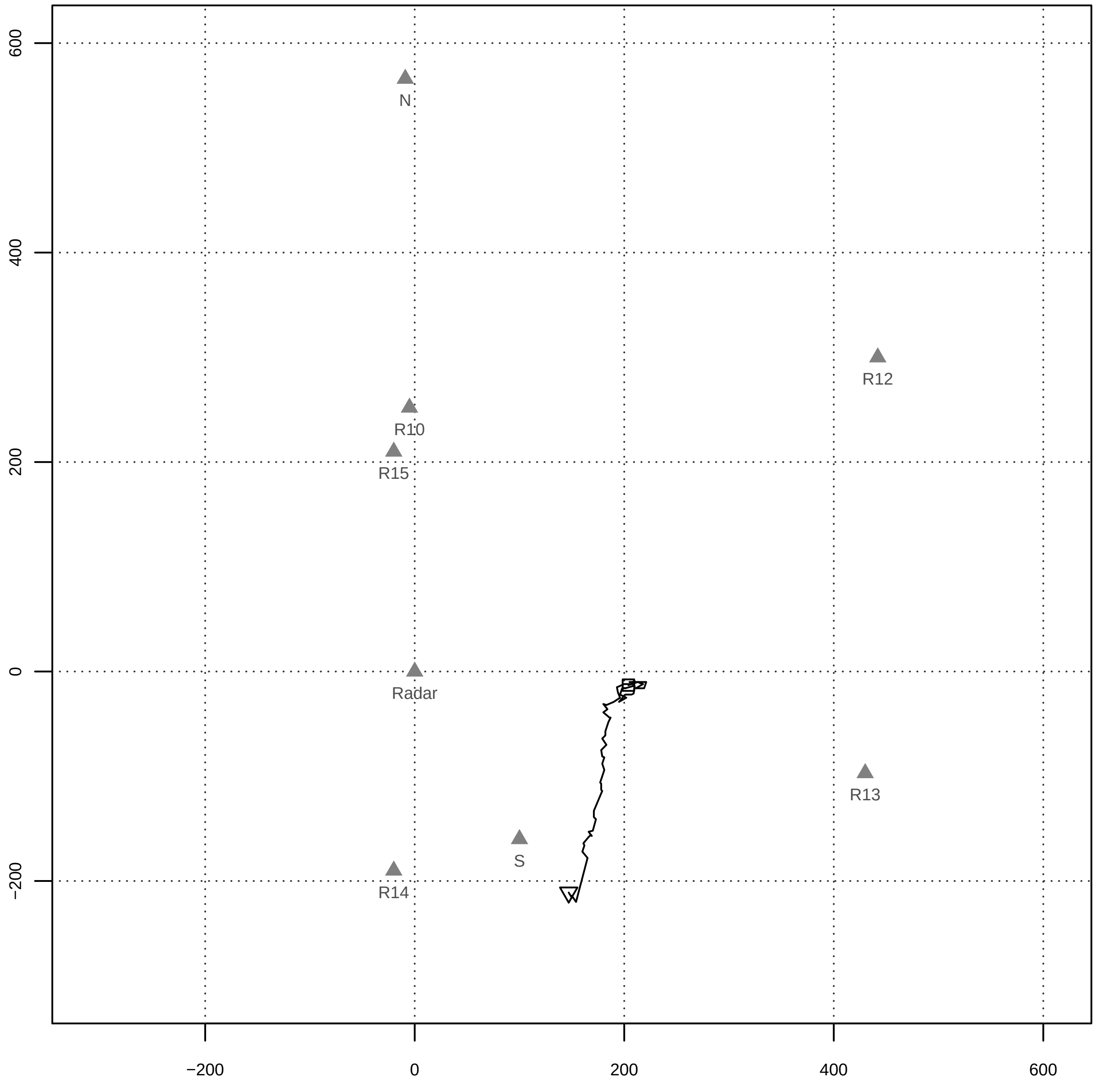

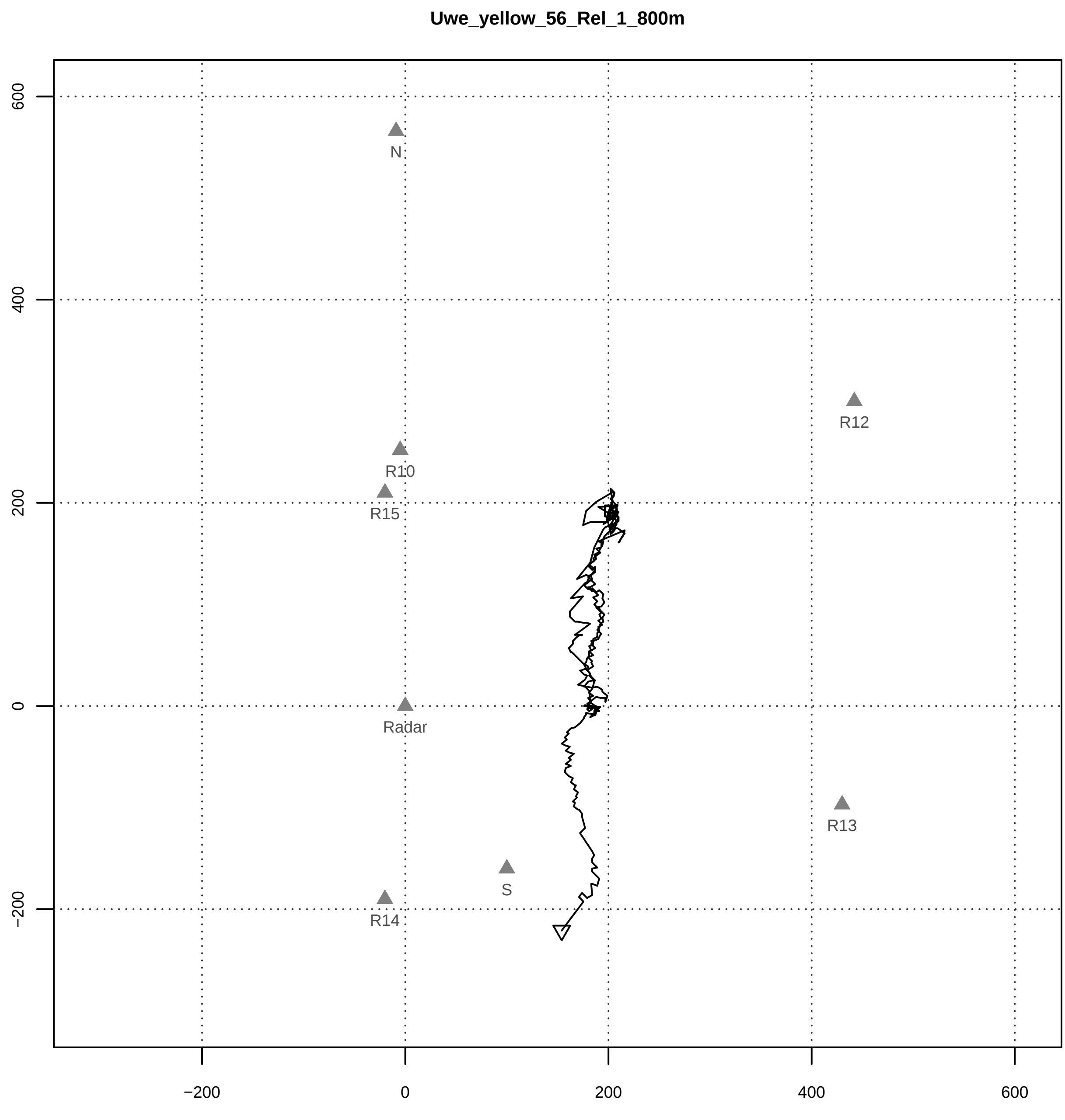

Uwe_yellow_56_Rel_1_800m
N
R10
R15
R12
Radar
R13
S
R14
600
400
200
0
−200
−200
0
200
400
600

Uwe_yellow_56_Rel_1_1200m−p1

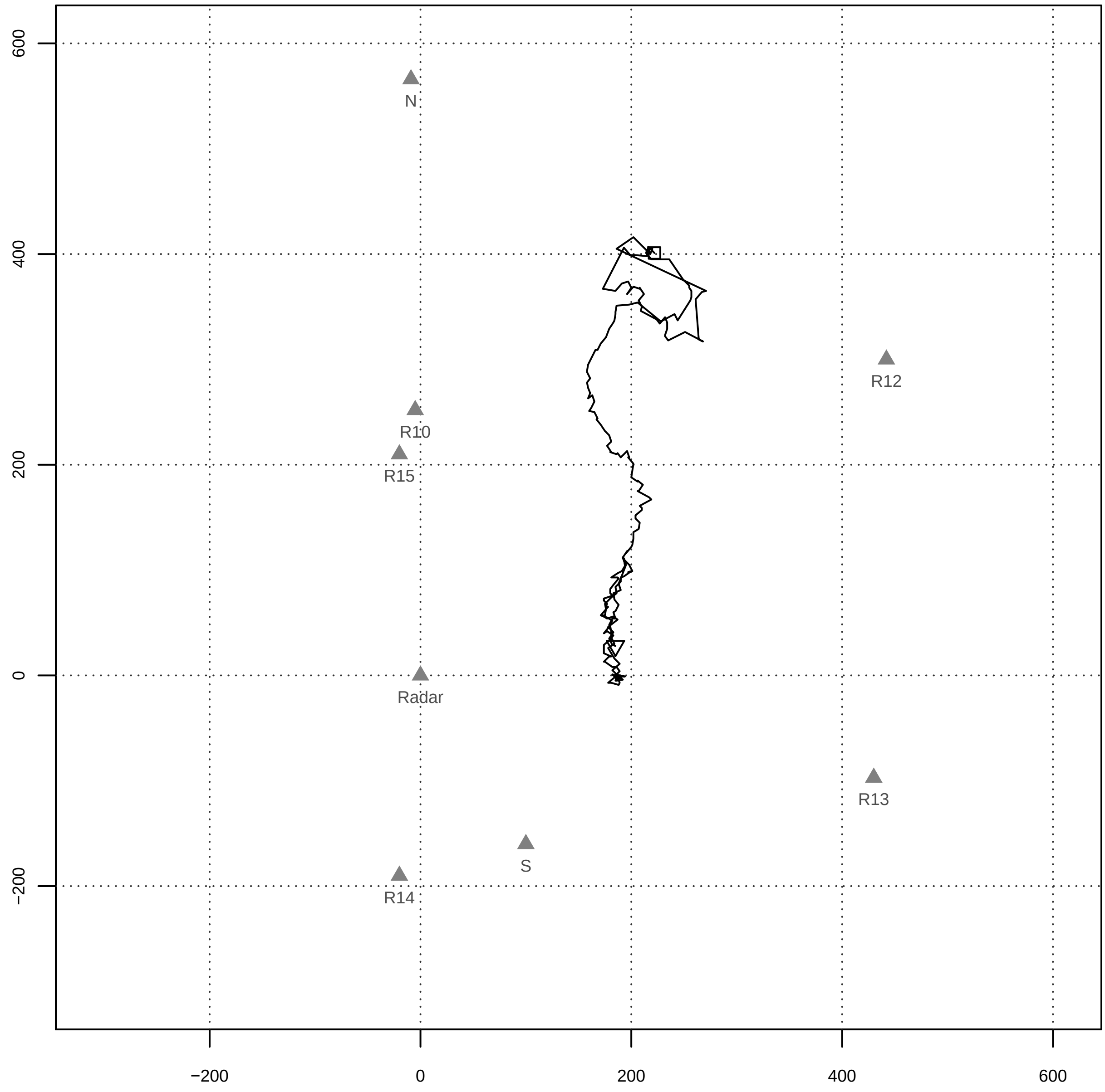

Uwe_yellow_57_Rel_1_800m

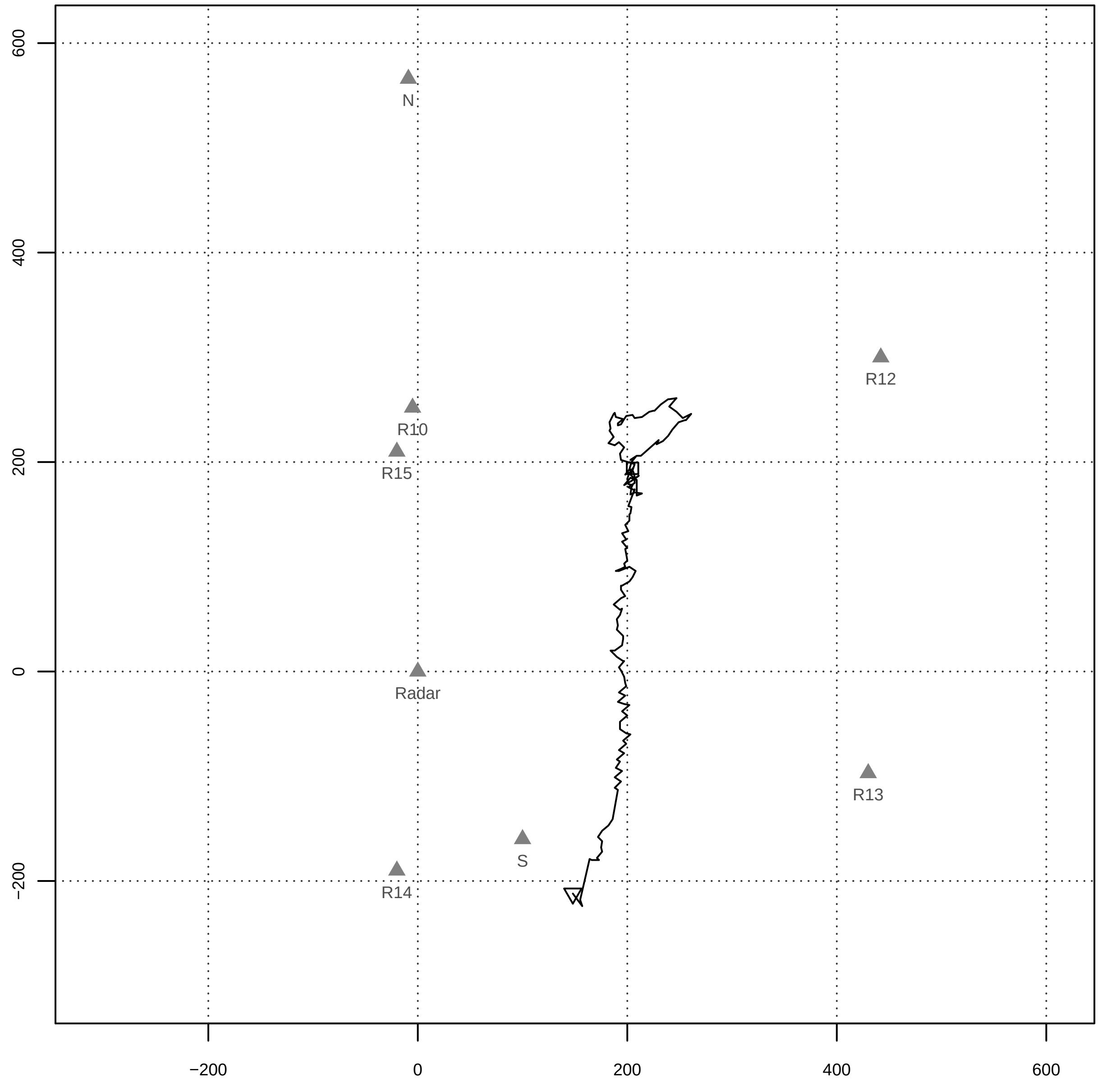

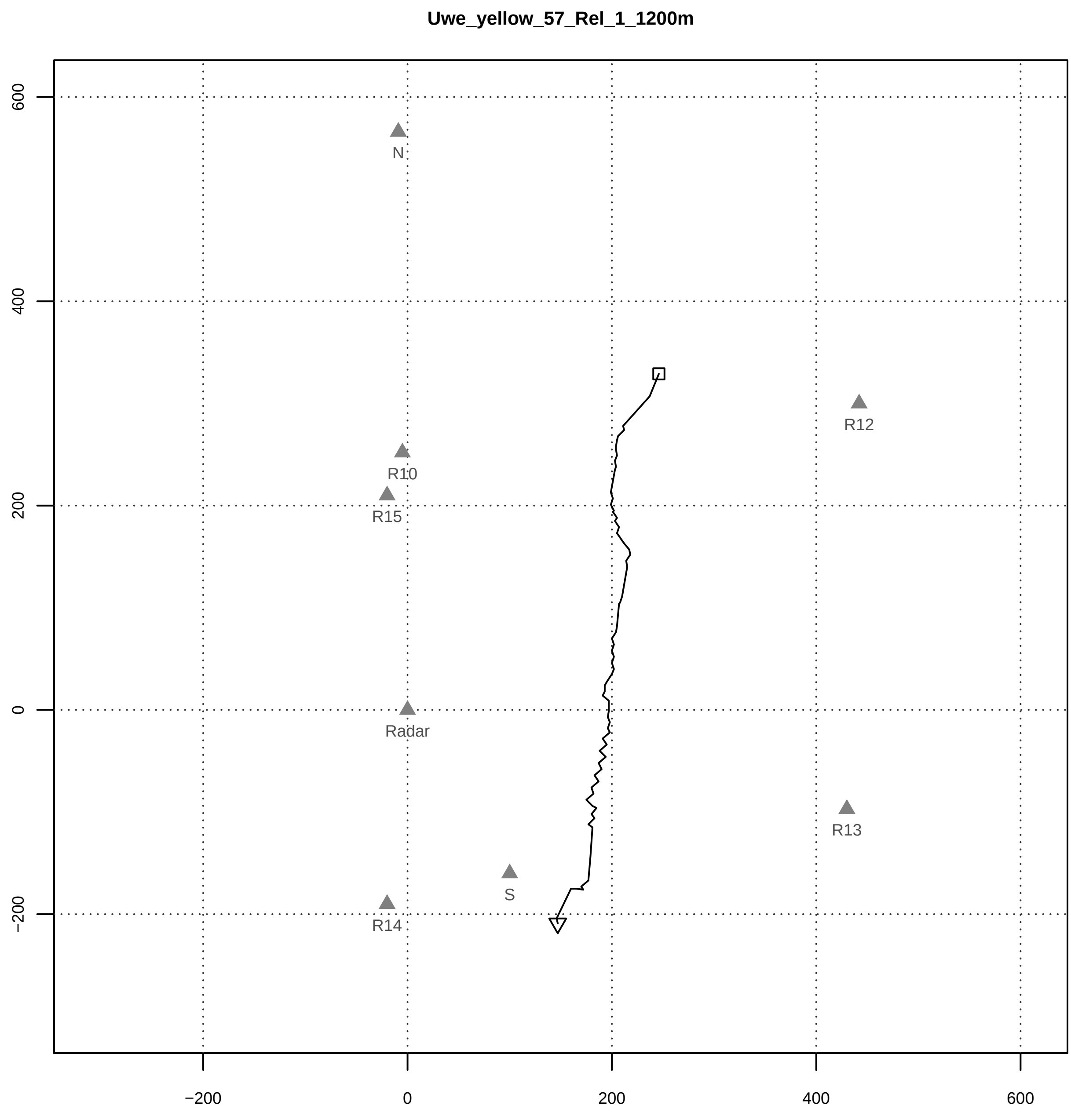
Uwe_yellow_57_Rel_1_1200m
N
R12
R10
R15
Radar
R13
S
R14
−200
0
200
400
600
−200
0
200
400
600

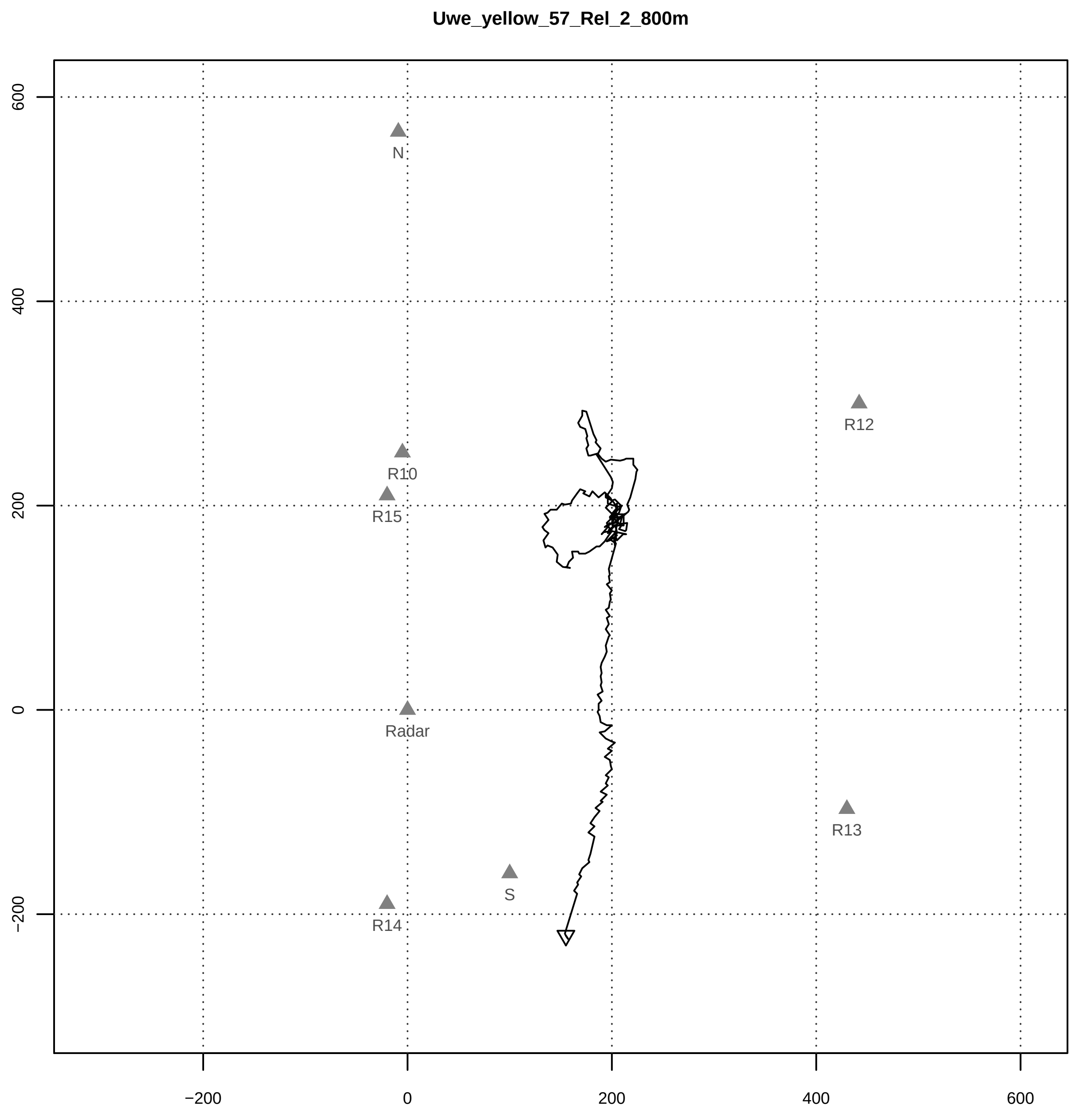

Uwe_yellow_57_Rel_2_800m
N
R12
R10
R15
Radar
R13
S
R14
−200
0
200
400
600

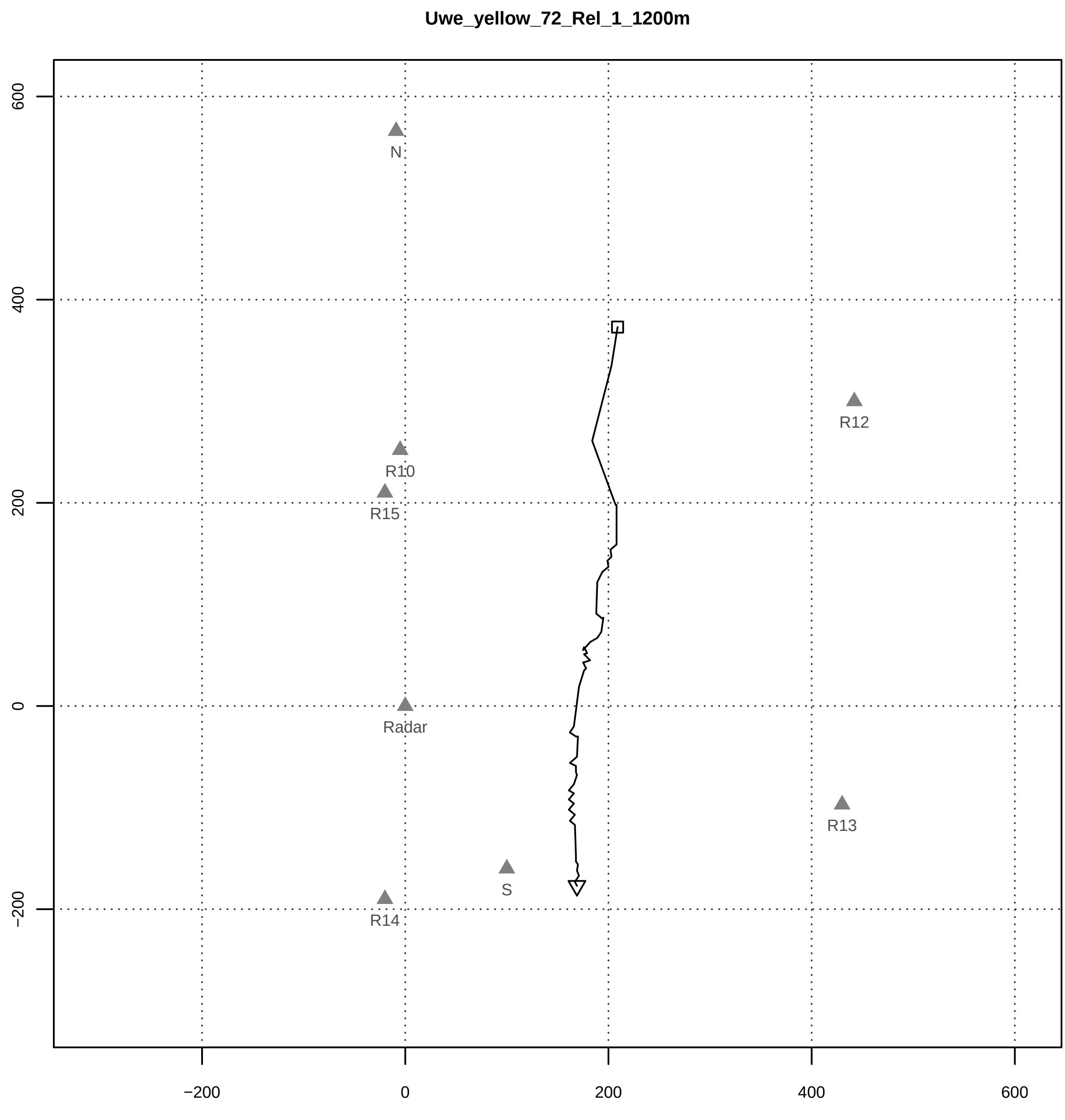
Uwe_yellow_72_Rel_1_1200m
N
R10
R15
R12
Radar
R13
S
R14
−200
0
200
400
600

Uwe_yellow_72_Rel_2_800m

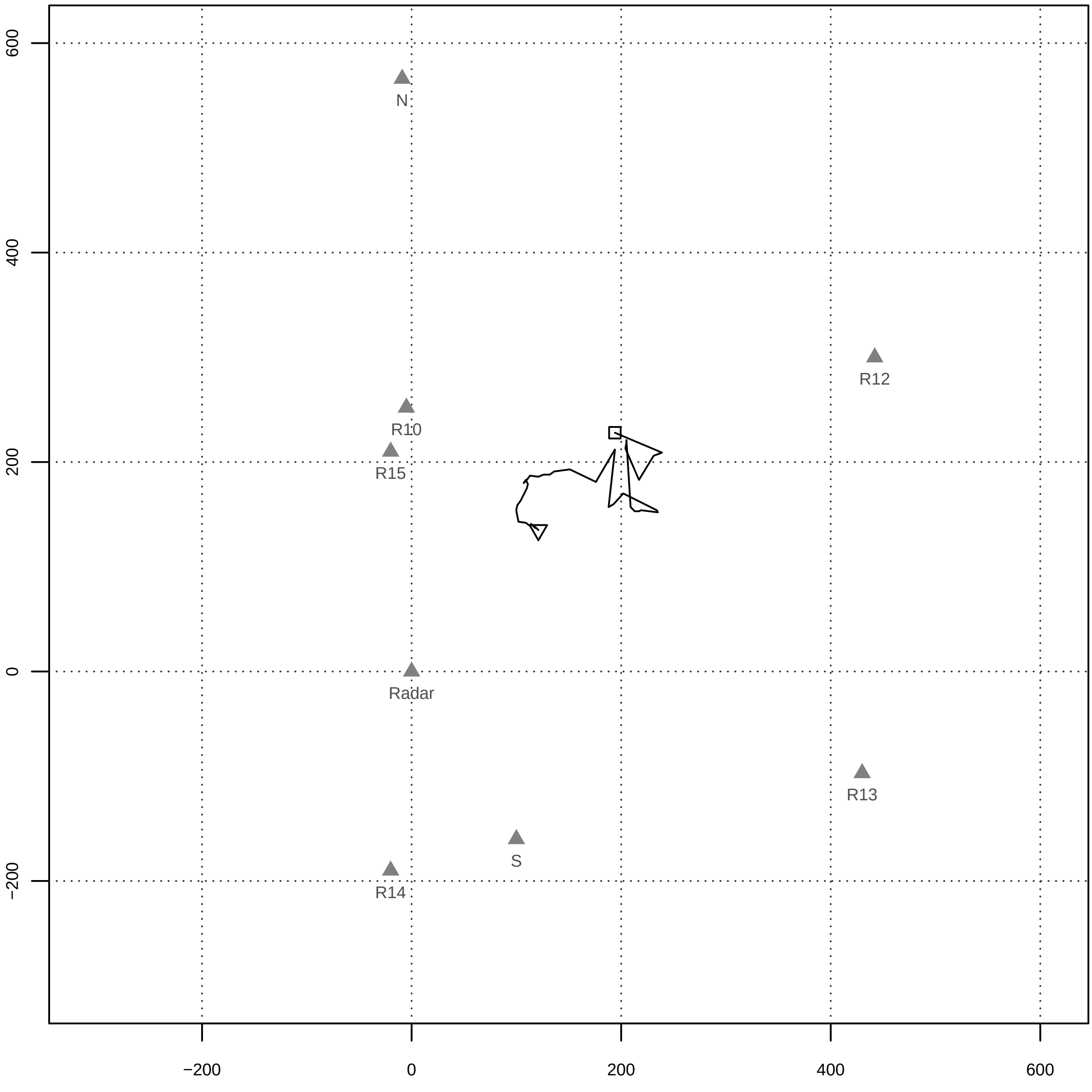

Uwe_yellow_87_Rel_1_800m

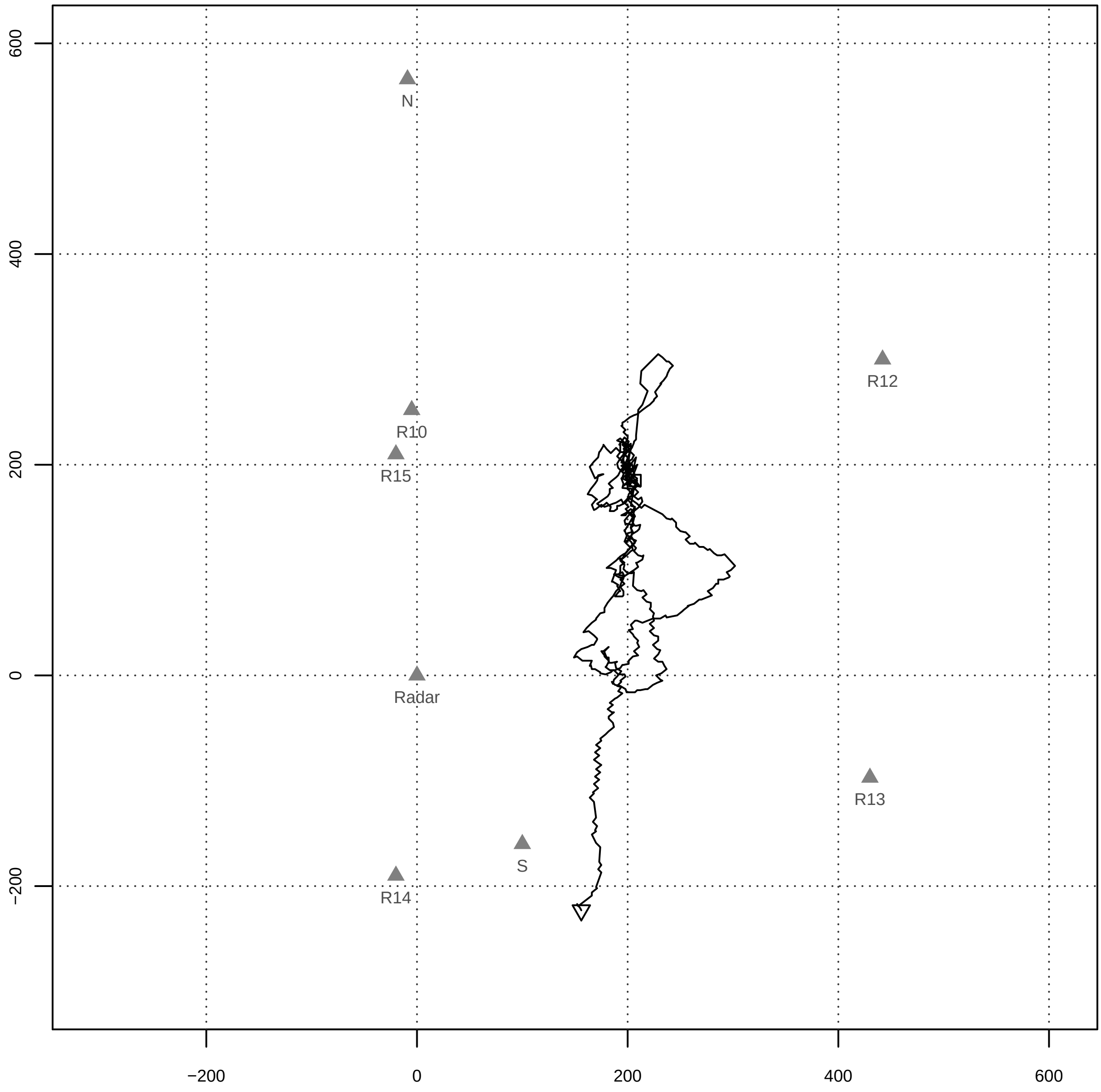

Uwe_yellow_87_Rel_1_1200m

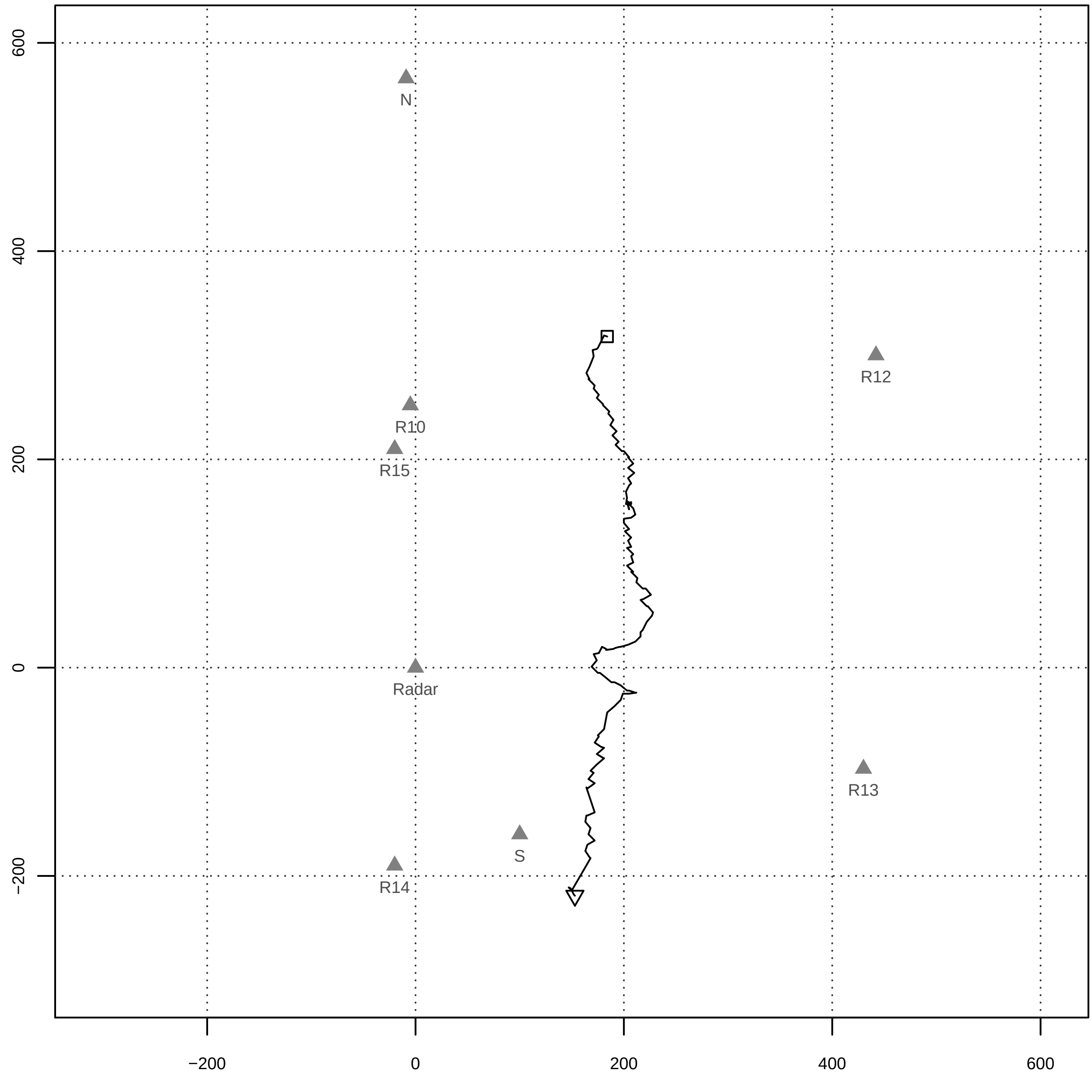

Supplement: Supplementary file 8 [file Data_Sheet_1.PDF]
